# ﻿Vascular plant biodiversity of Katannilik Territorial Park, Kimmirut and vicinity on Baffin Island, Nunavut, Canada: an annotated checklist of an Arctic flora

**DOI:** 10.3897/phytokeys.217.90573

**Published:** 2023-01-04

**Authors:** Jeffery M. Saarela, Paul C. Sokoloff, Lynn J. Gillespie, Roger D. Bull

**Affiliations:** 1 Centre for Arctic Knowledge and Exploration and Botany Section, Canadian Museum of Nature, Ottawa, Ontario, Canada Canadian Museum of Nature Ottawa Canada

**Keywords:** Canadian Heritage River, floristics, herbarium specimens, Joseph Dewey Soper, Malte Oscar Malte, Nicholas Polunin, Soper River

## Abstract

The Arctic ecozone is undergoing a rapid transformation in response to climate change. Establishing a baseline of current Arctic biodiversity is necessary to be able to track changes in species diversity and distribution over time. Here, we report a vascular plant floristic study of Katannilik Territorial Park, Kimmirut and vicinity within Circumpolar Arctic Bioclimate Subzone D on southern Baffin Island, Nunavut, Canada. We compiled a dataset of 1596 collections gathered in the study area throughout the last century, including 838 we made in 2012. The vascular flora comprises 35 families, 98 genera, 211 species, two nothospecies and seven infraspecific taxa. We newly recorded 51 taxa in 22 families in the study area: *Erigeroneriocephalus*, *Taraxacumholmenianum* (Asteraceae), *Drabaarctica*, *D.fladnizensis*, *D.lactea* (Brassicaceae), *Campanularotundifolia* (Campanulaceae), *Arenarialongipedunculata*, Honckenyapeploidessubsp.diffusa, *Sabulinarossii*, Sileneuralensissubsp.uralensis, *Viscariaalpina* (Caryophyllaceae), Carexbrunnescenssubsp.brunnescens, *C.krausei*, *C.microglochin*, *C.subspathacea*, *C.williamsii*, Eriophorumscheuchzerisubsp.arcticum (Cyperaceae), *Andromedapolifolia*, Orthiliasecundasubsp.obtusata (Ericaceae), *Oxytropispodocarpa* (Fabaceae), *Luzulagroenlandica* (Juncaceae), *Triglochinpalustris* (Juncaginaceae), *Utriculariaochroleuca* (Lentibulariaceae), *Huperziacontinentalis* (Lycopodiaceae), *Montiafontana* (Montiaceae), *Corallorhizatrifida*, Platantheraobtusatasubsp.obtusata (Orchidaceae), *Hippurislanceolata*, *H.vulgaris*, *Plantagomaritima* (Plantaginaceae), Calamagrostisneglectasubsp.groenlandica, *C.purpurascens*, Festucaproliferavar.lasiolepis, F.rubrasubsp.rubra, F.rubrasubsp.arctica, Hordeumjubatumsubsp.jubatum, Leymusmollissubsp.mollis, L.mollissubsp.villosissimus, *Puccinelliavaginata* (Poaceae), *Primulaegaliksensis* (Primulaceae), *Cryptogrammastelleri* (Pteridaceae), Coptidium×spitsbergense (Ranunculaceae), *Potentillacrantzii*, P.hyparcticasubsp.hyparctica, *Rubuschamaemorus*, *Sibbaldiaprocumbens* (Rosaceae), *Salixfuscescens* (Salicaceae), *Micranthesfoliolosa*, *M.nivalis*, *M.tenuis* (Saxifragaceae) and *Woodsiaalpina* (Woodsiaceae). We recorded 196 taxa in Katannilik Territorial Park (191 species, three infraspecific taxa and two nothospecies); 145 of these taxa are first records for the park. We recorded 170 taxa in Kimmirut and vicinity (166 species, three infraspecific taxa and one nothospecies) in Kimmirut and vicinity; 15 of these taxa are first records for Kimmirut and vicinity. All study area species are native, except two grasses that grew in Kimmirut: F.rubrasubsp.rubra, which may have been seeded and Hordeumjubatumsubsp.jubatum, of unknown origin. We summarize the distribution on Baffin Island for each taxon recorded in the study area, including several unpublished southern Baffin Island records.

## ﻿Introduction

The Arctic is warming more than twice as fast as the rest of the planet due to climate change. Due to this rapid warming, the Arctic environment is undergoing many changes. October 2020 to September 2021 was the Arctic’s seventh-warmest 12-month period on record. During this period, the surface air temperature anomaly for land north of 60° was 1.1 °C above the 1980–2010 mean (Ballinger et al. 2021). North American Arctic June snow cover extent has been below the long-term average yearly since 2006 (Mudryk et al. 2021). In the last 15 years, the 15 lowest September sea ice minimum extents have occurred, and multiyear sea ice reached its second-lowest level in September 2021 (Meier et al. 2021). Between 2007 and 2016, soil temperature in the continuous permafrost zone increased by 0.39 ± 0.15 °C (Biskaborn et al. 2019).

Warming air temperature, sea ice decline, changing snow cover and changing permafrost influence Arctic vegetation. Satellite observations show that yearly maximum tundra vegetation greenness, a tundra productivity measure, increased across most of the Arctic between 1982 and 2020 (Frost et al. 2021). Browning, or decreased tundra productivity, has occurred in some Arctic regions, including parts of the Canadian Arctic Archipelago and southwest Alaska (Frost et al. 2021). A major tundra greening driver is increasing shrub height and abundance, which researchers have documented at many Arctic locations (Sturm et al. 2001; Tape et al. 2006; Myers-Smith et al. 2011a, b; Moffat et al. 2016; Vowles and Björk 2019). Shrub growth response to climate varies with factors such as geography, soil moisture and sea ice decline (Myers-Smith et al. 2015; Gamm et al. 2018; Buchwal et al. 2020). Shrub expansion is often associated with decreased species diversity in tundra ecosystems and changes in plant community functional group composition (Cornelissen et al. 2001; Wilson and Nilsson 2009; Pajunen et al. 2011; Crofts et al. 2018). Heterogeneous High Arctic tundra landscapes may become homogeneous dwarf-shrub tundra due to climate change (Stewart et al. 2018). Increased productivity of non-shrub plant functional groups also contributes to greening (Beck and Goetz 2011). Models predict that decreasing snow cover will substantially modify Arctic plant community biodiversity and functional trait composition (e.g., plant size, structure and biogeochemistry) (Niittynen et al. 2020). At the Arctic’s southern edge, the forest-tundra ecotone is advancing north in some regions (Rees et al. 2020) and could advance north considerably by 2080 (Zhang et al. 2013). Researchers have observed phenology shifts in Arctic plant species in response to climate change (Panchen and Gorelick 2017; Prevéy et al. 2017, 2019; Collins et al. 2021).

Despite the rapid Arctic vegetation changes occurring and predicted over the coming decades, knowledge gaps remain in our understanding of the Arctic vascular flora’s diversity and distribution. Researchers have studied and documented the Canadian Arctic vascular flora for over 150 years (Porsild and Cody 1980; Aiken et al. 2007; Payette 2013, 2015, 2018). Yet, we do not know the species composition of many Arctic areas. Specimen-based sampling across the Canadian Arctic is sparse, given its size (ca. 40% of Canada). Furthermore, Arctic plant collections are biased in time, space, taxonomic composition and life history (Panchen et al. 2019). Vascular plant floristic studies in the Canadian Arctic demonstrate we have much to learn about the flora’s current composition and distribution. For example, recent studies have expanded species’ known ranges in the Arctic and documented new species records for local study areas, islands, broader regions and territories (Saarela et al. 2012, 2013, 2017, 2020a, b; Gillespie et al. 2015; Sokoloff 2015; Desjardins et al. 2021). Floristic studies like these generate biodiversity knowledge that provides information for biogeographical, ecological, taxonomic, evolutionary and related research. They also provide information relevant to conservation and protected areas establishment and management and contribute to knowledge and appreciation of our natural heritage. Critically, they serve as a baseline for monitoring and assessing biodiversity change over time in response to climate change and other factors, such as industrial activity.

**Figure 1.**
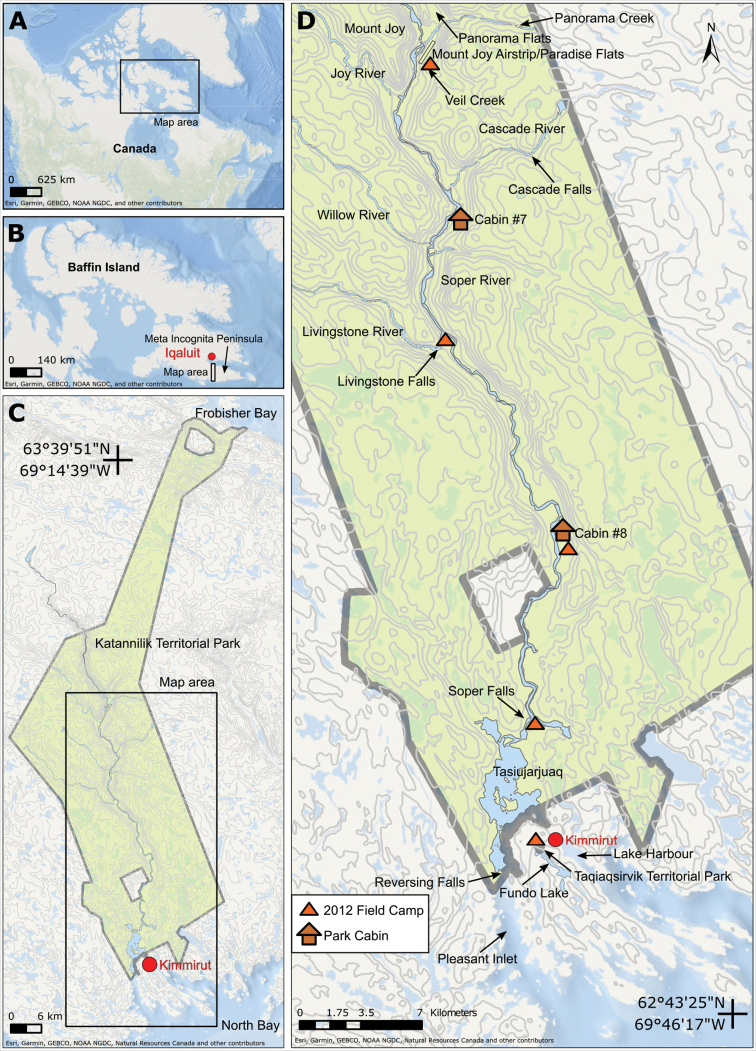
Maps of the study area **A** Canada **B** Baffin Island **C** Katannilik Territorial Park on the Meta Incognita Peninsula, southern Baffin Island **D** Area studied during our 2012 field expedition along the lower Soper River valley. Camps, referenced cabins and geographic features are indicated in our collection data.

Baffin Island, Nunavut, is Canada’s largest island and Earth’s fifth-largest island (Fig. 1A). Over the last century, Baffin Island’s plant diversity has been documented fairly extensively with collections. Many of these collections have been mentioned, mapped or both in Canadian Arctic floras (Polunin 1940; Porsild 1957, 1964; Porsild and Cody 1980; Aiken et al. 2007). Comprehensive collection-based floristic inventories, however, have been published for only a few local Baffin Island areas: Iqaluit (formerly Frobisher Bay) (Calder 1951), Ogac Lake on Frobisher Bay’s south side (McLaren 1964), the Penny Highlands on Cumberland Peninsula (Schwarzenbach 2010) and Dorset and Mallik islands along the southern Foxe Peninsula (Saarela et al. 2020a). Here, we report the results of a study of the vascular plants of Katannilik Territorial Park, Kimmirut and vicinity, based on historical and contemporary collections.

## ﻿Study area

### ﻿Geography

Inuit Nunangat, the Inuit homeland, stretches across Canadian Arctic lands and waters and spans the four Inuit regions: the Inuvialuit Settlement Region, Nunavut, Nunavik and Nunatsiavut. Inuit Nunangat is a distinct geographic, cultural and political region within Canada (Government of Canada 2022). Formerly part of the Northwest Territories, Nunavut became a Canadian territory on 1 April 1999. It comprises more than one-fifth of Canada and includes more than two-thirds of the country’s shoreline. Nunavut is divided into three regions: Kitikmeot, Kivalliq and Qikiqtaaluk. Of these, the Qikiqtaaluk Region is the easternmost, largest and most populated. Baffin Island is part of the Qikiqtaaluk Region. The Inuktitut word *qikiqtaaluk* means “very big island.” Hudson Strait separates Baffin Island from the Canadian mainland and Baffin Bay and Davis Strait separate it from Greenland. Southern Baffin Island includes Meta Incognita Peninsula, a highland area reaching an elevation of ca. 914 m (Fig. 1B). Hudson Strait bounds Meta Incognita Peninsula to the south, Frobisher Bay bounds it to the north and the peninsula extends inland to Amadjuak Lake, Baffin Island’s second-largest lake.

The Soper River on Meta Incognita Peninsula (Fig. 1C, D) is southern Baffin Island’s largest river. Its source is a lake at ca. 63°30'39"N, 69°37'28"W, from which the river flows 108 km into Hudson Strait. Numerous small streams, basins and rivers feed the Soper River, draining some 2,500 km^2^. Its major tributaries are the Joy, Willow, Livingstone and Cascade rivers. The Soper River valley ranges from ca. 0.5 km to 1.2 km wide (Soper 1936). In Inuktitut, the Soper River is called *Kuujuaq*, which means “big river” (Laird and Associates 2004). The river is navigable for ca. 50 km (Laird and Associates 2004). Naturalist Joseph Dewey Soper explored and mapped the river in June and July 1931; he called it the Koukdjuak River. The Geographic Board of Canada named the river and the lake into which it runs after J.D. Soper in 1946 to avoid confusion with Baffin Island’s Koukdjuak River that flows out of Nettilling Lake. The Canadian Heritage Rivers System declared the Soper River a Canadian Heritage River in 1992. The Soper River’s roles within the Canadian Heritage Rivers System include “to highlight an outstanding river environment which exemplifies the natural ecosystem and geological history of the southern Baffin region” and “to encourage protection, future scientific research and public understanding of the full range of natural and cultural heritage values of this northern region with a focus on the Soper River” (Laird and Associates 2004: 9).

The Soper River is a central feature of Katannilik Territorial Park (1,262 km^2^; Fig. 1C, D), established in 1993. At that time, the park fell within the Northwest Territories. Now it is part of Nunavut, administered by the Nunavut Department of Environment’s Parks and Special Places division. Nunavut’s territorial parks aim to protect cultural and natural landscapes, enhance community and visitor experience and engage the community in heritage appreciation and conservation (Government of Nunavut 2022). The Inuktitut word *katannilik* means “where there are waterfalls,” referring to the many falls and rapids that occur along or feed the Soper River. The park offers numerous recreation activities, including canoeing, kayaking, rafting, hiking and camping. The 120 km Itijjagiaq Trail, a traditional travel route between the communities of Iqaluit and Kimmirut, runs through the park (Laird and Associates 2004). The trail traverses the park from Bay of Two Rivers (Nunngarut Bay) on Frobisher Bay to Kimmirut, south of the park. Itijjagiaq Trail is part of the Trans Canada Trail, the world’s longest trail network (Trans Canada Trail 2021). Amenities along the trail within the Soper River valley include eight emergency shelters, a large group cabin near Mount Moore and a campground near Soper Falls (Laird and Associates 2004).

The Soper River enters Katannilik Territorial Park ca. 13 km north-northwest of Mount Joy (elevation 562 m). Mount Joy, just north of the Joy and Soper rivers’ confluence, is one of the park’s prominent geographic features. It portrays a human face on its southern slope; Soper (1933) illustrated this. The Soper Heritage River Guide Map refers to several geographic features near Mount Joy by unofficial names (Anonymous no date). “Paradise Flats,” a large terrace immediately east of Mount Joy, includes a gravel landing strip where small aircraft drop paddlers embarking on a river trip. “Panorama Flats” and “Panorama Creek” are northeast of “Paradise Flats”. “Veil Creek” feeds the Soper River southeast of “Paradise Flats”. “Cascade Rapids” occur along the river, ca. 10 km south of Mount Joy. Approximately 0.5 km further south, the Soper and Cascade rivers meet on the Soper River’s east side. Soper (1936:434) described the Cascade River as “wild and tumultuous”. Two falls (“Cascade Falls”) occur along the Cascade River: a smaller one ca. 1 km upriver that drops ca. 4.5 m and a larger one another 3 km upriver that drops ca. 29 m (Soper 1936). Some 14.5 km south of Mount Joy, the Willow River meets the Soper River on its west side. Some 3.5 km further south, the Livingstone and Soper rivers meet on the Soper River’s west side. Along the Livingstone River just above the confluence are “Livingstone Falls,” which drop 3 m over a 30 m ledge. About 50 km south of Mount Joy, the Soper River falls ca. 6 m along a 0.5 km series of rapids, Soper Falls. Below Soper Falls is Tasiujarjuaq (formerly Soper Lake), a lake fed by freshwater and saltwater. The Inuktitut word *tasiujarjuaq* means “big lake-like lake”. At high tide, saltwater flows into Tasiujarjuaq from Pleasant Inlet, which is immediately south of the lake and extends ca. 10 km from north-western North Bay. North Bay is a large bay on Hudson Strait’s north side that extends from Imiligaarjuit (formerly Cape Tanfield), ca. 26 km southeast of Kimmirut, to ca. 26 km west-northwest. At low tide, freshwater flows from Tasiujarjuaq into Pleasant Inlet. Just east of Pleasant Inlet is Glasgow (Westbourne) Bay, a large inlet that extends northwest ca. 18 km from North Bay.

Humans have occupied the North Bay area for thousands of years, dating to the pre-Dorset period (4500–2700 B.P.) (Maxwell 1973; Milne and Park 2016). Today, the Hamlet of Kimmirut is at the head of Glasgow Bay. The Inuktitut word *kimmirut* means "heel", referring to a marble outcrop near the community resembling the heel of a foot. Until 1 January 1996, Kimmirut was named Lake Harbour. An Anglican Church was erected in the community in 1909 and the Hudson’s Bay Company set up a trading post there in 1911. Qikiqtani Inuit Association (2013) recorded the history of the Kimmirummiut, the area’s people. Kimmirut’s area is 2.27 km^2^ and its elevation is 53 m. Its population in 2016 was 398 (Statistics Canada 2017). Kinngait (formerly Cape Dorset) is the only other settlement on Baffin Island’s south coast, 364 km northwest.

### ﻿Geology

The Soper River valley’s substrate is mainly till, outwash, deltaic gravel and sandy alluvium. Calcareous outcrops, including marble and crystalline limestone, occur just north of the Soper and Livingstone rivers’ confluence (Soper 1936; St-Onge et al. 1996). Much of the plateau above the Soper River valley comprises granitic layered tonalites and gneiss (Soper 1936; St-Onge et al. 1996). An enclave of Inuit Owned Land surrounded by the park contains surface deposits of lapis lazuli, mica and garnet (Hogarth 1971; Grice and Gault 1983). The park’s bedrock mostly comprises Cumberland batholith monzogranite, with small areas of Lake Harbour Group metasedimentary rocks (marble and monzogranite) and Ramsay River orthogneiss (Hodgson 2005).

### ﻿Vegetation

**Figure 2.**
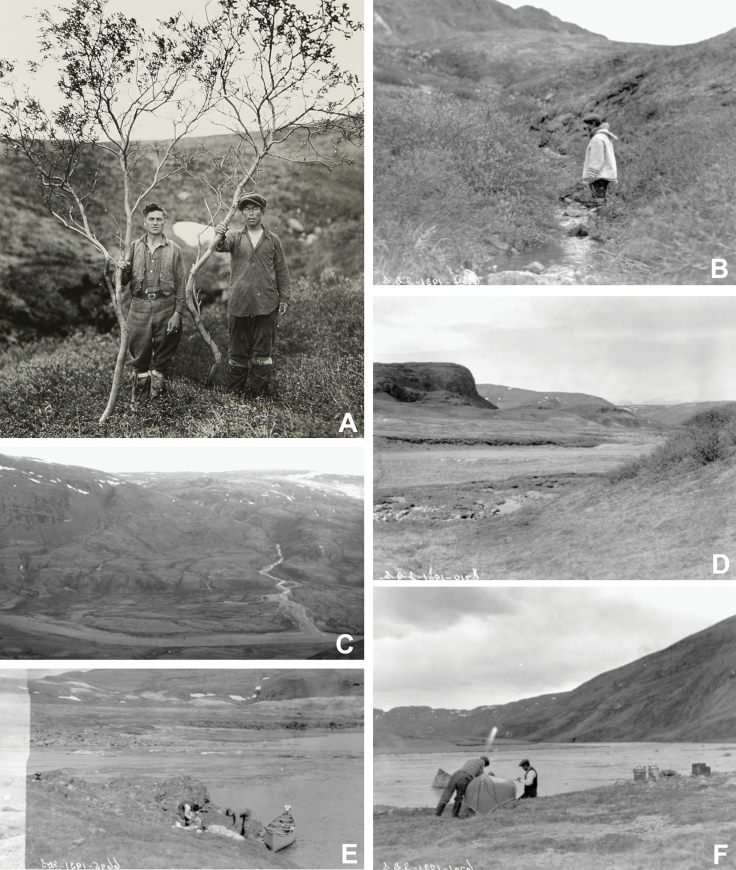
Images from Joseph Dewey Soper’s 1931 explorations along the Koukdjuak [Soper] River **A** J.D. Soper and one of his Inuit companions, Matuse or Matoosa, with willows 12'6" [3.8 m] high that grew at the mouth of the Willow River, a tributary to the Koukdjuak [Soper] River, 1 July 1931 [Library and Archives Canada MIKAN No. 5277159] **B** Matuse or Matoosa standing amongst willows 4'–5' [1.2–1.5 m] high along a tributary brook of the Koukdjuak [Soper] River, 1 July 1931 [Library and Archives Canada MIKAN No. 5277158] **C** looking down on the valley of the Koukdjuak [Soper] River (eastwardly) from a height of 1200' [366 m], (9:30 in the evening), 1 July 1931 [Library and Archives Canada MIKAN No. 5277160] **D** a view across the valley of the Koukdjuak [Soper] River to the northeast, 2 July 1931 [Library and Archives Canada MIKAN No. 5277161] **E** noon halt at rapids on the Koukdjuak [Soper] River, 30 June 1931 [Library and Archives Canada MIKAN No. 5277156] **F** patching canoe after a fight in the rapids of the Koukdjuak [Soper] River, 30 June 1931 [Library and Archives Canada MIKAN No. 5277157]. Credit: Joseph Dewey Soper/Department of Indian Affairs and Northern Development fonds/Library and Archives Canada/Public domain.

**Figure 3.**
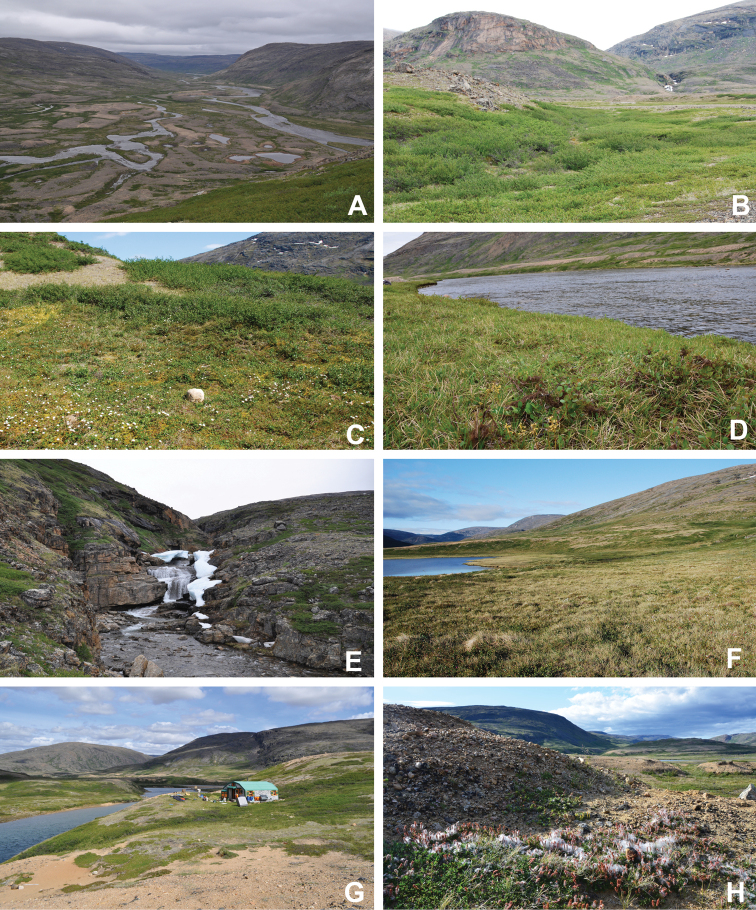
Habitat diversity within Katannilik Territorial Park **A** “Panorama Flats” and “Panorama Creek” (left) and the Soper River running past Mount Joy (right), 2 July 2012 **B** tundra depressions dominated by *Betulaglandulosa* on “Panorama Flats”, 5 July 2012 **C***Betulaglandulosa*–Rhododendrontomentosumsubsp.decumbens-dominated dry tundra on “Panorama Flats”, 30 June 2012 **D** moist tundra along the edge of Soper River, with *Corallorhizatrifida*, 1 July 2012. **E** “Panorama Falls”, 2 July 2012 **F** mesic tundra along Soper River, south of Mount Joy, 5 July 2012 **G** group/warden cabin # 7 beside Soper River, 6 July 2012 **H** dry, gravelly hills with *Salixuva-ursi*, 6 July 2012. Photos **A, E, G** by L.J. Gillespie, **B, C, F** by J.M. Saarela and **D, H** by R.D. Bull.

**Figure 4.**
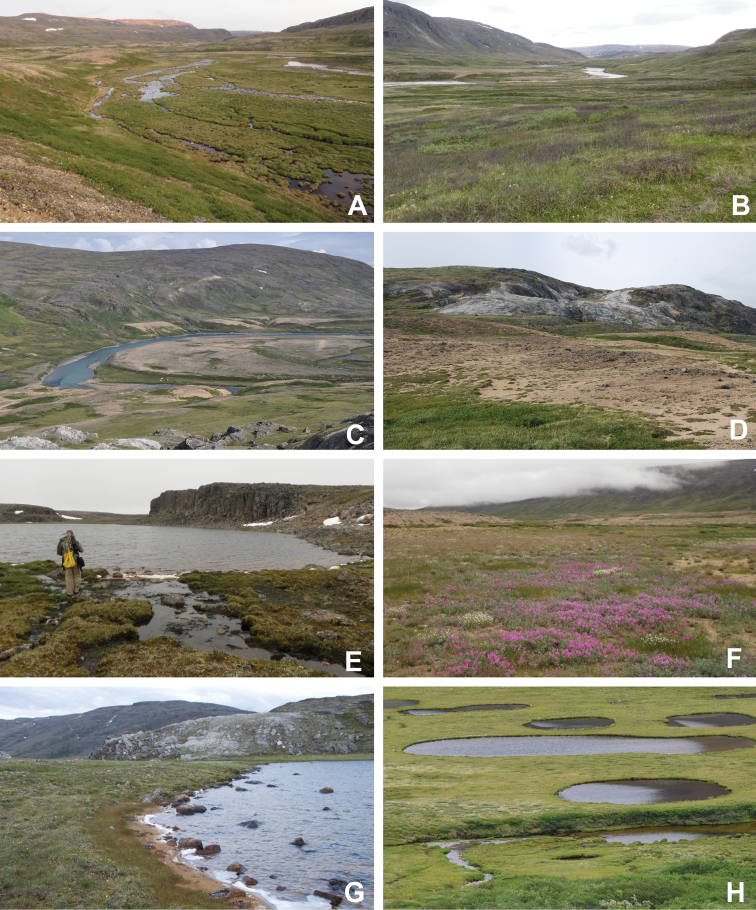
Habitat diversity within Katannilik Territorial Park. **A** wet sedge meadow near Cascade River, 6 July 2012 **B** dry tundra with *Betulaglandulosa* die-off near Willow River, 8 July 2012 **C** dry flats at the confluence of the Soper and Livingstone rivers, 12 July 2012 **D** crystalline limestone outcrops over gravelly hills at the confluence of Soper and Livingstone rivers, 12 July 2012 **E** R.D. Bull near a lake atop the plateau west of the confluence of Soper and Livingstone rivers, 11 July 2012 **F** sandy depressions on flats at the confluence of Soper and Livingstone rivers, dominated by *Chamaenerionlatifolium*, *Saxifragacernua* and *Artemisiaborealis*, 11 July 2012 **G** wet pond margin near the confluence of Soper and Livingstone rivers, with a large population of *Carexmicroglochin*, 12 July 2012 **H** shallow tundra ponds along Soper River near *Salixplanifolia* stand, 13 July 2012. Photos **A–C, E, F** by L.J. Gillespie, **D, G** by J.M. Saarela and **H** by R.D. Bull.

**Figure 5.**
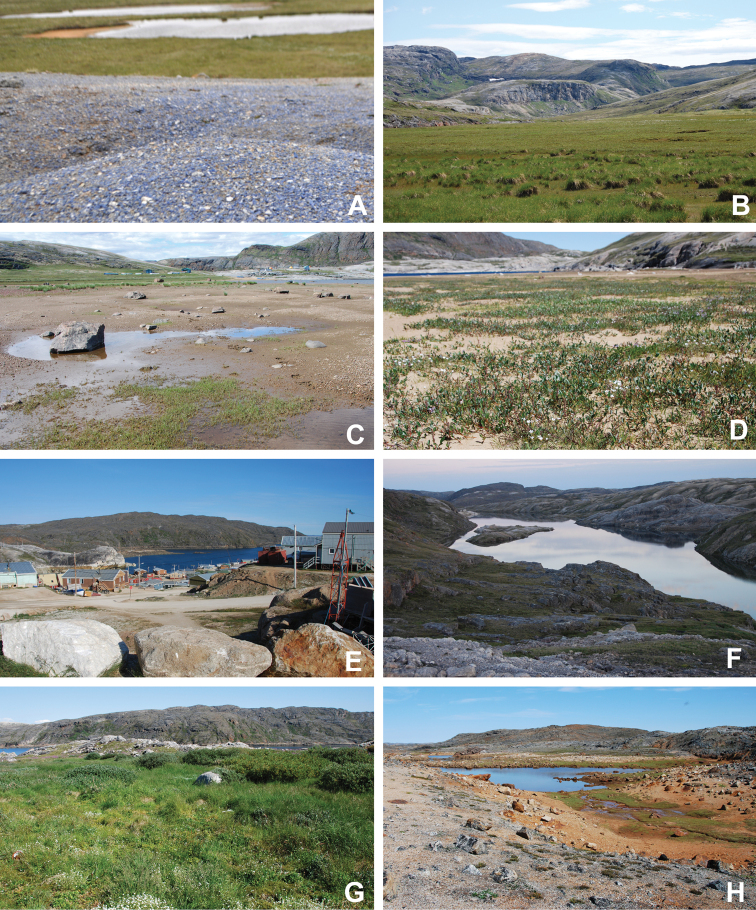
Habitat diversity within the study area **A** lapis lazuli deposit on the west bank of the Soper River, 16 July 2012 **B** hummocky sedge meadow on the east bank of Soper River, near Soper Falls, 17 July 2012 **C** wet, sandy flats near the campground at Soper Falls, with *Eleocharisacicularis*, 17 July 2012 **D** dry, sandy flats south of Soper Falls, with *Salixarctica*, *Agrostismertensii* and *Cerastium*, 18 July 2012 **E** Kimmirut, 20 July 2012 **F** Fundo Lake, Kimmirut, 22 July 2012 **G** enriched vegetation near Kimmirut dump and sewage outlet, 22 July 2012 **H** coastal shoreline and dry ridges near Pleasant Inlet, 21 July 2012. Photos **A** by P.C. Sokoloff and **B–H** by J.M. Saarela.

Soper (1933, 1936) briefly described the area’s vegetation, which becomes more luxuriant as the Soper River valley ascends. Near the coast, willows range from prostrate to 30–60 cm tall, while further up the valley, they reach more than 3.5 m tall. Soper (1933:133) published a photograph of these tall willows. Nicholas Polunin (1948) described Lake Harbour’s vegetation. He included species lists of vascular plants, bryophytes and lichens typical of common habitats. He focused on hill summits and slopes, lowlands, marshes, snow patch habitats, “flower slopes” (lushly vegetated south-facing slopes), freshwater habitats and seashore habitats. Figs 2–5 show the study area’s habitat diversity.

The Arctic bioclimate zone has an Arctic climate, Arctic flora and tundra vegetation. The Circumpolar Arctic Vegetation Map divided this circumpolar zone into five bioclimate subzones named A to E, defined by summer temperature and vegetation characteristics (Elvebakk 1999; CAVM Team 2003; Walker et al. 2005). Subzone A is restricted in Canada to the north-western Queen Elizabeth Islands. It is the coldest and harshest subzone, characterized by a 0–3 °C mean July temperature, < 5% vascular plant cover, vascular plant growth low to the ground (barely exceeding the height of mosses, woody plants absent) and local vascular floras with fewer than 50 species. Subzone E is restricted in Canada to the mainland. It is the warmest subzone, characterized by a 9–12 °C mean July temperature, 80–100% vascular plant cover, a 20–50(–80 cm) tall herbaceous/dwarf-shrub layer and local vascular floras with 200 to 500 species.

Southern Baffin Island spans two bioclimate subzones. The ca. western third of Foxe Peninsula and adjacent islands fall within Subzone C. This subzone has a 5–7 °C mean July temperature, 5–50% vascular plant cover, a moss layer 3–5 cm thick, a herbaceous layer 5–10 cm tall, dwarf shrubs less than 15 cm tall and local vascular floras with 75 to 150 species. The rest of southern Baffin Island, including the study area, falls within Subzone D, which extends north to ca. 67°47'N. This subzone has a 7–9 °C mean July temperature, 50–80% vascular plant cover, a moss layer 5–10 cm thick, herbaceous and dwarf shrub layers 10–40 cm tall and local vascular floras with 125 to 250 species. Elsewhere in Canada, Subzone D includes southern Banks, Southampton and Victoria islands and much of the mainland Arctic. Following earlier North American Arctic vegetation classifications, the study area's vegetation is Low Arctic (Polunin 1951; Bliss 1997), low erect shrub tundra (Edlund and Alt 1989; Edlund 1990), southern Arctic dwarf shrub tundra (Daniëls et al. 2000) or erect dwarf shrub tundra (Walker et al. 2002). The Soper River valley’s dominant circumpolar vegetation is erect dwarf shrub tundra, i.e., shrubs mostly less than 40 cm tall (CAVM Team 2003). Elsewhere on Baffin Island, this vegetation occurs in three areas of Meta Incognita Peninsula (two east of the Soper River and one along Frobisher Bay), near the tip of Hall Peninsula and near Pangnirtung (CAVM Team 2003).

Researchers divide the circumpolar Arctic into 21 floristic provinces, delimited primarily based on vascular plant species distributions (Elven et al. 2011). The study area falls within the Hudson Bay–Labrador Region comprising Hudson Bay’s shores and islands (including Southampton Island and its small surrounding islands), Ungava Peninsula, northernmost Labrador Peninsula and southern Baffin Island, north to Cumberland Sound. The region’s flora has a boreal American influence.

### ﻿Climate

The study area’s climate is Tundra (class ET) according to the Köppen-Geiger climate classification, characterized by an average temperature of the warmest month between 0 °C and 10 °C (Beck et al. 2018). Models predict that, by 2071–2100, the study area’s climate will be Subarctic (class Dfc), characterized, in part, by one to three months averaging above 10 °C (Beck et al. 2018). Meteorological station Kimmirut A records weather data for Kimmirut, but long-term data for the 1981–2010 climate normals period (i.e., three-decade averages of climate variables) are lacking. The nearest stations on Baffin Island with data for the 1981–2010 climate normals period are Cape Dorset A (Environment Canada 2021a) to the northwest and Iqaluit A (Environment Canada 2021b) to the north-northeast. During the 30-year period, Kinngait (formerly Cape Dorset) had a -8.9 °C ± 4.3 mean annual air temperature, a 7.8 °C ± 1.2 mean July temperature and a -25.4 °C ± 2.8 mean February temperature. Iqaluit had a -9.3 °C ± 3.7 mean annual air temperature, an 8.2 °C ± 1.0 mean July temperature and a -27.5 °C ± 4.4 mean February temperature. Kinngait’s annual precipitation was 418.5 mm; ca. 38% fell as rain and the rest as snow. Iqaluit’s was 403.7 mm; ca. 49% fell as rain. According to Nunavut Parks & Special Places (2008), the sheltered Soper River valley is four to five degrees Celsius warmer than the surrounding areas, including Kimmirut. We are unaware of long-term climate data for the valley.

### ﻿Collecting history

Collectors have gathered botanical specimens in the study area throughout the last century, beginning in the late 1920s. Botanist Malte Oscar Malte, then head of the National Herbarium of Canada, National Museum of Canada (now the Canadian Museum of Nature), travelled to Lake Harbour four times aboard Hudson’s Bay Company’s *RMS Nascopie* as part of the 1927, 1928 and 1933 Eastern Arctic Patrols. He collected at Lake Harbour on 1–2 and 25–26 August 1927, 29–30 August 1928 and 22 July 1933 (Malte 1934; Polunin 1940). Malte died soon after his last visit to Lake Harbour, having become ill on Charlton Island in James Bay during the 1933 patrol (Smith and Lackenbauer 2014). Danish biologist Frits Johansen collected 33 vascular plant species near Lake Harbour on 23 August 1927, during the Canadian Hudson Strait Expedition, “… in the outer part of the sailing into the post, … between a larger island … and the ‘Meta incognita’ part of Baffin Island…” (Johansen 1934). Joseph Dewey Soper collected plants while living in Lake Harbour in the early 1930s (Dalton 2010). In 1931, he collected at Lake Harbour on 21 June, 26 June, 5 July and 7 July and near the rapids [Soper Falls] of the Koukdjuak [Soper] River on 25 June. On 1 July 1931, he canoed up the Koukdjuak [Soper] River with two Inuit companions, Moosa and Mutuse, and collected inland (Fig. 2). Botanist Nicholas Polunin was part of the 1934 Eastern Arctic Patrol and collected in Lake Harbour from 30–31 August (Polunin 1940). Polunin returned to Lake Harbour two years later with the 1936 Eastern Arctic Patrol and again collected there from 26–28 July (Cockburn 1983). Alice M. Tallman, recorded on her labels as Mrs. George K. Tallman, also traveled with the 1936 Eastern Arctic Patrol. She collected a few specimens at Lake Harbour on 27 July (MIN) (Hudson’s Bay Company Archives, Gertrude Perrin fonds, HB2008/004) (Cockburn 1983). Père Arthème Dutilly joined the 1936 Eastern Arctic Patrol in Churchill and collected in Lake Harbour from 26–28 August (Smith and Lackenbauer 2014). Dutilly collected again in Lake Harbour on 28 August 1941. Norman Bethune Sanson, Banff Park Museum curator from 1896 to 1931 (Bird 1976), collected at least two specimens in Kimmirut on 17 August 1938. Though we are unaware of published accounts of Sanson’s Arctic fieldwork, label data from HUH, NYBG and TRT and specimen citations in Ball (1950) indicate he also collected that year at Fort Ross (Somerset Island), Pond Inlet and Clyde River (Baffin Island), Chesterfield Inlet, and Port Burwell (Killiniq Island). Margaret Oldenburg (Grand Marais, Minnesota), a former librarian who undertook numerous expeditions to the Canadian Arctic to collect plants from the late 1930s to the mid-1950s, visited Kimmirut with the 1939 Eastern Arctic Patrol and collected there on 22–23 July (University of Minnesota Archives, Margaret Oldenburg fonds). Given they were only in the area for one to two days during brief stops, we assume Malte, Polunin, Dutilly and Oldenburg collected in the immediate Lake Harbour settlement area. Canadian Arctic floristic treatments have considered most of the 1920s and 1930s collections from the study area (Polunin 1940; Porsild 1957, 1964; Porsild and Cody 1980; Aiken et al. 2007).

We know of few collections gathered in the study area between 1940 and the early 2000s. Weston Blake Jr. collected one specimen at a site just east of Kimmirut on 27 June 1965, while conducting geologic research on southern Baffin Island as part of “Operation Amadjuak” (Blake 1966). Peggy Fleming, US National Parks Service (Fleming and Raclare 1995), collected two specimens near the Livingstone and Soper rivers’ confluence on 26 July 1992, while on vacation canoeing the Soper River (P. Fleming, personal communication).

Botanists again visited the study area in the first decade of the 21^st^ century. Susan Aiken (Canadian Museum of Nature) and her associate Rosalind Iles collected in the Soper River valley in 2002. Many of their collections are vouchers for photographs published in the Flora of the Canadian Arctic Archipelago (Aiken et al. 2007). Annie Archambault collected in Kimmirut from 4–5 August 2005, while conducting fieldwork for her Ph.D. research on *Oxytropis* DC. (Fabaceae) (Archambault 2013). We collected in the area in 2012.

## ﻿Materials and methods

### ﻿Field research

We studied the flora of Katannilik Territorial Park and vicinity from 30 June to 23 July 2012. We conducted research under a Nunavut Territorial Parks Use Permit, Nunavut Wildlife Research Permit WL 2012-034, Nunavut Water Board Permit 3 BC-FAA1212, Qikiqtani Inuit Association Certificate of Exemption (Access to Inuit Owned Land) Q12X016 and Polar Continental Shelf Program Project Number 515-12. We explored botanical diversity along the Soper River while travelling by inflatable canoe from Mount Joy to Soper Falls. We spent 20 days within the park and four days outside the park, in Kimmirut and vicinity.

**Figure 6.**
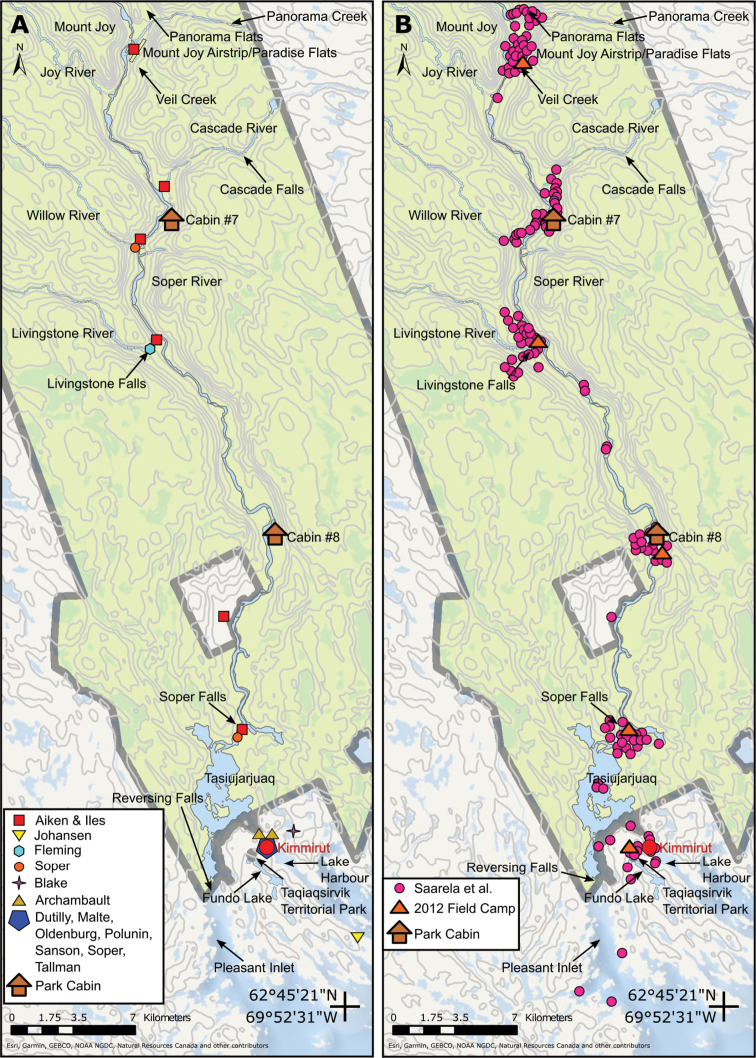
Vascular plant collection sites along the lower Soper River **A** sites where collections were made before 2012 **B** sites where we collected plants in 2012.

We flew from Iqaluit to “Paradise Flats” near Mount Joy, our starting point, by Twin Otter plane, which landed on the airstrip there. We established six field camps along the Soper River: at the airstrip (30 June to 5 July; 63°14'52"N, 69°36'28"W), at group/warden cabin #7 (6–9 July; 63°09'44"N, 69°39'28"W), at the Livingstone River and Soper River confluence (10–12 July; 63°06'33"N, 69°43'46"W), on the east riverbank south of emergency shelter #8 (13–15 July; 62°59'02"N, 69°43'01"W) and at the Soper Falls campground (17–19 July; 62°54'33"N, 69°50'12"W). We established a final camp at Taqaiqsirvik Territorial Park (campground) in Kimmirut (20–23 July; 62°50'56"N, 69°53'18"W). We explored each camp area on foot, seeking out as many habitats as possible and aiming to record all vascular plant species present in each area with at least one collection. While travelling between camps, we stopped to collect at two willow thickets along the river (13 July) and the lapis lazuli occurrence (16 July). Labels on our specimens from the lapis lazuli site (*Saarela et al. 2496–2506*) mistakenly indicate it is within Katannilik Territorial Park; it is not. On 17–18 July, we collected near Soper Falls. On 19 July, we collected on a small unnamed island within Tasiujarjuaq and outside the park, around the Kimmirut boat landing and Reversing Falls. On 20, 22 and 24 July, we collected in Kimmirut and vicinity. On 21 July, we collected along Pleasant Inlet, west of Kimmirut, travelling via a boat charter from the hamlet. Fig. 6 shows the locations of our collecting sites and Table 1 describes them. We assigned a code to each site with unique geographical coordinates and grouped them, based on the general collection area, which usually corresponds to our camp locations. We reference these codes in the annotated checklist.

**Table 1.** Detailed collecting localities and codes identifying each locality. Sites prefaced by an asterisk are those of collectors before 2012. We determined coordinates within square brackets by georeferencing. Suppl. material 1 includes complete collection data.

Site Code	Locality
**Katannilik Territorial Park, vicinity of Mount Joy**	
MJ-1	*Mount Joy, [63°16'N, 69°40'W ± 2000 m]
MJ-2	Two km northeast of Mount Joy, above creek running into Soper River overlooking Panorama Flats, 63°15'29"N, 69°35'4"W ± 100 m, elev. 155 m
MJ-3	Along wet creek bank running from “Panorama Falls” to Soper River, just beyond the end of Mount Joy near the river, 63°14'47"N, 69°36'48"W ± 2 m, elev. 75 m
MJ-4	Below Mount Joy, east of Soper River, adjacent to “Paradise Flats”, small drainage and sedge meadow running into river flats, 63°14'52"N, 69°36'28"W ± 25 m, elev. 78 m
MJ-5	Below Mount Joy, east of Soper River; meadow around small pond adjacent to Mount Joy, rocky river flats beside Soper River and below a small hill, 63°14'52"N, 69°36'32"W ± 50 m, elev. 50 m
MJ-6	Bottom of north-facing slope overlooking “Panorama Flats”, ca. 2 km north of Mount Joy, 63°15'55"N, 69°35'24"W ± 5 m, elev. 20 m
MJ-7	Densely vegetated river flat near Mount Joy, ca. 5 m wide band between river and dry, stony floodplain, 63°14'52.7"N, 69°36'45.7"W ± 10 m, elev. 75 m
MJ-8	Densely vegetated river flat near Mount Joy, ca. 5 m wide band between river and dry, stony floodplain, 63°14'53"N, 69°36'45"W ± 10 m, elev. 75 m
MJ-9	Dry rocky, sandy slopes at the end of Mount Joy near Soper River, 63°14'48"N, 69°36'43"W ± 5 m, elev. 80 m
MJ-10	Dry, rocky upper slopes of riverbank running from “Panorama Falls” to Soper River, adjacent to Mount Joy, 63°14'48"N, 69°36'32"W ± 10 m, elev. 81 m
MJ-11	Dry, stony flats along Soper River, 63°14'54"N, 69°36'42"W ± 25 m, elev. 80 m
MJ-12	Flats near the confluence of “Veil Creek” and “Panorama Creek”, 63°15'1"N, 69°35'53"W ± 25 m, elev. 90 m
MJ-13	Heath tundra adjacent to rocky river flats by Soper River, 63°14'47"N, 69°36'52"W ± 45 m, elev. 81 m
MJ-14	Just below flood line of birch-dominated riverbank along Soper River, 1.5 km north of Mount Joy, 63°15'39"N, 69°35'33"W ±15 m, elev. 94 m
MJ-15	Just south of “Veil Creek” near Mount Joy, near large boulder, 63°14'50"N, 69°36'13"W ± 1 m
MJ-16	Large hill between Soper River and “Panorama Creek”, above “Panorama Flats”, 2.5 km northeast of Mount Joy, steep boulder slope above Soper River, 63°15'47"N, 69°35'10"W ± 25 m, elev. 250 m
MJ-17	Large hill between Soper River and “Panorama Creek”, above “Panorama Flats”, 2.5 km northeast of Mount Joy, 63°15'43"N, 69°35'10"W ± 3 m, elev. 280 m
MJ-18	Lush, peaty meadow below “Panorama Falls”, 63°15'25"N, 69°35'1"W ± 5 m, elev. 110 m
MJ-19	Meadow below north-facing slope overlooking “Panorama Flats”, 63°15'52"N, 69°35'31"W ± 2 m, elev. 109 m
MJ-20	Mesic tundra hummocks adjacent to “Panorama Creek”, ca. 200 m above the confluence with “Veil Creek”, 63°15'5"N, 69°35'45"W ± 50 m, elev. 87 m
MJ-21	Near the summit of large hill overlooking “Panorama Flats” and Soper River, ca. 1.8 km north of Mount Joy, 63°15'42"N, 69°35'11"W ± 2 m, elev. 279 m
MJ-22	“Panorama Creek” at “Panorama Falls”, 2 km northeast of Mount Joy, 63°15'32"N, 69°34'53"W ± 2 m, elev. 200 m
MJ-23	“Panorama Creek”, at “Panorama Falls”, 2 km northeast of Mount Joy, 63°15'34"N, 69°34'42"W ± 10 m, elev. 170 m
MJ-24	Peaty, wet meadow along Soper River, ca. 0.5 km north of Mount Joy, 63°15'3"N, 69°36'6"W ± 1 m, elev. 86 m
MJ-25	Rocky slope near top of large hill overlooking “Panorama Flats” and Soper River, 63°15'32"N, 69°34'58"W ± 5 m, elev. 211 m
MJ-26	Sandy, seasonally wet depression surrounded by dense birch thickets below “Panorama Falls”, 63°15'24"N, 69°35'4"W ± 5 m, elev. 100 m
MJ-27	Sedge meadow along Soper River at base of large hill overlooking “Panorama Flats”, ca. 2 km north of Mount Joy, 63°15'51"N, 69°35'35"W ± 1 m, elev. 92 m
MJ-28	Soper River just upstream of “Panorama Flats”, 1.5 km northeast of Mount Joy, 63°15'49"N, 69°35'37"W, elev. 85 m
MJ-29	Soper River valley, west bank, ca. 13 km south of Mount Joy, moderate, south-facing slope, 63°09'39"N, 69°40'29"W, elev. 55 m
MJ-30	Soper River, across the river from Mount Joy, 63°15'01"N, 69°36'43"W ± 160 m, elev. 160 m
MJ-31	Soper River, east bank, 5.5 km downstream of Mount Joy, just above rapid, 63°13'48"N, 69°38'34"W ± 500 m, elev. 96 m
MJ-32	Soper River, east bank, ca. 0.5 km south of Mount Joy, 63°14'35"N, 69°36'25"W ± 10 m, elev. 110 m
MJ-33	Soper River, east bank, ca. 1 km south of Mount Joy, 63°14'19"N, 69°36'57"W, elev. 125 m
MJ-34	Soper River, east bank, ca. 1 km south of Mount Joy, 63°14'24"N, 69°37'12"W ± 5 m, elev. 80 m
MJ-35	Soper River, east bank, ca. 1 km south of Mount Joy, 63°14'25"N, 69°37'04"W ± 10 m, elev. 89 m
MJ-36	Soper River, east bank, ca. 1 km south of Mount Joy, 63°14'26"N, 69°36'52"W ± 2 m, elev. 108 m
MJ-37	Soper River, east bank, ca. 1 km south of Mount Joy, 63°14'28"N, 69°37'8"W ± 15 m, elev. 68 m
MJ-38	Soper River, east bank, ca. 1 km south of Mount Joy, 63°14'37"N, 69°36'50"W ± 1 m, elev. 89 m
MJ-39	Soper River, west bank, ca. 1 km downstream of Mount Joy, near the 1 m ledge, 63°14'26"N, 69°37'19"W ± 25 m, elev. 75 m
MJ-40	Steep, north-facing riverbank near Soper River at Mount Joy, 63°14'55"N, 69°36'21"W, elev. 85 m
MJ-41	Steep, rocky, northwest-facing slopes above horseshoe bend in Soper River at Mount Joy, 63°14'47"N, 69°36'52"W ±100 m, elev. 81 m
MJ-42	Steep, southwest-facing slope above creeks running into Soper River, overlooking “Panorama Flats”, 63°15'29"N, 69°35'04"W ± 50 m, elev. 155 m
MJ-43	Turfy sedge meadow along Soper River across from Mount Joy, 63°14'58"N, 69°36'27"W ± 50 m, elev. 80 m
MJ-44	West side of “Panorama Creek”, just below “Panorama Falls”, 2 km northeast of Mount Joy, 63°15'27"N, 69°34'51"W ± 10 m, elev. 120 m
MJ-45	South-facing slope of creek running from “Panorama Falls” to Soper River, adjacent to Mount Joy, 63°14'50"N, 69°36'20"W ± 1 m, elev. 84 m
**Katannilik Territorial Park, vicinity of Cascade River**	
CR-1	*Soper River at Cascade Creek [Cascade River], 63°10'N, 69°39'W [± 400 m]
CR-1a	*Soper River, near the mouth of Cascade River, 63°11'N, 69°44'W [± 400 m]
CR-2	Soper River valley, ca. 10.5 km south of Mount Joy, Cascade River valley, northwest-facing slope above Cascade River, downriver of Cascade Falls, 63°10'55"N, 69°36'14"W, elev. 185 m
CR-3	Soper River valley, ca. 10.5 km south of Mount Joy, east bank, steep, rocky, west-facing slope, 63°10'25"N, 69°37'53"W, elev. 150 m
CR-4	Soper River valley, ca. 10.5 km south of Mount Joy, lower Cascade River valley, south side, 63°10'48"N, 69°37'41"W ± 10 m, elev. 90 m
CR-5	Soper River valley, ca. 11 km south of Mount Joy, east bank, 63°10'17"N, 69°38'17"W ± 10 m, elev. 60 m
CR-6	Soper River, ca. 10.5 km south of Mount Joy, just south of the Cascade River valley, 63°10'37"N, 69°37'33"W ± 1 m, elev. 148 m
CR-7	Soper River, ca. 10.5 km south of Mount Joy, east bank, south of Cascade River/Cascade Falls, 63°10'24"N, 69°37'41"W ± 1 m, elev. 185 m
CR-8	Soper River, ca. 11 km south of Mount Joy, around waterfall creek south of Cascade River/Cascade Falls, 63°10'16"N, 69°38'18"W ± 25 m, elev. 63 m
CR-9	Soper River, ca. 11 km south of Mount Joy, east bank, 63°10'10"N, 69°38'32"W ± 5 m, elev. 60 m
CR-10	Soper River, ca. 11 km south of Mount Joy, east bank, 63°10'11"N, 69°38'51"W ± 5 m, elev. 50 m
CR-11	Soper River, ca. 11 km south of Mount Joy, 63°10'18"N, 69°38'1"W ± 50 m, elev. 112 m
CR-12	Soper River, ca. 12 km south of Mount Joy, east bank, 63°09'52"N, 69°38'55"W ± 20 m, elev. 45 m
CR-13	Soper River, ca. 12 km south of Mount Joy, east bank, 63°09'52"N, 69°38'46"W ± 10 m, elev. 45 m
CR-14	Soper River, ca. 12 km south of Mount Joy, east bank, 63°10'05"N, 69°38'42"W ± 1 m, elev. 66 m
CR-15	Soper River, ca. 12 km south of Mount Joy, east bank, 63°09'58"N, 69°38'37"W ± 50 m, elev. 56 m
CR-16	Soper River, ca. 12 km south of Mount Joy, 63°09'48"N, 69°39'13"W ± 25 m, elev. 53 m
**Katannilik Territorial Park, vicinity of Group/Warden Cabin #7**	
GC-1	Soper River valley, ca. 500 m up unnamed creek on the east bank of the river (just south of Group/Warden Cabin #7), ca. 12 km south of Mount Joy, 63°09'30"N, 69°40'2"W ± 5 m, elev. 43 m
GC-2	Soper River valley, ca. 500 m up unnamed creek on the east bank of the river (just south of Group/Warden Cabin #7), ca. 12 km south of Mount Joy, 63°09'31"N, 69°39'46"W ± 10 m, elev. 66 m
GC-3	Soper River valley, east bank, large sedge meadow with several small ponds, ca. 12.5 km south of Mount Joy, 0.5 km south of Group/Warden Cabin #7, 63°09'35"N, 69°40'3"W ± 75 m, elev. 41 m
GC-4	Soper River valley, muddy flats of creek running into river along east bank, ca. 12.5 km south of Mount Joy (0.5 km south of Group/Warden Cabin #7), 63°09'30"N, 69°40'02"W ± 5 m, elev. 43 m
GC-5	Soper River valley, west bank, ca. 12 km south of Mount Joy, meadow along the river opposite Group/Warden Cabin #7, 63°09'46"N, 69°40'08"W ± 10 m, elev. 49 m
GC-6	Soper River valley, west bank, ca. 12 km south of Mount Joy, meadow along the river opposite Group/Warden Cabin #7, 63°09'50"N, 69°39'55"W, elev. 40 m
GC-7	Soper River valley, west bank, ca. 12 km south of Mount Joy, meadow along the river opposite Group/Warden Cabin #7, 63°09'50"N, 69°40'02"W ± 20 m, elev. 40 m
GC-8	Soper River valley, west bank, ca. 13 km south of Mount Joy, moderate, south-facing slope, 63°09'39"N, 69°40'29"W, elev. 55 m
GC-9	Soper River valley, west bank, ca. 13 km south of Mount Joy, steep, rocky slope, 63°09'40"N, 69°40'24"W ± 2 m, elev. 60 m
GC-10	Soper River, east bank, 12 km south of Mount Joy along the river, at Group/Warden Cabin #7, 63°09'44"N, 69°39'28"W, elev. 50 m
**Katannilik Territorial Park, vicinity of Willow River**	
WR-1	*Koukdjuak River [Soper River], [63.1667°N, 69.9167°W ± 300 m]
WR-2	*Soper River, near mouth of Willow River, [63°09'N, 69°43'W ± 400 m]
WR-3	Soper River valley, west bank, ca. 13 km south of Mount Joy, 63°09'33"N, 69°40'49"W ± 5 m, elev. 48 m
WR-4	Soper River valley, west bank, ca. 14 km south of Mount Joy, 63°09'21"N, 69°41'41"W ± 50 m, elev. 41 m
WR-5	Soper River valley, west bank, near the confluence of Willow River, ca. 14 km south of Mount Joy, 63°09'18"N, 69°41'51"W ± 25 m, elev. 41 m
WR-6	Soper River valley, west bank, near the confluence of Willow River, ca. 14 km south of Mount Joy, 63°09'19"N, 69°41'52"W ± 5 m, elev. 51 m
WR-7	Soper River valley, west bank, near the confluence of Willow River, ca. 14 km south of Mount Joy, 63°09'20"N, 69°41'47"W ± 5 m, elev. 52 m
WR-8	Soper River valley, west bank, near the confluence of Willow River, ca. 14 km south of Mount Joy, 63°09'20"N, 69°41'48"W ± 10 m, elev. 54 m
WR-9	Soper River valley, west bank, near the confluence of Willow River, ca. 14 km south of Mount Joy, 63°09'22"N, 69°41'56"W ± 10 m, elev. 50 m
WR-10	Soper River valley, west bank, near the confluence of Willow River, ca. 14 km south of Mount Joy, 63°09'27"N, 69°42'24"W ± 5 m, elev. 95 m
**Katannilik Territorial Park, vicinity of Livingstone River**	
LR-1	*Livingstone Falls just before merging with Soper River, north of Lake Harbour [Kimmirut], [63.10756°N, 69.73259°W ± 850 m]
LR-2	*Livingstone River, [63°07'N, 69°44'W ± 850 m]
LR-3	Livingstone Falls, on Livingstone River (major tributary of Soper River), flats just above falls, 63°06'13"N, 69°44'41"W ± 5 m, elev. 50 m
LR-4	Livingstone Falls, on Livingstone River (major tributary of Soper River), flats just above falls, 63°06'13"N, 69°44'46"W ± 5 m, elev. 56 m
LR-5	Livingstone River (major tributary of Soper River), ca. 1 km from the confluence, 63°06'23"N, 69°45'24"W ± 20 m, elev. 50 m
LR-6	Livingstone River (major tributary of Soper River), ca. 2 km from the confluence, south bank, upper slope of the valley, 63°06'12"N, 69°46'16"W ± 10 m, elev. 370 m
LR-7	Livingstone River (major tributary of Soper River), north side, near confluence with Soper River, ca. 0.5 km northwest of Livingstone Falls, 63°06'32"N, 69°44'38"W ± 3 m, elev. 141 m
LR-8	Livingstone River (major tributary of Soper River), north side, near the confluence with Soper River, 63°06'30"N, 69°44'02"W ± 15 m, elev. 50 m
LR-9	Livingstone River (major tributary of Soper River), north side, near the confluence with Soper River, 63°06'33"N, 69°43'57"W ± 10 m, elev. 40 m
LR-10	Livingstone River (major tributary of Soper River), north side, near the confluence with Soper River, 63°06'46"N, 69°44'41"W ± 3 m, elev. 204 m
LR-11	Livingstone River (major tributary of Soper River), north side, near the confluence with Soper River, 63°06'59"N, 69°44'46"W ± 20 m, elev. 202 m
LR-12	Livingstone River, west side, along waterfall that runs into Livingstone River ca. 2 km from the confluence with Soper River, 1 km up the valley from Livingstone Falls, 63°06'23"N, 69°46'11"W ± 25 m, elev. 244 m
LR-13	Livingstone River, west side, along waterfall that runs into Livingstone River ca. 2 km from the confluence with Soper River, 1 km up the valley from Livingstone Falls, 63°06'25"N, 69°46'9"W ± 3 m, elev. 205 m
LR-14	Soper River, ca. 2 km from the confluence with Livingstone River, near the top of a steep slope above Soper River valley, 63°05'47"N, 69°45'37"W ± 10 m, elev. 390 m
LR-15	Soper River, ca. 2 km from the confluence with Livingstone River, upland plateau above Soper River valley, 63°05'42"N, 69°45'46"W ± 10 m, elev. 415 m
LR-16	Soper River, ca. 2 km south of confluence with Livingstone River, lower slopes of Soper valley, 63°05'48"N, 69°44'39"W ± 10 m, elev. 100 m
LR-17	Soper River, west bank, near the confluence with Livingstone River (north bank), 63°06'31"N, 69°44'02"W ± 1 m, elev. 66 m
LR-18	Soper River, west bank, near the confluence with Livingstone River, crystalline limestone ridge just north of confluence, 63°06'11"N, 69°44'03"W ± 5 m, elev. 78 m
LR-19	Soper River, west bank, near the confluence with Livingstone River, crystalline limestone ridge just north of confluence, 63°06'33"N, 69°44'11"W ± 2 m, elev. 70 m
LR-20	Soper River, west bank, near the confluence with Livingstone River, crystalline limestone ridge just north of confluence, 63°06'38"N, 69°44'14"W ± 20 m, elev. 100 m
LR-21	Soper River, west bank, near the confluence with Livingstone River, crystalline limestone ridge just north of confluence, 63°06'42"N, 69°44'17"W ± 5 m, elev. 110 m
LR-22	Soper River, west bank, near the confluence with Livingstone River, crystalline limestone ridge just north of confluence, 63°06'43"N, 69°44'13"W ± 5 m, elev. 98 m
LR-23	Soper River, west bank, near the confluence with Livingstone River, crystalline limestone ridge just north of confluence, 63°06'44"N, 69°44'18"W, elev. 120 m
LR-24	Soper River, west bank, near the confluence with Livingstone River, crystalline limestone ridge just north of confluence, 63°06'47"N, 69°44'17"W ± 1 m, elev. 90 m
LR-25	Soper River, west bank, near the confluence with Livingstone River, crystalline limestone ridge just north of confluence, 63°06'49"N, 69°44'28"W ± 5 m, elev. 140 m
LR-26	Soper River, west bank, near the confluence with Livingstone River, crystalline limestone ridge just north of confluence, 63°06'50"N, 69°44'11"W ± 10 m, elev. 81 m
LR-27	Soper River, west bank, near the confluence with Livingstone River, crystalline limestone ridge just north of confluence, 63°07'03"N, 69°44'24"W ± 2 m, elev. 103 m
LR-28	Soper River, west side at the confluence with Livingstone River, 63°06'33"N, 69°43'46"W ± 100 m, elev. 40 m
LR-29	Soper River, west side, 0.5–1 km south of confluence with Livingstone River, 63°06'20"N, 69°43'22"W ± 20 m, elev. 30 m
LR-30	Soper River, west side, just above the confluence with Livingstone River, 63°06'46"N, 69°44'27"W ± 5 m, elev. 150 m
LR-31	Soper River, west side, near Livingstone Falls (ca. 1 km up from the confluence of Livingstone and Soper rivers), 63°06'12"N, 69°44'10"W ± 6 m, elev. 6 m
LR-32	Soper River, west side, rocky flats above Soper River valley in vicinity of the confluence with Livingstone River, 63°05'47"N, 69°45'39"W ± 25 m, elev. 396 m
LR-33	Soper River, west side, rocky flats above Soper River valley in vicinity of the confluence with Livingstone River, 63°05'54"N, 69°46'06"W ± 3 m, elev. 429 m
LR-34	Soper River, west side, rocky flats above Soper River valley in vicinity of the confluence with Livingstone River, 63°06'37"N, 69°45'57"W ± 5 m, elev. 404 m
LR-35	Soper River, west side, south of Livingstone Falls, 63°05'22"N, 69°44'22"W ± 5 m, elev. 67 m
LR-36	Livingstone River (major tributary of Soper River), north side, near the confluence with Soper River, 63°07'14"N, 69°44'59"W ± 5 m, elev. 189 m
LR-37	Livingstone River (major tributary of Soper River), north side, near the confluence with Soper River, 63°06'35"N, 69°44'38"W ± 10 m, elev. 107 m
**Katannilik Territorial Park**	
LC-1	Soper River, 5 km south (downstream) of the confluence with Livingstone River, east bank, 63°04'27"N, 69°42'16"W ± 10 m, elev. 30 m
LC-2	Soper River, 5 km south (downstream) of the confluence with Livingstone River, east bank, 63°04'32"N, 69°42'11"W ± 20 m, elev. 30 m
LC-3	Soper River, 9.5 km south (downstream) of the confluence with Livingstone River, west bank, willow stands in gullies at base of east-facing slope, 63°02'32"N, 69°42'47"W ± 30 m, elev. 25 m
LC-4	Soper River, 9.5 km south (downstream) of the confluence with Livingstone River, west bank, willow stands in gullies at base of east-facing slope, 63°02'30"N, 69°42'46"W ± 10 m, elev. 20 m
**Katannilik Territorial Park, vicinity of Emergency Cabin #8**	
EC-1	Soper River, 18.5 km downstream (south) of its confluence with the Livingstone River, 1.5 km south of Emergency Cabin #8, east bank of the river, 62°58'45"N, 69°43'01"W ± 10 m, elev. 23 m
EC-2	Soper River, 18.5 km downstream (south) of its confluence with the Livingstone River, 1.5 km south of Emergency Cabin #8, east bank of the river, 62°58'48"N, 69°42'56"W ± 50 m, elev. 13 m
EC-2	Soper River, 18.5 km downstream (south) of its confluence with the Livingstone River, 1.5 km south of Emergency Cabin #8, east bank of the river, 62°58'48"N, 69°42'56"W ± 15 m, elev. 13 m
EC-3	Soper River, 18.5 km downstream (south) of its confluence with the Livingstone River, 1.5 km south of Emergency Cabin #8, east bank of the river, 62°58'48"N, 69°43'08"W ± 10 m, elev. 22 m
EC-4	Soper River, 18.5 km downstream (south) of its confluence with the Livingstone River, 1.5 km south of Emergency Cabin #8, east bank of the river, 62°59'40"N, 69°42'46"W ± 5 m, elev. 12 m
EC-5	Soper River, 18.5 km downstream (south) of its confluence with the Livingstone River, 1.5 km south of Emergency Cabin #8, flats south of the cabin, 62°59'18"N, 69°42'28"W ± 10 m, elev. 24 m
EC-6	Soper River, 18.5 km downstream (south) of its confluence with the Livingstone River, 2 km south of Emergency Cabin #8, east bank of the river, 62°58'46"N, 69°42'46"W, elev. 35 m
EC-7	Soper River, 18.5 km downstream (south) of its confluence with the Livingstone River, 2 km south of Emergency Cabin #8, east bank of the river, 62°59'02"N, 69°43'01"W, elev. 20 m
EC-8	Soper River, 18.5 km downstream (south) of its confluence with the Livingstone River, 2 km south of Emergency Cabin #8, east bank of the river, 62°59'08"N, 69°42'17"W ± 15 m, elev. 27 m
EC-9	Soper River, 18.5 km downstream (south) of its confluence with the Livingstone River, 2 km south of Emergency Cabin #8, east bank of the river, 62°59'10"N, 69°42'57"W ± 4 m, elev. 25 m
EC-10	Soper River, 18.5 km downstream (south) of its confluence with the Livingstone River, 2 km south of Emergency Cabin #8, east bank of the river, 62°59'13"N, 69°42'48"W ± 100 m, elev. 28 m
EC-11	Soper River, 18.5 km downstream (south) of its confluence with the Livingstone River, 2 km south of Emergency Cabin #8, west side of the river, 62°59'07"N, 69°43'15"W, elev. 20 m
EC-12	Soper River, 18.5 km downstream (south) of its confluence with the Livingstone River, 2 km south of Emergency Cabin #8, west side of the river, 62°59'08"N, 69°43'18"W ± 30 m, elev. 26 m
EC-13	Soper River, 18.5 km downstream (south) of its confluence with the Livingstone River, 2 km south of Emergency Cabin #8, west side of the river, 62°59'11"N, 69°43'42"W, elev. 43 m
EC-14	Soper River, 18.5 km downstream (south) of its confluence with the Livingstone River, 2 km south of Emergency Cabin #8, west side of the river, 62°59'17"N, 69°43'47"W, elev. 60 m
EC-15	Soper River, 18.5 km downstream (south) of its confluence with the Livingstone River, 2 km south of Emergency Cabin #8, west side of the river, 62°59'20"N, 69°43'41"W, elev. 36 m
EC-16	Soper River, 18.5 km downstream (south) of its confluence with the Livingstone River, 2 km south of Emergency Cabin #8, west side of the river, 62°59'21"N, 69°43'50"W, elev. 60 m
EC-17	Soper River, 18.5 km downstream (south) of its confluence with the Livingstone River, 2 km south of Emergency Cabin #8, west side of the river, 62°59'26"N, 69°43'42"W, elev. 60 m
EC-18	Soper River, 18.5 km downstream (south) of its confluence with the Livingstone River, 2 km south of Emergency Cabin #8, west side of the river, 62°59'28"N, 69°43'30"W, elev. 67 m
EC-19	Soper River, high water mark along riverbank, ca. 13 km downstream (south) of its confluence with the Livingstone River, 62°59'40"N, 69°42'46"W ± 200 m, elev. 35 m
EC-20	Soper River, just west (~300 m) of Emergency Cabin #8 along ATV track, 63°59'43"N, 69°42'32"W ± 100 m, elev. 20 m
**Vicinity of lapis lazuli site [not within Katannilik Territorial Park**]	
LS-1	*Soper River, lapis site, [62°57'N, 69°47.82'W ± 850 m]
LS-2	Soper River, west side, ca. 44.5 km south of Mount Joy along the river, ca. 17 km south of confluence with Livingstone River, 62°57'41"N, 69°48'03"W, elev. 20 m
LS-3	Soper River, west side, ca. 44.5 km south of Mount Joy along the river, ca. 17 km south of the confluence with Livingstone River, 62°57'46"N, 69°47'52"W ± 15 m, elev. 27 m
LS-4	Soper River, west side, ca. 44.5 km south of Mount Joy along the river, ca. 17 km south of the confluence with Livingstone River, 62°57'51"N, 69°47'53"W, elev. 33 m
**Katannilik Territorial Park, vicinity of Soper Falls**	
SF-1	*Rapids of Koukdjuak River [Soper River], 5 miles north of Lake Harbour [Kimmirut], [62.9097°N, 69.8375°W ± 600 m]
SF-2	*Soper River at Soper Falls, [62.9093°N, 69.8404°W ± 300 m], elev. 500 m
SF-2	*Soper River, at waterfall [Soper Falls] into Soper Lake [Tasiujarjuaq], [62.9093°N, 69.8404°W ± 300 m], elev. 500 m
SF-3	Across Soper Lake [Tasiujarjuaq] from Soper Falls, 62°54'04"N, 69°51'08"W ± 25 m, elev. 24 m
SF-4	Sand flats above Soper Falls, north side of Soper River, 62°54'40"N, 69°49'59"W, elev. 15 m
SF-5	Soper Falls, north side of Soper River, 62°54'35"N, 69°50'43"W, elev. 20 m
SF-6	Soper Falls, north side of Soper River, 62°54'44"N, 69°50'32"W ± 50 m, elev. 40 m
SF-7	Soper Falls, northeast corner of Soper Lake [Tasiujarjuaq], base of crystalline limestone hill adjacent to meadow and sand flats, east-facing slope, 62°54'35"N, 69°50'45"W ± 20 m, elev. 5 m
SF-8	Soper Falls, northeast corner of Soper Lake [Tasiujarjuaq], calcareous hill on north side of bay, 62°54'19"N, 69°51'08"W ± 5 m, elev. 36 m
SF-9	Soper Falls, south side of Soper Lake [Tasiujarjuaq], just southeast of Soper Falls, 62°51'17"N, 69°50'41"W ± 5 m, elev. 41 m
SF-10	Soper Falls, south side of Soper Lake [Tasiujarjuaq], just southeast of Soper Falls, 62°54'01"N, 69°50'54"W ± 1 m, elev. 6 m
SF-11	Soper Falls, south side of Soper Lake [Tasiujarjuaq], just southeast of Soper Falls, 62°54'20"N, 69°50'13"W, elev. 15 m
SF-12	Soper Falls, south side of Soper Lake [Tasiujarjuaq], vicinity of territorial park campground above Soper Falls, along ATV trail on a ridge, 62°54'20"N, 69°50'22"W ± 50 m, elev. 63 m
SF-13	Soper Falls, south side of Soper Lake [Tasiujarjuaq], vicinity of territorial park campground above Soper Falls, along ATV trail on a ridge, 62°54'22"N, 69°50'10"W ± 20 m, elev. 44 m
SF-14	Soper Falls, south side of Soper Lake [Tasiujarjuaq], vicinity of territorial park campground above Soper Falls, 62°54'33"N, 69°50'12"W ± 3 m, elev. 22 m
SF-15	Soper Falls, vicinity of territorial park campground, south side of Soper River, 62°54'26"N, 69°50'29"W, elev. 30 m
SF-16	Soper Falls, vicinity of territorial park campground, south side of Soper River, 62°54'28"N, 69°50'34"W, elev. 5 m
SF-17	Soper Falls/Soper Lake [Tasiujarjuaq], south side of Soper River, 62°54'04"N, 69°50'52"W ± 5 m, elev. 3 m
SF-18	Soper Falls/Soper Lake [Tasiujarjuaq], south side of Soper River, 62°54'06"N, 69°51'02"W ± 5 m, elev. 8 m
SF-19	Soper Falls/Soper Lake [Tasiujarjuaq], south side of Soper River, 62°54'08"N, 69°51'46"W ± 5 m, elev. 6 m
SF-20	Soper Falls/Soper Lake [Tasiujarjuaq], south side of Soper River, 62°54'17"N, 69°50'35"W ± 3 m, elev. 23 m
SF-21	Soper Falls/Soper Lake [Tasiujarjuaq], south side of Soper River, 62°54'33"N, 69°50'16"W ± 5 m, elev. 19 m
SF-22	Vicinity of territorial park campground, terminus of Soper River, just southeast of Soper Falls, 62°54'13"N, 69°49'39"W ± 13 m, elev. 13 m
SF-23	Vicinity of territorial park campground, terminus of Soper River, just southeast of Soper Falls, 62°54'27"N, 69°49'49"W ± 3 m, elev. 16 m
SF-24	Vicinity of territorial park campground, terminus of Soper River, just southeast of Soper Falls, 62°54'30"N, 69°50'15"W ± 4 m, elev. 14 m
SF-25	Soper Falls, south side of Soper Lake, just southeast of Soper Falls, 62°54'6"N, 69°50'52"W ± 3 m, elev. 6 m
SF-26	Soper Falls, south side of Soper Lake, just southeast of Soper Falls, 62°54'8"N, 69°50'42"W ± 1 m, elev. 6 m
SF-27	Soper Falls, vicinity of territorial park campground, large floodplain at the terminus of Soper River, just southeast of Soper Falls, 62°54'30"N, 69°49'56"W ± 20 m, elev. 14 m
SF-28	Soper Falls, south side of Soper Lake [Tasiujarjuaq], just southeast of Soper Falls, 62°54'01"N, 69°50'48"W ± 10 m, elev. 6 m
**Katannilik Territorial Park, Tasiujarjuaq**	
TJ-1	Small unnamed island on Soper Lake [Tasiujarjuaq] (eider duck colony), 62°53'16"N, 69°53'26"W, elev. 2–10 m
TJ-2	Small, unnamed island on Soper Lake [Tasiujarjuaq] (eider duck colony), 62°53'19"N, 69°53'24"W, elev. 10 m
TJ-3	Small, unnamed island on Soper Lake [Tasiujarjuaq] (eider duck colony), 62°53'6"N, 69°53'18"W ± 25 m, elev. 9 m
TJ-4	Soper Lake [Tasiujarjuaq], southeast corner, Kimmirut boat landing, 62°51'45"N, 69°52'56"W ± 5 m, elev. 16 m
TJ-5	Vicinity of Kimmirut, Reversing Falls, between southeast corner of Soper Lake [Tasiujarjuaq] and coastal bay, 62°51'31"N, 69°59'16"W ± 3 m, elev. 0 m
TJ-6	Vicinity of Kimmirut, Reversing Falls, between southeast corner of Soper Lake [Tasiujarjuaq] and coastal bay, 62°51'34"N, 69°55'14"W, elev. 0 m
**Kimmirut and vicinity**	
KM-1	*Lake Harbour [Kimmirut], [62.84667°N, 69.87194°W ± 4000 m]
KM-2	*2 miles east of Lake Harbour [Kimmirut], [62°51'N, 69°50'W]
KM-3	*Kimmirut, hameau, [62.84667°N, 69.87194°W ± 500 m]
KM-4	*Kimmirut, après le magasin Northern, en haut de la côte de sable, [62.85056°N, 69.87111°W]
KM-5	Kimmirut, ca. 5 km south of Reversing Falls at the end of Soper Lake, road between falls and hamlet, 62°51'33"N, 69°54'42"W ± 20 m, elev. 45 m
KM-6	Kimmirut, east-facing, lush, grassy slopes just above the coast, 62°50'52"N, 69°52'09"W ± 5 m, elev. 9 m
KM-7	Kimmirut, east side of hamlet, slope above the road south of the police station, 62°50'39"N, 69°52'14"W ± 5 m, elev. 50 m
KM-8	Kimmirut, north end of Fundo Lake below Taqaiqsirvik Campground, 62°50'50"N, 69°53'40"W ± 25 m, elev. 35 m
KM-9	Kimmirut, north end of Fundo Lake, moderate, calcareous, rocky slopes below Taqaiqsirvik Territorial Park, 62°50'49"N, 69°53'27"W, elev. 60 m
KM-10	Kimmirut, north end of hamlet, near Fundo Lake, 62°50'55"N, 69°53'42"W ± 5 m, elev. 51 m
KM-11	Kimmirut, north end of hamlet, near Taqaiqsirvik Territorial Park, 62°50'58"N, 69°53'30"W ± 50 m, elev. 86 m
KM-12	Kimmirut, north end of hamlet, Taqaiqsirvik Territorial Park, 62°50'56"N, 69°53'18"W ± 100 m, elev. 70 m
KM-13	Kimmirut, northwest end of Fundo Lake, ca. 2 km west of hamlet, 62°50'36"N, 69°54'10"W ± 25 m, elev. 30 m
KM-14	Kimmirut, rocky sand slope between Northern Store and coast, 62°50'57"N, 69°52'12"W
KM-15	Kimmirut, south end of hamlet, below garbage dump and above high tide line at the coast, 62°50'26"N, 69°52'20"W ± 50 m, elev. 68 m
KM-16	Kimmirut, south end of hamlet, below garbage dump and above high tide line at the coast, 62°50'26"N, 69°52'13"W ± 5 m, elev. 0–8 m
KM-16	Kimmirut, south end of hamlet, below garbage dump and above high tide line at the coast, 62°50'26"N, 69°52'13"W ± 10 m, elev. 12 m
KM-17	Kimmirut, south end of hamlet, one block southeast of school, 62°50'47"N, 69°52'11"W ± 5 m, elev. 14 m
KM-18	Kimmirut, south end of hamlet, rocky slope immediately opposite the entrance to the Kamik Co-op store, 62°50'43"N, 69°52'05"W, elev. 20 m
KM-19	Kimmirut, west end of Fundo Lake, ca. 2 km west of hamlet, 62°50'44"N, 69°54'06"W ± 25 m, elev. 40 m
KM-20	*Lake Harbour [Kimmirut], [62.770°N, 69.7967°W ± 9000 m]
**Pleasant Inlet**	
PI-1	Pleasant Inlet, ca. 10 km south of Reversing Falls at the end of Soper Lake, west of Kimmirut, west side of the inlet, 62°47'22"N, 69°59'51"W ± 50 m, elev. 10–25 m
PI-2	Pleasant Inlet, small unnamed island ca. 10 km south of Reversing Falls at the end of Soper Lake, west of Kimmirut, low rocky island with sand flats, 62°47'10"N, 69°59'02"W ± 50 m, elev. 0 m
PI-3	Pleasant Inlet, west of Kimmirut, south of Reversing Falls at the end of Soper Lake, 62°48'22.2"N, 69°57'02"W, elev. 0 m

We dried specimens in a standard plant press in the field. During processing, we subsampled specimens for a small amount of leaf tissue preserved in silica gel desiccant; we tagged the plant subsampled when possible. We have deposited all tissue samples in the Canadian Museum of Nature’s National Biodiversity Cryobank of Canada. J.M. Saarela, P.C. Sokoloff and L.J. Gillespie identified specimens. We have deposited a complete set of specimens in the National Herbarium of Canada (**CAN**) at the Canadian Museum of Nature and have distributed duplicates to the following herbaria, as indicated in the specimen citations: University of Alaska Museum of the North (**ALA**); University of Alberta Vascular Plant Herbarium (**ALTA**); Arizona State University (**ASU**); Gray Herbarium, Harvard University (**GH**); University of Michigan (**MICH**); Bell Museum, University of Minnesota (**MIN**); Missouri Botanical Garden (**MO**); Marie-Victorin Herbarium, University of Montreal (**MT**); The Rooms Provincial Museum, Newfoundland and Labrador (**NFM**); William and Lynda Steere Herbarium, New York Botanical Garden (**NY**); Botanical Museum, Oslo (**O**); Herbier Louis-Marie, Université Laval (**QFA**); Norwegian University of Science and Technology (**TRH**); Beaty Biodiversity Museum, University of British Columbia (**UBC**); United States National Herbarium, National Museum of Natural History, Smithsonian Institution (**US**); Intermountain Herbarium, Utah State University (**UTC**); University of Victoria (**UVIC**); University of Manitoba Herbarium (**WIN**); Wilfred Laurier University (**WLU**); University of Washington (**WTU**).

### ﻿Herbarium research

To generate a comprehensive checklist of the study area’s vascular flora, we attempted to account for all plant collections gathered therein. We searched the literature, herbaria and online collection databases to locate vascular plant specimens collected in the study area. We manually searched the collections at CAN, DAO, MIN, QFA and H (where we only searched through the monocots). Through direct enquiries to collection managers and searches on the Global Biodiversity Information Facility (**GBIF**) platform, we examined and confirmed specimen images from the E.C. Smith Herbarium at Acadia University (**ACAD**), ALTA, the general herbarium of vascular plants at the University of Copenhagen (**C**), the herbarium at the Field Museum of Natural History (**F**), GH, the herbarium at the Royal Botanic Gardens, Kew (**K**), the R.L. McGregor Herbarium at the University of Kansas (**KANU**), the herbarium at the Botanical Museum, Lund University (**LD**), MICH, MIN, MO, MT, the herbarium at McGill University, Macdonald Campus (**MTMG**), NY, O, the herbarium at the Swedish Museum of Natural History (**S**), the Green Plant Herbarium, Royal Ontario Museum (**TRT**), US, UTC and the herbarium at the Royal British Columbia Museum (**V**).

Malte’s specimen labels do not include collection numbers, but they bear unique six-digit numbers from an older National Herbarium of Canada number series. We used these numbers to identify duplicate specimens amongst herbaria and we cite this number in square brackets for each of Malte’s collections in the checklist to unambiguously refer to them. In addition to the specimen label from the National Herbarium of Canada, a subset of Malte’s collections housed at GH include handwritten collection numbers on separate pieces of paper, which we assume are fragments of the paper in which he pressed his specimens. To match numbered collections at GH with duplicates at CAN and elsewhere that exclude these numbers, we relied on the six-digit number on the label. We cite these in the checklist as *s.n./[collection number*] followed by the six-digit number in square brackets.

Soper’s specimen labels indicate that on 1 July 1931, the day he canoed upriver, he collected at 63°10'N, 69°55'W. These coordinates, however, point to a location 11.5 km northwest of the Soper and Livingstone rivers’ confluence on the plateau west of the Soper River. Dalton (2010) stated that, during this trip, the team stopped to collect at 63°50'N, but this is also an error because this latitude is north of Iqaluit. Soper (1936) described willows up to 12 ft [3.7 m] high at this location. He indicated that the group lined their canoe from this collecting locality ca. 3 mi [4.8 km] upriver while passing “rapids, cascades, and falls” and that two-thirds of the way up the river, the group reached a large waterfall, which he named “Cascade Falls.” The Soper and Willow rivers’ confluence is ca. three miles south of Cascade River, so we infer that on 1 July 1931, Soper collected around the large willow patch (*Salixplanifolia* Pursh) at the mouth of the Willow River, an area now part of Katannilik Territorial Park.

We combined all data obtained from physical and online herbarium searches into a spreadsheet (Suppl. material 1). We manually cleaned the dataset to standardize collector names, date format and locality descriptions amongst specimens gathered by the same collector at the same site. We combined records of duplicate specimens housed in different herbaria into single records, maintaining information on their disposition(s). Most of the earlier collections either lacked geographical coordinates or had imprecise ones. We georeferenced these sites following standard point-radius protocols, including estimates of coordinate uncertainty in metres. Fig. 6 shows the general locations of previous collectors’ collecting sites. We made maps with ArcMap 10.5.1 (Esri, Redlands, California 2016).

### ﻿Annotated checklist

We summarize the study area’s vascular flora in an annotated checklist. Lycophyte and monilophyte classifications follow Pteridophyte Phylogeny Group (2016). Angiosperm classification follows Angiosperm Phylogeny Group IV (2016). Within families, genera and species are listed alphabetically. We base taxonomy at genus, species and infraspecific levels on consideration of relevant global literature, including Elven et al. (2011), Flora of North America treatments (Flora of North America Editorial Committee 1993+) and recent revisions and nomenclatural updates, including Wiegleb et al. (2017), Barberá et al. (2019) and Morin (2020). For each species, we list selected synonyms, focussing on names used in critical Canadian (Polunin 1940; Porsild and Cody 1980; Aiken et al. 2007; Payette 2013, 2015, 2018) and international (Elven et al. 2011) Arctic taxonomic treatments, recent national or continental treatments, particularly Flora of North America, and other relevant taxonomic works. English common names follow Brouillet et al. (2010+). Global distribution summaries follow Elven et al. (2011). For all of our 2012 collections, the list of collectors is J.M. Saarela, L.J. Gillespie, P.C. Sokoloff and R.D. Bull; we abbreviate this in the checklist as Saarela et al.

In the annotated checklist, we indicate whether each taxon has been previously recorded in Kimmirut or Katannilik Territorial Park (i.e., from within the area currently the park). Previously recorded means the taxon was mentioned or mapped in the study area in one or more publications (Polunin 1940; Porsild 1957, 1964; Porsild and Cody 1980; Aiken et al. 2007). Usually, one or all vouchers we cite are the ones previous authors cited or mapped. Polunin (1940) cited voucher material from the study area, except for some common taxa he recorded as “everywhere-numerous records from almost all localities” (e.g., *Carexmembranacea*). Later authors, however, did not cite the specimens they mapped (Porsild 1957, 1964; Porsild and Cody 1980; Aiken et al. 2007). We also indicate taxa newly recorded for Kimmirut, the park, Pleasant Inlet and the study area. New records include collections we made in 2012, older collections overlooked in earlier publications and collections known to previous authors that were misidentified or for which a taxonomic concept has been revised. For all taxa recorded in the study area, we cite one or more specimens.

We also summarize the known distribution on Baffin Island for each taxon recorded in the study area. These summaries include site-level descriptions of collection localities on the Meta Incognita Peninsula, Foxe Peninsula and Hall Peninsula, as well as on islands immediately adjacent to Baffin Island, such as Dorset, Mallik and Resolution islands. We refer to these regions as southern Baffin Island. Sometimes, we summarize all known occurrences of a taxon on Baffin Island (for example, for taxa known from few collections across the island). We based these summaries on published literature, primarily Aiken et al. (2007) and Global Biodiversity Information Facility (GBIF)-mediated collection data that have not been published or included in maps. We accepted records discovered on GBIF for which we could confirm a specimen determination, based on an image. For all sites on Baffin Island for which records have not been published, we cite the collector, collector number, herbarium and accession or barcode number. Furthermore, through careful cross-checking of vouchered occurrence data on GBIF with distribution maps from Aiken et al. (2007), based primarily on material housed at CAN, we revealed some mapping errors; we describe these errors in the text.

We previously published a subset of our 2012 collections that are new records for one or more of Baffin Island (or, in one case, where known from one previous record), the eastern Canadian Arctic Archipelago, the Canadian Arctic Archipelago or Nunavut (Gillespie et al. 2015). These taxa are *Cryptogrammastelleri* (S.G.Gmel.) Prantl (Pteridaceae), Carexbrunnescens(Pers.)Poir.subsp.brunnescens (Cyperaceae), Calamagrostisneglectasubsp.groenlandica (Schrank) Matuszk. (as C.strictasubsp.groenlandica (Schrank) Á.Löve), HordeumjubatumL.subsp.jubatum, Leymusmollis(Trin.)Pilg.subsp.mollis (Poaceae), *Triglochinpalustris* L. (Juncaginaceae), *Corallorhizatrifida* Châtel., Platantheraobtusata(Banks ex Pursh)Lindl.subsp.obtusata (Orchidaceae), *Arenarialongipedunculata* Hultén (Caryophyllaceae), Orthiliasecundasubsp.obtusata (Turcz.) Böcher (Ericaceae), *Utriculariaochroleuca* R.W.Hartm. (Lentibulariaceae), *Primulaegaliksensis* Wormsk. (Primulaceae), *Coptidium×spitsbergense* (Hadač) Luferov & Prob. (Ranunculaceae) and *Salixfuscescens* Anderss. (Salicaceae). In that paper, we also included our study area collections of *Andromedapolifolia* L. and *Pinguiculavulgaris* L.; we reported both as new in other parts of the Canadian Arctic Archipelago. We do not repeat the information Gillespie et al. (2015) provided for the new records, including summaries of Arctic distribution, taxonomic history and photographs. However, we cite all known voucher material for each taxon from the study area, including specimens Gillespie et al. (2015) cited.

## ﻿Results

Our final dataset includes 1596 unique vascular plant collections from the study area. We gathered 838 of these collections in 2012 and other collectors gathered 758 before 2012. Our 2012 collections comprise 676 gatherings from Katannilik Territorial Park, 11 from the lapis lazuli site outside the park, 111 from Kimmirut and vicinity and 41 from Pleasant Inlet. Of the prior collections, 671 were gathered in Kimmirut and vicinity, 81 within what is now Katannilik Territorial Park and six at the lapis lazuli site. The number of specimens recorded in our database gathered by each prior collector is as follows: Aiken & Iles (27), Archambault (18), Blake (1), Dutilly (83), Fleming (2), Johansen (35), Malte (316), Oldenburg (44), Polunin (132), Sanson (2), Soper (96) and Tallman (2).

The study area’s vascular flora comprises 35 families, 98 genera, 211 species, two nothospecies and seven infraspecific taxa (Tables 2, 3). We recorded 196 taxa in Katannilik Territorial Park (191 species, three infraspecific taxa and two nothospecies), 170 taxa in Kimmirut and vicinity (166 species, three infraspecific taxa and one nothospecies) and 41 species from Pleasant Inlet (Table 3). We newly recorded 51 taxa (48 species, two infraspecific taxa and one nothospecies) in 22 families in the study area, 145 taxa (141 species, two nothospecies and two subspecies) in 26 families in Katannilik Territorial Park and 15 species in nine families in Kimmirut and vicinity (Table 3). All 41 species we collected along Pleasant Inlet are first records for that area. At the genus level, Poaceae is the most diverse within the study area, with 15 genera, followed by Ericaceae with 11, Caryophyllaceae with nine, Brassicaceae with seven and Asteraceae with six. Two families comprise four genera, three comprise three, eight comprise two and 17 comprise one. At the species level and below, the largest families, each with ten or more species, are Cyperaceae (38 species), Poaceae (24), Brassicaceae (16), Caryophyllaceae (16), Ericaceae (13), Saxifragaceae (11), Asteraceae (11) and Juncaceae (10). Two infraspecific taxa occur within the study area in *Eriophorumscheuchzeri* Hoppe, *Poaarctica* R.Br. and *Potentillahyparctica* Malte. We did not count putative hybrids between *Salix* species (see comments under *S.arctophila* in the checklist) as separate taxa.

**Table 2.** Number of genera and species in each vascular plant family recorded from Katannilik Territorial Park, Kimmirut and Pleasant Inlet on Baffin Island, Nunavut, Canada.

Unranked clade	Order	Family	Genera	Species/Taxa
**Lycophytes**	Lycopodiales	Lycopodiaceae	2	3
**Monilophytes**	Equisetales	Equisetaceae	1	3
	Polypodiales	Cystopteridaceae	1	1
		Dryopteridaceae	1	1
		Pteridaceae	1	1
		Woodsiaceae	1	2
**Monocots**	Alismatales	Juncaginaceae	1	1
		Tofieldiaceae	1	1
	Asparagales	Orchidaceae	2	2
	Poales	Juncaceae	3	11
		Cyperaceae	4	38/40
		Poaceae	15	24/27
**Eudicots**	Ranunculales	Papaveraceae	1	2
		Ranunculaceae	2	7/8
	Saxifragales	Saxifragaceae	3	11
	Fabales	Fabaceae	2	6
	Rosales	Rosaceae	4	8/9
	Fagales	Betulaceae	1	1
	Celastrales	Celastraceae	1	1
	Malpighiales	Salicaceae	1	9
	Myrtales	Onagraceae	1	2
	Brassicales	Brassicaceae	7	15/16
	Caryophyllales	Plumbaginaceae	1	1
		Polygonaceae	3	3
		Caryophyllaceae	9	16/17
		Montiaceae	1	1
	Ericales	Primulaceae	1	1
		Diapensiaceae	1	1
		Ericaceae	11	13
	Boraginales	Boraginaceae	1	1
	Lamiales	Plantaginaceae	2	3
		Lentibulariaceae	2	2
		Orobanchaceae	2	6
	Asterales	Campanulaceae	2	2
		Asteraceae	6	11
	**20**	**35**	**98**	**211/220**

**Table 3.** Checklist of vascular plant taxa recorded from Katannilik Territorial Park, Kimmirut and vicinity, and Pleasant Inlet, with first records indicated for the park, Kimmirut and vicinity and the study area.

	Taxon	Katannilik Territorial Park	New to park	Kimmirut and vicinity	New to Kimmirut and vicinity	Pleasant Inlet	New to study area
LYCOPHYTES							
**Lycopodiaceae**	*Huperziaarctica* Sipliv.			•			
	*Huperziacontinentalis* Testo, A.Haines & A.V.Gilman	•	•	•	•	•	•
	*Spinulumannotinum* (L.) A.Haines	•		•			
MONILOPHYTES							
Equisetales							
**Equisetaceae**	Equisetumarvensesubsp.alpestre (Wahlenb.) Schönsw. & Elven	•	•	•			
	*Equisetumscirpoides* Michx.			•			
	*Equisetumvariegatum* Schleich. ex F.Weber & D.Mohr subsp. variegatum	•		•			
Polypodiales							
**Cystopteridaceae**	*Cystopterisfragilis* (L.) Bernh.	•	•	•			
**Dryopteridaceae**	*Dryopterisfragrans* (L.) Schott	•					
	*Cryptogrammastelleri* (S.G.Gmel.) Prantl			•	•		•
**Woodsiaceae**	*Woodsiaalpina* (Bolton) Gray	•	•				•
	*Woodsiaglabella* R.Br	•	•	•			
MONOCOTS							
**Juncaginaceae**	*Triglochinpalustris* L.	•	•	•	•		•
**Tofieldiaceae**	*Tofieldiapusilla* (Michx.) Pers.	•	•	•			
**Orchidaceae**	*Corallorhizatrifida* Châtel.	•	•				•
	Platantheraobtusata(Banks ex Pursh)Lindl.subsp.obtusata	•	•				•
**Juncaceae**	JuncusarcticusWilld.subsp.arcticus	•	•	•			
	*Juncusbiglumis* L.	•	•	•			
	*Juncusleucochlamys* V.J.Zinger ex V.I.Krecz.	•	•	•			
	Juncustriglumissubsp.albescens (Lange) Hultén	•		•			
	*Luzulaconfusa* Lindeb.	•	•	•		•	
	*Luzulagroenlandica* Böcher	•	•				•
	Luzulamultiflorasubsp.frigida (Buchenau) V.I.Krecz.	•					
	*Luzulanivalis* (Laest.) Spreng.	•	•	•			
	*Luzulaspicata* (L.) DC.	•	•	•			
	*Luzulawahlenbergii* Rupr.	•	•	•			
	*Oreojuncustrifidus* (L.) Záv.Drábk. & Kirschner			•			
**Cyperaceae**	Carexaquatilissubsp.stans (Drejer) Hultén			•			
	*Carexarctogena* Harry Sm.	•	•	•			
	*Carexatrofusca* Schkuhr	•	•	•			
	*Carexbicolor* All.	•	•	•			
	CarexbigelowiiTorr. ex Schwein.subsp.bigelowii	•		•			
	Carexbrunnescens(Pers.)Poir.subsp.brunnescens	•	•				•
	Carexcapillarissubsp.fuscidula (V.I.Krecz. ex T.V.Egorova) Á.Löve & D.Löve	•	•	•			
	*Carexchordorrhiza* L.f.	•	•	•			
	Carexfuliginosasubsp.misandra (R.Br.) Nyman	•	•	•			
**Cyperaceae**	*Carexglacialis* Mack.	•	•	•			
	CarexglareosaWahlenb.subsp.glareosa	•	•	•		•	
	*Carexgynocrates* Wormsk. ex Drejer	•	•	•			
	*Carexholostoma* Drejer	•	•	•			
	*Carexkrausei* Boeckeler	•	•				•
	*Carexlachenalii* Schkuhr	•	•	•			
	*Carexmarina* Dewey	•	•	•			
	*Carexmaritima* Gunnerus	•	•	•		•	
	*Carexmembranacea* Hook.	•		•			
	*Carexmicroglochin* Wahlenb.	•	•	•	•		•
	*Carexmyosuroides* Vill.	•	•				
	*Carexnardina* Fr.	•	•	•		•	
	*Carexnorvegica* Retz.	•	•	•			
	*Carexrariflora* (Wahlenb.) Sm.	•	•	•			
	*Carexrupestris* All.	•	•	•			
	*Carexsaxatilis* L.	•	•	•			
	CarexscirpoideaMichx.subsp.scirpoidea	•	•	•			
	Carexsimpliciusculasubsp.subholarctica (T.V.Egorova) Saarela	•	•	•			
	*Carexsubspathacea* Wormsk.					•	•
	Carexsupinasubsp.spaniocarpa (Steud.) Hultén	•	•	•			
	*Carexursina* Dewey	•	•	•		•	
	*Carexvaginata* Tausch	•	•	•			
	*Carexwilliamsii* Britton	•	•				•
	*Eleocharisacicularis* (L.) Roem. & Schult.	•	•	•			
	*Eriophorumangustifolium* Honck.	•	•	•			
	*Eriophorumcallitrix* C.A.Mey.	•		•			
	Eriophorum×mediumsubsp.album J.Cay.	•	•	•			
	Eriophorumscheuchzerisubsp.arcticum M.S.Novos.			•	•		•
	EriophorumscheuchzeriHoppesubsp.scheuchzeri	•	•	•			
	Eriophorumvaginatumsubsp.spissum (Fernald) Hultén	•		•			
	Trichophorumcespitosum(L.)Hartm.subsp.cespitosum	•		•			
**Poaceae**	*Agrostismertensii* Trin.	•	•	•			
	*Alopecurusborealis* Trin.			•			
	Anthoxanthummonticolasubsp.alpinum (Sw. ex Willd.) Soreng	•		•			
	Arctagrostislatifolia(R.Br.)Griseb.subsp.latifolia	•	•	•			
	*Arctophilafulva* (Trin.) Andersson	•	•				
	Calamagrostiscanadensissubsp.langsdorffii (Link) Hultén	•		•			
	Calamagrostisneglectasubsp.groenlandica (Schrank) Matuszk.	•	•				•
	*Calamagrostispurpurascens* R.Br.	•	•				•
	*Deschampsiasukatschewii* (Popl.) Roshev.	•	•	•		•	
	*Dupontiafisheri* R.Br.	•	•				
	FestucabrachyphyllaSchult. & Schult. f.subsp.brachyphylla	•	•	•		•	
**Poaceae**	Festucaproliferavar.lasiolepis Fernald	•	•				•
	Festucarubrasubsp.arctica (Hack.) Govor.	•	•				•
	FestucarubraL.subsp.rubra			•	•		•
	HordeumjubatumL.subsp.jubatum			•	•		•
	*Koeleriaspicata* (L.) Barberá, A.Quintanar, Soreng & P.M.Peterson	•	•	•			
	Leymusmollis(Trin.)Pilg.subsp.mollis	•	•				•
	Leymusmollissubsp.villosissimus (Scribn.) Á.Löve & D.Löve	•	•				•
	*Phippsiaalgida* (Sol.) R.Br.	•	•	•			
	PoaalpinaL.subsp.alpina	•	•	•			
	PoaarcticaR.Br.subsp.arctica	•	•	•			
	Poaarcticasubsp.caespitans Simmons ex Nannf.	•	•	•			
	PoaglaucaVahlsubsp.glauca	•	•	•			
	Poapratensissubsp.alpigena (Lindm.) Hiitonen	•	•	•			
	Puccinelliaphryganodessubsp.neoarctica (Á.Löve & D.Löve) Elven	•	•	•		•	
	Puccinelliatenellasubsp.langeana (Berlin) Tzvelev	•	•	•		•	
	*Puccinelliavaginata* (Lange) Fernald & Weath.			•	•		•
EUDICOTS							
**Papaveraceae**	*Papaverlabradoricum* (Fedde) Solstad & Elven	•	•	•			
	*Papaverlapponicum* (Tolm.) Nordh.	•	•	•			
**Ranunculaceae**	*Coptidiumlapponicum* (L.) Gand	•	•	•			
	*Coptidiumpallasii* (Schltdl.) Tzvelev			•			
	*Coptidium×spitsbergense* (Hadač) Luferov & Prob.	•	•				•
	*Ranunculusarcticus* Richardson	•	•	•			
	RanunculushyperboreusRottb.subsp.hyperboreus	•	•			•	
	*Ranunculusnivalis* L.	•					
	*Ranunculuspygmaeus* Wahlenb.	•					
	*Ranunculustrichophyllus* Chaix	•	•	•		•	
**Saxifragaceae**	*Chrysospleniumtetrandrum* Th.Fr.			•			
	*Micranthesfoliolosa* (R.Br.) Gornall	•	•	•	•	•	•
	*Micranthesnivalis* (L.) Small	•	•	•	•		•
	*Micranthestenuis* (Wahlenb.) Small	•	•	•	•		•
	*Saxifragaaizoides* L.	•	•	•			
	*Saxifragacernua* L.	•	•	•			
	*Saxifragacespitosa* L.	•	•	•		•	
	*Saxifragahyperborea* R.Br.	•	•			•	
	*Saxifragaoppositifolia* L.	•	•	•			
	*Saxifragapaniculata* Mill.	•	•	•			
	*Saxifragatricuspidata* Rottb.	•		•			
**Fabaceae**	*Astragalusalpinus* L.	•	•	•			
	*Astragaluseucosmus* B.L.Rob.	•	•	•		•	
	Oxytropisdeflexavar.foliolosa (Hook.) Barneby	•	•	•		•	
	*Oxytropismaydelliana* Trautv.	•		•		•	
	*Oxytropispodocarpa* Gray	•	•				•
	*Oxytropisterrae-novae* Fernald			•			
**Rosaceae**	DryasintegrifoliaVahlsubsp.integrifolia	•		•			
	Potentillaanserinasubsp.groenlandica Tratt.	•	•	•		•	
	*Potentillacrantzii* (Crantz) Beck	•	•				•
	PotentillahyparcticaMaltesubsp.hyparctica	•	•				•
	Potentillahyparcticasubsp.elatior (Abrom.) Elven & D.F.Murray	•		•			
	*Potentillanivea* L.	•	•	•			
	*Potentillapulchella* R.Br.			•			
	*Rubuschamaemorus* L.	•	•				•
	*Sibbaldiaprocumbens* L.	•	•				•
**Betulaceae**	*Betulaglandulosa* Michx.	•		•			
**Celastraceae**	*Parnassiakotzebuei* Cham. ex Spreng.	•	•	•			
**Salicaceae**	*Salixarctica* Pall.	•	•	•			
	*Salixarctophila* Cockerell ex A.Heller	•		•			
	SalixcalcicolaFernald & Wiegandvar.calcicola	•	•	•		•	
	*Salixfuscescens* Anderss.	•	•				•
	Salixglaucavar.cordifolia (Pursh) Dorn	•		•		•	
	*Salixherbacea* L.	•	•	•			
	*Salixplanifolia* Pursh	•					
	*Salixreticulata* L.	•		•			
	*Salixuva-ursi* Pursh	•		•		•	
**Onagraceae**	Chamaenerionangustifolium(L.)Scop.subsp.angustifolium	•					
	*Chamaenerionlatifolium* (L.) Sweet	•		•		•	
**Brassicaceae**	*Arabidopsisarenicola* (Richardson) Al-Shehbaz, Elven, D.F.Murray & Warwick	•	•	•		•	
	*Arabisalpina* L.	•	•	•			
	BrayaglabellaRichardsonsubsp.glabella			•			
	Brayaglabellasubsp.purpurascens (R.Br.) Cody	•	•	•			
	*Cardaminebellidifolia* L.	•	•	•			
	*Cardaminepolemonioides* Rouy	•	•				
	*Cochleariagroenlandica* L.	•	•	•			
	*Drabaalpina* L.	•	•	•			
	*Drabaarctica* J.Vahl	•	•				•
	*Drabacrassifolia* Graham			•			
	*Drabafladnizensis* Wulfen	•	•				•
	*Drabaglabella* Pursh	•		•		•	
	*Drabalactea* Adams	•	•				•
	*Drabanivalis* Lilj.	•		•			
	*Eutremaedwardsii R.Br.*	•		•			
	*Physariaarctica* (Wormsk. ex Hornem.) O’Kane & Al-Shehbaz	•	•	•			
**Plumbaginaceae**	*Armeriascabra* Pall. ex Roem. & Schult.	•	•	•			
**Polygonaceae**	*Bistortavivipara* (L.) Delarbre	•	•	•			
	*Koenigiaislandica* L.	•	•	•			
	*Oxyriadigyna* (L.) Hill	•	•	•		•	
**Caryophyllaceae**	*Arenariahumifusa* Wahlenb.	•	•	•		•	
	*Arenarialongipedunculata* Hultén	•	•				•
	*Cerastiumalpinum* L.	•		•			
	*Cerastiumarcticum* Lange			•			
	*Cherleriabiflora* (L.) A.J.Moore & Dillenb.	•	•	•			
**Caryophyllaceae**	Honckenyapeploidessubsp.diffusa (Hornem.) Hultén	•	•			•	•
	*Sabulinarossii* (R.Br. ex Richardson) Dillenb. & Kadereit	•	•				•
	*Sabulinarubella* (Wahlenb.) Dillenb. & Kadereit	•	•	•			
	*Sabulinastricta* (Sw.) Rchb.	•	•	•			
	Saginanodosasubsp.borealis G.E.Crow			•			
	*Sileneacaulis* (L.) Jacq.	•	•	•			
	*Sileneinvolucrata* (Cham. & Schltdl.) Bocquet	•	•	•		•	
	Sileneuralensissubsp.arctica (Fr.) Bocquet			•			
	Sileneuralensis(Rupr.)Bocquetsubsp.uralensis	•	•				•
	*Stellariahumifusa* Rottb.	•	•	•		•	
	*Stellarialongipes* Goldie	•		•			
	*Viscariaalpina* (L.) G.Don	•	•				•
**Montiaceae**	*Montiafontana* L.			•	•		•
**Primulaceae**	*Primulaegaliksensis* Wormskj.			•	•		•
**Diapensiaceae**	*Diapensialapponica* L.	•		•		•	
**Ericaceae**	*Andromedapolifolia* L.	•	•				•
	*Arctousalpina* (L.) Nied.	•	•	•			
	Cassiopetetragona(L.)D.Donsubsp.tetragona	•	•	•		•	
	*Empetrumnigrum* L.	•		•		•	
	*Harrimanellahypnoides* (L.) Coville	•	•	•		•	
	*Kalmiaprocumbens* (L.) Gift, Kron & P.F.Stevens ex Galasso, Banfi & F.Conti.	•	•	•		•	
	Orthiliasecundasubsp.obtusata (Turcz.) Böcher	•	•				•
	*Phyllodocecaerulea* (L.) Bab.	•		•		•	
	*Pyrolagrandiflora* Radius	•		•			
	*Rhododendronlapponicum* (L.) Wahlenb.	•		•			
	Rhododendrontomentosumsubsp.decumbens (Aiton) Elven & D.F.Murray	•		•			
	*Vacciniumuliginosum* L.	•		•			
	*Vacciniumvitis*-*idaea* subsp. minus (Lodd., G.Lodd. & W.Lodd.) Hultén	•	•	•			
**Boraginaceae**	Mertensiamaritimasubsp.tenella (Th.Fr.) Elven & Skarpaas			•		•	
**Plantaginaceae**	*Hippurislanceolata* Retz	•	•	•	•	•	•
	*Hippurisvulgaris* L.	•	•	•	•		•
	*Plantagomaritima* L.					•	•
**Lentibulariaceae**	*Pinguiculavulgaris* L.	•		•			
	*Utriculariaochroleuca* R.W.Hartm.	•	•				•
**Orobanchaceae**	*Bartsiaalpina* L.	•		•			
	*Pedicularisflammea* L.	•		•			
	*Pedicularishirsuta* L.	•					
	*Pedicularislabradorica* Wirsing	•		•			
	*Pedicularislanata* Willd. ex Cham. & Schltdl.	•		•			
	*Pedicularislapponica* L.	•		•			
**Campanulaceae**	*Campanularotundifolia* L.	•	•				•
	*Melanocalyxuniflora* (L.) Morin	•	•	•			
**Asteraceae**	Antennariaalpinasubsp.canescens (Lange) Chmiel.	•	•	•			
	Antennariafriesiana(Trautv.)E.Ekmansubsp.friesiana	•					
	Antennariamonocephalasubsp.angustata (Greene) Hultén	•		•			
	ArnicaangustifoliaVahlsubsp.angustifolia	•		•			
	ArtemisiaborealisPallassubsp.borealis	•		•			
	*Erigeroneriocephalus* J.Vahl	•	•				•
	*Erigeronhumilis* Graham	•		•		•	
	*Hulteniellaintegrifolia* (Richardson) Tzvelev	•		•			
	*Taraxacumceratophorum* (Ledeb.) DC.	•	•	•			
	*Taraxacumholmenianum* Sahlin	•	•				•
	*Taraxacumlapponicum* Kihlman ex Hand.-Mazz.	•	•	•			

## ﻿Discussion

Our study establishes baseline information on vascular plant diversity in Katannilik Territorial Park and Kimmirut and vicinity. The flora’s richness is within the 125 to 250 species range expected for local floras within Circumpolar Arctic Bioclimate Subzone D (CAVM Team 2003), of which the study area is part. Five of the families with the greatest species richness within the study area (Cyperaceae, Poaceae, Brassicaceae, Caryophyllaceae and Asteraceae) are amongst the eight families with the highest species richness in the circumpolar Arctic (Daniëls et al. 2013).

All vascular plant taxa recorded in the study area are native, except for two grass species that grew in Kimmirut: FestucarubraL.subsp.rubra and Hordeumjubatumsubsp.jubatum. The former species may have been seeded and the introduction pathway of the latter species is unknown (Gillespie et al. 2015). No information is available about their statuses in Kimmirut since we documented them in 2012. Periodic monitoring of these species in Kimmirut, particularly *Hordeumjubatum*, which is conspicuous and easy for non-botanists to recognize, would provide valuable information, such as whether they have persisted over time, are expanding within the region or are affecting natural communities. Although the occurrence of naturally-persisting non-native vascular plant species is currently rare in the Canadian Arctic Archipelago, milder climatic conditions, longer growing seasons and anthropogenic disturbance may facilitate a shift in the Arctic’s non-native vascular flora composition (Wasowicz et al. 2020). As such, we need regular surveys to detect non-native species introductions throughout the region, particularly in Arctic communities, where non-native species, often associated with disturbance, generally first appear and are first detected.

Many of the taxa we collected in 2012 have been reported in the study area in one or more treatments (Polunin 1940; Porsild 1957, 1964; Porsild and Cody 1980; Aiken et al. 2007), as summarized in the annotated checklist, often based on the collections gathered many decades ago. Nevertheless, our 2012 collections and review of existing collections newly documented 51 taxa in the study area, 145 in the park and 15 in Kimmirut and vicinity. We expected many new records, given the limited botanical exploration of the area in which we worked. Although researchers have collected considerably in the immediate Kimmirut area, the only botanists that have collected within what now is Katannilik Territorial Park are J.D. Soper in 1931 and Susan Aiken in 2002. Neither of these collectors comprehensively surveyed the region’s flora, which we aimed to do. The discovery of many new floristic records in the study area is consistent with the results of comprehensive floristic surveys we have conducted elsewhere in the Canadian Arctic (Saarela et al. 2013; Gillespie et al. 2015; Saarela et al. 2017, 2020a, 2020b). The current study and our previous studies demonstrate that we still have much to learn about the Canadian Arctic vascular flora’s diversity and distribution. Further botanical exploration in the study area will likely result in the discovery of additional unrecorded taxa and will contribute to closing gaps in our knowledge of species’ distributions within the study area and the Canadian Arctic.

Our study area included three main subregions: Katannilik Territorial Park, Kimmirut and vicinity (outside the park boundary) and along Pleasant Inlet. We recorded 19 taxa only in Kimmirut and vicinity (Table 3), where four collectors or collector teams have documented them over time. Malte collected two of these taxa (*Cerastiumarcticum* Lange, *Potentillapulchella* R.Br.), Polunin six [BrayaglabellaRichardsonsubsp.glabella, *Coptidiumpallasii* (Schltdl.) Tzvelev, *Drabacrassifolia* Graham, *Equisetumscirpoides* Michx., Saginanodosasubsp.borealis G.E. Crow, Sileneuralensissubsp.arctica (Fr.) Bocquet], Malte and Polunin one (*Huperziaarctica*), Sanson one (*Alopecurusborealis*), Polunin and our team one [*Puccinelliavaginata* (Lange) Fernald & Weath.], Malte and our team one (*Drabaalpina* L.) and our team seven (*Arenarialongipedunculata*, *Cryptogrammastelleri*, Eriophorumscheuchzerisubsp.arcticum M.S.Novos., Festucarubrasubsp.rubra, Hordeumjubatumsubsp.jubatum, *Montiafontana* and *Primulaegaliksensis*). Some or all these taxa may occur within Katannilik Territorial Park, where they have not been recorded. We do not know if the 11 taxa recorded in Kimmirut and vicinity before 2012 and not found by us in 2012, persist there.

We recorded 46 taxa only in Katannilik Territorial Park (Table 3). We collected all but seven of these taxa within the park for the first time in 2012, whereas Aiken first collected Antennariafriesiana(Trautv.)E.Ekmansubsp.friesiana in the park, Soper first collected Chamaenerionangustifolium(L.)Scop.subsp.angustifolium, *Dryopterisfragrans* (L.) Schott, *Ranunculusnivalis* L., *R.pygmaeus* Wahlenb. and *Salixplanifolia* in what is now the park and Johansen first collected *Potentillacrantzii* (Crantz) Beck in what is now the park. We recorded two taxa only from Pleasant Inlet (*Carexsubspathacea* Wormsk. and *Plantagomaritima* L.), where we collected them in 2012. Both species occur on seashores, so they likely do not occur within Katannilik Territorial Park, but they may occur elsewhere in the area.

We recorded 28 taxa in the study area based on a single record. Eight of these are historical collections. Sanson collected one of them, *Alopecurusborealis*, in 1938 and Polunin or Malte collected the rest (Brayaglabellasubsp.glabella, *Cerastiumarcticum*, *Coptidiumpallasii*, *Drabacrassifolia*, *Equisetumscirpoides*, *Potentillapulchella* and Saginanodosasubsp.borealis) in 1936 or earlier. Some of these occurrences are significant regionally. The Saginanodosasubsp.borealis collection is the taxon’s only Baffin Island record. *Equisetumscirpoides* is not recorded elsewhere on Baffin Island, but is recorded from adjacent Dorset Island. *Coptidiumpallasii* is recorded elsewhere on Baffin Island and the Canadian Arctic Archipelago only in Iqaluit. Brayaglabellasubsp.glabella is recorded elsewhere on Baffin Island only in Iqaluit. We do not know if these species still exist within the study area. None of them has been recorded there in 84 or more years.

We collected the remaining 20 taxa known in the study area from a single record in 2012, namely *Andromedapolifolia*, *Arenarialongipedunculata*, *Carexkrausei* Boeckeler, *C.subspathacea*, *Cryptogrammastelleri*, Eriophorum×mediumsubsp.album J.Cay., E.scheuchzerisubsp.arcticum, Festucaproliferavar.lasiolepis Fernald, F.rubrasubsp.arctica (Hack.) Govor., F.rubrasubsp.rubra, Leymusmollissubsp.villosissimus (Scribn.) Á.Löve & D.Löve, L.mollissubsp.mollis, *Montiafontana*, Orthiliasecundasubsp.obtusata, *Oxytropispodocarpa* Gray, *Plantagomaritima*, *Rubuschamaemorus* L., *Sibbaldiaprocumbens* L., *Taraxacumholmenianum* and *Utriculariaochroleuca*. Many of these records are regionally significant. The *Andromedapolifolia* record is the only one from the eastern Canadian Arctic Archipelago. The *Arenarialongipedunculata* record is the only one from the Canadian Arctic Archipelago and Nunavut. The *Cryptogrammastelleri*, Leymusmollissubsp.mollis and Festucaproliferavar.lasiolepis records are the only ones from the Canadian Arctic Archipelago. The F.rubrasubsp.arctica record is the only one from Baffin Island. The *Utriculariaochroleuca* record is the only one from the Canadian Arctic Archipelago and Nunavut.

The study region’s vascular flora includes several species whose south-eastern Canadian Arctic Archipelago distributions are largely restricted to Circumpolar Arctic Bioclimate Subzone D. In the eastern Canadian Arctic, this subzone includes Coats Island, south-western Southampton Island and southern Baffin Island north to Cumberland Sound’s north shore and adjacent islands, but excluding western Foxe Peninsula and adjacent islands (CAVM Team 2003). Within this area, a subset of these species is recorded only from southern Baffin Island: *Astragaluseucosmus* B.L.Rob., *Bartsiaalpina* L., Calamagrostiscanadensissubsp.langsdorffii (Link) Hultén, *Campanularotundifolia* L., *Carexarctogena* Harry Sm., Chamaenerionangustifoliumsubsp.angustifolium, Coptidium×spitsbergense, *C.lapponicum* (L.) Gand, *C.pallasii*, *Drabacrassifolia*, *Eleocharisacicularis* (L.) Roem. & Schult., *Kalmiaprocumbens*, *Luzulaspicata* (L.) DC., *Oreojuncustrifidus* (L.) Záv.Drábk. & Kirschner, *Phyllodocecaerulea* (L.) Bab., *Plantagomaritima*, *Salixuva-ursi* Pursh, *Saxifragapaniculata* Mill. (however, a record Aiken et al. (2007) mapped on northern Baffin Island, based on Porsild and Cody’s (1980) map, requires confirmation), *Sibbaldiaprocumbens*, *Spinulumannotinum* (L.) A.Haines, *Taraxacumlapponicum* Kihlman ex Hand.-Mazz., *Trichophorumcespitosum* (L.) Hartm. and *Viscariaalpina* (L.) G.Don. Another subset of species in this area is recorded from southern Baffin Island and Southampton Island, including *Carexnorvegica* Retz., *C.williamsii* Britton and *Oxytropispodocarpa*.

The study region’s vascular flora also includes species whose distributions in the south-eastern Canadian Arctic Archipelago are restricted to Bioclimate Subzone D and one or more Hudson Strait islands within Bioclimate Subzone C. These species include *Arabisalpina* L. (recorded on southern Baffin Island and Coats, Salisbury and Southampton islands), *Harrimanellahypnoides* (L.) Coville (southern Baffin Island and Coats and Salisbury islands), *Poaalpina* L. (southern Baffin Island and Coats and Southampton islands), *Potentillacrantzii* (southern Baffin Island and Nottingham Island), Saginanodosasubsp.borealis (southern Baffin Island and Southampton Island) and *Salixplanifolia* (southern Baffin Island and Nottingham Island).

Some species recorded in the study area occur elsewhere in the Canadian Arctic Archipelago, primarily within Bioclimate Subzone D in the east and Bioclimate Subzones C and D in the west, on Banks Island, Victoria Island or both. These species include ArtemisiaborealisPallassubsp.borealis, *Betulaglandulosa* Michx., *Carexbicolor* All., *C.lachenalii* Schkuhr, *C.microglochin*, *C.vaginata* Tausch, *Equisetumscirpoides*, Luzulamultiflorasubsp.frigida (Buchenau) V.I.Krecz., *L.wahlenbergii* Rupr., *Montiafontana*, Orthiliasecundasubsp.obtusata, Oxytropisdeflexavar.foliolosa (Hook.) Barneby, *Parnassiakotzebuei* Cham. & Schlecht., *Pedicularislabradorica* Wirsing, *Pinguiculavulgaris*, Potentillaanserinasubsp.groenlandica Tratt. and *Rubuschamaemorus* (Aiken et al. 2007; Gillespie et al. 2015; Saarela et al. 2020b).

Some 50 species known on southern Baffin Island within Bioclimate Subzones D and C have not been recorded within the study area. Many of these species have restricted distributions (based on existing knowledge) on Baffin Island. Several are recorded from one, often historical, locality, including Achilleamillefoliumsubsp.borealis (Bong.) Breitung [Iqaluit], Antennariaalpinasubsp.porsildii (E.Ekman) Chmiel. [Cumberland Peninsula; Chmielewski (1998)], *Deschampsiaalpina* (L.) Roem. & Schult. [Resolution Island], *Diphasiastrumalpinum* (L.) Holub [Hall Peninsula], *Coptistrifolia* (L.) Salisb. [Iqaluit], *Cerastiumarvense* L. [Lower Savage Islands], *Puccinelliapumila* (Macoun ex Vasey) Hitchc. [Iqaluit], *Ranunculusallenii* B.L.Rob. [north shore of Frobisher Bay], *Solidagomultiradiata* Aiton [Iqaluit; requires confirmation] and *Stuckeniafiliformis* (Pers.) Börner [Iqaluit]. Some of these species are recorded from a few localities on Baffin Island, including *Cerastiumcerastoides* (L.) Britton [Beekman Peninsula, Iqaluit, Ogac Lake], *Descurainiasophioides* (Fisch.) Schulz [Iqaluit, Pangnirtung and vicinity], *Euphrasiadisjuncta* Fernald & Wiegand [Hall Peninsula] and *Veronicawormskjoldii* Roem. & Schult. [Ogac Lake, Hall Peninsula] (Aiken et al. 2007). Another subset of these species is recorded from sites on southern Baffin Island west or northwest of the study area, including *Anthoxanthumarcticum* Veldkamp [Dorset Island, Longstaff Bluff, Nettilling Lake], *Askelliapygmaea* (Ledeb.) Sennikov [Dorset Island], *Comarumpalustre* L. [Burwash Bay, Bowman Bay], *Drabamicropetala* Hook. [Amadjuak Lake, Taverner Bay], *D.oblongata* R.Br. ex DC. [Dorset Island, Taverner Bay], *D.subcapitata* Simmons [Taverner Bay], *Eriophorumtriste* (Th.Fr.) Hadač & Á.Löve [Mallik Island], *Festucahyperborea* Holmen ex Fred. [Nettilling Lake], *Myriophyllumsibiricum* Komarov [Bowman Bay, Hantzsch River, Nettilling Lake], *Pediculariscapitata* Adams [Amadjuak vicinity, Bowman Bay vicinity, Nettilling Lake], P.langsdorffiisubsp.arctica (R.Br.) Pennell ex Hultén [Amadjuak vicinity, Bowman Bay vicinity], Poapratensissubsp.colpodea (Th.Fr.) Tzvelev [Foxe Peninsula near Bird [Wildbird] Islands, Nettilling Lake, Taverner Bay] and *Taraxacumphymatocarpum* J.Vahl [Mallik Island, north-western Foxe Peninsula] (Aiken et al. 2007; Saarela et al. 2020a). Some species are more widespread, known from scattered sites across southern Baffin Island, but not recorded within the study area. They include *Cerastiumregelii* Ostenf., *Epilobiumarcticum* Sam., Eriophorumrusseolumsubsp.leiocarpum M.S.Novos., *Festucabaffinensis* Polunin, *Micrantheshirculus* L., *Oxytropisarctobia* Bunge, *Pleuropogonsabinei* R.Br., *Puccinelliaandersonii* Swallen, *Ranunculussulphureus* Sol., *Saginacaespitosa* (J.Vahl) Lange, *S.nivalis* (Lindblom) Fr., *Saxifragarivularis* L., *Silenesorensenis* (B.Boivin) Bocquet, *Stellariacrassifolia* Ehrh. and Tripleurospermummaritimumsubsp.phaeocephalum (Rupr.) Hämet-Ahti (Aiken et al. 2007; Saarela et al. 2020a). Finally, a few species not known in the study area are recorded elsewhere on southern Baffin Island primarily east to northeast of the study area. They include *Euphrasiawettsteinii* G.L.Gusarova, Potentillaarenosasubsp.chamissonis (Hultén) Elven & D.F.Murray, *Rhodiolarosea* L., *Tofieldiacoccinea* Richardson and *Woodsiailvensis* (L.) R.Br. (Aiken et al. 2007). Some of these species recorded elsewhere on southern Baffin Island may occur within the study area, but have not yet been documented.

The Canadian Arctic Archipelago’s known vascular plant flora continues to increase as researchers survey unexplored areas. Gillespie et al. (2015), building on the *Flora of the Canadian Arctic Archipelago* (Aiken et al. 2007), reported 375 species and infraspecific taxa from the Canadian Arctic Archipelago, including several new records from the current study area. Saarela et al. (2020b) added eight taxa newly recorded on Victoria Island to the Canadian Arctic Archipelago flora (Anthoxanthummonticola(Bigelow)Veldkampsubsp.monticola, *Bromuspumpellianus* Scribn., Deschampsiacespitosa(L.)P.Beauv.subsp.cespitosa, *Drabajuvenilis* Kom., *D.pilosa* Adams ex DC., PoapratensisL.subsp.pratensis (not native), SalixovalifoliaTrautv.var.ovalifolia and *Seneciolugens* Richardson) and Saarela et al. (2020a) added one additional species to the flora (*Matricariadiscoidea* DC.; not native). Including the two taxa we here report as new to the Canadian Arctic Archipelago (Festucaproliferavar.lasiolepis, *Luzulagroenlandica* Böcher) brings the number of species and infraspecific vascular plant taxa known in the Canadian Arctic Archipelago to 387. Fifty-seven percent of the Canadian Arctic Archipelago’s vascular flora occurs within the study area, including several taxa not known elsewhere within the Canadian Arctic Archipelago, namely *Arenarialongipedunculata*, Carexbrunnescenssubsp.brunnescens, *Coptidium×spitsbergense*, *Cryptogrammastelleri*, Leymusmollissubsp.mollis, Platantheraobtusatasubsp.obtusata, *Primulaegaliksensis*, *Salixfuscescens*, *Utriculariaochroleuca* and *Triglochinpalustris*.

Including the current study, researchers have characterized vascular plant species diversity in four of Nunavut’s territorial parks. Saarela et al. (2017) recorded 207 taxa in Kugluk Territorial Park in western Nunavut. Saarela et al. (2020b) recorded 57 taxa in Ovayok Territorial Park, near Cambridge Bay on Victoria Island. However, limited exploration has occurred within Ovayok Territorial Park and undocumented vascular plant species likely occur there (Saarela et al. 2020b). Saarela et al. (2020a) recorded 102 taxa in Mallikjuak Territorial Park on Mallik Island, next to Foxe Peninsula. Kugluk occurs within Bioclimate Subzone E, Ovayok and Katannilik within Subzone D and Mallikjuak within Subzone C. Although Katannilik and Ovayok territorial parks both occur in Bioclimate Subzone D, the former’s vascular flora comprises nearly 3.5 times more taxa than the latter’s. The species richness difference between these two parks is predictable, as Katannilik (1,262 km^2^) is nearly 80 times larger than Ovayok (16 km^2^). Furthermore, the former contains considerable habitat diversity, whereas the latter centres on Uvayuq, an esker with less habitat diversity. Similarly, Kugluk (10.5 km^2^) is 120 times smaller than Katannilik Territorial Park. Despite its much smaller area, its vascular species richness is six percent greater than that of Katannilik Territorial Park. We attribute the richer vascular plant diversity in Kugluk Territorial Park compared to Katannilik Territorial Park to three factors. First, Kugluk Territorial Park’s mainland location within the milder Bioclimate Subzone E (vs. Subzone D) favours greater species diversity in local floras. Indeed, the park includes several primarily boreal-distributed species that reach or are near their northern limits in Nunavut within the park, in the Coppermine River valley. Second, Kugluk Territorial Park has considerable habitat diversity despite its small size and third, it is easier to characterize the flora of a small area such as Kugluk Territorial Park, which can be traversed on foot, than a much larger area such as Katannilik Territorial Park. About 1.9 times more species are recorded in Katannilik Territorial Park than in Mallikjuak Territorial Park (ca. 40 km^2^), although knowledge of Mallikjuak's flora is incomplete (Saarela et al. 2020a). We attribute these differences to the bioclimate subzones in which the parks occur, the former within Subzone D and the latter within Subzone C and habitat variation, which is greater in Katannilik than in Mallikjuak Territorial Park. We have also completed botanical inventories of two other parks on Baffin Island: Sylvia Grinnell Territorial Park near Iqaluit (J.M. Saarela et al., unpublished data) and Agguttinni Territorial Park near Clyde Inlet (L.J. Gillespie et al., unpublished data), but comparisons of their floras await synthesis of the results.

## ﻿Annotated checklist

For each taxon, we include synonyms (≡ denotes homotypic synonyms, = denotes heterotypic synonyms), a common name, a summary of the global distribution, voucher information and comments. Codes in square brackets correspond to localities described in Table 1.

### ﻿LYCOPHYTES

**Lycopodiales**

#### Lycopodiaceae

##### *Huperzia* Bernh.

***Huperziaarctica* (Grossh. ex Tolm.) Sipliv.** (≡ H.selagosubsp.arctica (Grossh. ex Tolm). Á.Löve & D.Löve, ≡ Lycopodiumselagosubsp.arcticum Grossh. ex Tolm.)—Arctic fir clubmoss | Circumpolar?

Previously recorded in Kimmirut as *H.selago* (L.) Bernh. ex Schrank & Mart. Widespread across Baffin Island and elsewhere on southern Baffin Island, recorded from Dorset and Mallik islands, Iqaluit, Jackman Sound (*Potter 7407*, GH 02077289) and Resolution Island (Aiken et al. 2007; Saarela et al. 2020a). Some *H.arctica* records, particularly from southern Arctic sites, may be *H.continentalis*; the specimens require review.

**Kimmirut**: *Malte s.n.* [118356] (CAN), *s.n.* [118358] (CAN), *s.n.*/*1198* [121040] (CAN, GH), *Dutilly 1058* (CAN, QFA), *1063* (CAN) [KM-1].

**Figure 7.**
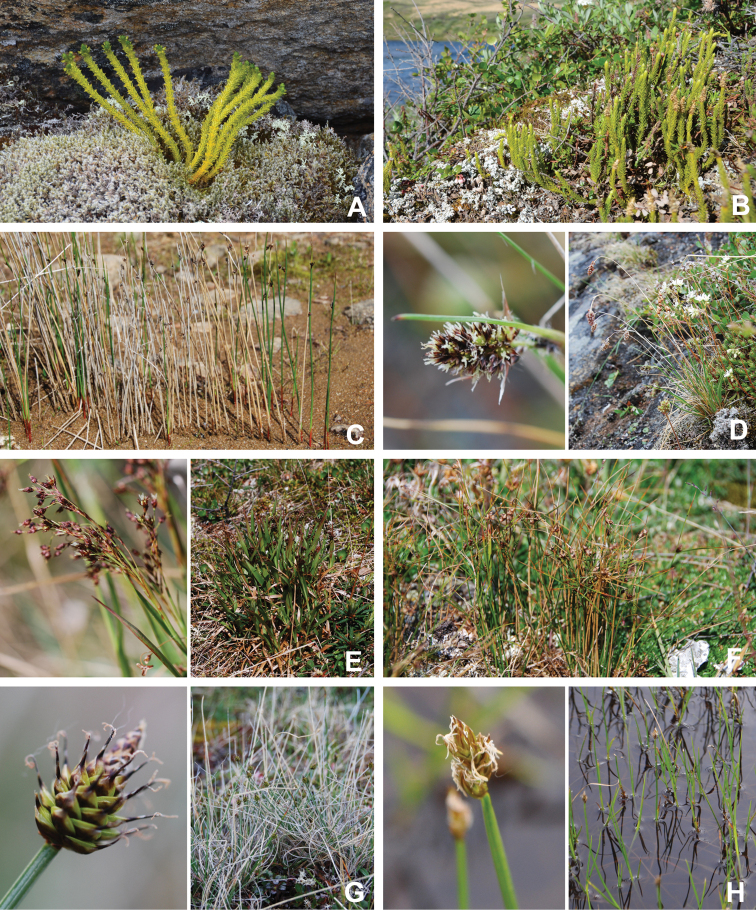
**A***Huperziacontinentalis* habit, *Saarela et al. 2054***B***Spinulumannotinum* habit, *Saarela et al. 2131***C**Juncusarcticussubsp.arcticus habit, *Saarela et al. 2520***D***Luzulaspicata* inflorescence (left) and habit (right), *Saarela et al. 2014***E***Luzulawahlenbergii* inflorescence (left) and habit (right), *Saarela et al. 1926***F***Oreojuncustrifidus* habit, *Saarela et al. 2759***G***Carexarctogena* inflorescence (left) and habit (right), *Saarela et al. 2349***H***Carexchordorrhiza* inflorescence (left) and habit (right), *Saarela et al. 2182*. Photos **A, B** by R.D. Bull, **C–G** by J.M. Saarela and **H** by P.C. Sokoloff.

***Huperziacontinentalis* Testo, A.Haines & A.V.Gilman** (Fig. 7A)—Continental firmoss | Amphi-Beringian–North American

Newly recorded from Kimmirut, the park, Pleasant Inlet and the study area. This recently described species (Testo et al. 2016) differs from *H.arctica* by leaf size, presentation and colour and gemmae size and shape, as described in the key below. *Huperziacontinentalis* occurs throughout north-western and northern North America, including Greenland, and in northeast Asia and northwest Europe. In the Canadian Arctic, Testo et al. (2016) recorded it from the following sites: Northwest Territories: Hornaday River, *Owen & Larsen 74-4011* (DAO) [Saarela et al. (2013) did not review this collection]. Nunavut: Coppermine [Kugluktuk], *Findlay 258* (DAO) [Saarela et al. (2017) did not review this collection]; Canoe Lake, *Peterson 21758* (DAO); Whale Point, *Comer s.n.* (GH); mainland near Depot Island, *Comers s.n.* (GH); Wager Bay area, *Scotter & Zoltai 76140* (DAO); inland north of Wager Bay, *Tremblay 085–2005* (DAO); Baffin Island, Tanner Bay, *Elven 3503/99* (ALA); Baffin Island, Pangnirtung, *Malte s.n.* (GH); Coats Island, *Porsild 5865* (GH). Northern Quebec: Richmond Gulf, mainland south of Cairn Island, *Abbe & Abbe 3171* (GH); Monts de Puvirnituq, *Tremblay 332–11* (DAO). Not all *Huperzia* material from Arctic Canada has been reviewed since the taxon’s description.

**Katannilik Territorial Park**: *Aiken & Iles 02-059* (CAN) [CR-1], *Saarela et al. 1999* (CAN, O) [MJ-20], *2054* (ALA, CAN) [MJ-17], *2318* (CAN, MT) [LR-15]. **Kimmirut**: *Malte s.n.*/*1168* [121010] (CAN, GH), *Oldenburg 115* (MIN) [KM-1]. **Pleasant Inlet**: *Saarela et al. 2722* (CAN) [PI-1].

### ﻿Key to *Huperziaarctica* and *H.continentalis* [adapted from Testo et al. (2016)]

**Table:** 

1	Leaves arched-ascending, 2.5–3.5 × 0.7–1.0 mm in the proximal portion of the shoots, 2.0–2.5 × 0.5–1 mm in the distal portion of the shoots; older shoots mostly stramineous; gemmae 2.4–2.7 × 2.1–2.3 mm, typically symmetrically arched with acute apices	***H.arctica***
–	Leaves generally straight-ascending to appressed, 5–7 × 0.7–1.5 mm in the proximal portion of the shoots, 2.5–5 × 0.7–1.5 mm in the distal portion of the shoots; older shoots orange-yellow in the northern portion of its range; gemmae 3.0–3.5 × 2.9–3.4 mm, typically with unequally arched lateral leaves with obtuse apices	***H.continentalis***

#### *Spinulum* A.Haines

***Spinulumannotinum* (L.) A.Haines** (≡ *Lycopodiumannotinum* L.; = L.annotinumsubsp.alpestre (Hartm.) Á.Löve & D.Löve, = L.annotinumvar.alpestre Hartm.) (Fig. 7B)—Stiff clubmoss | Circumpolar-alpine

Previously recorded in Kimmirut and the park (Polunin 1940; Porsild 1957; Aiken et al. 2007). Elsewhere on Baffin Island, recorded from Beekman Peninsula (*McLaren 166*, CAN 10004078), Cormack Bay, the head of Cumberland Sound (Dutch Polar Station), Iqaluit, Newall Sound (*Wynne-Edwards 7346*, CAN 10004075) and Ogac Lake (*Aiken & LeBlanc 04-027*, CAN 10004070) (Aiken et al. 2007).

**Katannilik Territorial Park**: *Soper s.n.* (CAN, LD) [WR-1], *Saarela et al. 2131* (CAN, GH, MICH, MIN, NFM, QFA, US, UVIC) [CR-13], *2211* (CAN, MO, NYBG) [GC-8], *2413* (ALA, ALTA, CAN, MT, O, UBC, WIN) [LC-3]. **Kimmirut**: *Dutilly 1053* (QFA, 2 ex), *1054* (CAN), *Polunin 1254* (GH), *1771* (GH), *1249* (CAN) [KM-1], *Johansen 1104* (C) [KM-20].

### ﻿MONILOPHYTES

**Equisetales**

#### Equisetaceae

##### *Equisetum* L.

**Equisetumarvensesubsp.alpestre (Wahlenb.) Schönswetter & Elven**—Alpine field horsetail | Circumpolar-alpine

Previously recorded in Kimmirut (Polunin 1940; Porsild 1957; Porsild and Cody 1980; Aiken et al. 2007). Newly recorded in the park. Widespread across Baffin Island and elsewhere on southern Baffin Island, recorded from Amadjuak Bay, between Amadjuak and Chorkbak Bays, Dorset Island and York Sound (*Wynne-Edwards 7336*, CAN 10004593) (Aiken et al. 2007; Saarela et al. 2020a).

**Katannilik Territorial Park**: *Saarela et al. 2031* (ALTA, CAN, UBC) [MJ-19], *2096* (CAN) [MJ-37], *2130* (ALA, CAN, MT, O, WIN) [CR-13], *2218* (CAN, O) [GC-8], *2403* (CAN, MO, US) [LC-3]. **Kimmirut**: *Malte 6* [118353] (CAN), *s.n.* [121013] (CAN), *s.n.* [121035] (CAN) [KM-1], *Saarela et al. 2788* (CAN, NFM, NYBG, UVIC) [KM-13].

***Equisetumscirpoides* Michx.**—Dwarf scouring rush | Circumboreal-polar

Previously recorded in Kimmirut (Polunin 1940; Aiken et al. 2007). Not known in the park. We did not collect this taxon in 2012. Elsewhere on Baffin Island, recorded on Dorset Island (Aiken et al. 2007; Saarela et al. 2020a).

**Kimmirut**: *Polunin 2347* (CAN) [KM-1].

**EquisetumvariegatumSchleich.subsp.variegatum**—Variegated scouring rush | Circumpolar-alpine

Previously recorded in Kimmirut and the park (Polunin 1940; Porsild 1957; Aiken et al. 2007). Widespread across Baffin Island and elsewhere on southern Baffin Island, recorded from Dorset and Mallik islands, Foxe Peninsula near Wildbird Islands, Iqaluit, Lower Savage Islands, Resolution Island and Ukiurjak (formerly King Charles Cape) (*Baldwin 1867*, CAN 10005023) (Aiken et al. 2007; Saarela et al. 2020a).

**Katannilik Territorial Park**: *Aiken & Iles 02-062* (CAN) [SF-2], *Saarela et al. 2262* (CAN) [LR-20], *2298* (CAN) [LR-21], *2373* (CAN, NYBG, UVIC) [LR-11], *2476* (CAN) [EC-15], *2525* (ALA, CAN, O) [SF-24], *2584* (ALA, CAN, MT, O, US, WIN) [SF-21]. **Vicinity of lapis lazuli site**: *Saarela et al. 2496* (CAN, MT, WIN) [LS-3]. **Kimmirut**: *Malte s.n.* [118355] (CAN, V), *s.n.* [121036] (CAN), *Dutilly 1052* (CAN, QFA), *9080* (QFA, 2 ex) [KM-1], *Saarela et al. 2649* (ALTA, CAN, UBC) [KM-8], *2782* (CAN) [KM-19].

**Polypodiales**

#### Cystopteridaceae

##### *Cystopteris* Bernh.

***Cystopterisfragilis* (L.) Bernh.**—Fragile fern | Cosmopolitan

Previously recorded in Kimmirut (Porsild 1957; Porsild and Cody 1980; Aiken et al. 2007). Newly recorded in the park. Widespread across Baffin Island and elsewhere on southern Baffin Island, recorded from Bowdoin Harbour [Schooner Harbour] (*Wynne-Edwards 7190*, CAN 10005429), Dorset Island, Iqaluit, Ogac Lake (*Aiken & LeBlanc 04-215*, CAN 10005316) and Resolution Island (*Wynne-Edwards 7221*, CAN 10005333) (McLaren 1964; Aiken et al. 2007; Saarela et al. 2020a).

**Katannilik Territorial Park**: *Saarela et al. 2083* (CAN) [MJ-33], *2107* (CAN) [MJ-36], *2202* (ALA, CAN, O, WIN), *2203* (CAN) [GC-9], *2277* (CAN, MO, NYBG, UBC, US, UVIC) [LR-25], *2360* (CAN, MIN, QFA) [LR-30], *2626* (ALTA, CAN, MT) [TJ-3]. **Kimmirut**: *Malte s.n.* [120304] (CAN), *s.n.* [126871] (CAN, NY), *Soper s.n.* (CAN), *Polunin 371* (US) [KM-1], *Saarela et al. 2772* (CAN) [KM-19].

#### Dryopteridaceae

##### *Dryopteris* Adans.

***Dryopterisfragrans* (L.) Schott** (≡ *Polypodiumfragrans* L.)—Fragrant wood fern | European (NE)–Asian–Amphi-Beringian–North American (N)

Previously recorded in the park (Porsild and Cody 1980; Aiken et al. 2007). Not known in Kimmirut. The species grew on rocky slopes above the Soper River at Mount Joy. Known from scattered sites across Baffin Island and elsewhere on southern Baffin Island, recorded from Amadjuak Bay, Amadjuak Lake, Iqaluit and Ogac Lake (*Aiken & LeBlanc 04-077*, CAN 10005887) (Aiken et al. 2007). Several sites Aiken et al. (2007) mapped west of Iqaluit, based on Porsild and Cody’s (1980) map, are errors.

**Katannilik Territorial Park**: *Soper s.n.* (CAN, 2 ex; LD) [WR-1], *Saarela et al. 2024* (ALA, CAN, O) [MJ-21].

#### Pteridaceae

##### *Cryptogramma* R.Br.

***Cryptogrammastelleri* (S.G.Gmel.) Prantl**—Steller’s rockbrake | European (NE)–Asian (N/C)–Amphi-Beringian–Cordilleran & North American (NE)

Our collection is the first one of the species, genus and family for the study area, Baffin Island and the Canadian Arctic Archipelago. Gillespie et al. (2015) provide details. Not known in the park. Elsewhere in Nunavut, recorded in Kugluk Territorial Park (Saarela et al. 2017).

**Kimmirut**: *Saarela et al. 2774* (ALA, CAN) [KM-19].

#### Woodsiaceae

##### *Woodsia* R.Br.

***Woodsiaalpina* (Bolton) Gray**—Alpine woodsia | Circumpolar-alpine

Newly recorded in the park and study area. Not recorded in Kimmirut. Elsewhere on Baffin Island, recorded from along Littlecote Channel in Cumberland Sound (*Wynne-Edwards 9338*, CAN 10005123), Iqaluit, Nuvuttiq (formerly Cape Searle) and the vicinity of Tuurngait (formerly Kingnait Harbour) (Polunin 1940; Porsild and Cody 1980).

**Katannilik Territorial Park**: *Saarela et al. 2050* (CAN) [MJ-23], *2204* (ALA, CAN) [GC-9].

***Woodsiaglabella* R.Br.**—Smooth woodsia | Circumpolar-alpine.

Previously recorded in Kimmirut (Polunin 1940; Porsild 1957; Porsild and Cody 1980; Aiken et al. 2007). Newly recorded in the park. Widespread across Baffin Island and elsewhere on southern Baffin Island, recorded from Iqaluit, Kinngait (formerly Cape Dorset) (*Robinson CD_SLR01*, CAN 10041128), Newell Sound (*McLaren 58*, CAN 10005109) and Ogac Lake (Aiken et al. 2007).

**Katannilik Territorial Park**: *Saarela et al. 2287* (ALA, CAN) [LR-22], *2491* (CAN) [EC-13], *2621* (CAN) [TJ-1], *2627* (CAN, MT, WIN) [TJ-3]. **Kimmirut**: *Oldenburg 108B* (MIN), *Polunin 439* (CAN) [KM-1], *Saarela et al. 2773* (CAN, O) [KM-19].

### ﻿MONOCOTS

**Alismatales**

#### Juncaginaceae

##### *Triglochin* L.

***Triglochinpalustris* L.**—Marsh arrowgrass | Circumboreal-polar

Our collections are the first records for Kimmirut, the park, the study area, Baffin Island and the Canadian Arctic Archipelago. Gillespie et al. (2015) provide details. Elsewhere in the Canadian Arctic, known from scattered mainland sites in northern Quebec (Blondeau and Cayouette 2002; Hay 2013), mainland Nunavut and mainland Northwest Territories (Porsild and Cody 1980; Blondeau and Cayouette 2002; Hay 2013; Saarela et al. 2013, 2017).

**Katannilik Territorial Park**: *Saarela et al. 2535* (ALA, CAN, MT) [SF-10]. **Kimmirut**: *Saarela et al. 2652* (CAN, O, WIN) [KM-8].

#### Tofieldiaceae

##### *Tofieldia* Hudson

***Tofieldiapusilla* (Michx.) Pers.** (= *T.borealis* (Wahlenb.) Wahlenb.)—Bog asphodel | Circumpolar-alpine

Previously recorded in Kimmirut (Polunin 1940; Porsild and Cody 1980; Aiken et al. 2007). Newly recorded in the park, where first collected by P. Fleming near the Livingstone River. Recorded at scattered sites on Baffin Island, mostly south of 70°N (Aiken et al. 2007) and elsewhere on southern Baffin Island, recorded from between Amadjuak Bay and Chorkbak Inlet, Amadjuak Bay, Dorset and Mallik islands, Iqaluit and Ogac Lake (*Aiken & LeBlanc 04-075*, CAN 10042148) (McLaren 1964; Aiken et al. 2007; Saarela et al. 2020a).

**Katannilik Territorial Park**: *Fleming 3021* (US) [LR-1], *Saarela et al. 1968* (ALTA, CAN, MO, US) [MJ-8], *2291* (CAN, MT, UBC, WIN) [LR-26]. **Kimmirut**: *Malte s.n.* [120288] (CAN), *s.n.* [120288] (CAN), *s.n.* [118597] (CAN), *s.n.* [118596] (CAN), *s.n.* [118595] (CAN), *Oldenburg 84* (MIN), *Soper s.n.* (CAN), *Dutilly 9101* (QFA), *1063B* (MT), *Polunin 881* (US) [KM-1], *Archambault AA*259 (CAN) [KM-4], *Saarela et al. 2666* (CAN, O) [KM-9], *2744* (ALA, CAN) [KM-12].

**Asparagales**

#### Orchidaceae

##### *Corallorhiza* Gagnebin

***Corallorhizatrifida* Châtel.**—Early coralroot | Circumboreal-polar

Our three collections are the first records for the park and the study area and increase the known Baffin Island records to four; the first record is from Auyuittuq National Park (Gould 1997). Gillespie et al. (2015) provide details. Not known in Kimmirut. Elsewhere in the Canadian Arctic, recorded on Victoria Island and at scattered mainland sites (Porsild and Cody 1980; Saarela et al. 2017).

**Katannilik Territorial Park**: *Saarela et al. 1970* (CAN) [MJ-7], *2036* (CAN) [MJ-24], *2415* (CAN) [EC-19].

##### *Platanthera* Rich.

**Platantheraobtusata(Banks ex Pursh)Lindl.subsp.obtusata** (≡ *Habenariaobtusata* (Banks ex Pursh) Richardson, ≡ *Lysiellaobtusata* (Banks ex Pursh) Rydb.)—Blunt-leaved orchid | North American (N)

Our collections are the first records from the park, the study area, Baffin Island and the Canadian Arctic Archipelago. Gillespie et al. (2015) provide details. Not known in Kimmirut.

**Katannilik Territorial Park**: *Saarela et al. 2209* (CAN) [MJ-29], *2197* (CAN) [GC-7], *2488* (ALA, CAN, O) [EC-18].

**Poales**

#### Juncaceae

##### *Juncus* L.

***Juncusarcticus* Willd. subsp**. ***arcticus*** (Fig. 7C)—Arctic rush | North American (NE)–Amphi-Atlantic–European (N)–Asian (NW)

**Figure 11.**
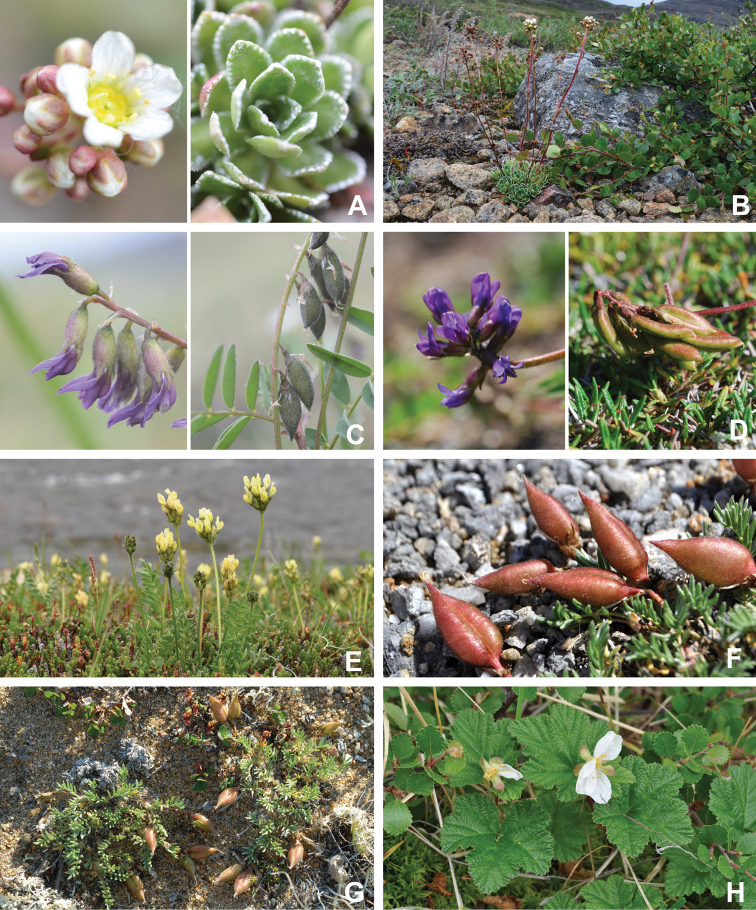
**A***Saxifragapaniculata* inflorescence (left) and basal rosette (right), *Saarela et al. 2240***B***Saxifragapaniculata* habit, *Saarela et al. 2240***C***Astragaluseucosmus* inflorescence (left) and infructescence (right), *Saarela et al. 2302***D**Oxytropisdeflexavar.foliolosa inflorescence (left) and infructescence (right), *Saarela et al. 2530***E***Oxytropismaydelliana* habit, 11 July 2012 **F***Oxytropispodocarpa* infructescence, *Saarela et al. 2541***G***Oxytropispodocarpa* habit, *Saarela et al. 2541***H***Rubuschamaemorus* habit, *Saarela et al. 2304*. Photos **A–C** by R.D. Bull, **D** by J.M. Saarela, **E, G, H** by L.J. Gillespie and **F** by P.C. Sokoloff.

Previously recorded in Kimmirut (Polunin 1940; Porsild and Cody 1980; Aiken et al. 2007). Newly recorded in the park. Known from scattered sites across Baffin Island and elsewhere on southern Baffin Island, recorded from Dorset and Mallik islands and Iqaluit (Aiken et al. 2007; Saarela et al. 2020a). *Juncusarcticus* taxonomy follows Kirschner (2002).

**Katannilik Territorial Park**: *Saarela et al. 2297* (CAN, MO, UBC, WIN) [LR-21], *2397* (ALTA, CAN) [LC-2], *2471* (CAN, NFM, UTC) [EC-3], *2520* (CAN, NYBG, UVIC) [SF-22]. **Kimmirut**: *Malte s.n.* [118559] (CAN), *s.n.* [118558] (CAN, 2 ex), *s.n.* (V) [KM-1], *Saarela et al. 2643* (ALA, CAN, MT, O) [KM-8].

***Juncusbiglumis* L.**—Two-flowered bog rush | Circumpolar-alpine

Previously recorded in Kimmirut (Polunin 1940; Porsild and Cody 1980), but Aiken et al. (2007) did not map it there. Newly recorded in the park. We also collected this species at the lapis lazuli site beyond the park boundary. Widespread across Baffin Island and elsewhere on southern Baffin Island, recorded from Dorset Island, Lower Savage Islands, Ogac Lake, Resolution Island and Ukiurjak (formerly King Charles Cape) (McLaren 1964; Aiken et al. 2007; Saarela et al. 2020a).

**Katannilik Territorial Park**: *Saarela et al. 2136* (CAN) [CR-15]. **Vicinity of lapis lazuli site**: *Saarela et al. 2499* (ALA, CAN, MT, O) [LS-3]. **Kimmirut**: *Polunin 549* (CAN), *754* (US) [KM-1].

***Juncusleucochlamys* V.J.Zinger ex V.I.Krecz.** (≡ J.castaneussubsp.leucochlamys (V.J.Zinger ex V.I.Krecz.) Hultén)—Chestnut rush | Asian (N/C)–Amphi-Beringian–North America (N)–Amphi-Atlantic (W)

Previously recorded in Kimmirut (Polunin 1940; Aiken et al. 2007). Newly recorded in the park. Widespread across Baffin Island and elsewhere on southern Baffin Island, recorded from Dorset Island, Iqaluit, Newell Sound and Ogac Lake (Aiken et al. 2007; Saarela et al. 2020a).

**Katannilik Territorial Park**: *Saarela et al. 2007* (CAN, MT) [MJ-42], *2219* (ALA, ALTA, CAN, O) [WR-3], *2467* (CAN, UBC) [EC-1]. **Kimmirut**: *Malte s.n.* [121039] (CAN) [KM-1], *Archambault AA265*, *AA276* (CAN) [KM-3], *Saarela et al. 2752* (CAN, NYBG, UVIC) [KM-11].

**Juncustriglumissubsp.albescens (Lange) Hultén** (≡ J.triglumisvar.albescens Lange, ≡ *J.albescens* (Lange) Fernald)—Northern white rush | Asian (N)–Amphi-Beringian–North American (N)–Amphi-Atlantic (W).

Previously recorded in Kimmirut and the park (Polunin 1940; Porsild and Cody 1980; Aiken et al. 2007). Known from scattered sites across Baffin Island and elsewhere on southern Baffin Island, recorded from Dorset Island, Iqaluit, Lower Savage Islands and Ogac Lake (Aiken et al. 2007; Saarela et al. 2020a).

**Katannilik Territorial Park**: *Aiken & Iles 02-046* (CAN) [MJ-1], *Saarela et al. 2198* (CAN, UBC) [GC-7], *2380* (CAN, MO) [LR-37], *2466* (ALA, ALTA, CAN) [EC-1], *2506* (CAN, MT, O) [LS-2]. **Kimmirut**: *Malte s.n.* [121034] (CAN), *Dutilly 9117* (QFA), *Polunin 7* (US), *2154* (US) [KM-1].

##### *Luzula* DC.

***Luzulaconfusa* Lindeb.**—Northern woodrush | Circumpolar-alpine

Previously recorded in Kimmirut (Polunin 1940; Porsild and Cody 1980; Aiken et al. 2007). Newly recorded in the park and from Pleasant Inlet. Widespread across Baffin Island and elsewhere on southern Baffin Island, recorded from Dorset and Mallik islands, Iqaluit, Lower Savage Islands and Ogac Lake (Aiken et al. 2007; Saarela et al. 2020a).

**Katannilik Territorial Park**: *Saarela et al. 1928* (CAN, NYBG) [MJ-4], *2172* (CAN, MT) [GC-2], *2176* (CAN) [GC-1], *2285* (CAN, UBC) [LR-22], *2341* (CAN, WIN) [LR-12]. **Kimmirut**: *Malte s.n.* [126860] (CAN), *s.n.* [118592] (CAN), *s.n.* [118593] (CAN), *s.n.* [118583] (CAN) [KM-1]. **Pleasant Inlet**: *Saarela et al. 2693* (ALTA, CAN) [PI-3], *2698* (ASU, CAN, MO, UTC) [PI-2].

***Luzulagroenlandica* Böcher**—Greenland woodrush | North American (N)

Newly recorded for the park, study area, Baffin Island and the Canadian Arctic Archipelago. We collected this species at three sites. At the Soper and Livingstone rivers’ confluence, it grew in a lush meadow with Anthoxanthummonticolasubsp.alpinum, *Arctousalpina*, *Astragalusalpinus*, *Betulaglandulosa*, *Oxytropismaydelliana* and *Pyrolagrandiflora*. At a site between Livingstone River and Emergency Cabin #8, it grew in a grassy meadow surrounded by a *Salixplanifolia* thicket with *B.glandulosa*, *Calamagrostiscanadensis*, *Carexbigelowii*, *Chamaenerionangustifolium* and *Pedicularislapponica*. At Soper Falls, it grew on sandy flats of the lake floodplain with *Agrostismertensii*, *Artemisiaborealis*, *Astragalusalpinus*, *Cerastiumalpinum*, *Festucabrachyphylla*, *Salixarctophila* and *Sileneacaulis*. Elsewhere on Baffin Island, recorded from Beekman Peninsula (*McLaren 128*, CAN 10041965, det. J.M. Saarela, 2018); this collection was previously determined as Luzulamultiflorasubsp.frigida. Elsewhere in the Canadian Arctic, known from scattered sites across mainland Nunavut and northern Quebec and Labrador (Porsild and Cody 1980; Hay and Payette 2013; Saarela et al. 2017).

**Katannilik Territorial Park**: *Saarela et al. 2358* (CAN, MT) [LR-28], *2406* (CAN, O) [LC-3], *2572* (CAN) [SF-17].

**Luzulamultiflorasubsp.frigida (Buchenau) V.I.Krecz.** (≡ *L.frigida* (Buchenau) Sam. ex Lindm.)—Northern many-flowered woodrush | Europe (N), Alaska, Canada, Greenland

Aiken et al. (2007) recorded this taxon in the study area based on an Archambault collection, but we were unable to locate the voucher for confirmation. Our collections confirm its occurrence in the park. It grew in a dried-up pond amongst a dense *Salix* thicket near Group/Warden Cabin #7 with *Bistortavivipara*, *Calamagrostiscanadensis*, *Carexsaxatilis*, *Pyrolagrandiflora* and *Stellarialongipes*. It grew on south-facing, sandy slopes near the Livingstone and Soper rivers’ confluence with *Astragalusalpinus*, *Chamaenerionlatifolium* and *Oxytropismaydelliana*. Elsewhere on Baffin Island, recorded from Beekman Peninsula and Ogac Lake (McLaren 1964; Aiken et al. 2007).

**Katannilik Territorial Park**: *Saarela et al. 2201* (CAN) [GC-5], *2301* (ALA, ALTA, CAN, WIN) [LR-17].

***Luzulanivalis* (Laest.) Spreng.** (= *L.arctica* Blytt)—Arctic wood rush | Circumpolar-alpine

Previously recorded in Kimmirut (Polunin 1940; Porsild and Cody 1980; Aiken et al. 2007). Newly recorded in the park. Widespread across Baffin Island and elsewhere on southern Baffin Island, recorded from Bowdoin Harbour [Schooner Harbour] (*Wynne-Edwards 7182*, CAN 12173), Dorset and Mallik islands, Iqaluit, Lower Savage Islands, Ogac Lake and Resolution Island (McLaren 1964; Aiken et al. 2007; Saarela et al. 2020a).

**Katannilik Territorial Park**: *Saarela et al. 2030* (CAN, QFA) [MJ-6], *2071* (CAN) [MJ-43], *2378* (ASU, CAN, NFM, UTC) [LR-37], *2579* (CAN, MO, MT) [SF-3]. **Kimmirut**: *Polunin 654*, *798*, *2266* (CAN) [KM-1], *Saarela et al. 2751* (CAN, NYBG, WIN) [KM-11].

***Luzulaspicata* (L.) DC.** (Fig. 7D)—Spiked woodrush | Amphi-Atlantic–European & Asian (C) & American Pacific–Cordilleran

Previously recorded in Kimmirut (Aiken et al. 2007). Newly recorded in the park. This species grew in various habitats, including a moist creek bed on a riverbank, on south- and southwest-facing slopes and on the sandy flats of Tasiujarjuaq. Elsewhere on Baffin Island, recorded from Beekman Peninsula, Brewster Point, Iqaluit (head of Tarr Inlet), Newell Sound, Ogac Lake, Pangnirtung and a site on the south side of the Meta Incognito Peninsula (*Scott 26*, ACAD-ECS006361) (Porsild and Cody 1980; Aiken et al. 2007).

**Katannilik Territorial Park**: *Saarela et al. 2014* (CAN) [MJ-42], *2116* (CAN, O) [MJ-39], *2242* (CAN, WIN) [WR-7], *2300* (ALA, ALTA, CAN) [LR-17], *2573* (CAN, MT) [SF-17]. **Kimmirut**: *Polunin 1258* (CAN) [KM-1], *Archambault AA264*, *AA293* (CAN) [KM-3], *Saarela et al. 2746* (CAN, NYBG) [KM-11].

***Luzulawahlenbergii* Rupr.** (≡ L.spadiceavar.wahlenbergii (Rupr.) Buchenau) (Fig. 7E)—Wahlenberg’s woodrush | Circumpolar-alpine

Previously recorded in Kimmirut (Polunin 1940; Porsild and Cody 1980; Aiken et al. 2007). Newly recorded in the park. Elsewhere on Baffin Island, recorded in Iqaluit and Pangnirtung (Aiken et al. 2007) and elsewhere in the Canadian Arctic Archipelago, recorded on Victoria Island (Gillespie et al. 2015; Saarela et al. 2020b).

**Katannilik Territorial Park**: *Saarela et al. 1926* (CAN, GH, MIN, QFA) [MJ-4], *1932* (ALA, ALTA, CAN) [MJ-5], *2108* (ALA, CAN, MT, O) [MJ-32], *2190* (CAN, US) [GC-3], *2474* (CAN, MT, O) [EC-10]. **Kimmirut**: *Polunin 1231* (CAN) [KM-1].

##### *Oreojuncus* Záv.Drábk. & Kirschner

***Oreojuncustrifidus* (L.) Záv.Drábk. & Kirschner** (≡ *Juncustrifidus* L.) (Fig. 7F)—Highland rush | Amphi-Atlantic–European (N)–Asian (NW) & European (C-S) & Asian(C)

Previously recorded in Kimmirut (Polunin 1940; Porsild 1957; Aiken et al. 2007). Not known in the park. We made two collections of this species. At the lapis lazuli site, outside the park boundary, it grew on slopes above a small creek with *Arnicaangustifolia*, *Astragalusalpinus*, *Bartsiaalpina*, *Bistortavivipara*, *Carexbigelowii*, *Equisetumarvense* and *Poaalpina.* At Kimmirut, it grew on south-facing slopes below the garbage dump with *Bistortavivipara*, *Carexscirpoides*, *Luzulaspicata*, *Poaalpina* and *Salixglauca*. Malte collected the species on a “springy slope,” according to label data. Elsewhere on Baffin Island, recorded from Cornelius Grinnell Bay (*Aiken 08-012*, CAN 10041323), Beekman Peninsula, Ogac Lake and York Sound (McLaren 1964; Aiken et al. 2007).

**Kimmirut**: *Malte s.n.* [118571] (CAN), *s.n.* [118570] (ACAD, CAN), *Dutilly 1031* (CAN) [KM-1], *Polunin 882* (US), *Saarela et al. 2759* (ALA, CAN, MT, O) [KM-15]. **Lapis lazuli site**: *Saarela et al. 2501* (CAN, NFM, UTC, UVIC, WTU) [LS-2].

#### Cyperaceae

##### *Carex* L.

***Carexarctogena* Harry Sm.** (≡ C.capitatasubsp.arctogena Böcher) (Fig. 7G)—Tufted black sedge | Amphi-Atlantic

Previously recorded in Kimmirut (Polunin 1940 as *C.capitata* L., Porsild 1957; Aiken et al. 2007). Newly recorded in the park, where we made collections at multiple mesic sites, including grassy meadows and a ravine amongst birch-willow scrub. Associates recorded at two or more of our six sites include *Anthoxanthummonticola*, Calamagrostiscanadensissubsp.langsdorffii, *Carexbigelowii*, *Luzulaconfusa*, *Poaarctica* and *Vacciniumvitis-idaea*. Elsewhere on Baffin Island, known from Beekman Peninsula and the Pangnirtung area (Aiken et al. 2007). Aiken et al. (2007) mistakenly mapped the Pangnirtung area collection (*Blouin*, CAN) at the tip of the Cumberland Peninsula. Not known elsewhere in the Canadian Arctic Archipelago.

**Katannilik Territorial Park**: *Saarela et al. 2118* (CAN, NFM, QFA) [MJ-39], *2171* (ALA, ALTA, CAN, MT, UBC) [GC-2], *2234* (CAN, O) [WR-5], *2377* (CAN, US, UTC, UVIC, WTU) [LR-10], *2349* (CAN, MO) [LR-35], *2445* (CAN, MICH, NYBG, WIN) [EC-8]. **Kimmirut**: *Polunin 32* (US), *1213* (CAN) [KM-1].

**Carexaquatilissubsp.stans (Drejer) Hultén** (= C.aquatilisvar.minor Boott, ≡ *C.stans* Drejer)—Aquatic sedge | Circumpolar-alpine

Polunin (1940) reported this taxon in Lake Harbour and Porsild and Cody (1980) also mapped it there. We are unaware of voucher specimens supporting those records, however. Polunin determined his no. 436 as intermediate between *C.concolor* R.Br. (= C.bigelowiisubsp.bigelowii) and *C.stans*. We assume the 1936 collection Polunin (1940) cited, without number, under *C.aquatilis* Wahlenb. as ‘intermediate’ is this specimen. In 1955, Ernest Lepage determined this specimen as the nothotaxon Carex×nearctica Raymond (C.aquatilissubsp.stans × *C.bigelowii*). We have re-determined this collection as C.aquatilissubsp.stans. Widespread across Baffin Island and elsewhere on southern Baffin Island, recorded from Bowman Bay (Soper’s “Camp Kungovik”), Dorset and Mallik islands, Iqaluit and Silliman’s Fossil Mount (Aiken et al. 2007; Saarela et al. 2020b).

**Kimmirut**: *Polunin 436* (CAN) [KM-1].

***Carexatrofusca* Schkuhr**—Dark brown sedge | Circumpolar-alpine

Previously recorded in Kimmirut (Polunin 1940; Porsild 1957; Porsild and Cody 1980; Aiken et al. 2007). Newly recorded in the park. Widespread on Baffin Island and elsewhere on southern Baffin Island, recorded from Amadjuak Bay, Dorset and Mallik islands, Iqaluit and Ogac Lake (Aiken et al. 2007; Saarela et al. 2020b).

**Katannilik Territorial Park**: *Saarela et al. 2371* (ALTA, CAN, MO, MT, UBC) [LR-11], *2292* (CAN, UVIC, WTU) [LR-26], *2454* (ALA, CAN, O, WIN) [EC-2], *2585* (ASU, CAN, NFM, UTC) [SF-21], *2654* (CAN, MICH, NYBG) [KM-8]. **Kimmirut**: *Dutilly 9122* (US), *Malte s.n.* [120312] (CAN, MICH), *Polunin 293* (US) [KM-1].

***Carexbicolor* All.**—Bicoloured sedge | Circumpolar-alpine

Previously recorded in Kimmirut (Polunin 1940; Porsild 1957; Porsild and Cody 1980; Aiken et al. 2007). According to label data, Malte found the taxon there on a moist, sandy mud flat. Newly recorded in the park. It grew on a sandy mud flat and wet, sandy ground in a dried-up depression with *Dupontiafisheri*, *Eriophorumscheuchzeri*, *Juncusarcticus* and *Leymusmollis*. Elsewhere on Baffin Island, known from Dorset Island and Iqaluit (Aiken et al. 2007; Saarela et al. 2020a).

**Katannilik Territorial Park**: *Saarela et al. 2534* (CAN, MICH, NYBG) [SF-10], *2622* (CAN, WIN) [TJ-3]. **Kimmirut**: *Malte s.n.* [120284] (CAN), *s.n.* [118483] (CAN, MT), *Dutilly 1034a* (US) [KM-1].

**CarexbigelowiiTorr. ex Schwein.subsp.bigelowii** (= *C.concolor* R.Br.)—Bigelow’s sedge | North American–Amphi-Atlantic

Previously recorded in Kimmirut and the park (Polunin 1940; Porsild 1957; Aiken et al. 2007). Widespread on Baffin Island and elsewhere on southern Baffin Island, recorded from Dorset and Mallik islands, Iqaluit, Lower Savage Islands, Ogac Lake, Resolution Island and York Sound (McLaren 1964; Aiken et al. 2007; Saarela et al. 2020a).

**Katannilik Territorial Park**: *Saarela et al. 1936* (CAN, US) [MJ-5], *1959* (CAN, GH, MICH, MIN, NYBG, QFA) [MJ-9], *Aiken & Iles 02-042 b* (CAN) [MJ-1], *Saarela et al. 2256* (CAN, MICH, NYBG, WIN) [GC-10], *2335* (CAN, MIN, QFA) [LR-4], *2450* (ALA, CAN, O) [EC-8], *2465* (ALTA, CAN, MO, MT, UBC) [EC-1]. **Kimmirut**: *Archambault AA*267 (CAN) [KM-3], *Saarela et al. 2650* (CAN, GH) [KM-8], *Dutilly 1034* (US), *Malte s.n.* [120323] (CAN), *s.n.* [120301] (CAN, US), *s.n.* [126877] (CAN), *s.n.* [118522] (MT), *s.n.* [118520] (CAN), *s.n.* [118523] (CAN), *Polunin 290* (MICH), *1098* (F), *1228* (US), *1279* (NY), *1587* (US), *410* (KANU), *492* (KANU), *537* (MIN) [KM-1], *Johansen 1106* (C) [KM-20].

**Carexbrunnescens(Pers.)Poir.subsp.brunnescens**—Brownish sedge | North American–Amphi-Atlantic–European–Asian

Our collections are the first records for the park, the study area, Baffin Island and the Canadian Arctic Archipelago. Gillespie et al. (2015) provide details. Not known in Kimmirut. Elsewhere in the Canadian Arctic, recorded in northern Quebec and northern Labrador (Porsild and Cody 1980; Cayouette 2008) and elsewhere in Nunavut, recorded at a few subarctic sites (Porsild and Cody 1980).

**Katannilik Territorial Park**: *Saarela et al. 2232* (CAN) [WR-5], *2346* (ALA, ALTA, CAN, MO, MT, O, UBC, UVIC, WTU) [LR-35], *2407* (CAN, MICH, NYBG, WIN) [LC-3].

**Carexcapillarissubsp.fuscidula (V.I.Krecz. ex T.V.Egorova) Á.Löve & D.Löve** (≡ *C.fuscidula* V.I.Krecz. ex T.V.Egorova)—Hair sedge | Circumpolar-alpine

Previously recorded in Kimmirut (Polunin 1940; Porsild 1957; Porsild and Cody 1980), but Aiken et al. (2007) did not map it there. The collection *Polunin 356* from Kimmirut, which Polunin originally determined as this species, was later re-determined as *C.williamsii*; however, we agree with Polunin’s original identification. Newly recorded in the park. Widespread, but scattered on Baffin Island and elsewhere on southern Baffin Island, recorded from Dorset Island, Iqaluit and Ogac Lake (Aiken et al. 2007; Saarela et al. 2020a).

**Katannilik Territorial Park**: *Saarela et al. 1996* (CAN, NFM, US, UVIC, WTU) [MJ-12], *2009* (CAN, MT, UBC), *Saarela et al. 2010* (ASU, CAN, MICH) [MJ-42], *2143* (ALA, CAN, NYBG, O, WIN) [CR-11], *2135* (ALTA, CAN, GH, MIN, QFA) [CR-15], *2236* (ALA, ALTA, CAN) [WR-5], *2289* (ALTA, CAN, MO, MT, QFA, UBC) [LR-26], *2344* (CAN, MICH, NYBG, O, WIN) [LR-14], *2456* (CAN, GH, MIN, UVIC, WTU) [EC-2]. **Kimmirut**: *Polunin 356* (CAN), *1219* (US) [KM-1].

***Carexchordorrhiza* L.f.** (Fig. 7H)—Creeping sedge | Circumboreal-polar

Previously recorded in Kimmirut (Polunin 1940; Porsild 1957; Porsild and Cody 1980). Newly recorded in the park. Elsewhere on Baffin Island, known from Iqaluit (Aiken et al. 2007) and several sites on northern Baffin Island: Baffinland Tote Road ~ km 81 (*Bennett et al. 16-0546*, BABY-09721 *n.v.*), *Burt s.n.* (CAN 10037549, CAN 10036537, CAN 10036535, CAN 10040755l), *Tremblay & Pouliot 304-2004* (CAN, QFA) and Isortoq Fiord (*Webber 413*, CAN 10036532).

**Katannilik Territorial Park**: *Saarela et al. 2182* (ALA, ALTA, CAN, O, UBC) [GC-3], *2411* (CAN, GH, MIN, MO, MT, QFA) [LC-3], *2439* (CAN, MICH, NYBG, WIN) [EC-10]. **Kimmirut**: *Polunin 1207* (CAN), *1203* (MTMG), *1208* (US), *1196* (NY) [KM-1].

**Carexfuliginosasubsp.misandra (R.Br.) Nyman** (≡ *C.misandra* R.Br.)—Short leaf sedge | Circumpolar-alpine

Previously recorded in Kimmirut (Polunin 1940; Porsild 1957; Porsild and Cody 1980; Aiken et al. 2007). Newly recorded in the park. Widespread on Baffin Island and elsewhere on southern Baffin Island, recorded from between Amadjuak Bay and Chorkbak Inlet, Dorset and Mallik islands, Foxe Peninsula near Bird [Wildbird] Islands (*Manning 248*, CAN 10037001), Iqaluit, Lower Savage Islands, Ogac Lake (*Aiken & LeBlanc 04-088*, CAN 10036917, *04-049*, CAN 10036875), Perry Bay (*Jotcham s.n.*, CAN 10037089), Resolution Island, Ukiurjak (formerly King Charles Cape) and York Sound (*Walker 805*, US 2311594) (Aiken et al. 2007; Saarela et al. 2020a).

**Katannilik Territorial Park**: *Saarela et al. 2006* (CAN, GH, MICH, MIN, MO, NYBG, QFA) [MJ-42], *2142* (CAN, GH, US, UVIC, WTU) [CR-11], *2293* (ALTA, CAN, MT, UBC) [LR-26], *2458* (ALA, CAN, O, WIN) [EC-2]. **Kimmirut**: *Malte s.n.* [126879] (CAN, H), *s.n.* [118495] (CAN) [KM-1], *Saarela et al. 2739* (ALA, CAN, MICH, NYBG, O, WIN) [KM-12].

***Carexglacialis* Mack.**—Glacier sedge | Circumpolar-alpine

Previously recorded in Kimmirut (Polunin 1940; Porsild and Cody 1980; Aiken et al. 2007). Newly recorded in the park. Known from scattered sites on Baffin Island, including a site on the northern part of the island (71.3776°N, 79.7344°W, *Burt s.n.*, CAN 10037252) and elsewhere on southern Baffin Island, recorded from Beekman Peninsula, Dorset Island, Foxe Peninsula and Iqaluit (Aiken et al. 2007; Saarela et al. 2020a).

**Katannilik Territorial Park**: *Saarela et al. 1980* (CAN, MIN, QFA) [MJ-11], *2283* (ASU, CAN, GH, NFM) [LR-22], *2389* (CAN, UVIC, WTU) [LR-29], *2545* (CAN, MO, MT, UBC, US) [SF-12], *2592* (CAN) [SF-14]. **Kimmirut**: *Malte s.n.* [126890] (CAN, H, NY), *s.n.* [118517] (CAN), *Dutilly 9121* (US), *Polunin 1912* (US) [KM-1], *Saarela et al. 2748* (CAN, MICH, NYBG, WIN) [KM-11].

**Figure 8.**
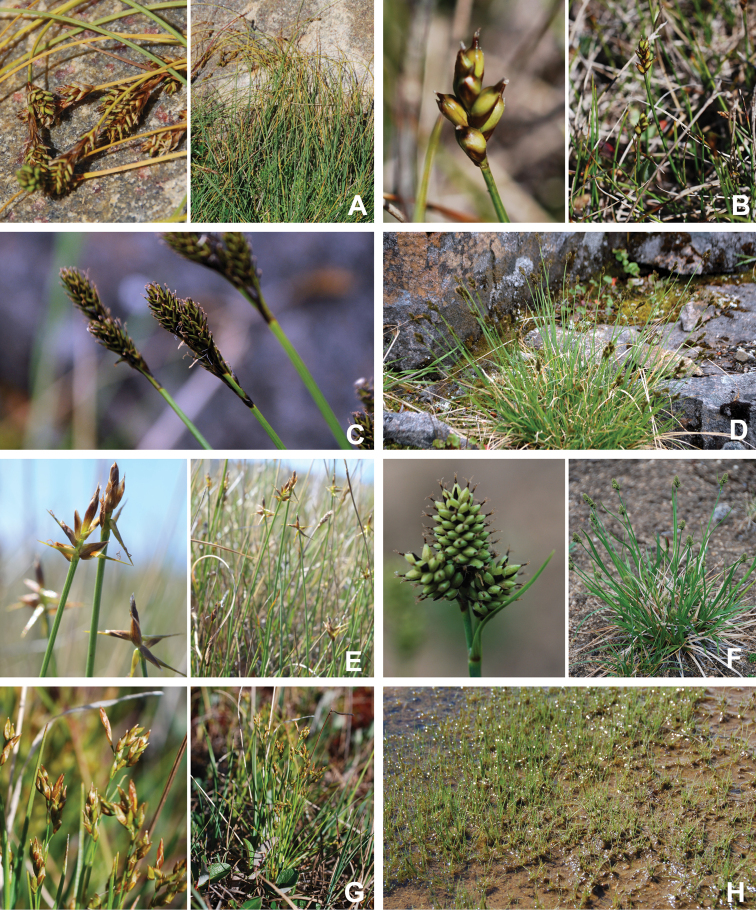
**A**Carexglareosasubsp.glareosa inflorescence (left) and habit (right), *Saarela et al. 2764***B***Carexgynocrates* inflorescence (left) and habit (right), *Saarela et al. 2618***C***Carexlachenalii* inflorescence, *Saarela et al. 2339***D***Carexlachenalii* habit, *Saarela et al. 2339***E***Carexmicroglochin* inflorescence (left) and habit (right), *Saarela et al. 2580***F***Carexnorvegica* inflorescence (left) and habit (right), *Saarela et al. 2001***G***Carexwilliamsii* inflorescences (left) and habit (right), *Saarela et al. 2532***H***Eleocharisacicularis* plants in habitat, *Saarela et al. 2473*. Photos **A,B, F–H** by J.M. Saarela, **C, D** by P.C. Sokoloff and **E** by R.D. Bull.

**CarexglareosaWahlenb.subsp.glareosa** (= C.glareosavar.amphigena Fernald) (Fig. 8A)—Gravel sedge | Circumpolar

Previously recorded in Kimmirut (Polunin 1940; Porsild 1957; Porsild and Cody 1980; Aiken et al. 2007). Newly recorded in the park and from Pleasant Inlet. Known from scattered sites on Baffin Island and elsewhere on southern Baffin Island, recorded from Dorset and Mallik islands, Iqaluit and York Sound (*Walker 804*, US 3157134) (Polunin 1940; Aiken et al. 2007; Saarela et al. 2020a).

**Katannilik Territorial Park**: *Saarela et al. 2615* (CAN, MIN, MO, MT, QFA, UBC) [TJ-5]. **Kimmirut**: *Malte s.n.* [118497] (CAN, V) [KM-1], *Saarela et al. 2764* (ALA, ALTA, CAN, MICH, NYBG, O, WIN) [KM-16]. **Pleasant Inlet**: *Saarela et al. 2690* (CAN, GH, US) [PI-3], *2710* (CAN) [PI-2].

***Carexgynocrates* Wormsk. ex Drejer** (Fig. 8B)—Northern bog sedge | Asian (NE)–Amphi-Beringian–North American (N)

Previously recorded in Kimmirut (Polunin 1940; Porsild 1957; Porsild and Cody 1980; Aiken et al. 2007). Newly recorded in the park, where it grew at the Kimmirut boat landing on Tasiujarjuaq along the wet edge of a creek with *Carexmembranacea*, Juncustriglumissubsp.albescens, *Salixcalcicola* and *Saxifragaaizoides*. Polunin (1940:112) recorded the species as “forming at Lake Harbour a loose turf in damp and muddy, poorly vegetated places—most frequently around freshwater pools whose level recedes in summer.” In Kimmirut, it grew in a sedge meadow along Fundo Lake’s north end with *Carexatrofusca*, C.bigelowiisubsp.bigelowii, *C.membranacea*, *C.microglochin*, *C.rariflora*, *C.simpliciuscula*, *Equisetumvariegatum*, *Eriophorumangustifolium*, *E.callitrix*, *E.scheuchzeri*, *Juncusarcticus*, *Trichophorumcaespitosum* and *Triglochinpalustre*. Not known elsewhere on Baffin Island or in the Canadian Arctic Archipelago. A record Aiken et al. (2007) mapped from Isortoq Fiord (*Webber 413*, CAN 10036532) was misidentified; it is *C.chordorrhiza*.

**Katannilik Territorial Park**: *Saarela et al. 2618* (ALA, ALTA, CAN, MICH, NYBG, O, WIN) [TJ-4]. **Kimmirut**: *Polunin 2341* (CAN), *2336* (US) [KM-1], *Saarela et al. 2657* (CAN) [KM-8].

***Carexholostoma* Drejer**—Arctic marsh sedge | Circumpolar?

Previously recorded in Kimmirut (Polunin 1940; Porsild 1957; Porsild and Cody 1980; Aiken et al. 2007). Newly recorded in the park. Elsewhere on Baffin Island, recorded from Beekman Peninsula, Cumberland Sound, Kingnait [Fiord?] (“Cumberland Gulf, Kingnait,” Taylor in 1860, K, as cited by Polunin 1940; this record should be verified), Iqaluit and Ogac Lake (McLaren, 1964; Porsild and Cody 1980; Aiken et al. 2007).

**Katannilik Territorial Park**: *Saarela et al. 2144* (ALTA, CAN, UBC) [CR-11], *2124* (ALA, CAN, MICH, NYBG, O) [CR-16], *2372* (CAN) [LR-11], *2440* (CAN, MICH) [EC-10]. **Kimmirut**: *Polunin 2341* (CAN), *2283* (US) [KM-1].

***Carexkrausei* Boeckeler** (= C.capillarissubsp.robustior (Lange) Böcher)—Krause’s sedge | Circumpolar-alpine

Newly recorded in the park and study area. This species grew in a sedge meadow around rocky outcrops just south of the campground at Soper Falls with *Astragalusalpinus*, *Bartsiaalpina*, *Carexscirpoidea*, *Dryasintegrifolia*, *Oxytropismaydelliana*, *Salixcalcicola* and *Saxifragaaizoides*. Not recorded in Kimmirut. Elsewhere on Baffin Island, recorded from Auyuittuq National Park (*Ponomarenko Au052*, CAN 10026963), Dorset Island, Iqaluit, Milne Inlet (*Bennett et al. 16-0509*, BABY-09700, *n.v.*, V248453, *n.v.*), a small island along the north shore of Steensby Inlet (*Burt s.n.*, 70.2743°N, 78.5258°W, *Burt s.n.*, CAN 10037565) and two inland sites north of Steensby Inlet (71.3267°N, 79.4408°W, *Burt s.n.*, CAN 10037564; 71.3274°N, 79.4412°W, *Burt s.n.*, CAN 10037563) (Aiken et al. 2007; Saarela et al. 2020a).

**Katannilik Territorial Park**: *Saarela et al. 2586* (CAN, MICH, NYBG) [SF-21].

***Carexlachenalii* Schkuhr** (Fig. 8C, D)—Lachenal’s sedge | Circumpolar-alpine

Previously recorded in Kimmirut (Polunin 1940; Porsild 1957; Porsild and Cody 1980; Aiken et al. 2007). Newly recorded in the park. Elsewhere on Baffin Island, recorded from Cape Searle, Dorset and Mallik islands, Iqaluit, Kivitoo (*Starr 08-246*, CAN 10020884), Maujatuurusiq Inlet and Ogac Lake (McLaren 1964; Aiken et al. 2007; Saarela et al. 2020a).

**Katannilik Territorial Park**: *Saarela et al. 2177* (CAN, GH) [GC-1], *2288* (ALTA, ASU, CAN, US, UTC) [LR-24], *2339* (CAN, MICH, NYBG, WIN) [LR-12], *2334* (CAN) [LR-31], *2435* (CAN, O) [EC-9], *2503* (CAN) [LS-2], *2505* (CAN, MIN, QFA, WTU) [LS-2], *2624* (ALA, ALTA, CAN) [TJ-3]. **Kimmirut**: *Malte s.n.* [120328] (CAN), *s.n.* [118503] (CAN, MICH, US) [KM-1], *Saarela et al. 2655* (ALA, CAN, MO, MT, NFM, UBC) [KM-8].

***Carexmarina* Dewey** (= *C.amblyorhyncha* V.I.Krecz.)—Sea sedge | Circumpolar-alpine

Previously recorded in Kimmirut (Porsild 1957; Porsild and Cody 1980; Aiken et al. 2007). Newly recorded in the park. Known from scattered sites across Baffin Island and elsewhere on southern Baffin Island, recorded from Iqaluit, Lower Savage Islands (*Gillespie et al. 6741*, CAN 585044) and Mallik Island (Aiken et al. 2007; Saarela et al. 2020a).

**Katannilik Territorial Park**: *Saarela et al. 2137* (CAN) [CR-14], *2183* (CAN, NYBG, WIN) [GC-3], *2374* (CAN, MICH) [LR-11], *2461* (ALA, ALTA, CAN) [EC-1], *2495* (CAN, O) [EC-11], *2593* (CAN) [SF-21]. **Kimmirut**: *Malte s.n.* (CAN) [KM-1].

***Carexmaritima* Gunnerus**—Maritime sedge | Circumpolar-alpine

Previously recorded in Kimmirut (Polunin 1940; Porsild 1957; Porsild and Cody 1980; Aiken et al. 2007). Newly recorded in the park and from Pleasant Inlet. Known from scattered sites across Baffin Island and elsewhere on Baffin Island recorded from Dorset and Mallik islands and Iqaluit (Aiken et al. 2007; Saarela et al. 2020a).

**Katannilik Territorial Park**: *Saarela et al. 2369* (CAN, MICH, NYBG) [LR-9], *2332* (ALA, ALTA, CAN, MICH, MO, MT, NYBG, O, UBC, WIN) [LR-29], *2528* (ASU, CAN, US, UTC, UVIC, WTU) [SF-26], *2623* (CAN, GH, MIN, NFM, QFA) [TJ-3]. **Kimmirut**: *Polunin 383* (CAN) [KM-1]. **Pleasant Inlet**: *Saarela et al. 2697* (ALA, CAN, O, WIN) [PI-2].

***Carexmembranacea* Hook.**—Fragile sedge | Amphi-Beringian–North America (N)

Previously recorded in Kimmirut and the park (Porsild and Cody 1980; Aiken et al. 2007). Widespread across Baffin Island and elsewhere on southern Baffin Island, recorded from Amadjuak Bay, Dorset and Mallik islands, Iqaluit, Lower Savage Islands, Ogac Lake, Perry Bay (*Jotcham s.n.*, CAN 10037972, CAN 10037981, CAN 10037980), Pritzler Harbour (*Zika 12150*, MICH 1378483; *Warr 14*ACAD-ECS005624) and Resolution Island (Ford and Ball 1992; Aiken et al. 2007; Saarela et al. 2020a).

**Katannilik Territorial Park**: *Saarela et al. 1925* (ALA, CAN, MO, MT, O, UBC) [MJ-4], *2187* (ASU, CAN, MICH, NFM, NYBG, WTU) [GC-3], *2294* (CAN, MIN, QFA) [LR-26], *2460* (ALA, CAN, MICH, NYBG, O, WIN) [EC-2], *Aiken & Iles 02-049a* (CAN) [CR-1]. **Kimmirut**: *Dutilly 9124* (US), *Malte s.n.* [118533] (US), *s.n.* [126862] (CAN), *s.n.* [126868] (CAN, H, NY), *s.n.* [118531] (CAN), *Oldenburg 80C* (MIN), *Soper s.n.* (CAN, H, NY) [KM-1], *Archambault AA292* (CAN) [KM-3], *2641* (CAN, GH, US, UVIC) [KM-8].

***Carexmicroglochin* Wahlenb.** (Fig. 8E)—Few-seeded fen sedge | American Beringian–North American–Amphi-Atlantic–European (N/C) & Asian (C)

Newly recorded in the park, Kimmirut and study area. Elsewhere on Baffin Island, Aiken et al. (2007) mapped a record near the tip of the Meta Incognito Peninsula, based on Porsild and Cody’s (1980) map. The voucher is probably *Potter 8293* from Brewster Point (62°57'N, 66°03'W), which Polunin (1939) cited. Elsewhere in the Canadian Arctic Archipelago, known from Victoria Island (Saarela et al. 2020b).

**Katannilik Territorial Park**: *Saarela et al. 2376* (CAN, MO, MT, UBC, US) [LR-36], *2580* (CAN, MICH, NYBG) [SF-19]. **Vicinity of lapis lazuli site**: *Saarela et al. 2497* (ALA, ALTA, CAN, O, WIN) [LS-3]. **Kimmirut**: *Saarela et al. 2646* (ALA, ALTA, CAN, MICH, MO, MT, NYBG, O, UBC, WIN) [KM-8].

***Carexmyosuroides* Vill.** (≡ *Kobresiamyosuroides* (Vill.) Fiori)—Mouse-tail bog sedge | Circumpolar-alpine

Previously recorded in Kimmirut (Polunin 1940; Porsild and Cody 1980; Aiken et al. 2007), based on Polunin’s 1936 record, but we are unaware of a voucher specimen. Newly recorded in the park. Widespread across Baffin Island, but elsewhere on southern Baffin Island, recorded only in Iqaluit (Aiken et al. 2007).

**Katannilik Territorial Park**: *Saarela et al. 1976* (CAN, MICH, NYBG) [MJ-11], *2132* (ALA, ALTA, CAN, O, UBC) [CR-15], *2633* (CAN) [TJ-3].

***Carexnardina* Fr.** (= C.nardinavar.atriceps Kuk.)—Nard sedge | Amphi-Beringian–North American–Amphi-Atlantic (W)

Previously recorded in Kimmirut (Polunin 1940; Porsild 1957; Porsild and Cody 1980; Aiken et al. 2007). Newly recorded in the park and Pleasant Inlet. Widespread across Baffin Island and elsewhere on southern Baffin Island, recorded from Dorset and Mallik islands, Iqaluit, Ogac Lake and Resolution Island (*Dutilly 9444*, US-3586019) (Aiken et al. 2007; Saarela et al. 2020a).

**Katannilik Territorial Park**: *Saarela et al. 1979* (ASU, CAN, GH, US, UVIC, WTU) [MJ-11], *2282* (ALA, CAN, MICH, NYBG, O, WIN) [LR-22], *2385* (ALA, ALTA, CAN, MICH, NYBG, O, UBC, WIN) [LR-29], *2589* (CAN, MO, MT, UBC, US, WTU) [SF-14]. **Kimmirut**: *Malte s.n.* [120285] (CAN), *s.n.* [126888] (CAN, NY, UTC), *s.n.* [118509] (CAN), *s.n.* [118510] (CAN), *Dutilly 1024*, *9120* (US) [KM-1]. **Pleasant Inlet**: *Saarela et al. 2694* (ALTA, CAN) [PI-3].

***Carexnorvegica* Retz.** (= C.norvegicasubsp.inserrulata Kalela, = C.norvegicasubsp.conicorostrata Kalela, = *C.vahlii* Schkuhr) (Fig. 8F)—Norway sedge | North American (NE)–Amphi-Atlantic–European (N) & Asian Beringian (or Amphi-Beringian?)

Previously recorded in Kimmirut (Polunin 1940; Porsild and Cody 1980; Aiken et al. 2007). Newly recorded in the park. Elsewhere on Baffin Island, recorded from Beekman Peninsula, Brewster Point (*Potter 8292*, US-2030471), Cumberland Sound, Dorset Island, Iqaluit and Ogac Lake (Aiken et al. 2007; Saarela et al. 2020a). We have not seen vouchers for records Porsild and Cody (1980) and Aiken et al. (2007) mapped from the vicinity of Lower Savage Islands and the head of Cumberland Sound.

**Katannilik Territorial Park**: *Saarela et al. 2008* (CAN, O, WIN) [MJ-42], *2001* (ALA, CAN) [MJ-26], *2199* (CAN, NYBG) [GC-5], *2436* (CAN, MICH) [EC-10], *2469* (ALA, ALTA, CAN, O) [EC-3]. **Kimmirut**: *Malte s.n.* [118480] (CAN) [KM-1].

***Carexrariflora* (Wahlenb.) Sm.**—Loose-flowered alpine sedge | Circumpolar

Previously recorded in Kimmirut (Polunin 1940; Porsild 1957; Porsild and Cody 1980; Aiken et al. 2007). Newly recorded in the park. Elsewhere on Baffin Island, recorded from Beekman Peninsula, Burwash Bay, Dorset and Mallik islands, Iqaluit, Longstaff Bluff, the head of Maktak Fiord (*La Farge 145*, ALTA-VP-52648, *n.v.*), Pangnirtung, Perry Bay (*Jotcham s.n.*, CAN 10038758) and Peter Force Island (Aiken et al. 2007; Saarela et al. 2020a).

**Katannilik Territorial Park**: *Saarela et al. 2032* (CAN, MIN, MO, MT, QFA) [MJ-27], *2109* (ALA, CAN, MICH, NYBG, O, WIN) [MJ-32], *2127* (ALA, ALTA, CAN, O, UBC, WIN) [CR-16], *2438* (CAN, MICH, NYBG, O, UBC, WIN) [EC-10], *2651* (CAN, NFM, US, UTC, UVIC, WTU) [KM-8]. **Kimmirut**: *Malte s.n.* [118519] (CAN), *s.n.* [118518] (CAN) [KM-1], *Johansen 1105* (C) [KM-20].

***Carexrupestris* All.**—Rock sedge | Circumpolar-alpine

Previously recorded in Kimmirut (Polunin 1940; Porsild 1957; Porsild and Cody 1980; Aiken et al. 2007). Newly recorded in the park. Known from scattered sites across Baffin Island and elsewhere on southern Baffin Island, recorded from Amadjuak Lake (*Carroll s.n.*, CAN 10039492), Dorset and Mallik islands, Iqaluit, Lower Savage Islands and Ogac Lake (McLaren 1964; Porsild and Cody 1980; Aiken et al. 2007; Saarela et al. 2020a).

**Katannilik Territorial Park**: *Saarela et al. 2013* (CAN, MT, UBC) [MJ-42], *2065* (ALTA, CAN) [MJ-30], *1977* (CAN, MICH, NYBG, O, WIN) [MJ-11], *2281* (CAN, MO, US, UVIC, WTU) [LR-22], *2590* (ALA, CAN) [SF-14]. **Kimmirut**: *Malte s.n.* [120300] (CAN), *s.n.* [118539], (CAN, MT), *s.n.* [118536] (CAN), *s.n.* [118538] (CAN), *s.n.* [121017] (CAN) [KM-1], *Saarela et al. 2745* (ASU, CAN, NFM, UTC) [KM-11].

***Carexsaxatilis* L.** (= *C.physocarpa* Presl, = C.saxatilissubsp.laxa (Trautv.) Kalela, = C.saxatilisvar.rhomalea Fernald)—Russet sedge | Circumboreal-polar

Previously recorded in Kimmirut (Porsild 1957; Porsild and Cody 1980; Ford and Ball 1992; Aiken et al. 2007). Newly recorded in the park. Widespread across Baffin Island and elsewhere on southern Baffin Island, recorded from Beekman Peninsula, Dorset and Mallik islands, Foxe Peninsula near Wildbird Islands, Iqaluit and Ogac Lake (*McLaren 66*, CAN 10039149) (Aiken et al. 2007; Saarela et al. 2020a).

**Katannilik Territorial Park**: *Saarela et al. 1935* (CAN, MICH, NYBG, WIN) [MJ-5], *2188* (ALA, CAN, O) [GC-3], *2331* (ALTA, CAN, MO, MT, UBC) [LR-29], *2427* (CAN, GH, MIN, QFA) [EC-5]. **Kimmirut**: *Malte s.n.* [121006] (CAN, MT, NY, US), *s.n.* [118540] (CAN), *s.n.* [118533] (CAN), *Polunin 1192* (CAN), *1190* (US), *1226* (F), *1663* (MICH), *1656* (MIN), *1225* (US) [KM-1].

**CarexscirpoideaMichx.subsp.scirpoidea**—Scirpus sedge | Amphi-Beringian–North America (N)–Amphi-Atlantic (W)

Previously recorded in Kimmirut (Polunin 1940; Porsild and Cody 1980; Aiken et al. 2007). Newly recorded in the park. Widespread across Baffin Island and elsewhere on southern Baffin Island, recorded from Cormack Bay, Dorset and Mallik islands, Iqaluit, Ogac Lake (*Consaul et al. 2359c*, CAN 10039217) and York Sound (*Wynne-Edwards 7343*, CAN 10039550) (Aiken et al. 2007; Saarela et al. 2020a).

**Katannilik Territorial Park**: *Saarela et al. 2002* (CAN, MICH) [MJ-26], *2003* (CAN) [MJ-26], *2141* (ALA, CAN, O) [CR-11], *2237* (CAN, NYBG, WIN) [WR-5]. **Kimmirut**: *Malte s.n.* [126889] (CAN, H, NY), *s.n.* [118541] (CAN), *s.n.* [118545] (CAN), *Dutilly 9123* (US), *1018* (MT), *1083a* (US), *Dutilly 9123* (US), *1018* (MT), *1083a* (US) [KM-1], *Oldenburg 80D* (MIN), *Saarela et al. 2656* (ALA, CAN) [KM-8].

**Carexsimpliciusculasubsp.subholarctica (T.V.Egorova) Saarela** (≡ Kobresiasimpliciusculasubsp.subholarctica T.V.Egorova)—Simple bog sedge | Asian (NE)–Amphi-Beringian–North American (N)–Amphi-Atlantic (W)

Previously recorded in Kimmirut (Polunin 1940; Porsild 1957; Aiken et al. 2007). Newly recorded in the park. Known from scattered sites on Baffin Island and elsewhere on southern Baffin Island, recorded from Foxe Peninsula, Iqaluit and Ogac Lake (Aiken et al. 2007). We have not seen a voucher for the Foxe Peninsula site.

**Katannilik Territorial Park**: *Saarela et al. 2375* (ALA, ALTA, CAN) [LR-11], *2295* (CAN, MICH, NYBG, WIN) [LR-26]. **Vicinity of lapis lazuli site**: *Saarela et al. 2498* (CAN, MO, MT, UBC) [LS-3]. **Kimmirut**: *Malte s.n.* [120282] (CAN), *s.n.* [118557] (CAN), *s.n.* [118664], (CAN), *s.n.* [118668] (CAN) [KM-1], *Saarela et al. 2648* (CAN, US) [KM-8].

***Carexsubspathacea* Wormsk.**—Hoppner’s sedge | Circumpolar

Newly recorded from Pleasant Inlet and the study area. This species grew in a saline meadow bordering a small inlet with *Carexursina*, *Puccinelliaphryganodes*, P.tenellasubsp.langeana and *Stellariahumifusa*. Not known in Kimmirut or the park. Widespread across Baffin Island and elsewhere on southern Baffin Island, recorded from Beekman Peninsula, Brewster Point, Dorset and Mallik islands and Iqaluit (Aiken et al. 2007; Saarela et al. 2020a).

**Pleasant Inlet**: *Saarela et al. 2689* (CAN, MICH, NYBG) [PI-3].

**Carexsupinasubsp.spaniocarpa (Steud.) Hultén** (≡ *C.spaniocarpa* Steud., ≡ C.supinavar.spaniocarpa (Steud.) B.Boivin)—Weak arctic sedge | Asian (NE)–Amphi-Beringian–North American (N)

Previously recorded in Kimmirut (Polunin 1940; Porsild 1957; Porsild and Cody 1980; Aiken et al. 2007). Newly recorded in the park. Polunin (1940:72) described the species at Lake Harbour as “growing on rock ledges piled with coarse, crystalline sand unbound by other plants”. At Mount Joy, it grew on dry, rocky upper slopes of a riverbank and a steep, southwest-facing, densely vegetated slope above creeks running into the Soper River. At Willow River, it grew on a rocky river floodplain surrounded by *Salixglauca*–*S.planifolia* willow thicket. At Livingstone River, it grew amongst dense herb growth on a steep, south-facing riverbank slope with a stony-sand substrate. Elsewhere on Baffin Island, recorded from the head of Clyde Inlet, Iqaluit, Pond Inlet and the vicinity of Steensby Inlet (*Burt s.n.*, CAN 10039810) (Aiken et al. 2007). We have not seen a voucher for a record that Aiken et al. (2007) mapped at the tip of the Meta Incognito Peninsula.

**Katannilik Territorial Park**: *Saarela et al. 2011* (ALA, CAN, O) [MJ-42], *1951* (ALTA, CAN, MO, UBC) [MJ-10], *2223* (CAN, MT, UTC) [WR-4], *2368* (CAN, MICH, NYBG, WIN) [LR-9]. **Kimmirut**: *Polunin 2303* (CAN), *2305* (US) [KM-1].

***Carexursina* Dewey**—Bear sedge | Circumpolar

Previously recorded in Kimmirut (Polunin 1940; Aiken et al. 2007). Newly recorded in the park and Pleasant Inlet. Widespread across Baffin Island and elsewhere on southern Baffin Island, recorded from Beekman Peninsula, Dorset and Mallik islands, Iqaluit and Taverner Bay (*Boles et al. RB 99-85*, CAN 10039928) (Porsild and Cody 1980; Aiken et al. 2007; Saarela et al. 2020a).

**Katannilik Territorial Park**: *Saarela et al. 2613* (ALA, ALTA, CAN, O) [TJ-6]. **Kimmirut**: *Polunin 390* (CAN, 2 ex) [KM-1]. **Pleasant Inlet**: *Saarela et al. 2688* (CAN, MICH, NYBG, O, WIN) [PI-3].

***Carexvaginata* Tausch**—Sheathed sedge | Circumboreal-polar

Previously recorded in Kimmirut (Polunin 1940; Porsild 1957; Porsild and Cody 1980; Aiken et al. 2007). Newly recorded in the park. This species grew in a lush peaty meadow along a small stream near the Soper River (Mount Joy), a dried-up pond amongst dense *Salix* thicket (Group/Warden Cabin #7), a sedge meadow (Livingstone River) and mesic tundra in a slight depression grading into a creek (Emergency Cabin #8). Near Kimmirut, it grew on dry slopes with large rock outcrops. Not otherwise known from Baffin Island.

**Katannilik Territorial Park**: *Saarela et al. 2005* (CAN, MIN, QFA) [MJ-18], *2064* (CAN, MO, MT, US) [MJ-30], *2200* (ALA, CAN, O, WIN) [GC-5], *2290* (ALTA, CAN, UBC) [LR-26], *2455* (CAN, MICH, NYBG) [EC-2]. **Kimmirut**: *Malte s.n.* [118548] (CAN, MT), *Polunin 1159* (CAN), *2084* (US) [KM-1], *Saarela et al. 2753* (CAN, WIN) [KM-11].

***Carexwilliamsii* Britton** (Fig. 8G)—Williams’ sedge | Asian (N/C)–Amphi-Beringian–North American (N)

Newly recorded in the park and study area. This species grew in hummocky and turfy sedge meadows. Not known in Kimmirut. One of Polunin’s collections (no. 356) from Lake Harbour, determined by him as *C.capillaris*, was later re-determined as *C.williamsii*; we agree with Polunin’s original identification. Elsewhere on Baffin Island, recorded from Cormack Bay (*Aiken 89-056*, CAN 10039990), Iqaluit and Ogac Lake (Aiken et al. 2007).

**Katannilik Territorial Park**: *Saarela et al. 1998* (CAN, MIN, MO, QFA) [MJ-20], *2068* (CAN, MT, UBC) [MJ-43], *2220* (CAN) [WR-3], *2437* (CAN, MICH, NYBG, WIN) [EC-10], *2532* (ALA, CAN, O, US) [SF-28].

##### *Eleocharis* R.Br.

***Eleocharisacicularis* (L.) Roem. & Schult.** (Fig. 8H)—Needle spikerush | Circumboreal-polar

Previously recorded in Kimmirut (Polunin 1940; Porsild and Cody 1980; Aiken et al. 2007). Newly recorded in the park. This species grew on wet banks at the confluence of a small creek and the Soper River south of Emergency Cabin #8 and on a wet, sandy floodplain at the Soper River’s terminus just southeast of Soper Falls. Associated species at these sites included *Arctophilafulva*, *Carexmaritima*, *C.saxatilis*, *Dupontiafisheri*, *Equisetumarvense*, *Eriophorumscheuchzeri*, *Juncusarcticus* and *Salixarctophila*. Elsewhere on Baffin Island, recorded from the Dewey Soper Migratory Bird Sanctuary (*Dickson et al. s.n.*, CAN 10033574) and Iqaluit (Aiken et al. 2007). Not otherwise known in the Canadian Arctic Archipelago.

**Katannilik Territorial Park**: *Saarela et al. 2473* (CAN, NYBG) [EC-3], *2516* (CAN, MICH) [SF-27]. **Kimmirut**: *Polunin 1341* (US), *1182* (CAN) [KM-1].

##### *Eriophorum* L.

***Eriophorumangustifolium* Honck.**—Narrow-leaved cottongrass | Circumboreal-polar

Previously recorded in Kimmirut (Polunin 1940; Aiken et al. 2007). Newly recorded in the park. Widespread across Baffin Island and elsewhere on southern Baffin Island, recorded from Amadjuak Bay, Dorset Island, Foxe Peninsula, Lower Savage Island, Resolution Island and Silliman’s Fossil Mount (Porsild and Cody 1980; Aiken et al. 2007; Saarela et al. 2020a).

**Katannilik Territorial Park**: *Saarela et al. 2000* (CAN) [MJ-20], *2110* (CAN, MICH) [MJ-32], *2151* (CAN, WIN) [CR-10], *2125* (ALTA, CAN) [CR-16], *2388* (CAN) [LR-29], *2452* (CAN, O) [EC-2], *2462* (CAN) [EC-1]. **Kimmirut**: *Dutilly 9119* (US), *Malte s.n.* [118670] (CAN), *Soper s.n.* (CAN, 2 ex; US) [KM-1], *Johansen 1107* (C) [KM-20], *Saarela et al. 2642* (ALA, CAN, NYBG, WTU) [KM-8].

***Eriophorumcallitrix* Cham.**—Arctic cottongrass | Asian (N)–Amphi-Beringian–North American (N)

Previously recorded in Kimmirut and the park (Polunin 1940; Porsild 1957; Porsild and Cody 1980), but Aiken et al. (2007) did not map these records. Known from scattered sites across Baffin Island and elsewhere on southern Baffin Island, recorded from Dorset Island, Iqaluit, Lower Savage Islands, Ogac Lake, Perry Bay and Silliman’s Fossil Mount (*Jotcham s.n.*, CAN 10033910, CAN 10033911) (Porsild and Cody 1980; Aiken et al. 2007; Saarela et al. 2020a).

**Katannilik Territorial Park**: *Soper s.n.* (CAN) [WR-1], *Saarela et al. 2370* (CAN, MO, US) [LR-11], *2457* (CAN, MT, UBC) [EC-2]. **Kimmirut**: *Saarela et al. 2653* (CAN, MICH) [KM-8], *Malte s.n.* [126887] (CAN, H, UBC), *s.n.* [118683] (CAN), *Soper s.n.* (CAN) [KM-1].

**Eriophorum×mediumsubsp.album J.Cay.**—Intermediate cottongrass | North American (N)

Previously recorded in Kimmirut (Cayouette 2004). Newly recorded in the park, where the species grew in a hummocky, peaty sedge meadow with Arctagrostislatifoliasubsp.latifolia, *Betulaglandulosa*, *Huperzia*, *Carexmembranacea*, *C.norvegica*, *C.rariflora*, *Eriophorumvaginatum*, *Luzulawahlenbergii*, *Salixarctophila* and *Vacciniumvitis-idaea.* The parent species of this nothotaxon are E.russeolumsubsp.leiocarpum and E.scheuchzerisubsp.scheuchzeri. The latter species occurs in the study area, whereas *E.russeolum* has been reported in the study area, but we have not seen a voucher (see Excluded Taxa). Elsewhere on Baffin Island, recorded from Clyde River, the head of Tarr Inlet (Iqaluit) and Nettilling Lake (holotype: *Soper s.n.*, CAN 28144) (Cayouette 2004; Aiken et al. 2007). Elsewhere in the Canadian Arctic, recorded from Chesterfield Inlet, Southampton Island and northern Quebec (Cayouette 2004).

**Katannilik Territorial Park**: *Saarela et al. 2443* (CAN, MICH, NYBG, O, WIN) [EC-10]. **Kimmirut**: *Polunin 1172* (GH, *n.v.*, det. J. Cayouette).

**Eriophorumscheuchzerisubsp.arcticum M.S.Novos.**—Scheuchzer’s cottongrass | Circumpolar

Newly recorded in Kimmirut and the study area. Not known in the park. Elsewhere on southern Baffin Island, recorded from Dorset and Mallik islands, Iqaluit, Foxe Peninsula near “Storm Cove” (*Manning 169*, CAN 10034265; Aiken et al. (2007) incorrectly mapped this record at the mid-point of Foxe Peninsula), Lower Savage Islands (*Gillespie et al. 6708*, CAN 10034315), Resolution Island and Ukiurjak (formerly King Charles Cape) (Aiken et al. 2007; Saarela et al. 2020a). We have not seen Johansen’s collection of *E.scheuchzeri*, at C, from south of the study area (Johansen 1934; Polunin 1940).

**Kimmirut**: *Saarela et al. 2645* (CAN, MICH) [KM-8].

**EriophorumscheuchzeriHoppesubsp.scheuchzeri**—Scheuchzer’s cottongrass | Circumpolar-alpine

Previously recorded from Kimmirut (Aiken et al. 2007), but we could not locate a voucher. Newly recorded in the park. Elsewhere on southern Baffin Island, recorded from between Amadjuak Bay and Chorkbak Inlet, Amadjuak Bay, Iqaluit and Mallik Island (Aiken et al. 2007; Saarela et al. 2020a).

**Katannilik Territorial Park**: *Saarela et al. 2070* (CAN) [MJ-43], *2181* (CAN, NYBG) [GC-3], *2184* (CAN) [GC-3], *2387* (ALA, ALTA, CAN, O) [LR-29], *2463* (CAN) [EC-1], *2575* (CAN) [SF-10]. **Kimmirut**: *Saarela et al. 2644* (CAN, WIN) [KM-8].

**Figure 9.**
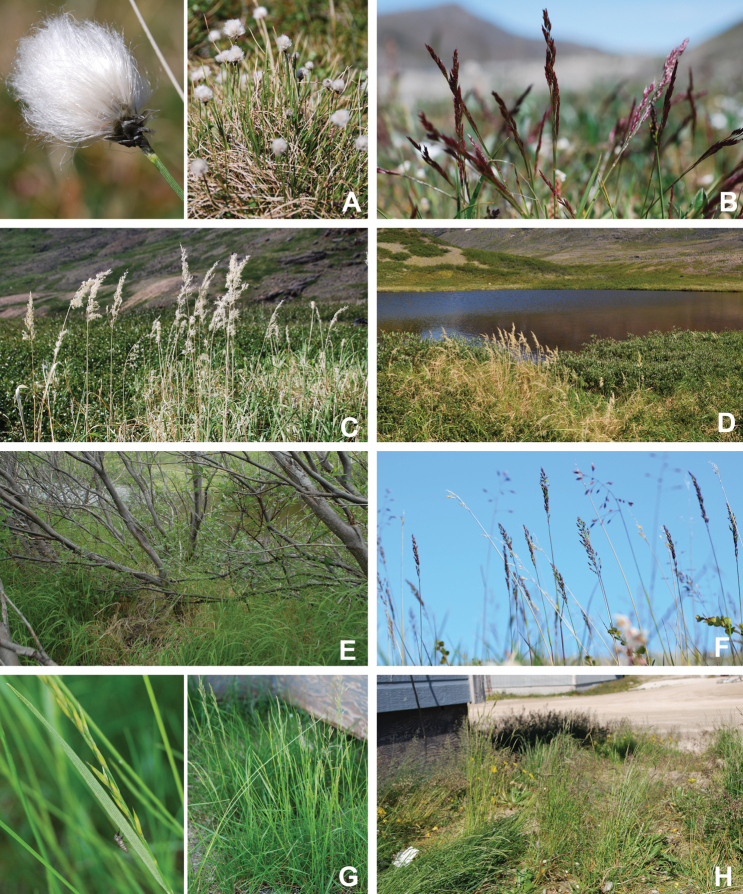
**A**Eriophorumvaginatumsubsp.spissum inflorescence (left) and habit (right), *Saarela et al. 1924***B***Agrostismertensii* inflorescences, *Saarela et al. 2571***C**Calamagrostiscanadensissubsp.langsdorffii habit, *Saarela et al. 1938***D**Calamagrostiscanadensissubsp.langsdorffii habitat, *Saarela et al. 1938***E**Calamagrostiscanadensissubsp.langsdorffii habitat under willows (*Salixplanifolia*), 13 June 2012 **F**Calamagrostisneglectasubsp.groenlandica habit (with *Poa*), *Saarela et al. 2576***G**Festucarubrasubsp.rubra inflorescence (left) and habit (right), *Saarela et al. 2771***H**Festucarubrasubsp.rubra habitat, *Saarela et al. 2771*. Photos **A–C, G, H** by J.M. Saarela, **D** by P.C. Sokoloff, **E** by L.J. Gillespie and **F** by R.D. Bull.

**Eriophorumvaginatumsubsp.spissum (Fernald) Hultén** (≡ *E.spissum* Fernald) (Fig. 9A)—Dense cottongrass | North American (NE)

Previously recorded in Kimmirut and the park (Polunin 1940; Porsild 1957; Porsild and Cody 1980; Aiken et al. 2007). Known from scattered sites across Baffin Island and elsewhere on southern Baffin Island, recorded from Iqaluit and Ogac Lake (Aiken et al. 2007; Saarela et al. 2020a).

**Katannilik Territorial Park**: *Saarela et al. 1924* (CAN, MICH, NYBG) [MJ-4], *2150* (CAN, WIN) [CR-10], *2185* (ALA, CAN, O) [GC-3], *Soper s.n.* (CAN) [WR-1], *s.n.* (CAN) [SF-1]. **Kimmirut**: *Dutilly 1015* (US), *Malte s.n.* [118679] (CAN) [KM-1].

##### *Trichophorum* Pers.

**Trichophorumcespitosum(L.)Hartm.subsp.cespitosum** (≡ *Scirpuscaespitosus* L.)—Tufted clubrush | Circumboreal-polar

Previously recorded in Kimmirut and the park (Polunin 1940; Porsild 1957; Porsild and Cody 1980; Aiken et al. 2007). Elsewhere on Baffin Island, recorded from Beekman Peninsula, Cormack Bay and Ogac Lake (Aiken et al. 2007). We have not validated a record Aiken et al. (2007) mapped from Kinngait (formerly Cape Dorset) (Saarela et al. 2020a).

**Katannilik Territorial Park**: *Aiken & Iles 02-048* (CAN) [CR-1a], *Saarela et al. 2126* (CAN, MO, MT, NFM, US) [CR-16], *2359* (CAN, MICH, NYBG) [LR-28], *2453* (ALA, CAN, O, WIN) [EC-2], *2587* (CAN, MICH) [SF-21]. **Kimmirut**: *Saarela et al. 2647* (ALTA, CAN, UBC) [KM-8], *Malte s.n.* [118684] (CAN), *s.n.* [118685] (CAN, MICH, US), *Polunin 1216* (US) [KM-1].

#### Poaceae

##### *Agrostis* L.

***Agrostismertensii* Trin.** (= *A.borealis* Hartm., = A.mertensiisubsp.borealis (Hartm.) Tzvelev) (Fig. 9B)—Northern bentgrass | Amphi-Pacific–North American (N)–Amphi-Atlantic–European (N)

Previously recorded in Kimmirut (Polunin 1940; Aiken et al. 2007). Newly recorded in the park, where this species grew in sandy flats, in a rocky, dried-up creek bed, in a grassy meadow, amongst mossy turf and in a mesic ravine between birch-willow scrub. Elsewhere on Baffin Island, recorded from Beekman Peninsula, Cormack Bay, Iqaluit and Ogac Lake (Aiken et al. 2007). A Cumberland Sound collection (*Taylor s.n.* in 1861, CAN 10015290 frag. ex K) that Aiken et al. (2007) mapped as this species was misidentified; it is Calamagrostisneglectasubsp.groenlandica. Not otherwise known in the Canadian Arctic Archipelago.

**Katannilik Territorial Park**: *Saarela et al. 2119* (ALTA, CAN, MT, UBC) [MJ-39], *2235* (CAN) [WR-5], *2409* (CAN, O) [LC-3], *2446* (ALA, CAN) [EC-8], *2571* (CAN, UBC, WIN) [SF-17], *2602* (CAN) [TJ-1]. **Kimmirut**: *Malte s.n.* [120302] (CAN, MT, QFA) [KM-1].

##### *Alopecurus* L.

***Alopecurusborealis* Trin.** (= *A.alpinus* Sm., *nom. illeg.*, = *A.magellanicus* Lam. s.l.)—Alpine foxtail | Circumpolar-alpine

Previously recorded in Kimmirut (Polunin 1940; Porsild 1957, 1964; Porsild and Cody 1980; Aiken et al. 2007). Not known in the park. We did not encounter this species in 2012. Widespread across Baffin Island and elsewhere on southern Baffin Island, recorded from Dorset and Mallik islands, Foxe Peninsula near Wildbird Islands (*Baldwin 1862*, CAN 10008406), Iqaluit, and Ukiurjak (formerly King Charles Cape) (Aiken et al. 2007).

**Kimmirut**: *Sanson 24* (TRT) [KM-1].

##### *Anthoxanthum* L.

**Anthoxanthummonticolasubsp.alpinum (Sw. ex Willd.) Soreng** (≡ *Hierochloealpina* (Sw. ex Willd.) Roem. & Schult.)—Alpine sweet grass | Circumpolar-alpine

Previously recorded in Kimmirut and the park (Polunin 1940; Porsild 1957; Porsild and Cody 1980). Widespread across Baffin Island and elsewhere on southern Baffin Island, recorded from Amadjuak Bay, Dorset Island, Iqaluit, Lower Savage Islands, Ukiurjak (formerly King Charles Cape) and York Sound (*Walker 810*, CAN 10012910) (Aiken et al. 2007; Saarela et al. 2020a).

**Katannilik Territorial Park**: *Saarela et al. 1960* (CAN, UBC) [MJ-9], *2087* (CAN) [MJ-33], *2355* (CAN) [LR-28], *2591* (CAN) [SF-14], *Soper s.n.* (CAN, UBC) [WR-1], *s.n.* (CAN, H) [SF-1]. **Kimmirut**: *Malte s.n.* [118380] (CAN, QFA) [KM-1].

##### *Arctagrostis* Griseb.

**Arctagrostislatifolia(R.Br.)Griseb.subsp.latifolia**—Wide-leaved polargrass | Circumpolar-alpine

Previously recorded in Kimmirut (Polunin 1940; Aiken et al. 2007). Newly recorded in the park. Widespread across Baffin Island and elsewhere on southern Baffin Island, recorded from between Amadjuak Bay and Chorkbak Inlet, Amadjuak Bay, Dorset and Mallik islands, Foxe Peninsula near Wildbird Islands, Iqaluit, Ogac Lake, Silliman’s Fossil Mount, near “Storm Cove” and Ukiurjak (formerly King Charles Cape) (Aiken et al. 2007; Saarela et al. 2020a).

**Katannilik Territorial Park**: *Saarela et al.* 1927 (CAN) [MJ-4], *2097* (CAN) [MJ-35], *2379* (CAN) [LR-37], *2468* (CAN, US) [EC-1]. **Kimmirut**: *Malte s.n.* [118828] (CAN), *s.n.* [118827] (CAN), *Dutilly 1014* (QFA, 2 ex; US, 2 ex) [KM-1].

##### *Arctophila* (Rupr.) Rupr. ex Andersson

***Arctophilafulva* (Trin.) Andersson** (≡ *Colpodiumfulvum* (Trin.) Griseb., ≡ *Dupontiafulva* (Trin.) Röser & Tkach)—Pendent grass | Circumpolar-alpine

Previously recorded in Kimmirut (Polunin 1940; Porsild 1957; Porsild and Cody 1980), but we have not seen the voucher *Polunin 1229* that Polunin (1940) cited. Newly recorded in the park. On Baffin Island, recorded as far north as Taverner Bay and elsewhere on southern Baffin Island, recorded from Mallik Island (Aiken et al. 2007; Saarela et al. 2020a). Some authors merge the monotypic sister genera *Arctophila* and *Dupontia*; the latter name has priority (Kellogg 2015; Tkach et al. 2020).

**Katannilik Territorial Park**: *Saarela et al. 2470* (ALTA, CAN, WIN) [EC-3], *2517* (CAN, O, US) [SF-27].

##### *Calamagrostis* Adans.

**Calamagrostiscanadensissubsp.langsdorffii (Link) Hultén** (≡ C.canadensisvar.langsdorffii (Link) Inman) (Fig. 9C–E)—Langsdorff’s reedgrass | Nearly circumboreal-polar

Previously recorded in Kimmirut and the park (Polunin 1940; Porsild 1957; Porsild and Cody 1980; Aiken et al. 2007). At Mount Joy, it grew among dense birch thickets on south-facing slopes, along a pond’s edge with *Betulaglandulosa* and willow and in a sand blowout with *Anthoxanthummonticola* subsp. alpinum. At group/warden cabin #7, it grew in disturbed ground around the shelter. Along the Livingstone River, it grew in a grassy meadow with *Carexarctogena*, *C.bigelowii*, *Bistortavivipara* and *Taraxacumceratophorum*. Elsewhere on Baffin Island, recorded from Pritzler Harbour (*Jotcham s.n.*, QFA0624887; *Warr 9* [2 ex], QFA0210706). A record Aiken et al. (2007) mapped in the Amadjuak Bay area, based on Porsild and Cody’s (1980) map, is an error. The record Porsild and Cody (1980) mapped is likely Polunin’s Kimmirut collection.

**Katannilik Territorial Park**: *Aiken & Iles 02-041* (CAN) [MJ-1], *Saarela et al. 2347* (ALTA, CAN) [LR-35], *2028* (ALA, CAN) [MJ-6], *1938* (CAN, MT, UBC, WIN) [MJ-5], *2113* (CAN, O, US) [MJ-15], *2254* (CAN, MO) [GC-10]. **Kimmirut**: *Polunin 1223* (CAN) [KM-1].

***Calamagrostispurpurascens* R.Br.**—Purple reedgrass | Asian (NE)–Amphi-Beringian–North American–Amphi-Atlantic (W)

Newly recorded in the park and study area. Not known in Kimmirut. This species grew in sparsely vegetated, windswept rocky barrens with *Betulaglandulosa*, *Carexmyosuroides*, *C.nardina*, *C.supina* and *Salixuva-ursi*; on south-facing, sandy slopes with *Anthoxanthummonticola*, *Arctousalpina*, *Empetrumnigrum* and *Saxifragatricuspidata*; in a dried lake bed with *Arabidopsisarenicola*, *Artemisiaborealis*, *Carexmaritima*, *Cerastium*, *Chamaenerionlatifolium*, *Poaglauca* and *Sileneacaulis*; on a dry, rocky slope with *Arctousalpina*, *Chamaenerionlatifolium* and *Saxifragatricuspidata*; and on a rocky river floodplain with *Artemisiaborealis*, *Astragalusalpinus*, *Cerastiumalpinum*, *Chamaenerionlatifolium*, *Potentilla* and *Saxifragatricuspidata*. Known from scattered sites across Baffin Island (Aiken et al. 2007), but not otherwise known from southern Baffin Island.

**Katannilik Territorial Park**: *Saarela et al. 2123* (CAN, O, US) [MJ-31], *2133* (ALA, CAN) [CR-15], *2228* (CAN, NYBG) [WR-4], *2299* (CAN, UBC, WIN) [LR-17], *2383* (CAN, MO, MT) [LR-29].

**Calamagrostisneglectasubsp.groenlandica (Schrank) Matuszk.** (≡ C.neglectavar.groenlandica (Schrank) Druce, ≡ C.strictasubsp.groenlandica (Schrank) Á.Löve) (Fig. 9F)—Narrow-spiked reedgrass | Circumpolar

Our collections are the first records in the park and study area and confirm the taxon’s presence in the eastern Canadian Arctic Archipelago. Gillespie et al. (2015) provide details. Not known in Kimmirut. During this study, we also confirmed records on Baffin Island from Nadluardjuk Lake [68°38'N, 73°05'W] (*Clark s.n.*, DAO 800069) and along the Sylvia Grinnell River, Iqaluit (*Calder 2155*, DAO 106575, DAO 19019, US 04029952). Confusion as to which of the names *C.neglecta* (Ehrh.) G.Gaertn., B.Mey. & Scherb. or *C.stricta* (Timm) Koeler has priority has persisted. In North America, recent authors have recognized the species as *C.stricta* (Marr et al. 2007; Marr et al. 2011; Saarela et al. 2020b) or *C.neglecta* (Aiken et al. 2007). Sennikov (2022) confirmed that *C.neglecta* is the taxon’s correct name.

**Katannilik Territorial Park**: *Saarela et al. 2255* (ALA, CAN) [GC-10], *2191* (CAN, US) [GC-3], *2398* (CAN) [LC-2], *2442* (ALTA, CAN) [EC-10], *2576* (CAN, O) [SF-18].

##### *Deschampsia* P.Beauv.

***Deschampsiasukatschewii* (Popl.) Roshev.** (= *D.pumila* (Griseb.) Ostenf., *illeg. hom.*)—Hairgrass | Circumpolar

Previously recorded in Kimmirut (Porsild 1957; Porsild and Cody 1980), but Aiken et al. (2007) did not map the record. Newly recorded in the park and Pleasant Inlet. Known from scattered sites across Baffin Island and elsewhere on southern Baffin Island, recorded from Dorset Island, Foxe Peninsula and Iqaluit (Porsild and Cody 1980; Aiken et al. 2007; Saarela et al. 2020a). We are unaware of a voucher from the Foxe Peninsula site.

**Katannilik Territorial Park**: *Saarela et al. 2414* (CAN) [EC-20], *2521* (CAN) [SF-22], *2619* (CAN, US) [TJ-4]. **Kimmirut**: *Malte s.n.* [118857] (CAN) [KM-1]. **Pleasant Inlet**: *Saarela et al. 2719* (CAN, US) [PI-1].

##### *Dupontia* R.Br.

***Dupontiafisheri* R.Br.** (= D.fisherisubsp.psilosantha (Rupr.) Hultén)—Fisher’s tundra grass | Circumpolar

Previously recorded in Kimmirut (Polunin 1940; Porsild 1957; Porsild and Cody 1980; Aiken et al. 2007), but we have not seen vouchers for Polunin’s 1936 observations at Lake Harbour that he cited (Polunin 1940). Newly recorded in the park. Widespread across Baffin Island and elsewhere on southern Baffin Island, recorded from Dorset and Mallik islands, Iqaluit, Silliman’s Fossil Mount and “Storm Cove” (Porsild and Cody 1980; Aiken et al. 2007; Saarela et al. 2020a). Some authors merge the monotypic sister genera *Arctophila* and *Dupontia*; the latter name has priority (Kellogg 2015; Tkach et al. 2020).

**Katannilik Territorial Park**: *Saarela et al. 2195* (CAN, US) [GC-6], *2412* (CAN) [LC-3], *2533* (CAN) [SF-10].

##### *Festuca* L.

**FestucabrachyphyllaSchult. & Schult.f.subsp.brachyphylla**—Alpine fescue | Circumpolar-alpine

Previously recorded in Kimmirut (Polunin 1940; Porsild 1957; Porsild and Cody 1980; Aiken et al. 2007). Newly recorded in the park and from Pleasant Inlet. Widespread across Baffin Island and elsewhere on southern Baffin Island, recorded from Chorkbak Inlet, Dorset and Mallik islands, Iqaluit, Lower Savage Islands, Pritzler Harbour (*Warr 1*, QFA0546091), Resolution Island, Silliman’s Fossil Mount and Ukiurjak (formerly King Charles Cape) (Aiken et al. 2007; Saarela et al. 2020a).

**Katannilik Territorial Park**: *Saarela et al. 2015* (CAN, MT, NYBG, US) [MJ-42], *2069* (CAN) [MJ-43], *1961* (CAN, US) [MJ-9], *2122* (CAN) [MJ-39], *2140* (ALA, CAN, O) [CR-8], *2148* (CAN) [CR-9], *2280* (CAN, UBC) [LR-22], *2356* (CAN, MT) [LR-28], *2382* (CAN, UBC, WIN) [LR-29]. **Kimmirut**: *Malte s.n.*/*643* [118374] (CAN, GH, MICH), *s.n.* [120322] (CAN), *s.n.* [118373] (CAN), *s.n.*/*660* [118375] (CAN, GH) [KM-1]. **Pleasant Inlet**: *Saarela et al. 2696* (ALTA, CAN, MT) [PI-2].

**Festucaproliferavar.lasiolepis Fernald**—Pubescent proliferous fescue

Newly recorded in the park, the study area, Baffin Island and the Canadian Arctic Archipelago. This species grew on a small, unnamed island in Tasiujarjuaq amongst an eider duck colony with *Carexscirpoidea*, *Juncusarcticus*, *Salixherbacea*, *S.reticulata* and *Potentillahyparctica.* Not recorded in Kimmirut. Elsewhere in the Canadian Arctic, recorded from mainland Nunavut and northern Quebec and Labrador (Porsild and Cody 1980; Darbyshire and Pavlick 2007).

**Katannilik Territorial Park**: *Saarela et al. 2637* (CAN) [TJ-1].

**Festucarubrasubsp.arctica (Hack.) Govor.** (= *F.richardsonii* Hook.) (Fig. 9G, H) —Arctic red fescue | Circumpolar

Newly recorded for the park, the study area and Baffin Island. Not recorded in Kimmirut. It grew on a small, unnamed island in Tasiujarjuaq amongst an eider duck colony with *Dupontiafisheri*, *Juncusarcticus*, *Leymusmollis*, Potentillaanserinasubsp.groenlandica, Puccinelliaphryganodessubsp.neoarctica and *Saxifragacespitosa*. Known from the adjacent mainland Arctic (northern Quebec) and elsewhere in the Canadian Arctic Archipelago, recorded on Banks and Victoria islands (Aiken et al. 2007; Saarela et al. 2020b).

**Katannilik Territorial Park**: *Saarela et al. 2638* (CAN, US) [TJ-3].

**FestucarubraL.subsp.rubra**—Red fescue | Circumboreal-polar

Newly recorded in Kimmirut and the study area. Not recorded in the park. This species grew around an abandoned house in Kimmirut with *Cerastiumalpinum*, *Poaalpina* and *Taraxacumlapponicum.* It was likely seeded there. Elsewhere on Baffin Island, known from Iqaluit (Aiken et al. 2007) and elsewhere in the Canadian Arctic, known from Cambridge Bay (Victoria Island), Eglinton Island (needs confirmation) and scattered mainland sites (Porsild and Cody 1980; Aiken and Darbyshire 1990; Gould and Walker 1997; Saarela et al. 2017; Saarela et al. 2020b). A record Aiken et al. (2007) mapped south of Clyde River is an error; the collection (*Elven 3553/99*, CAN 10013373) is from Iqaluit.

**Kimmirut**: *Saarela et al. 2771* (ALA, ALTA, CAN, O) [KM-17].

##### *Hordeum* L.

**HordeumjubatumL.subsp.jubatum**—Foxtail barley | Asian (NE) & North American & South American

Newly recorded in Kimmirut and the study area. Kimmirut is the second occurrence area of this non-native species on Baffin Island. It has not been seen in Iqaluit, where previously recorded, since 2003. Gillespie et al. (2015) provide details. Not recorded in the park. Elsewhere in the Canadian Arctic, the species is recorded from scattered mainland sites (Porsild and Cody 1980; Gould and Walker 1997; Saarela et al. 2017); those records are determined as H.jubatumsubsp.intermedium Bowden.

**Kimmirut**: *Saarela et al. 2737* (ALA, ALTA, CAN) [KM-14], *2755* (CAN, O, US) [KM-15].

##### *Koeleria* Pers.

***Koeleriaspicata* (L.) Barberá, A.Quintanar, Soreng & P.M.Peterson** (≡ *Trisetumspicatum* (L.) K.Richt.)—Narrow false-oat | Circumpolar-alpine

Previously recorded in Kimmirut (Polunin 1940; Porsild 1957; Porsild and Cody 1980; Aiken et al. 2007). Newly recorded in the park. Widespread across Baffin Island and elsewhere on southern Baffin Island, recorded from Amadjuak Bay, Dorset and Mallik islands, Lower Savage Islands, Nuwata, Ogac Lake, Perry Bay (*Jotcham s.n.*, CAN 10021591), Resolution Island and York Sound (Aiken et al. 2007; Saarela et al. 2020a). Taxonomy follows Barberá et al. (2019).

**Katannilik Territorial Park**: *Saarela et al. 2022* (CAN, NYBG, UVIC) [MJ-25], *2112* (CAN, O) [MJ-32], *2128* (CAN, WTU) [CR-12], *2230* (ALTA, CAN, MO, UBC) [WR-4], *2340* (CAN, MT) [LR-12], *2632* (ALA, CAN, WIN) [TJ-3]. **Kimmirut**: *Saarela et al. 2743* (CAN, US) [KM-12], *Dutilly 1029* (CAN, US), *9128B* (CAN), *Malte s.n.* [120299] (CAN), *s.n.* [126845] (CAN), *s.n.* [126847] (CAN, H), *s.n.* [118475] (CAN, US), *s.n.* [118476] (CAN, MT), *Polunin 161* (CAN), *561* (CAN), *2346* (US), *Soper s.n.* (CAN, 2 ex; H) [KM-1], *Johansen 1110* (C) [KM-20].

##### *Leymus* Hochst.

**Leymusmollis(Trin.)Pilg.subsp.mollis**—Sea lymegrass | Amphi-Pacific–North American

Newly recorded in the park, the study area and the Canadian Arctic Archipelago. Gillespie et al. (2015) provide details.

**Katannilik Territorial Park**: *Saarela et al. 2529* (CAN, US) [SF-26].

**Leymusmollissubsp.villosissimus (Scribn.) Á.Löve & D.Löve** (≡ Elymusarenariussubsp.villosissimus (Scribn.) Á.Löve)—Arctic lymegrass | Asian (NE)–Amphi-Beringian–North American (N)

Newly recorded in the park and study area. At Tasiujarjuaq, it grew in moist, mossy ground amongst rocky outcrops near the coast with *Dupontiafisheri*, *Juncusarcticus*, Potentillaanserinasubsp.groenlandica, Puccinelliaphryganodessubsp.neoarctica and *Saxifragacespitosa*. Not recorded in Kimmirut. Known from scattered sites across Baffin Island and elsewhere on southern Baffin Island, recorded from Dorset and Mallik islands, Foxe Peninsula near Wildbird Islands, Iqaluit and Pritzler Harbour (*Warr 8*, QFA0186985) (Aiken et al. 2007; Saarela et al. 2020a).

**Katannilik Territorial Park**: *Saarela et al. 2630* (CAN) [TJ-3].

##### *Phippsia* (Trin.) R.Br.

***Phippsiaalgida* (Sol.) R.Br.**—Icegrass | Circumpolar-alpine

Previously recorded in Kimmirut (Polunin 1940; Porsild 1957; Porsild and Cody 1980; Aiken et al. 2007). Newly recorded in the park. Widespread across Baffin Island and elsewhere on southern Baffin Island, recorded from Dorset Island, Iqaluit, Ogac Lake (*Aiken & LeBlanc 04-223*, CAN 586605) and Resolution Island (Aiken et al. 2007; Saarela et al. 2020a).

**Katannilik Territorial Park**: *Saarela et al. 2192* (CAN) [GC-3], *2350* (CAN, O, US) [LR-34], *2519* (CAN) [SF-22], *2540b* (CAN) [SF-25]. **Kimmirut**: *Malte s.n.* [126844] (K, H, UTC), *s.n.* [126898] (CAN), *s.n.* [126900] (CAN) [KM-1], *Saarela et al. 2758* (CAN, O, US) [KM-15].

##### *Poa* L.

**PoaalpinaL.subsp.alpina**—Alpine bluegrass | Amphi-Beringian–North American–Amphi-Atlantic–European–Asian (NW-C)

Previously recorded in Kimmirut (Polunin 1940; Porsild 1957; Porsild and Cody 1980; Aiken et al. 2007). Newly recorded in the park. Known on Baffin Island from as far north as the head of Clyde Inlet, although this record’s identification (*Wynn-Edwards 9080A*, CAN 10015681) is problematic. A record Aiken et al. (2007) mapped south of Clyde Inlet is an error; the collection (*Elven 3554/99*, CAN 10015644) is from Iqaluit. The northernmost confirmed record on Baffin Island is from the Pangnirtung area (Aiken et al. 2007). Elsewhere on southern Baffin Island, recorded from Dorset and Mallik islands, Iqaluit, Ogac Lake, Perry Bay and Silliman’s Fossil Mount (Aiken et al. 2007; Saarela et al. 2020a).

**Katannilik Territorial Park**: *Saarela et al. 2263* (CAN, MT, UBC) [LR-20], *2434* (CAN, NFM, NYBG) [EC-9], *2511* (CAN, WTU) [SF-15], *2738* (ALA, ALTA, CAN, O, US, WIN) [KM-12]. **Kimmirut**: *Dutilly 9127* (CAN), *Polunin 1144* (CAN), *1164* (CAN), *Oldenburg 76A* (MIN, 2 ex), *101* (MIN), *Dutilly 1030a* (QFA, US), *1032* (QFA, 2 ex), *9127* (QFA), *9128* (QFA, 2 ex), *Malte s.n.* [12366] (V), *s.n.* [118403] (CAN, DAO, QFA, US, UTC) [KM-1].

**PoaarcticaR.Br.subsp.arctica**—Arctic bluegrass | Circumpolar-alpine

Previously recorded in Kimmirut (Porsild 1957; Porsild and Cody 1980; Aiken et al. 2007). Newly recorded in the park. Widespread across Baffin Island and elsewhere on southern Baffin Island, recorded from Amadjuak Bay, Dorset and Mallik islands, Iqaluit, Lower Savage Islands, Nuwata, Ogac Lake, Perry Bay, Pritzler Harbour (*Warr 3*, QFA-210705), Resolution Island, Silliman’s Fossil Mount, Ukiurjak (formerly King Charles Cape) and York Sound (*Wynne-Edward 7341*, CAN 10017134) (Aiken et al. 2007; Saarela et al. 2020a).

**Katannilik Territorial Park**: *Saarela et al. 2023* (CAN) [MJ-25], *1929* (CAN) [MJ-4], *2121* (CAN, MT) [MJ-39], *2134* (CAN, UBC, WIN) [CR-15], *2257* (CAN, NYBG) [GC-10], *2441* (CAN, US) [EC-10]. **Kimmirut**: *Dutilly 9128D* (CAN), *Johansen 1109* (C) [KM-20], *Malte s.n.* [118403] (CAN, DAO, QFA, US, UTC), *s.n.* [118404] (CAN), *s.n.* [118432] (CAN, MT, QFA, US), *s.n.* [121030] (CAN), *s.n.* [121007] (CAN), *Blake 1c* (DAO) [KM-2].

**Poaarcticasubsp.caespitans Simmons ex Nannf.**—High Arctic bluegrass | North American (NE)–Amphi-Atlantic–European (N)

Previously recorded in Kimmirut (Polunin 1940; Porsild 1957; Porsild and Cody 1980; Aiken et al. 2007). Newly recorded in the park. Widespread across Baffin Island and elsewhere on southern Baffin Island, recorded from Dorset Island, Foxe Peninsula near “Storm Cove” [*Manning 172*, CAN 10017611; Aiken et al. (2007) mistakenly mapped this record on the middle of Foxe Peninsula], Iqaluit and Ogac Lake (Aiken et al. 2007; Saarela et al. 2020a).

**Katannilik Territorial Park**: *Saarela et al. 2386* (CAN, US) [LR-29]. **Kimmirut**: *Malte s.n.* [118431] (CAN, 2 ex), *Soper s.n.* (CAN) [KM-1], *Saarela et al. 2741* (CAN, O, US) [KM-12].

**PoaglaucaVahlsubsp.glauca**—Glaucus bluegrass | Circumpolar-alpine

Previously recorded in Kimmirut (Polunin 1940; Porsild 1957; Porsild and Cody 1980; Aiken et al. 2007). Newly recorded in the park. Widespread across Baffin Island and elsewhere on southern Baffin Island, recorded from Dorset Island, Iqaluit, Ogac Lake and Taverner Bay (*Manning 4*, CAN 10015916) (Aiken et al. 2007; Saarela et al. 2020a).

**Katannilik Territorial Park**: *Saarela et al. 1978* (CAN) [MJ-11], *1994* (CAN) [MJ-41], *2284* (ALA, CAN) [LR-22], *2384* (CAN, O, US) [LR-29], *2588* (ALTA, CAN) [SF-14]. **Kimmirut**: *Dutilly 990a* (CAN), *9126D* (CAN, QFA), *1030A*, *9128C* (QFA), *Malte s.n.* [126846] (ALTA-VP, CAN), *s.n.* [126848] (CAN, H, NY, UTC), *s.n.* [121015] (CAN, MT, US), *Oldenburg 76B* (MIN), *Polunin 381* (CAN), *Soper s.n.* (H) [KM-1], *Saarela et al. 2742* (CAN, MT, UBC, WIN) [KM-12].

**Poapratensissubsp.alpigena (Lindm.) Hiitonen** (≡ *P.alpigena* Lindm.)—Northern meadow-grass | Circumboreal-polar

Previously recorded in Kimmirut, but we have not seen vouchers that support Polunin’s 1934 and 1936 records (Polunin 1940). Our collections confirm the taxon’s presence in the Kimmirut area. Newly recorded in the park. Widespread across Baffin Island and elsewhere on southern Baffin Island, recorded from Amadjuak Bay, Dorset and Mallik islands, Iqaluit and Pritzler Harbour (*Warr 3*, QFA-153929) (Aiken et al. 2007; Saarela et al. 2020a).

**Katannilik Territorial Park**: *Saarela et al. 1950* (CAN, US) [MJ-45], *2111* (CAN, MO) [MJ-32], *2233* (CAN, O) [WR-5], *2574* (CAN) [SF-17]. **Kimmirut**: *Saarela et al. 2793* (CAN, MO, MT, NYBG) [KM-6], *2740* (ALA, CAN) [KM-12], *2757* (CAN, US) [KM-15], *2762* (ALA, ALTA, CAN, UBC, WIN) [KM-16].

##### *Puccinellia* Parl.

**Puccinelliaphryganodessubsp.neoarctica (Á.Löve & D.Löve) Elven**—Goosegrass | North American (N)

Previously recorded in Kimmirut (Polunin 1940; Porsild 1957; Porsild and Cody 1980; Aiken et al. 2007). Newly recorded in the park and Pleasant Inlet. Widespread across Baffin Island and elsewhere on southern Baffin Island, recorded from Dorset and Mallik islands, Iqaluit, Lower Savage Islands, Ogac Lake (*McLaren 75*, CAN 10019927), Resolution Island and the Silliman’s Fossil Mount area (Aiken et al. 2007; Saarela et al. 2020a). **Katannilik Territorial Park**: *Saarela et al. 2631* (CAN) [TJ-3], *2614* (ALA, CAN, O) [TJ-6]. **Kimmirut**: *Malte s.n.* [118444] (CAN) [KM-1], *Saarela et al. 2766* (ALTA, CAN, UBC, WIN) [KM-16]. **Pleasant Inlet**: *Saarela et al. 2706* (CAN, MT) [PI-2], *2692* (CAN, MO, US, WIN) [PI-3].

**Puccinelliatenellasubsp.langeana (Berlin) Tzvelev** (≡ *P.langeana* Berlin)—Lange’s alkaligrass | Amphi-Beringian?–North American (N)

Previously recorded in Kimmirut (Polunin 1940; Porsild 1957; Porsild and Cody 1980; Aiken et al. 2007). Newly recorded in the park and from Pleasant Inlet. Widespread across Baffin Island and elsewhere on southern Baffin Island, recorded from Dorset Island, Iqaluit, Lower Savage Islands and Ogac Lake (Aiken et al. 2007; Saarela et al. 2020a).

**Katannilik Territorial Park**: *Saarela et al. 2538* (CAN, MICH, MT, O) [SF-25], *2540a* (CAN) [SF-25], *2610* (CAN, UBC) [TJ-6], *2616* (CAN, MO, US, WIN) [TJ-5]. **Kimmirut**: *Malte s.n.* [120326] (CAN) [KM-1]. **Pleasant Inlet**: *Saarela et al. 2691* (ALA, ALTA, CAN, WTU) [PI-3], *2711* (CAN, NYBG, UTC) [PI-2].

***Puccinelliavaginata* (Lange) Fernald & Weath.**—Tussock alkaligrass | Amphi-Beringian–North American (N)

Newly recorded in Kimmirut and the study area. Not known in the park. Polunin’s collection from Lake Harbour (no. 1163) was originally identified as *P.angustata* (R.Br.) E.L.Rand & Redfield. Another Polunin collection from Lake Harbour identified as *P.angustata* (*Polunin 701*, F image! MICH image!) is likely *P.vaginata*, but the specimens need to be examined for confirmation. Below the Kimmirut garbage dump, it grew along the coast above the high tide line in sewage-enriched ground with *Carexbicolor*, *Koenigiaislandica*, Potentillaanserinasubsp.groenlandica, Puccinelliaphryganodessubsp.neoarctica and P.tenellasubsp.langeana. Elsewhere on Baffin Island, recorded from Iqaluit (e.g., *Gillespie 6279*, CAN 10020101; *Saarela et al.* 2794, CAN 10020100) and scattered sites along the east coast (Aiken et al. 2007).

**Kimmirut**: *Polunin 1163* (CAN) [KM-1], *Saarela et al. 2768* (ALA, ALTA, CAN, O, US) [KM-16].

### ﻿EUDICOTS

**Ranunculales**

#### Papaveraceae

##### *Papaver* L.

The taxonomy of Papaversect.Meconella Spach, to which all Arctic species belong, is challenging (Elven et al. 2009; Elven et al. 2011). We accept the taxonomy Solstad (2009) proposed for *Papaver* in the Canadian Arctic; see also Aiken et al. (2007) and Elven et al. (2011). We identified *Papaver* material using an unpublished key (H. Solstad and R. Elven, pers. comm.). Distribution maps for the multiple *Papaver* taxa now recognized across the Canadian Arctic Archipelago and the Arctic mainland are unavailable. Maps for several taxa as now understood, however, exist for smaller areas, including northern Quebec and Labrador (Payette 2013, 2015, 2018) and Victoria Island (*P.cornwallisense* D.Löve, *P.dahlianum* Nordh., *P.hultenii* Knaben, *P.lapponicum*) (Saarela et al. 2020b).

***Papaverlabradoricum* (Fedde) Solstad & Elven** (≡ P.nudicaulevar.labradoricum Fedde, ≡ P.radicatumsubsp.labradoricum (Fedde) Fedde, ≡ P.lapponicumsubsp.labradoricum (Fedde) Knaben)—Labrador poppy | North American (NE)

Previously recorded in Kimmirut (Polunin 1940; Porsild 1957, 1964; Porsild and Cody 1980). Newly recorded in the park. Researchers previously recorded poppies in the study area as *P.radicatum* L. (Polunin 1940; Porsild 1957, 1964; Porsild and Cody 1980). Aiken et al. (2007) did not map any poppy records in the study area under *Papaver* spp., the name she used for all Canadian Arctic Archipelago poppies, except the Amphi-Beringian *P.keelei* A.E.Porsild, from Banks Island. Based on revised specimens at CAN, *P.labradoricum* is recorded in the Canadian Arctic from across Baffin Island, Belcher Islands, Lower Savage Islands, Big, Coats, Dorset (Saarela et al. 2020a), Mill, Resolution, Salisbury and Southampton Islands and the Nunavut mainland to just west of Aberdeen Lake along the Thelon River. Elven et al. (2011) report the taxon’s distribution as “ … known from eastern Canada, southern and western Greenland and from one alpine locality (CAN) in eastern Greenland”.

**Katannilik Territorial Park**: *Saarela et al. 1986* (ALA, CAN, O, TRH) [MJ-40], *2101* (CAN) [MJ-36], *2208* (CAN) [GC-9], *2244* (CAN, O, TRH) [WR-10], *2366* (CAN, O, TRH) [LR-9]. **Kimmirut**: *Malte s.n.* [121011] (CAN)), *Oldenburg 103* (MIN), *Soper s.n.* (CAN) [KM-1].

***Papaverlapponicum* (Tolm.) Nordh.**—Lapland poppy | North American (N)–Amphi-Atlantic–European (N)–Asian (N)

Previously recorded in Kimmirut. Newly recorded in the park. Following Elven et al. (2011), plants in the study area correspond to P.lapponicumsubsp.occidentale (C.E.Lundstr.) Knaben, the only subspecies they recorded in the Canadian Arctic.

**Katannilik Territorial Park**: *Saarela et al. 2056* (CAN) [MJ-16]. **Kimmirut**: *Polunin 403* (GH) [KM-1].

**Saxifragales**

#### Ranunculaceae

##### *Coptidium* (Prantl) Rydb.

**Figure 10.**
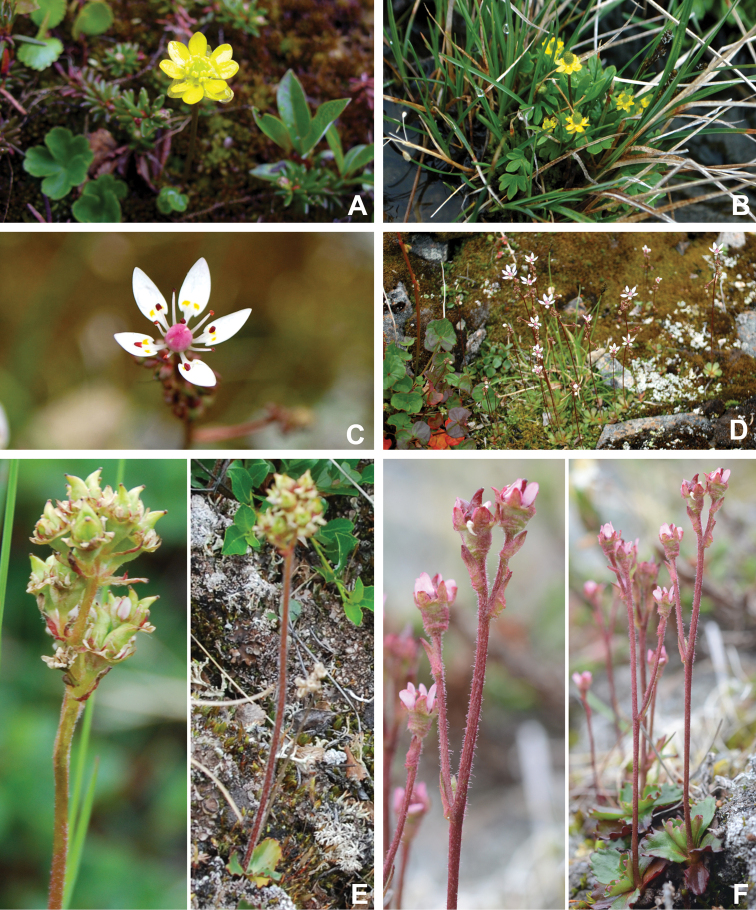
**A***Coptidiumlapponicum* inflorescence, *Saarela et al. 2094***B***Ranunculuspygmaeus* habit, *Saarela et al. 2342***C***Micranthesfoliolosa* inflorescence, *Saarela et al. 2338***D***Micranthesfoliolosa* habit, *Saarela et al. 2338***E***Micranthesnivalis* inflorescence (left) and habit (right), *Saarela et al. 2017***F***Micranthestenuis* inflorescence (left) and habit (right), *Saarela et al. 2308*. Photos **A–D** by P.C. Sokoloff, **E** by J.M. Saarela and **F** by R.D. Bull.

***Coptidiumlapponicum* (L.) Gand.** (≡ *Ranunculuslapponicus* L.) (Fig. 10A, B)—Lapland buttercup | Circumboreal-polar

Previously recorded in Kimmirut (Polunin 1940; Porsild and Cody 1980; Aiken et al. 2007). Newly recorded in the park. Elsewhere on Baffin Island, recorded from Iqaluit, Nettilling Lake and Pangnirtung (Porsild and Cody 1980; Aiken et al. 2007).

**Katannilik Territorial Park**: *Saarela et al. 2029* (CAN, MT) [MJ-6], *2061* (CAN, MO, UBC, US) [MJ-28], *2094* (ALA, CAN) [MJ-37], *Soper s.n.* (CAN) [WR-1], *Saarela et al. 2396* (ALTA, CAN) [LC-2], *2492* (CAN, O, WIN) [EC-12]. **Kimmirut**: *Johansen 1120* (C) [KM-20].

***Coptidiumpallasii* (Schltdl.) Tzvelev** (≡ *Ranunculuspallasii* Schltdl.)—Pallas’ buttercup | European (N)–Asian (N)–Amphi-Beringian–North American (N)

Previously recorded in Kimmirut by Polunin in 1936, whose “collection was growing 10–20 cm high on wet mud by the margin of a freshwater pool” in the Lake Harbour vicinity (Polunin 1940:211). We did not encounter this species in 2012. Elsewhere on Baffin Island, recorded from Iqaluit (Porsild and Cody 1980; Aiken et al. 2007) and not otherwise known in the Canadian Arctic Archipelago.

**Kimmirut**: *Polunin 1173* (CAN) [KM-1].

***Coptidium×spitsbergense* (Hadač) Elven**—Spitzbergen’s buttercup | Circumpolar

Our collections of this sterile triploid hybrid (*C.lapponicum* × *C.pallasii*) are the first records for the park, the study area, Baffin Island and the Canadian Arctic Archipelago. Gillespie et al. (2015) provide details.

**Katannilik Territorial Park**: *Saarela et al. 2194* (ALA, CAN, MT, O, WIN) [GC-6], *2419* (ALA, CAN, O) [EC-7].

##### *Ranunculus* L.

***Ranunculusarcticus* Richardson** (= R.pedatifidusvar.affinis (R.Br.) L.D.Benson, = R.pedatifidusvar.leiocarpus (Trautv.) Fernald)—Birdfoot buttercup | Circumpolar-alpine

Previously recorded in Kimmirut (Polunin 1940; Porsild and Cody 1980; Aiken et al. 2007). Newly recorded in the park. Known from scattered sites across Baffin Island and elsewhere on southern Baffin Island, recorded from Amadjuak Bay, Dorset and Mallik islands and Iqaluit (Aiken et al. 2007; Saarela et al. 2020a).

**Katannilik Territorial Park**: *Saarela et al.* 2120 (CAN, WIN) [MJ-39], *2603* (CAN) [TJ-2]. **Kimmirut**: *Malte s.n.* [118862] (CAN), *s.n.* [118863] (CAN) [KM-1].

**RanunculushyperboreusRottb.subsp.hyperboreus**—Far-northern buttercup | Circumpolar-alpine

Previously recorded in the study area (Polunin 1940; Porsild and Cody 1980; Aiken et al. 2007), but we were unable to locate a voucher specimen. Newly recorded in the park and from Pleasant Inlet. Widespread across Baffin Island and elsewhere on southern Baffin Island, recorded from Amadjuak Bay, Dorset Island, Perry Bay, Resolution Island and Ukiurjak (formerly King Charles Cape) (Aiken et al. 2007; Saarela et al. 2020a).

**Katannilik Territorial Park**: *Saarela et al. 2279* (CAN, MT) [LR-19], *2472* (CAN, WIN) [EC-3], *2607* (CAN) [TJ-4]. **Pleasant Inlet**: *Saarela et al. 2718* (ALA, CAN) [PI-1].

***Ranunculusnivalis* L.**—Snow buttercup | Circumpolar-alpine

Previously recorded in the park (Polunin 1940; Porsild and Cody 1980; Aiken et al. 2007). Not known in Kimmirut. Widespread across Baffin Island and elsewhere on southern Baffin Island, recorded from Dorset and Mallik islands, near Griffin Bay (*Potter 7939*, GH 01836282), Jackman Sound (*Potter 8196*, GH 01836281), Lower Savage Islands, Ogac Lake, Silliman’s Fossil Mount and York Sound (*Wynne-Edwards 7312*, CAN 10050794) (Aiken et al. 2007).

**Katannilik Territorial Park**: *Saarela et al. 2026* (ALA, CAN, MT, UBC, WIN) [MJ-6], *2104* (CAN, WIN) [MJ-36], *2079* (ALTA, CAN, MO, NYBG, US) [MJ-33], *2557* (CAN, O, WIN) [SF-7], *Soper s.n.* (CAN) [SF-1].

***Ranunculuspygmaeus* Wahlenb.** (Fig. 10B)—Pygmy buttercup | Circumpolar-alpine

Previously recorded in the park (Polunin 1940; Porsild and Cody 1980), but Aiken et al. (2007) did not map it, nor have we seen a voucher for Polunin’s 1936 record that Polunin (1940) cited. Our collections confirm the taxon’s presence in the park. Widespread across Baffin Island and elsewhere on southern Baffin Island, recorded from Dorset and Mallik islands, Iqaluit, Ogac Lake and Resolution Island (Aiken et al. 2007).

**Katannilik Territorial Park**: *Saarela et al. 2078* (CAN) [MJ-33], *2086* (CAN) [MJ-33], *2193* (CAN, O, WIN) [GC-6], *2314* (CAN) [LR-6], *2342* (ALA, CAN) [LR-33], *2558* (CAN) [SF-7].

***Ranunculustrichophyllus* Chaix**—Thread-leaved water-crowfoot

Previously recorded in Kimmirut (Polunin 1940; Porsild and Cody 1980; Aiken et al. 2007). Newly recorded in the park and from Pleasant Inlet. Polunin (1940) reported his Lake Harbour collection as R.trichophyllusvar.eradicatus (Laest.) Drew, and Porsild and Cody (1980) treated the record under that name. Following the taxonomy of Ranunculussect.Batrachium DC. of Wiegleb et al. (2017), R.trichophyllusvar.eradicatus is a synonym of *R.confervoides* (Fr.) Fr., a taxon with a restricted Arctic-boreal distribution in Northern Europe. Aiken et al. (2007) treated the taxon as R.aquatilisvar.diffusus With., a synonym of *R.trichophyllus* s.str. (Wiegleb et al. 2017). The correct name for the taxon Elven et al. (2011) recognized as *R.confervoides* is *R.trichophyllus* (Wiegleb et al. 2017). Elsewhere on southern Baffin Island, recorded from Dorset, Mallik and Resolution (*Wynne-Edwards 7249*, CAN 10048788) islands (Aiken et al. 2007; Saarela et al. 2020a). The only other R.sectionBatrachium species in the Canadian Arctic is *R.codyanus* B.Boivin (Wiegleb et al. 2017), not known in the study area.

**Katannilik Territorial Park**: *Saarela et al. 2605* (CAN, O, WIN) [TJ-2]. **Kimmirut**: *Polunin 1137* (CAN) [KM-1]. **Pleasant Inlet**: *Saarela et al. 2716* (ALA, CAN, MT, UBC) [PI-1].

#### Saxifragaceae

##### *Chrysosplenium* L.

***Chrysospleniumtetrandrum* Th.Fr.**—Northern golden saxifrage | Circumpolar & Cordilleran

Previously recorded in Kimmirut (Polunin 1940), but we have not seen a voucher for Polunin’s 1936 collection, which he cited. Neither Porsild and Cody (1980) nor Aiken et al. (2007) mapped the taxon in the study area. Our collections and an Oldenburg one from Kimmirut confirm this species’ presence in the study area. On a rocky slope opposite the Kamik Co-op store entrance, it grew with *Arabisalpina*, *Cerastiumalpinum*, *Poaalpina*, P.glaucasubsp.glauca, P.pratensissubsp.alpigena and *Saxifragacernua*. Below the Kimmirut garbage dump, it grew in a lush sewage-enriched grassy meadow with *Arabisalpina*, *Cerastiumalpinum*, *Salixcalcicola* and *S.glauca*. Known from scattered sites across Baffin Island and elsewhere on southern Baffin Island, recorded from Dorset Island, Foxe Peninsula near Wildbird Islands, Newell Sound (*McLaren 53*, CAN 10056002) and Nuwata (Aiken et al. 2007; Saarela et al. 2020a).

**Kimmirut**: *Oldenburg 86* (MIN) [KM-1], *Saarela et al. 2661* (CAN, MT) [KM-18], *2761* (ALA, ALTA, CAN, MO, MT, O, UBC, US, WIN) [KM-16].

##### *Micranthes* Haw.

***Micranthesfoliolosa* (R.Br.) Gornall** (≡ *Saxifragafoliolosa* R.Br., = S.stellarisvar.comosa Retz.) (Fig. 10C, D)—Leafy-stemmed saxifrage | Circumpolar

Newly recorded from Kimmirut, the park, Pleasant Inlet and the study area. Widespread across Baffin Island and elsewhere on southern Baffin Island, recorded from Amadjuak Bay, Dorset and Mallik islands, Foxe Peninsula near “Storm Cove” (*Manning 213*, CAN 10060500), Iqaluit, Jackman Sound (*Potter 8110*, GH 01711518), Resolution Island (*Potter 8111*, GH 01711520) and Ukiurjak (formerly King Charles Cape) (*Baldwin 1863*, CAN 10060496) (Aiken et al. 2007; Saarela et al. 2020a).

**Katannilik Territorial Park**: *Saarela et al. 2074* (CAN, QFA) [MJ-34], *2157* (CAN) [CR-5], *2178* (CAN, NYBG, US) [GC-1], (ALA, ALTA, CAN, MT, O, UBC, WIN) [LR-16], *2338* (CAN, MO, MT) [LR-12]. **Kimmirut**: *Saarela et al. 2728* (CAN) [KM-5]. **Pleasant Inlet**: *Saarela et al. 2679* (CAN) [PI-3].

***Micranthesnivalis* (L.) Small** (≡ *Saxifraganivalis* L.) (Fig. 10E)—Snow saxifrage | Circumpolar-alpine

Previously recorded in the study area (Aiken et al. 2007), but the specimen from Kimmirut (*Archambault AA271*) has been re-identified as *M.tenuis*. Our collections are, thus, the first confirmed records of the species in Kimmirut. Newly recorded in the park. Widespread across Baffin Island and elsewhere on southern Baffin Island, recorded from Dorset Island, Foxe Peninsula near Wildbird Islands, near Griffin Bay (*Potter 7676*, GH 0171220), Iqaluit, Nuwata, Ogac Lake, Resolution Island (*Potter 8117*, GH 01712002) and Ukiurjak (formerly King Charles Cape) (Aiken et al. 2007; Saarela et al. 2020a).

**Katannilik Territorial Park**: *Saarela et al. 2017* (ALA, CAN, MO, WIN) [MJ-42], *2041* (CAN, O) [MJ-23], *2551* (CAN) [SF-11]. **Kimmirut**: *Saarela et al. 2736* (CAN, MT) [KM-7].

***Micranthestenuis* (Wahlenb.) Small** (Fig. 10F)—Slender saxifrage | Circumpolar-alpine

Newly recorded from the park, Kimmirut and the study area. Archambault’s collection was previously identified as *M.nivalis*. It grew in the park on a moist, north-facing, rocky slope with *Cassiopetetragona*, *Oxyriadigyna* and *Salixherbacea*. Known from scattered sites across Baffin Island and elsewhere on southern Baffin Island, recorded from Dorset Island, Iqaluit, Lower Savage Islands, Ogac Lake and Resolution Island (Aiken et al. 2007; Saarela et al. 2020a).

**Katannilik Territorial Park**: *Saarela et al. 2308* (CAN, MT) [LR-6]. **Kimmirut**: *Archambault AA271* (CAN) [KM-3].

##### *Saxifraga* L.

***Saxifragaaizoides* L.**—Yellow mountain saxifrage | North American (N)–Amphi-Atlantic–European

Previously recorded in Kimmirut (Polunin 1940; Aiken et al. 2007). Newly recorded in the park. Known from scattered sites across Baffin Island and elsewhere on southern Baffin Island, recorded from Dorset Island, Iqaluit, Ogac Lake (*Aiken & LeBlanc 04-058*, CAN 10060005), Resolution Island and York Sound (*Wynne-Edwards 7310*, CAN 10060075) (Aiken et al. 2007; Saarela et al. 2020a).

**Katannilik Territorial Park**: *Saarela et al. 2259* (ALA, ALTA, CAN, UBC, WIN) [LR-20], *2493* (CAN, MT, O) [EC-12]. **Kimmirut**: *Malte s.n.*/*462* [120303] (CAN, GH), *s.n.* [118952] (CAN), *s.n.* [118957] (CAN), *s.n.* [118956] (CAN, GH, US), *s.n.* [118951] (CAN, GH, QFA), *Polunin 289* (GH), *Dutilly 966a* (CAN, MIN), *100B* (MIN), *1060* (CAN, QFA), *9091* (QFA) [KM-1].

***Saxifragacernua* L.**—Nodding saxifrage | Circumpolar-alpine

Previously recorded in Kimmirut (Polunin 1940; Aiken et al. 2007). Newly recorded in the park. Widespread across Baffin Island and elsewhere on southern Baffin Island, recorded from Amadjuak Bay, Dorset and Mallik islands, Foxe Peninsula near Wildbird Islands, Iqaluit and Resolution Island (*Potter 80858*, GH 01619959) (Aiken et al. 2007; Saarela et al. 2020a). **Katannilik Territorial Park**: *Saarela et al. 2077* (ALA, CAN, MT, O) [MJ-33], *2100* (CAN) [MJ-36], *2243* (CAN, NYBG) [WR-10], *2567* (CAN, MO, UBC) [SF-8]. **Kimmirut**: *Soper s.n.* (CAN) [KM-1], *Saarela et al. 2660* (CAN, MT, WIN) [KM-18].

***Saxifragacespitosa* L**.—Tufted saxifrage | Circumpolar-alpine

Previously recorded in Kimmirut (Polunin 1940; Porsild and Cody 1980; Aiken et al. 2007). Newly recorded in the park and from Pleasant Inlet. Widespread across Baffin Island and elsewhere on southern Baffin Island, recorded from Dorset and Mallik islands, Iqaluit, Jackman Sound (*Potter 8093*, GH 01619477), Lower Savage Islands, Ogac Lake and Resolution Island (Aiken et al. 2007; Saarela et al. 2020a).

**Katannilik Territorial Park**: *Saarela et al. 2049* (CAN) [MJ-23], *2082* (CAN, MO, NYBG) [MJ-33], *2103* (CAN, UBC) [MJ-36], *2275* (CAN) [LR-25], *2351* (CAN, GH, MIN, QFA) [LR-32], *2639* (ALTA, CAN) [TJ-3]. **Kimmirut**: *Malte s.n.* [126902] (CAN, GH), *s.n.* [126878] (CAN, GH), *Oldenburg 85* (MIN), *Dutilly 1020* (QFA) [KM-1], *Saarela et al. 2663* (ALA, CAN, WIN) [KM-18]. **Pleasant Inlet**: *Saarela et al. 2701* (CAN, MT, O) [PI-2].

***Saxifragahyperborea* R.Br.** (≡ S.rivularisvar.hyperborea (R.Br.) Hook., ≡ S.rivularissubsp.hyperborea (R.Br.) Dorn, ≡ S.rivularisf.hyperborea (R.Br.) Engl. & Irmsch.)—Pygmy saxifrage | Circumpolar-alpine

Polunin (1940) recorded *S.rivularis* in the study area and mentioned that most southern Arctic plants are S.rivularisf.hyperborea (= *S.hyperborea*), but he did not distinguish infraspecific taxa in his specimen citations. We are unaware of vouchers for his 1934 and 1936 observations nor have we seen a Dutilly collection from Lake Harbour (*Dutilly 9108*, 28 August 1941, CM415539 Carnegie Museum of Natural History, Pittsburgh, Pennsylvania]) determined as S.rivularisf.hyperborea. Our collections confirm the taxon’s presence in the study area. Newly recorded in the park and from Pleasant Inlet. Known from scattered sites across Baffin Island and elsewhere on southern Baffin Island, recorded from Dorset Island, Iqaluit and Ogac Lake (Aiken et al. 2007; Saarela et al. 2020a).

**Katannilik Territorial Park**: *Saarela et al. 1992* (ALA, CAN) [MJ-40], *2076* (CAN, MT, O) [MJ-34], *2179* (ALTA, CAN, WIN) [GC-1], *2309* (CAN) [LR-6], *2336* (CAN) [LR-13]. **Pleasant Inlet**: *Saarela et al. 2680* (CAN) [PI-3].

***Saxifragaoppositifolia* L.**—Purple saxifrage | Circumpolar-alpine

Previously recorded in Kimmirut (Polunin 1940; Porsild and Cody 1980; Aiken et al. 2007). Newly recorded in the park. Widespread across Baffin Island and elsewhere on southern Baffin Island, recorded from Bowdoin Harbour [Schooner Harbour] (*Soper s.n.*, CAN 10001574), Dorset Island, Foxe Peninsula near Wildbird Islands (*Manning 257*, CAN 10060810), Iqaluit, Jackman Sound (*Potter 8107*, GH 01621508), Lower Savage Islands, Nuwata (*Manning 216*, CAN 10060811), Ogac Lake and Resolution Island (Aiken et al. 2007; Saarela et al. 2020a).

**Katannilik Territorial Park**: *Saarela et al. 2012* (CAN) [MJ-42], *2102* (CAN, MT, O) [MJ-36], *2145* (ALA, ALTA, CAN, UBC, WIN) [CR-7]. **Kimmirut**: *Malte s.n.* [118995] (CAN), *Soper s.n.* (CAN, 2 ex) [KM-1].

***Saxifragapaniculata* Mill.** (= *S.aizoon* Jacq., = S.aizoonvar.neogaea Butters, = S.aizoonsubsp.neogaea (D.Löve) Butters) (Fig. 11A, B)—White mountain saxifrage | North American (NE)–Amphi-Atlantic–European

Previously recorded in Kimmirut (Polunin 1940; Aiken et al. 2007). Newly recorded in the park. Elsewhere on Baffin Island, recorded from Amadjuak, Beekman Peninsula, near Griffin Bay (*Potter 8104*, GH 01616858), Iqaluit, Ogac Lake, Pangnirtung and Pond Inlet (Polunin 1940; Porsild and Cody 1980; Aiken et al. 2007).

**Katannilik Territorial Park**: *Saarela et al. 2240* (CAN, NYBG, QFA, US) [WR-6], *2286* (CAN) [LR-22], *2486* (ALA, ALTA, CAN, MT, O, UBC, WIN) [EC-16], *2561* (CAN) [SF-11]. **Kimmirut**: *Malte s.n.*/*1195* [121037] (CAN, GH), *Polunin 429* (GH), *Dutilly 1043A* (MT) [KM-1], *Saarela et al. 2669* (CAN, GH, MIN, NFM) [KM-9], *2749* (CAN, MO) [KM-11].

***Saxifragatricuspidata* Rottb.**—Prickly saxifrage | North American (N)

Previously recorded in Kimmirut and the park (Polunin 1940; Porsild and Cody 1980; Aiken et al. 2007). Widespread across Baffin Island and elsewhere on southern Baffin Island, recorded from Amadjuak Bay, Bowdoin Harbour [Schooner Harbour] (*Soper s.n.*, CAN 66378), Chorkbak Inlet (*Carroll s.n.*, CAN 212184), Dorset Island, Foxe Peninsula near Wildbird Islands (*Manning 252*, CAN 204666), near Griffin Bay (*Potter 8098*, GH 01621812) and York Sound (Aiken et al. 2007; Saarela et al. 2020a).

**Katannilik Territorial Park**: *Soper s.n.* (CAN) [WR-1], *Fleming 3023* (US) [LR-1], *Saarela et al. 1955* (CAN, MT) [MJ-10], *2628* (CAN, GH, MIN, MO, MT, NYBG, QFA, US, UVIC) [TJ-3]. **Kimmirut**: *Malte s.n.*/*484* [120325] (CAN, GH), *s.n.* [126891] (CAN, GH), *s.n.* [119011] (CAN, GH), *s.n.*/*1184* [121026] (CAN, GH), *Soper s.n.* (CAN), *Polunin 332* (GH), *Oldenburg 88*, *95* (GH), *Dutilly 1043*, *9093* (QFA) [KM-1], *Johansen 1121* (C) [KM-20], *Saarela et al. 2670* (ALA, CAN, O) [KM-9].

**Fabales**

#### Fabaceae

##### *Astragalus* L.

***Astragalusalpinus* L.**—Alpine milk-vetch | Circumpolar-alpine

Previously recorded in Kimmirut (Polunin 1940; Porsild and Cody 1980; Aiken et al. 2007). Newly recorded in the park. Widespread across Baffin Island and elsewhere on southern Baffin Island, recorded from Iqaluit, Dorset and Mallik islands and Newell Sound (Aiken et al. 2007; Saarela et al. 2020a).

**Katannilik Territorial Park**: *Soper s.n.* (CAN, LD, 2 ex) [WR-1], *Saarela et al. 2224* (ALA, CAN, GH, MT, NYBG, O, QFA, UBC, US, WIN) [WR-4], *2352* (ALTA, CAN, MO, UBC, US, UTC, UVIC, WTU) [LR-28], *2636* (ASU, CAN) [TJ-3]. **Kimmirut**: *Malte s.n.* [126901] (CAN, GH), *s.n.* [120290] (CAN, US), *s.n.*/*455* [120296] (CAN, GH, US), *s.n.*/*475* [119772] (CAN, GH), *s.n.* [118341] (CAN), *s.n.* [118342] (CAN, GH), *Soper s.n.* (CAN) [KM-1]. **Pleasant Inlet**: *Saarela et al. 2713* (ALA, ASU, CAN, MT, NFM, O, WIN) [PI-1].

***Astragaluseucosmus* B.L.Rob.** (Fig. 11C)—Elegant milk-vetch | North American

Previously recorded in Kimmirut (Polunin 1940; Porsild and Cody 1980; Aiken et al. 2007). Newly recorded in the park and from Pleasant Inlet. Elsewhere on Baffin Island, recorded from Iqaluit and Newell Sound (Aiken et al. 2007) and not otherwise known in the Canadian Arctic Archipelago. A record Aiken et al. (2007) mapped on north-eastern Hall Peninsula is an error; the collection (*Hainault & Norman 6040*, CAN 10070573) is from Frobisher Bay [Iqaluit].

**Katannilik Territorial Park**: *Saarela et al. 2225* (ALA, ALTA, ASU, CAN, MO, MT, NFM, O, UBC, US, UTC, WIN, WTU) [WR-4], *2302* (ASU, CAN, NYBG, QFA) [LR-18], *2500* (CAN, NFM) [LS-3]. **Kimmirut**: *Malte s.n./447* [119771] (CAN, GH, 2 ex; LD, MT, US), *Polunin 417* (CAN) [KM-1], *Archambault AA*253 (CAN) [KM-4]. **Pleasant Inlet**: *Saarela et al. 2712* (ALA, ALTA, ASU, CAN, GH, NFM, O) [PI-1].

##### *Oxytropis* DC., nom. cons.

**Oxytropisdeflexavar.foliolosa (Hook.) Barneby** (≡ O.deflexasubsp.foliolosa (Hook.) Cody) (Fig. 11D)—Pendant pod oxytrope | Amphi-Beringian–North American (W)

Previously recorded in Kimmirut (Polunin 1940; Aiken et al. 2007). Newly recorded in the park and from Pleasant Inlet. Gillespie et al. (2015) reported our collections from the study area. Elsewhere on Baffin Island, recorded in Iqaluit. A record Aiken et al. (2007) mapped on north-eastern Hall Peninsula is an error; the collection (*Hainault & Norman 5427*, CAN 10072227) is from Frobisher Bay [Iqaluit]. Elsewhere in the Canadian Arctic, recorded from western Victoria Island and mainland sites (Porsild and Cody 1980; Cody and Reading 2005; Aiken et al. 2007; Saarela et al. 2013, 2017, 2020b).

**Katannilik Territorial Park**: *Saarela et al. 2530* (ALA, CAN) [SF-26]. **Vicinity of lapis lazuli site**: *Saarela et al. 2504* (ALA, ALTA, CAN, MO, NFM, US, UTC, UVIC, WIN, WTU) [LS-4]. **Kimmirut**: *Polunin 1399* (US), *2333* (CAN) [KM-1], *Saarela et al. 2658* (CAN) [KM-8]. **Pleasant In let**: *Saarela et al. 2714* (ALA, CAN, MT, O, UBC) [PI-1].

***Oxytropismaydelliana* Trautv.** (= O.maydellianasubsp.melanocephala (Hook.) A.E.Porsild) (Fig. 11E)—Maydell’s locoweed | Amphi-Beringian–North American (N)

Previously recorded in Kimmirut and the park (Polunin 1940; Porsild and Cody 1980; Aiken et al. 2007). Newly recorded from Pleasant Inlet. Widespread across Baffin Island and elsewhere on southern Baffin Island, recorded from Amadjuak Bay, Amadjuak Lake, Bowdoin Harbour [Schooner Harbour] (*Soper s.n.*, CAN 10072518), Dorset and Mallik islands, Newell Sound and Silliman’s Fossil Mount (Aiken et al. 2007; Saarela et al. 2020a).

**Katannilik Territorial Park**: *Aiken & Iles 02-060* (CAN) [LS-1] *Saarela et al. 2226* (ALA, ASU, CAN, NFM, UTC, UVIC, WTU) [WR-4], *2353* (ALA, ALTA, CAN, US, WIN) [LR-28], *2426* (ALA, CAN, NYBG) [EC-4]. **Kimmirut**: *Malte s.n.* [118336] (CAN), *s.n.* [118335] (CAN), *s.n.* [118338] (CAN), *s.n.* [121024] (CAN), *s.n.* [119789] (CAN), *Soper s.n.* (CAN), *Polunin s.n.* (US), *334* (US) [KM-1]. **Pleasant Inlet**: *Saarela et al. 2681* (CAN) [PI-3], *2715* (ALA, CAN, MT, O, UBC) [PI-1].

***Oxytropispodocarpa* Gray** (Fig. 11F, G)—Inflated locoweed | Cordilleran & North American (NE)

Newly recorded in the park and study area. Elsewhere on Baffin Island, recorded from Amadjuak Bay and Iqaluit (Aiken et al. 2007) and elsewhere in the Canadian Arctic Archipelago, recorded from Southampton Island (Aiken et al. 2007). A record Aiken et al. (2007) mapped on north-eastern Hall Peninsula is an error; the collection (*Hainault & Norman 5409*, 1970-07-06, CAN 342845) is from Frobisher Bay [Iqaluit].

**Katannilik Territorial Park**: *Saarela et al. 2541* (ALA, ALTA, CAN, MO, MT, O, UBC, WIN) [SF-14].

***Oxytropisterrae-novae* Fernald** (≡ O.campestrisvar.terrae-novae (Fernald) Barneby)—Tundra locoweed | North American (NE)

Aiken et al. (2007) stated Polunin (1940) knew this species from Kimmirut (Lake Harbour), but he reported the taxon only from northern Quebec. Aiken et al. (2007) mapped the taxon in Kimmirut, based on Malte’s collection, the only Canadian Arctic Archipelago record. We did not encounter this species in 2012.

**Kimmirut**: *Malte s.n.* (CAN) [KM-1].

**Rosales**

#### Rosaceae

##### *Dryas* L.

**DryasintegrifoliaVahlsubsp.integrifolia**—Mountain avens | Amphi-Beringian–North American (N)

Previously recorded in Kimmirut and the park (Porsild and Cody 1980; Aiken et al. 2007). Widespread across Baffin Island and elsewhere on southern Baffin Island, recorded from Chorkbak Inlet, Dorset and Mallik islands, Foxe Peninsula near Wildbird Islands, near Griffin Bay (*Potter 7855*, GH 01588266), Iqaluit, Jackman Sound (*Potter 8048*, GH 01588267), Lower Savage Islands, Perry Bay and Resolution Island (Aiken et al. 2007; Saarela et al. 2020a).

**Katannilik Territorial Park**: *Saarela et al. 1972* (ALA, CAN, MT, UBC, WIN) [MJ-8], *Soper s.n.* (CAN) [WR-1], *Saarela et al. 2459* (CAN, O) [EC-2]. **Kimmirut**: *Dutilly 1040* (QFA), *1044* (MT, QFA), *Malte s.n.*/*473* [120314] (CAN, GH), *s.n.* [126859] (CAN, GH), *s.n.*/*617* [119070] (CAN, GH), *s.n.*/*1170* [121012] (CAN, GH), *Oldenburg 89* (MIN), *96B* (MIN), *121* (MIN), *Sanson 23* (TRT), *Soper s.n.* (CAN), *Tallman s.n.* (MIN) [KM-1], *Johansen 1123* (C) [KM-20].

##### *Potentilla* L.

**Potentillaanserinasubsp.groenlandica Tratt.** (= *Argentinaegedii* (Wormsk.) Rydb., = P.anserinasubsp.egedii (Wormsk.) Hiitonen)—Greenland silverweed | Amphi-Beringian–North American (N)–Amphi-Atlantic–European (N)

Aiken et al. (2007) published photos of this taxon taken in 2002 at “Soper Lake, landing beach near Kimmirut” and indicated no voucher exists for the occurrence. The record they mapped is likely the unvouchered observation; we are unaware of collections from the area. Our Kimmirut collection vouchers its occurrence there. Newly recorded in the park and from Pleasant Inlet. Elsewhere on Baffin Island, recorded from Iqaluit and Brewster Point (Aiken et al. 2007) and elsewhere in the Canadian Arctic Archipelago, recorded from Victoria Island (Aiken et al. 2007; Saarela et al. 2020b).

**Katannilik Territorial Park**: *Saarela et al. 2629* (ALA, CAN, O) [TJ-3]. **Kimmirut**: *Saarela et al. 2765* (ALA, ALTA, CAN, NYBG, O) [KM-16]. **Pleasant Inlet**: *Saarela et al. 2683* (CAN) [PI-3].

***Potentillacrantzii* (Crantz) Beck**—Crantz’s cinquefoil | Amphi-Atlantic–European–Asian (W)

Newly recorded in Kimmirut and the study area, based on our collection and Johansen’s in 1927. Elsewhere on Baffin Island, recorded from Ogac Lake (Aiken et al. 2007). A collection from Cumberland Sound by L. Kumlien in 1878 has been reported as this species (Polunin 1940; McLaren 1964; Porsild and Cody 1980); the voucher should be confirmed. Elsewhere in the Canadian Arctic Archipelago, recorded on Nottingham Island (Porsild and Cody 1980; Aiken et al. 2007).

**Kimmirut**: *Johansen 1122* (C) [KM-20], *Saarela et al. 2754* (ALA, ALTA, CAN, MO, MT, O, UBC, US, WIN) [KM-10].

**PotentillahyparcticaMaltesubsp.hyparctica**—Arctic cinquefoil | Circumpolar

Newly recorded in the park and the study area. Elsewhere on southern Baffin Island, recorded on Mallik Island (Saarela et al. 2020a). This is the more northerly of the two subspecies. Widespread on Baffin Island north of Nettilling Lake (Aiken et al. 2007).

**Katannilik Territorial Park**: *Saarela et al. 2044* (CAN, O) [MJ-23], *2169* (ALA, CAN) [CR-4], *2311* (CAN) [LR-6].

**Potentillahyparcticasubsp.elatior (Abrom.) Elven & D.F.Murray** (≡ P.emarginatavar.elatior Abrom.)—Tall Arctic cinquefoil | North American (N)

Previously recorded in Kimmirut and the park (Polunin 1940; Porsild and Cody 1980; Aiken et al. 2007). This is the more southerly of the two subspecies and we collected it more frequently than subsp. hyparctica in the park. On Baffin Island, recorded as far north as Clyde River. Elsewhere on southern Baffin Island, recorded from Dorset and Mallik islands, Jackman Sound (*Potter 8354*, MT00056284, det. L. Brouillet), Lower Savage Islands, Ogac Lake (*Aiken and LeBlanc 04-039*, CAN 10063431) and Resolution Island (Aiken et al. 2007; Saarela et al. 2020a).

**Katannilik Territorial Park**: *Saarela et al. 1985* (ALA, CAN, MT) [MJ-13], *2106a* (CAN) [MJ-36], *2160* (CAN) [CR-3], *2138* (CAN) [CR-8], *2212* (CAN, US) [GC-8], *Soper s.n.* (CAN) [WR-1], *Aiken & Iles 02-58* (CAN) [LS-1], *Soper s.n.* (CAN) [SF-1], *Saarela et al. 2598* (ALA, CAN, O) [TJ-1]. **Kimmirut**: *Dutilly* 1008 (MT, CAN, 2 ex), *Malte s.n.* [119102] (CAN), *s.n.* [119090] (CAN) [KM-1].

***Potentillanivea* L.**—Snow cinquefoil | Circumpolar-alpine

Previously recorded in Kimmirut (Polunin 1940; Aiken et al. 2007). Newly recorded in the park. Elsewhere on Baffin Island, recorded from Amadjuak Bay (*Soper s.n.*, CAN 10064312), Amadjuak Lake, Beekman Peninsula, Inugsuin Fiord, Iqaluit, Nettilling Lake, Ogac Lake (*Aiken and LeBlanc 04-217*, CAN 10064304) and Silliman’s Fossil Mount (Aiken et al. 2007).

**Katannilik Territorial Park**: *Saarela et al. 2016* (CAN) [MJ-42], *2018a* (CAN) [MJ-42], *2018b* (ALA, CAN) [MJ-42], *1965* (ALA, ALTA, CAN, O, US) [MJ-11], *1957* (ALA, CAN, MT, O, UBC) [MJ-10], *2106b* (CAN) [MJ-36], *2170* (CAN) [CR-4], *2207* (ALA, CAN, MT, O) [GC-9], *2227* (CAN, MO, NYBG, UVIC, WTU) [WR-4], *2273* (ALA, CAN, O) [LR-25], *2274* (CAN, O) [LR-25], *2550* (CAN, MIN, QFA) [SF-11], *2625* (CAN) [TJ-3]. **Kimmirut**: *Malte s.n.* [119080] (CAN), *s.n.* [119093] (CAN) [KM-1], *Saarela et al. 2778* (CAN), *2785* (CAN, WIN) [KM-19].

***Potentillapulchella* R.Br.**—Pretty cinquefoil | Circumpolar

Previously recorded in Kimmirut (Polunin 1940; Porsild and Cody 1980; Aiken et al. 2007). We did not encounter this taxon in 2012. Known from scattered sites across Baffin Island. Porsild and Cody (1980) mapped a record for Hall Peninsula; we are unaware of a voucher. Not otherwise recorded on southern Baffin Island (Aiken et al. 2007). A record Aiken et al. (2007) mapped west of the study area, based on Porsild and Cody’s (1980) map is an error; the record mapped along southern Baffin Island in the latter treatment is the Kimmirut one.

**Kimmirut**: *Malte s.n.* [119100] (CAN) [KM-1].

##### *Rubus* L.

***Rubuschamaemorus* L.** (Fig. 11H)—Cloudberry | Circumboreal-polar

Newly recorded in the park and study area. This species grew along a stream just above the Livingstone River falls in dense moss under *Betulaglandulosa*. Elsewhere on Baffin Island, recorded from Cape Tanfield, ca. 25 km southeast of Kimmirut (*Sutherland s.n.*, CAN 10070339); Aiken et al. (2007) mapped this record. Elsewhere on southern Baffin Island, recorded from Foxe Peninsula, the north side of Frobisher Bay, eastern Meta Incognito Peninsula and Resolution Island (Porsild and Cody 1980; Aiken et al. 2007). We are unaware of vouchers for these sites. Elsewhere in the Canadian Arctic Archipelago, known from Upper Savage Islands southeast of Kimmirut (*Bell s.n.*, 1884-08-15, CAN 10070329) and Coats, King William, Southampton and Victoria islands (Porsild and Cody 1980; Aiken et al. 2007; Saarela et al. 2020b).

**Katannilik Territorial Park**: *Saarela et al. 2304* (ALA, CAN, MT, O, UBC, US, WIN) [LR-3].

##### *Sibbaldia* L.

***Sibbaldiaprocumbens* L.**—Creeping sibbaldia | Circumpolar-alpine

Newly recorded in the park and study area. This species grew in a grassy meadow with *Calamagrostiscanadensis*, *Carexarctogena* and *C.bigelowii*. Elsewhere on Baffin Island, recorded from Beekman Peninsula, Brewster Point, Cornelius Grinnell Bay (*Aiken 08-10*, CAN 10070499), Iqaluit (*Aiken 06-036*, CAN 10070498), Newell Sound (*McLaren 39*, CAN 10070493), Ogac Lake, Sunneshine Fiord and York Sound (*Wynne-Edwards 7334*, CAN 10070492) (Polunin 1939; Aiken et al. 2007). Not otherwise known in the Canadian Arctic Archipelago.

**Katannilik Territorial Park**: *Saarela et al. 2345* (ALA, CAN, MT, O, US, WIN) [LR-35].

**Fagales**

#### Betulaceae

##### *Betula* L.

**Figure 12.**
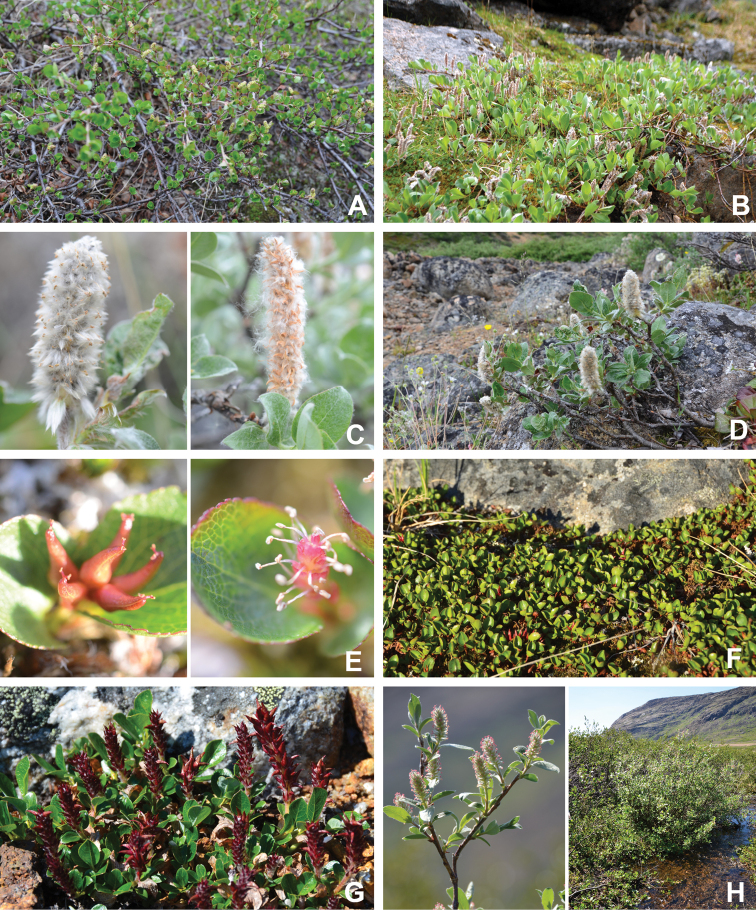
**A***Betulaglandulosa* habit, *Saarela et al. 2027***B***Salixarctophila* habit, *Saarela et al. 2325***C***Salixcalcicola* staminate catkin (left), *Saarela et al. 2247*, pistillate catkin (right), *Saarela et al. 2236***D***Salixcalcicola* habit, *Saarela et al. 2247***E***Salixherbacea* pistillate catkin (left), *Saarela et al. 2164*, staminate catkin (right), *Saarela et al. 2165***F***Salixherbacea* habit, *Saarela et al. 2164***G***Salixuva-ursi* habit and pistillate catkins, *Saarela et al. 2699***H**Salixglaucavar.cordifolia pistillate catkins (left) and habit (right), *Saarela et al. 1943*. Photos **A, B** by P.C. Sokoloff and **C–H** by R.D. Bull.

***Betulaglandulosa* Michx.** (Fig. 12A)—Glandular birch | North American (N)

Previously recorded in Kimmirut and the park (Polunin 1940; Aiken et al. 2007). Elsewhere on Baffin Island, recorded from Auyuittuq National Park, Burwash Bay, Cormack Bay, Cumberland Gulf, Iqaluit, Ogac Lake, Peter Force Island (*Wynne-Edwards 7385*, CAN 10026660) and Ward Inlet (*Freeman s.n.*, NY 2475853) (Aiken et al. 2007).

**Katannilik Territorial Park**: *Saarela et al. 2027* (ALTA, CAN, UBC, UVIC) [MJ-6], *1919* (CAN, GH, MIN, NFM, O, QFA, WLU, WTU) [MJ-4], *Soper s.n.* (CAN, NY) [WR-1]. **Kimmirut**: *Malte s.n.* [118711] (CAN, GH), *s.n.* [118712] (CAN, GH), *Polunin 328* (US) [KM-1], *Johansen 1114* (C) [KM-20], *Archambault AA269* (CAN) [KM-3].

**Celastrales**

#### Celastraceae

##### *Parnassia* L.

***Parnassiakotzebuei* Cham. & Schlecht.**—Kotzebue’s grass-of-Parnassus | Amphi-Beringian–North American (N)

Previously recorded in Kimmirut (Aiken et al. 2007). Newly recorded in the park. Not known elsewhere on Baffin Island. Aiken et al. (2007) erroneously mapped a record in the Amadjuak Bay area, based on Porsild and Cody’s (1980) map, which includes only the Lake Harbour site. At our northernmost site (EC-19), this species grew at the high watermark along the bank of the Soper River with *Astragalusalpinus*, Carexbigelowiisubsp.bigelowii and *Salixglauca*. Near Soper Falls, it grew along the sandy banks of a small pond near the park emergency shelter and outhouse with *Agrostismertensii*, *Chamaenerionlatifolium* and *Oxyriadigyna*. On a small island in Tasiujarjuaq, it grew on mossy turf along a rocky beach below the high water line with *Chamaenerionlatifolium*, *Juncusarcticus* and *Salixarctophila*. Elsewhere in the Canadian Arctic Archipelago, recorded on Banks and Victoria islands (Porsild and Cody 1980; Aiken et al. 2007; Saarela et al. 2020b).

**Katannilik Territorial Park**: *Saarela et al. 2416* (CAN, WIN) [EC-19], *2522* (ALA, CAN, O) [SF-24], *2599* (CAN, MT) [TJ-1]. **Kimmirut**: *Polunin 2320*, *1467* (GH) [KM-1].

**Malpighiales**

#### Salicaceae

##### *Salix* L.

***Salixarctica* Pall.**—Arctic willow | Circumpolar-alpine

Previously recorded in Kimmirut (Porsild and Cody 1980), but neither Polunin (1940) nor Aiken et al. (2007) mapped these records. Newly recorded in the park. Widespread across Baffin Island and elsewhere on southern Baffin Island, recorded from Amadjuak Bay, Beekman Peninsula, Brewster Point, Dorset and Mallik islands, Iqaluit, Lower Savage Islands, Ogac Lake, Peale Point, Resolution Island, Silliman’s Fossil Mount, Ukiurjak (formerly King Charles Cape) and a site on south-eastern Baffin Island (*Scott BSL-36*, CAN 10001982) (Aiken et al. 2007; Saarela et al. 2020a). Ostenfeld determined *Johansen 1111* (C), from the Lake Harbour area, as *Salixarctica* × *S.glauca*.

**Katannilik Territorial Park**: *Saarela et al. 2058* (ALA, CAN, O) [MJ-16], *2060* (ALA, CAN, O) [MJ-16], *2316* (CAN) [LR-6], *2343* (CAN) [LR-14], *2569* (CAN) [SF-8]. **Kimmirut**: *Polunin 2124*, *475* (F), *285* (MICH), *302*, *536* (US), *479* (NY), *907* (MIN) [KM-1].

***Salixarctophila* Cockerell ex A.Heller** (Fig. 12B)—Northern willow | North American (N)

Previously recorded in Kimmirut and the park (Polunin 1940; Aiken et al. 2007). Widespread across Baffin Island and elsewhere on southern Baffin Island, recorded from Beekman Peninsula, Dorset and Mallik islands, Iqaluit, near Cape Haven, Ogac Lake (*Consaul et al. 2358*, CAN 10023425), York Sound (*Wynne-Edwards 7338*, CAN 10023445) and a site on south-eastern Baffin Island (*Scott 40*, CAN 10023446) (Aiken et al. 2007; Saarela et al. 2020a). *Salixarctophila* forms natural hybrids with *S.arctica*, S.glaucavar.cordifolia and *S.uva-ursi*. (Argus 2010). George Argus (CAN) determined a collection from Lake Harbour (*Malte s.n.* [118635], CAN 10023676) as a putative hybrid between *S.arctophila* and *S.uva-ursi* in 2001. In 1932, Björn Floderus determined several of Soper’s collections from the park as hybrids between *S.arctophila* and *S.glauca* (CAN 10023670, CAN 10023671, CAN 10023672, CAN 10023673). Argus later annotated (without date) CAN 10023673 as “probably Salixglaucavar.cordifolia”, but did not annotate the rest of the putative hybrids Floderus determined.

**Katannilik Territorial Park**: *Saarela et al. 1917* (ALA, CAN, O, US, WIN) [MJ-4], *1918* (ALA, CAN, O, US, WIN) [MJ-4], *1944* (CAN, MT, UBC) [MJ-5], *Soper s.n.* (CAN) [WR-1], *Saarela et al. 2325* (ALTA, CAN, MO) [LR-14], *2326* (CAN) [LR-14], *2451* (CAN) [EC-8]. **Kimmirut**: *Malte s.n.* [120311] (CAN, NY, US), *s.n.* [118804] (CAN), *s.n.* [118803] (CAN, US), *Oldenburg 102B* (MIN) [KM-1].

**SalixcalcicolaFernald & Wiegandvar.calcicola** (≡ S.lanatasubsp.calcicola (Fernald & Wiegand) Hultén) (Fig. 12C, D)—Limestone willow | North American (NE)

Previously recorded in Kimmirut (Polunin 1940; Porsild and Cody 1980; Aiken et al. 2007). Newly recorded in the park and from Pleasant Inlet. Recorded on Baffin Island from scattered sites as far north as Inugsuin Fiord and elsewhere on southern Baffin Island, recorded from Amadjuak Bay, Dorset and Mallik islands and Silliman’s Fossil Mount (Aiken et al. 2007; Saarela et al. 2020a).

**Katannilik Territorial Park**: *Saarela et al. 2246* (CAN, MT, UBC, WIN) [WR-9], *2247* (CAN, MT, UBC, WIN) [WR-9], *2260* (ALA, CAN, O) [LR-20], *2261* (CAN) [LR-20], *2481* (CAN, MO, US) [EC-15]. **Kimmirut**: *Dutilly 1062* (MT), *Oldenburg 98A*, *99* (MIN), *Tallman s.n.* (MIN), *Malte s.n.* [120315] (CAN), *s.n.* [120313] (CAN, NY, US), *s.n.* [126863] (CAN), *s.n.* [126865] (CAN, NY, US) [KM-1]. **Pleasant Inlet**: *Saarela et al. 2703* (ALTA, CAN) [PI-2].

***Salixfuscescens* Anderss.**—Alaska bog willow

Our collections are the first records for the park, the study area and Baffin Island. Gillespie et al. (2015) provide details. Elsewhere in the Canadian Arctic Archipelago, Aiken et al. (2007) reported the species from a collection on south-eastern Victoria Island (Aiken et al. 2007). George Argus revised the poor specimen to “possibly” *S.planifolia* (Saarela et al. 2020b). *Salixfuscescens* is thus not known elsewhere in the Canadian Arctic Archipelago.

**Katannilik Territorial Park**: *Saarela et al. 2361* (CAN), *2362* (ALA, CAN, O) [LR-8].

**Salixglaucavar.cordifolia (Pursh) Dorn** (= S.glaucavar.callicarpaea (Trautv.) Argus, = S.glaucasubsp.callicarpaea (Trautv.) Böcher, = S.cordifoliavar.callicarpaea (Trautv.) Fernald) (Fig. 12H)—Beautiful willow | North American (NE)

Previously recorded in Kimmirut and the park (Polunin 1940; Aiken et al. 2007). Newly recorded from Pleasant Inlet. Elsewhere on Baffin Island, recorded from between Amadjuak Bay and Chorkbak Inlet, Beekman Peninsula, Iqaluit, Lake Gillian, Ogac Lake, a few sites in the Pangnirtung area and Peter Force Island (Aiken et al. 2007).

**Katannilik Territorial Park**: *Saarela et al. 2059* (ASU, CAN, NFM, UTC) [MJ-16], *1943* (ALA, CAN, GH, MIN, O, QFA, WIN) [MJ-5], *1949* (CAN, QFA) [MJ-5], *2155* (ALA, CAN, O) [CR-5], *2156* (ALA, CAN, O) [CR-5], *2251* (CAN, MO, MT, NYBG, UBC, WIN) [WR-8], *2252* (CAN, MO, MT, NYBG, UBC, WIN) [WR-8], *2253* (CAN, MT, UBC, WIN) [WR-8], *Soper s.n.* (CAN) [WR-1], *Aiken & Iles 02-051a* (CAN) [WR-2], *Saarela et al. 2324* (CAN, UVIC, WTU) [LR-14], *2594* (ALTA, CAN, UVIC, WTU) [TJ-1], *2595* (ALTA, CAN) [TJ-1]. **Kimmirut**: *Dutilly 9144* (MT), *Malte s.n.* [118611] (CAN), *s.n.* [118686] (CAN), *s.n.* [118697] (CAN, NY), *s.n.* [118698] (CAN, NY, US), *s.n.* [118707] (CAN, NY, US), *s.n.* [118708] (CAN, F), *s.n.* [118709] (CAN, NY), *s.n.* [118710] (CAN), *s.n.* [118809] (CAN), *s.n.* [118810] (CAN, NY, US), *s.n.* [118813] (CAN), *s.n.* [118814] (CAN), *s.n.* [120297] (CAN, US), *s.n.* [120306] (CAN), *s.n.* [120308] (CAN, NY, US), *s.n.* [120309] (CAN), *s.n.* [120310] (CAN), *s.n.* [120319] (CAN), *s.n.* [120321] (CAN, NY), *s.n.* [120324] (CAN, NY, US), *s.n.* [120327] (CAN), *s.n.* [120329] (CAN, NY), *s.n.* [121017] (CAN), *s.n.* [121018] (CAN), *s.n.* [121020] (CAN), *s.n.* [121025] (CAN), *s.n.* [121029] (CAN), *s.n.* [121031] (CAN, NY, US), *s.n.* [121033] (CAN, NY), *s.n.* [126857] (CAN, NY), *s.n.* [126867] (CAN, NY, US), *s.n.* [126872] (CAN), *s.n.* [126874] (CAN), *s.n.* [126876] (CAN), *s.n.* [126895] (CAN, NY), *s.n.* [126904] (CAN), *s.n.* [118812] (CAN), *s.n.* [118814-B] (CAN), *s.n.* [12031-] (CAN), *Archambault AA255* (CAN) [KM-4], *Saarela et al. 2671* (CAN, US) [KM-9], *2791* (CAN, NFM, UTC) [KM-19], *2763* (CAN, US) [KM-16]. **Pleasant Inlet**: *Saarela et al. 2704* (CAN) [PI-2].

***Salixherbacea* L.** (Fig. 12E, F)—Snowbed willow | North American (NE)–Amphi-Atlantic–European (N/C)

Previously recorded in Kimmirut (Polunin 1940; Aiken et al. 2007). Newly recorded in the park. Widespread across Baffin Island east of 81°N. Elsewhere on southern Baffin Island, recorded from near Cape Dorchester, Dorset and Mallik islands, Lower Savage Islands, Ogac Lake, Resolution Island, Silliman’s Fossil Mount and a site on southeast Baffin Island (Aiken et al. 2007; Saarela et al. 2020a).

**Katannilik Territorial Park**: *Saarela et al. 1952* (CAN, MT, WIN) [MJ-10], *2164* (ALA, CAN, O) [CR-2], *2165* (ALA, CAN, O) [CR-2], *2315* (CAN) [LR-6]. **Kimmirut**: *Malte s.n.* [118636] (CAN) [KM-1], *Johansen 1113* (C) [KM-20].

**Figure 13.**
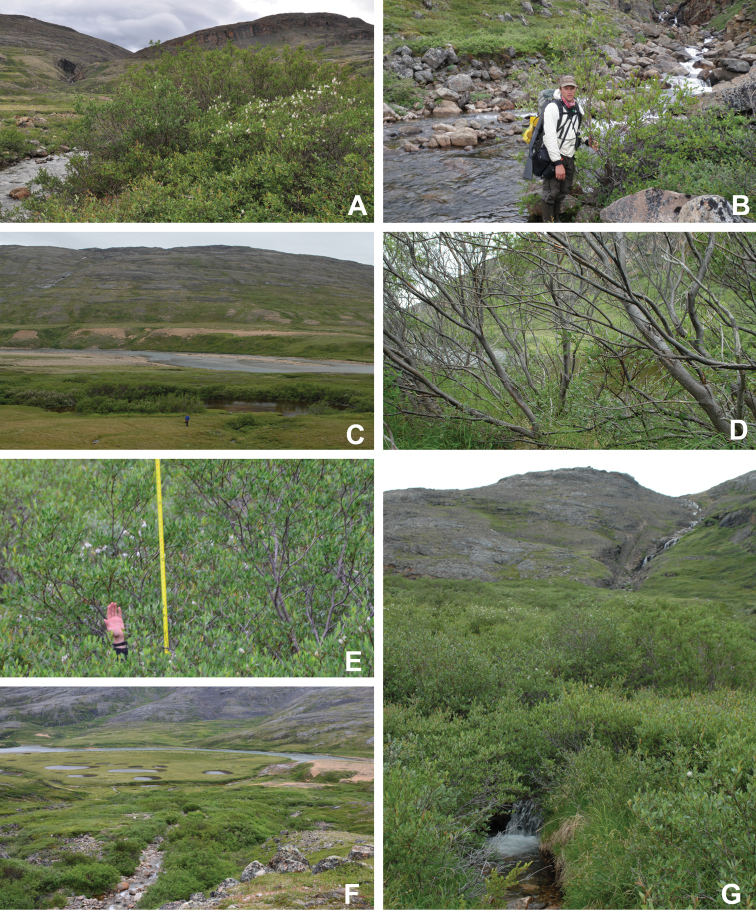
*Salixplanifolia***A** Stand near the mouth of Willow River, *Saarela et al. 2248*, *2249*, *2250***B** R.D. Bull next to *Salixplanifolia* stems at Willow River mouth **C–G** Large stands ca. 5 km south of the confluence of Soper and Livingstone rivers, *Saarela et al. 2393–2395***C** habitat **D** understorey **E** P.C. Sokoloff’s hand while measuring stand height **F, G** habit. Photos **A–E, G** by L.J. Gillespie and **F** by R.D. Bull.

***Salixplanifolia* Pursh** (Fig. 13)—Tea-leaved willow | North American (N)

Previously recorded in the park (Polunin 1940; Porsild 1957, 1964; Porsild and Cody 1980; Aiken et al. 2007). On his maps, Porsild placed the dot for Soper’s 1931 collections from Willow River slightly west of the actual locality. In 2002, Aiken and Iles collected the species in the same area as Soper. Aiken et al. (2007) mapped the site accurately; however, they also included a dot on their map further west, near Amadjuak Lake, based on Porsild and Cody’s (1980) map. They mistakenly interpreted the dot in Porsild’s maps as representing a different site from the Willow River one. Not otherwise known on Baffin Island. Elsewhere in the Canadian Arctic Archipelago, recorded on one or more of Nottingham Island, Mill Island and Salisbury Island in Hudson Strait. The dot in Porsild and Cody’s (1980) map covers the area of these islands. Johansen (1934) did not include the species in his Nottingham Island species list, Polunin (1952) did not include the species in his Mill Island species list and Aiken et al. (2007) did not map any records on these islands. The species’ occurrence in this area requires confirmation. Also recorded on Victoria Island, based on a depauperate specimen previously determined as *S.fuscescens* (Saarela et al. 2020b). This occurrence requires field-based verification.

Individuals of this species are the largest plants on Baffin Island. They grow in sheltered places in the park where moisture is plentiful during the growing season and snow builds up in the winter and spring, providing protection (Polunin 1940). Soper documented large *S.planifolia* stands along Willow River in 1931. He observed that willows “… reach the greatest observed height [within the Soper River valley] of more than 12 ft [3.6 m]” on the banks of Willow River (Soper 1936: 434). His collection labels indicate the plants grew “along streams”. Susan Aiken visited the same site in 2002. The label on her voucher (*Aiken & Iles 02-051*) states, “plants growing to over 3 m tall in the shelter of the river valley”. We visited the site in 2012 and determined that the largest individuals reached 3.4 m (11 ft) high. The maximum heights recorded for the tallest plants in the population in 1931, 2002 and 2012 are similar. Based on these data, environmental factors appear to limit the maximum heights of the plants at this locality.

We also studied the species at several other localities in the Soper River valley. Near Mount Joy, it formed a dense thicket in a damp meadow around a pond with *Betulaglandulosa* and *Calamagrostiscanadensis*. Plants at this site reached heights of ca. 1 m. At a site near Cascade River, it formed a thicket with *Betulaglandulosa* on a moist, rocky slope along a creek (Fig. 12C–F). At this locality, plants reached 1.2 m. At a site 5 km downstream of the Livingstone and Soper rivers confluence, it formed a thicket along a pond’s edge at the base of a steep, rocky, west-facing slope. The thicket’s understorey was species-poor, comprising dense *Calamagrostiscanadensis* and *Pyrolagrandiflora*. At this locality, plants reached 3.7 m [12 ft] high. At a site 9.5 km downstream of the Livingstone and Soper rivers’ confluence, *S.planifolia* formed a dense stand ca. 30 × 50 m along gullies at the base of an east-facing slope (Fig. 12F, G). At this site, the tallest plants reached 3.7 m [12 ft] high. Other species present at the site were *Calamagrostiscanadensis*, *Chamaenerionangustifolium*, *Pyrolagrandiflora*, Rhododendrontomentosumsubsp.decumbens and Salixglaucavar.cordifolia. Elsewhere in the Canadian Arctic Archipelago, willow thickets as tall as those in the Soper River valley occur on Victoria Island, formed by *Salixalaxensis* Coville (Saarela et al. 2020b). Willow thickets reaching heights well beyond that of typical Low Arctic vegetation also occur in Nunavik (northern Quebec). Maycock and Matthews (1966) characterized the ecology of a thicket dominated by *S.planifolia* and *S.alaxensis* that grew in a deep valley some 32 mi [51 km] south of Deception Bay, Nunavik, in which plants reached nearly 16 ft [4.9 m].

**Katannilik Territorial Park**: *Saarela et al. 1947* (CAN), *1948* (ASU, CAN, NFM) [MJ-5], *2153* (ALTA, CAN, MO, US), *2154* (ALTA, CAN, MO, US) [CR-5], *2248* (CAN, NFM, NYBG, UTC, UVIC, WTU), *2249* [wood sample taken] (CAN, NYBG, UVIC, WTU), *2250* (CAN, WIN) [WR-8], *Aiken & Iles 02-051* (CAN) [WR-2], *Soper s.n.* (CAN), *Soper s.n.* (CAN), *Soper s.n.* (CAN) [WR-1], *Saarela et al. 2393* (ALA, CAN, GH, MIN, O, QFA, WIN), *2394* (ALA, CAN, O, WIN) [LC-1], *2395* (ALA, CAN, O) [LC-1], *2404* (CAN, MT, UBC), *2405* (CAN, MT, QFA, UBC) [LC-4].

***Salixreticulata* L.**—Net-vein willow | Circumpolar-alpine

Previously recorded in Kimmirut and the park (Polunin 1940; Aiken et al. 2007). Widespread on Baffin Island and elsewhere on southern Baffin Island, recorded from Dorset and Mallik islands, Foxe Peninsula near Wildbird Islands, Iqaluit, Jackman Sound (*Potter 8691*, MT00056285, *n.v.*), Lower Savage Islands, Resolution Island, York Sound (*Wynne-Edwards 7269*, CAN 10030333) and a site on south-eastern Baffin Island (*Scott s.n.*, CAN 10030273) (Aiken et al. 2007; Saarela et al. 2020a).

**Katannilik Territorial Park**: Soper *s.n.* (CAN) [WR-1], *Saarela et al. 2147* (CAN, MT, UBC) [CR-6], *2216* (ALA, CAN, O), *2217* (ALA, CAN, O) [GC-8]. **Kimmirut**: *Malte s.n.* [126880] (CAN), *s.n.* [120307] (CAN, NY), *s.n.* [126870] (CAN, NY, US), *s.n.* [118650] (CAN), *s.n.* [121016] (CAN), *s.n.* [121043] (CAN), *s.n.* [121019] (CAN), *Polunin 453* (US), *Oldenburg 82* (MIN, 2 ex), *Archambault AA256* (CAN) [KM-4], *Saarela et al. 2672* (CAN, WIN), *2673* (CAN, WIN) [KM-9].

***Salixuva-ursi* Pursh** (Fig. 12G)—Bearberry willow | North American (NE)

Previously recorded in Kimmirut and the park (Polunin 1940; Aiken et al. 2007). Newly recorded from Pleasant Inlet. Elsewhere on Baffin Island, recorded from between Amadjuak Bay and Chorkbak Inlet, Beekman Peninsula, Dorset Island, Iqaluit, Ogac Lake (*McLaren s.n.*, CAN 10032628; *Consaul et al. 2361*, CAN 10032612), Peale Point and the Penny Highlands (Aiken et al. 2007; Saarela et al. 2020a).

**Katannilik Territorial Park**: *Aiken & Iles 02-050 a* (CAN) [CR-1], *Saarela et al. 2149* (ALA, CAN, MO, WIN) [CR-9]. **Kimmirut**: *Malte s.n.* [118657] (CAN), *s.n.* [118655] (CAN), *s.n.* [118634] (CAN, NY), *s.n.* [118656] (CAN, US), *Polunin 301* (US) [KM-1]. **Pleasant Inlet**: *Saarela et al. 2695* (CAN, MT) [PI-3], *2699* (CAN, MT) [PI-2], *2721* (CAN) [PI-1].

**Myrtales**

#### Onagraceae

##### *Chamaenerion* Ség.

**Chamaenerionangustifolium(L.)Scop.subsp.angustifolium** (≡ *Epilobiumangustifolium* L., ≡ *Chamaerionangustifolium* (L.) Holub)—Fireweed | Circumboreal-polar

Previously recorded in the park (Polunin 1940; Porsild 1957, 1964; Porsild and Cody 1980; Aiken et al. 2007). We encountered two populations of this species. Near Mount Joy, it grew on a dry, rocky riverbank slope with *Arnicaangustifolia*, *Betulaglandulosa*, *Carexbigelowii*, *Chamaenerionlatifolium*, *Festucabrachyphylla*, *Saxifragatricuspidata*, *Vacciniumuliginosum* and *V.vitis-idaea.* Plants at this site were not in bud or flower on 1 July 2012, our sampling date. Soper’s collection, gathered along the Soper River on 1 July 1931, also bears no reproductive material. Near Livingstone River, it grew along a meadow edge, in a low *Betula*–*Salix* thicket and in the understorey of a large *Salix* stand. At this site, plants were in bud (13 July 2012). Elsewhere on Baffin Island, recorded from Apex Hill (Iqaluit area), Beekman Peninsula, the head of Cumberland Sound and Pangnirtung (Porsild and Cody 1980; Aiken et al. 2007). Not otherwise known in the Canadian Arctic Archipelago.

**Katannilik Territorial Park**: *Soper s.n.* (CAN) [WR-1], *Saarela et al. 1954* (ALTA, CAN, MO, WIN) [MJ-10], *2402* (ALA, CAN, O) [LC-3].

***Chamaenerionlatifolium* (L.) Sweet** (≡ *Epilobiumlatifolium* L., ≡ *Chamerionlatifolium* (L.) Holub)—River beauty | Circumpolar-alpine

Previously recorded in Kimmirut and the park (Polunin 1940; Porsild 1957, 1964; Porsild and Cody 1980; Aiken et al. 2007). Newly recorded from Pleasant Inlet. Widespread across Baffin Island and elsewhere on southern Baffin Island, recorded from “Aitken Lakes” (ca. 13 km west-northwest of Kinngait), Amadjuak Bay, Bowdoin Harbour [Schooner Harbour] (*Soper s.n.*, CAN 10001730), Dorset and Mallik islands, Foxe Peninsula near Wildbird Islands (*Manning 281*, CAN 10001713), near Griffin Bay (*Potter 8208*, GH 01675678), Iqaluit, Jackman Sound (*Potter 8207*, GH 01675690) and York Sound (*Wynne*-*Edwards 7309*, CAN 10001756) (Aiken et al. 2007; Saarela et al. 2020a).

**Katannilik Territorial Park**: *Soper s.n.* (CAN) [WR-1], *Saarela et al. 1967* (ALTA, CAN, MO, MT, NYBG, UBC, US) [MJ-11]. **Kimmirut**: *Malte s.n.*/*479* [120320] (CAN, GH), *s.n.* [126853] (CAN, GH), *s.n.*/*559* [119128] (CAN, GH), *Soper s.n.* (CAN) [KM-1], *Johansen 1124* (C) [KM-20]. **Pleasant Inlet**: *Saarela et al. 2700* (ALA, CAN, O, WIN) [PI-2].

**Brassicales**

#### Brassicaceae

##### *Arabidopsis* Heynh.

**Figure 14.**
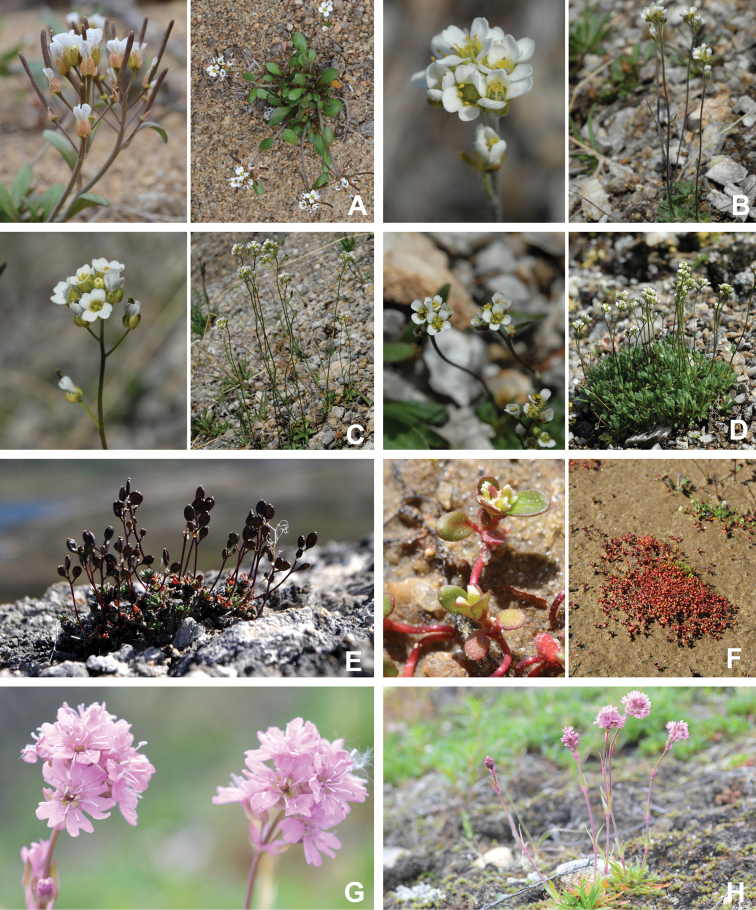
**A***Arabidopsisarenicola* inflorescence (left) and habit (right), *Saarela et al. 2429***B***Drabaarctica* inflorescence (left) and habit (right), *Saarela et al. 2509***C***Drabaglabella* inflorescence (left) and habit (right), *Saarela et al. 2508***D***Drabalactea* inflorescence (left) and habit (right), *Saarela et al. 2510***E***Drabanivalis* habit, *Saarela et al. 2542***F***Koenigiaislandica* inflorescence (left) and habit (right), *Saarela et al. 2359***G***Viscariaalpina* inflorescences, *Saarela et al. 2241***H***Viscariaalpina* habit, *Saarela et al. 2241*. Photos by **A, F–H** by R.D. Bull, **B–D** by L.J. Gillespie and **E** by P.C. Sokoloff.

***Arabidopsisarenicola* (Richardson ex Hook.) Al-Shehbaz, Elven, D.F.Murray & Warwick** (≡ *Arabisarenicola* (Richardson ex Hook.) Gelert) (Fig. 14A)—Arctic rockcress | North American (NE)

Previously recorded in Kimmirut (Polunin 1940; Aiken et al. 2007). Newly recorded in the park and from Pleasant Inlet. Scattered on Baffin Island, north to southern Bylot Island (*Drury 5491*, CAN 10051626) (Aiken et al. 2007). Elsewhere on southern Baffin Island, recorded from Dorset Island, Iqaluit, Jackman Sound (*Potter 8027*, GH 00974180) and Ukiurjak (formerly King Charles Cape) (Aiken et al. 2007; Saarela et al. 2020a).

**Katannilik Territorial Park**: *Saarela et al. 1963* (CAN) [MJ-11], *2222* (CAN, MT, UBC, US) [WR-4], *2329* (CAN, WIN) [LR-29], *2429* (CAN) [EC-7], *2560* (ALTA, CAN) [SF-7], *2570* (CAN) [SF-4]. **Kimmirut**: *Malte s.n.* [118878] (CAN, DAO, GH, MT, US) [KM-1]. **Pleasant Inlet**: *Saarela et al. 2705* (ALA, CAN, O) [PI-2].

##### *Arabis* L.

***Arabisalpina* L.**—Alpine rockcress | Amphi-Atlantic–European–Asian (W) & tropical mountains

Previously recorded in Kimmirut (Polunin 1940; Porsild and Cody 1980; Aiken et al. 2007). Newly recorded in the park. Elsewhere on Baffin Island, recorded from scattered sites north to Cape Searle, Cumberland Peninsula. Elsewhere on southern Baffin Island, recorded from Amadjuak Lake, Beekman Peninsula, Iqaluit, Nettilling Lake, Ogac Lake and Silliman’s Fossil Mount (Aiken et al. 2007).

**Katannilik Territorial Park**: *Saarela et al.* 2559 (CAN, NYBG, US) [SF-7]. **Kimmirut**: *Malte s.n.* [126873] (CAN, GH, V), *s.n.*/*1181* [121023] (CAN, 2 ex; GH), *Soper s.n.* (CAN, 3 ex), *Polunin 347*, *1973* (GH), *421* (CAN), *910* (US), *Dutilly 993* (DAO), *1004* (CAN, DAO), *1046* (CAN), *Oldenburg 97*, *100A* (GH) [KM-1], *Saarela et al. 2659* (ALA, ALTA, CAN, O, UBC) [KM-18].

**BrayaglabellaRichardsonsubsp.glabella**—Smooth northern rockcress | Amphi-Beringian–North American (N)

Previously recorded in Kimmirut (Aiken et al. 2007). We did not encounter the taxon in 2012. Elsewhere on Baffin Island, known from Iqaluit (Aiken et al. 2007). Aiken et al. (2007) erroneously mapped a record in the Clyde River area, based on Porsild and Cody’s (1980) map, which does not include a record from there.

**Kimmirut**: *Polunin 1121* (CAN) [KM-1]

**Brayaglabellasubsp.purpurascens (R.Br.) Cody** (≡ *B.purpurascens* R.Br.)—Purple rockcress | Circumpolar–Cordilleran

Previously recorded in Kimmirut (Polunin 1940; Aiken et al. 2007). Newly recorded in the park. Known from scattered sites across Baffin Island and elsewhere on southern Baffin Island, recorded from Iqaluit, Mallik Island and Silliman’s Fossil Mount (Aiken et al. 2007; Saarela et al. 2020a). A record Aiken et al. (2007) mapped on Resolution Island is an error; the collection (*Porsild 21552*, CAN 10053157) is from Iqaluit.

**Katannilik Territorial Park**: *Saarela et al. 2269* (CAN) [LR-23], *2544* (CAN) [SF-12], *2549* (CAN) [SF-9]. **Kimmirut**: *Malte s.n.*/*453* [120294] (CAN, GH, US), *Polunin 2327* (GH) [KM-1].

##### *Cardamine* L.

***Cardaminebellidifolia* L.**—Alpine bittercress | Circumpolar-alpine

Previously recorded in Kimmirut (Polunin 1940). Aiken et al. (2007) did not map Polunin’s collection. Newly recorded in the park. Widespread across Baffin Island and elsewhere on southern Baffin Island, recorded from Chorkbak Inlet, Dorset Island, Iqaluit, Jackman Sound (*Potter 8023*, GH 01098636), Ogac Lake, Resolution Island (*Wynne-Edwards 7223*, CAN 10054118) and York Sound (*Wynne-Edwards 7332*, CAN 10054119) (Aiken et al. 2007; Saarela et al. 2020a).

**Katannilik Territorial Park**: *Saarela et al. 2025* (CAN) [MJ-21], *2040* (CAN) [MJ-23], *2114* (CAN) [MJ-37], *2158* (CAN) [CR-5], *2312* (CAN) [LR-6]. **Kimmirut**: *Polunin 1243* (GH), *1408* (US) [KM-1], *Saarela et al. 2727* (CAN, O) [KM-5].

***Cardaminepolemonioides* Rouy** (= *C.nymanii* Gand., = C.pratensissubsp.angustifolia (Hook.) O.E.Schultz)—Nyman’s bittercress | Circumpolar

Previously recorded in Kimmirut, but we have not seen vouchers for Polunin’s 1935 and 1936 records (Polunin 1940). Newly recorded in the park. Known from scattered sites across Baffin Island and elsewhere on southern Baffin Island, recorded from “Aitken Lakes” (ca. 13 km west-northwest of Kinngait), Dorset and Mallik islands, Foxe Peninsula near Wildbird Islands, Iqaluit and Lower Savage Islands (*Wynne-Edwards 7278*, CAN 10054252) (Aiken et al. 2007; Saarela et al. 2020a).

**Katannilik Territorial Park**: *Saarela et al. 2494* (ALA, CAN) [EC-11], *2518* (CAN) [SF-23], *2597* (CAN, O) [TJ-1].

##### *Cochlearia* L.

***Cochleariagroenlandica* L.** (≡ C.officinalissubsp.groenlandica (L.) A.E.Porsild, = C.officinalissubsp.arctica (D.F.K.Schltdl.) Hultén)—Greenland scurvygrass | Circumpolar

Previously recorded in Kimmirut (Polunin 1940). Aiken et al. (2007) did not map Polunin’s Kimmirut collection. Newly recorded in the park. The species grew on the outer sandy floodplains of Tasiujarjuaq near Soper Falls with *Carexmaritima*, *Juncusarcticus*, J.triglumissubsp.albescens and *Salixarctophila* and in a moist mossy depression near the Kimmirut boat landing on Tasiujarjuaq. Widespread on Baffin Island and elsewhere on southern Baffin Island, recorded from Dorset and Mallik islands, Foxe Peninsula near Wildbird Islands, Iqaluit, Ogac Lake and at least two additional sites along Hudson Strait (Aiken et al. 2007; Saarela et al. 2020a).

**Katannilik Territorial Park**: *Saarela et al. 2526* (CAN) [SF-26], *2608* (CAN) [TJ-4]. **Kimmirut**: *Polunin 1100* (GH) [KM-1].

##### *Draba* L.

***Drabaalpina* L.**—Alpine draba | Amphi-Atlantic

Previously recorded in Kimmirut (Polunin 1940; Aiken et al. 2007). Malte collected the taxon in “wet ground among rocks at waterfall”, according to label data. Newly recorded in the park, where the species grew at the base of a hill (lower slopes igneous rock, upper white calcareous crystalline limestone) by a large meadow near Soper Falls with *Equisetumarvense* and *Salixherbacea*. Known from scattered sites across Baffin Island and elsewhere on southern Baffin Island, recorded from Bowdoin Harbour [Schooner Harbour] (*Soper s.n.*, CAN 10053698), Dorset and Mallik islands, Foxe Peninsula near Wildbird Islands (*Manning 260*, CAN 10053734, *Manning 229*, CAN 10053737) and Lower Savage Islands (Aiken et al. 2007; Saarela et al. 2020a).

**Kimmirut**: *Malte s.n.* [126869] (CAN), *s.n.* [126881] (CAN), *Oldenburg 93* (MIN) [KM-1]. **Katannilik Territorial Park**: *Saarela et al. 2553* (CAN) [SF-11].

***Drabaarctica* J.Vahl** (Fig. 14B)—Arctic draba | Probably Amphi-Atlantic

Newly recorded in the park, study area and southern Baffin Island. Near Mount Joy, the species grew on a steep, rocky slope with *Arctousalpina*, *Betulaglandulosa*, *Empetrumnigrum* and *Rhododendronlapponicum*. Near Soper Falls, it grew in a fine gravel band between a steep, mostly bare slope and a *Cassiope* snow patch community with *Erigeronhumilis*, *Poaalpina* and *Salixreticulata*. Otherwise known on Baffin Island from scattered east coast sites (Aiken et al. 2007).

**Katannilik Territorial Park**: *Saarela et al. 2048* (CAN) [MJ-23], *2509* (CAN) [SF-15].

***Drabacrassifolia* Graham**—Snowbed draba | Cordilleran & Amphi-Atlantic (W)

Previously recorded in Kimmirut (Polunin 1940; Aiken et al. 2007). Elsewhere on Baffin Island, recorded from Beekman Peninsula, Brewster Point, Coutts Inlet, Iqaluit (Apex area; known from a single collection), Inugsuin Fiord, Newell Sound and York Sound (Aiken et al. 2007; Polunin 1939). A record Aiken et al. (2007) mapped on the tip of the Cumberland Peninsula is an error; the collection (*McLaren 31*, CAN 10054987) is from the Beekman Peninsula. Elsewhere in the Canadian Arctic Archipelago, recorded on Southampton Island (Aiken et al. 2007).

**Kimmirut**: *Polunin 2311* (CAN) [KM-1].

***Drabafladnizensis* Wulfen**—Austrian draba | Circumpolar-alpine

Newly recorded in the park and the study area. Near Mount Joy, this species grew on rocky cliff edges with *Bistortavivipara*, *Carexnorvegica*, *Rhododendronlapponicum* and *Saxifragatricuspidata*. At “Panorama Falls”, it grew on a steep, rocky slope with *Arctousalpina*, *Betulaglandulosa*, *Empetrumnigrum* and *R.lapponicum*. Elsewhere on Baffin Island, recorded from ca. eight scattered sites (Aiken et al. 2007).

**Katannilik Territorial Park**: *Saarela et al. 2019a* (CAN) [MJ-42], *2045* (CAN), *2047* (CAN) [MJ-23].

***Drabaglabella* Pursh** (Fig. 14C)—Smooth draba | Circumboreal-polar

Previously recorded in Kimmirut and the park (Polunin 1940; Aiken et al. 2007). Newly recorded from Pleasant Inlet. Widespread across Baffin Island and elsewhere on southern Baffin Island, recorded from Dorset and Mallik islands, Foxe Peninsula near Wildbird Islands, Iqaluit, Ogac Lake and York Sound (Aiken et al. 2007; Saarela et al. 2020a).

**Katannilik Territorial Park**: *Soper s.n.* (DAO) [WR-1], *Aiken & Iles 02-060 a* (CAN) [LS-1], *Saarela et al. 2035* (CAN) [MJ-14], *2042* (CAN, UTC, UVIC) [MJ-23], *2214* (CAN, GH, MIN, O, QFA) [GC-8], *2268* (ASU, CAN, NFM, NYBG), *2272* (CAN) [LR-23], *2303* (CAN, WTU) [LR-18], *2365* (ALA, CAN, MT, O) [LR-9], *2508* (ALA, CAN, O) [SF-15], *2515* (CAN, MO, UBC, WIN) [SF-16]. **Kimmirut**: *Malte s.n.* [126864] (CAN, GH), *s.n.*/*451* [120292] (CAN, GH), *s.n.* [126849] (CAN, GH), *s.n.* [118917] (CAN), *s.n.* [118918] (CAN, GH), *s.n.*/*608* (CAN, GH), *Polunin 375* (CAN) [KM-1], *Saarela et al. 2662* (ALTA, CAN, US) [KM-18], *2734* (CAN) [KM-7], *2781* (CAN), *2786* (CAN) [KM-19]. **Pleasant Inlet**: *Saarela et al. 2726* (CAN) [PI-1].

***Drabalactea* Adams** (Fig. 14D)—Milky draba | Circumpolar-alpine

Newly recorded in the park and the study area. Not known from Kimmirut. Widespread across Baffin Island and elsewhere on southern Baffin Island, recorded from “Aitken Lakes” (ca. 13 km west-northwest of Kinngait), Dorset and Mallik islands, Foxe Peninsula near Wildbird Islands, Iqaluit, Lower Savage Islands, Ogac Lake, Resolution Island and York Sound (Aiken et al. 2007; Saarela et al. 2020a).

**Katannilik Territorial Park**: *Saarela et al. 2020* (CAN) [MJ-42], *2084* (CAN, MO, NYBG, UBC, UTC, UVIC, WTU), *2085* (CAN) [MJ-33], *2105* (ALTA, CAN, MICH) [MJ-36], *2310* (CAN) [LR-6], *2510* (CAN) [SF-15], *2568* (CAN, MT) [SF-8], *2635* (CAN, US, WIN) [TJ-3].

***Drabanivalis* Lilj.** (Fig. 14E)—Snow draba | Circumpolar-alpine

Previously recorded in Kimmirut and the park (Polunin 1940; Aiken et al. 2007). Widespread across Baffin Island and elsewhere on southern Baffin Island, recorded from Dorset and Mallik islands, Iqaluit, Ogac Lake, Resolution Island and York Sound (Aiken et al. 2007; Saarela et al. 2020a).

**Katannilik Territorial Park**: *Saarela et al. 2399* (ALA, CAN) [LC-3], *1993* (CAN) [MJ-41], *2019b* (CAN) [MJ-42], *2021* (CAN) [MJ-42], *2043* (CAN), *2046* (CAN) [MJ-23], *2052* (CAN), *2053* (CAN) [MJ-22], *2270* (CAN) [LR-23], *2333* (CAN) [LR-29], *2542* (CAN), *2547* (CAN) [SF-12], *2556* (CAN) [SF-11]. **Kimmirut**: *Malte s.n.* [118925] (CAN) [KM-1], *Saarela et al. 2730* (CAN) [KM-5].

##### *Eutrema* R.Br.

***Eutremaedwardsii* R.Br.**—Edward’s eutrema | Circumpolar-alpine

Previously recorded in Kimmirut and the park (Polunin 1940; Porsild and Cody 1980), but Aiken et al. (2007) did not map these records. Known from scattered sites across Baffin Island and elsewhere on southern Baffin Island, recorded from Chorkbak Inlet (*Carroll s.n.*, CAN 10057728), Dorset and Mallik islands and Iqaluit (Aiken et al. 2007; Saarela et al. 2020a).

**Katannilik Territorial Park**: *Soper s.n.* (CAN) [SF-1], *s.n.* (CAN) [WR-1], *Saarela et al. 2004* (ALA, CAN, MT) [MJ-18], *2055* (CAN, US) [MJ-17], *2066* (CAN) [MJ-30], *2088* (CAN) [MJ-33], *2098* (CAN, O, WIN) [MJ-35], *2313* (CAN) [LR-6], *2735* (CAN) [KM-7]. **Kimmirut**: *Dutilly 9096* (QFA) [KM-1].

##### *Physaria* (Nutt.) A.Gray

***Physariaarctica* (Wormsk. ex Hornem.) O’Kane & Al-Shehbaz** (≡ *Lesquerellaarctica* (Wormsk. ex Hornem.) S.Watson)—Arctic bladderpod | Asian (N)–Amphi-Beringian–North American (N)

Previously recorded in Kimmirut (Polunin 1940; Aiken et al. 2007). Newly recorded in the park. Polunin (1940) remarked that this species grew “plentifully if very locally at Lake Harbour…”; we did not encounter it there in 2012. Known from scattered sites across Baffin Island and elsewhere on southern Baffin Island, recorded from Silliman’s Fossil Mount (Aiken et al. 2007).

**Katannilik Territorial Park**: *Saarela et al. 2271* (CAN) [LR-23], *2543* (ALA, CAN, MT, O, WIN) [SF-12]. **Kimmirut**: *Malte s.n.* [118935] (CAN, GH), *s.n.*/*1180* [121022] (CAN, GH, MT, V), *Oldenburg 117* (GH) [KM-1], *Archambault AA274* (CAN) [KM-3], *Saarela et al. 2732* (CAN) [KM-5].

**Caryophyllales**

#### Plumbaginaceae

##### *Armeria* Willd.

***Armeriascabra* Pall. ex Roem. & Schult.** (≡ A.maritimasubsp.sibirica (Turcz. ex Boiss.) Nyman)—Sea thrift | Circumpolar

Previously recorded in Kimmirut (Polunin 1940; Aiken et al. 2007). Newly recorded in the park. Near Mount Joy, this species grew on a sparsely vegetated, stony floodplain with *Artemisiaborealis*, *Carexbigelowii*, *C.nardina*, *C.rupestris*, *Chamaenerionlatifolium* and *Sileneacaulis.* At the Soper and Livingstone rivers’ confluence, it grew in a lush meadow with *Anthoxanthummonticola*, *Arctousalpina*, *Astragalusalpinus*, *Betulaglandulosa*, *Oxytropismaydelliana* and *Pyrolagrandiflora*. Widespread across Baffin Island and elsewhere on southern Baffin Island, recorded from Amadjuak Bay, between Amadjuak Bay and Chorkbak Inlet, Dorset and Mallik islands, Iqaluit, Perry Bay (*Jotcham s.n.*, CAN 10079155) and Ukiurjak (formerly King Charles Cape) (Aiken et al. 2007; Saarela et al. 2020a).

**Katannilik Territorial Park**: *Saarela et al. 1964* (CAN, MO, UBC, US, WIN) [MJ-11], *2354* (ALA, CAN, O) [LR-28]. **Kimmirut**: *Malte s.n.* [119108] (CAN), *s.n.* [119110] (CAN, GH), *s.n.* [119113] (CAN, GH, US), *Polunin 351* (CAN, GH), *Dutilly 1027* (QFA) [KM-1].

#### Polygonaceae

##### *Bistorta* (L.) Scop.

***Bistortavivipara* (L.) Delarbre** (≡ *Polygonumviviparum* L.)—Alpine bistort | Circumboreal-polar

Previously recorded in Kimmirut (Polunin 1940; Porsild 1957, 1964; Porsild and Cody 1980; Aiken et al. 2007). Newly recorded in the park. Widespread across Baffin Island and elsewhere on southern Baffin Island, recorded from Amadjuak Bay, Chorkbak Inlet, Dorset and Mallik islands, Foxe Peninsula near Wildbird Islands, Iqaluit, Ogac Lake, Perry Bay, Resolution Island, Ukiurjak (formerly King Charles Cape) and York Sound (*Wynne-Edwards 7326*, CAN 10033070) (Aiken et al. 2007; Saarela et al. 2020a).

**Katannilik Territorial Park**: *Saarela et al. 2180* (CAN, O) [GC-4], *2539* (ALA, CAN) [SF-25], *1997* (CAN, MT, WIN) [MJ-12], *2038* (ALA, CAN, O, WLU) [MJ-44]. **Kimmirut**: *Malte s.n.* [126893] (CAN), *s.n.* [118721] (CAN), *Oldenburg 79* (MIN), *Soper s.n.* (CAN), *Dutilly 1002* (QFA) [KM-1], *Johansen 1115* (C) [KM-20], *Archambault AA252* (CAN) [KM-4].

##### *Koenigia* L.

***Koenigiaislandica* L.** (Fig. 14F)—Iceland purslane | Circumpolar-alpine

Previously recorded in Kimmirut (Polunin 1940; Porsild and Cody 1980; Aiken et al. 2007). Newly recorded in the park. This diminutive, annual species grew on a muddy river flat ca. 0.5 km south of group/warden cabin #7 with *Salixarctica*, on sandy flats near Soper Falls with *Artemisiaborealis*, *Chamaenerionlatifolium*, *Eriophorumscheuchzeri*, *Juncusarcticus* and J.triglumissubsp.albescens and in Kimmirut in a grassy delta below the garbage dump. Widespread across Baffin Island and elsewhere on southern Baffin Island, recorded from Dorset and Mallik islands, Iqaluit, Lower Savage Islands and Resolution Island (Aiken et al. 2007; Saarela et al. 2020a).

**Katannilik Territorial Park**: *Saarela et al. 2180* (CAN, O) [GC-4], *2539* (ALA, CAN) [SF-25]. **Kimmirut**: *Polunin 1141* (CAN) [KM-1], *Saarela et al. 2770* (CAN, MT, UBC, WIN, WLU) [KM-16].

##### *Oxyria* Hill

***Oxyriadigyna* Hill**—Mountain sorrel, alpine sorrel | Circumpolar-alpine

Previously recorded in Kimmirut (Polunin 1940; Porsild and Cody 1980; Aiken et al. 2007). Newly recorded in the park and from Pleasant Inlet. Widespread across Baffin Island and elsewhere on southern Baffin Island, recorded from Amadjuak Bay, Chorkbak Inlet, Dorset and Mallik islands, Foxe Peninsula near Wildbird Islands, Iqaluit, Lower Savage Islands, Perry Bay (*Jotcham s.n.*, ACAD- ECS004504, CAN 10035611, QFA-210571), Resolution Island and Ukiurjak (formerly King Charles Cape) (Aiken et al. 2007; Saarela et al. 2020a).

**Katannilik Territorial Park**: *Saarela et al. 1990* (ALA, CAN, MT, O, WIN) [MJ-40], *2080* (CAN, US, WLU) [MJ-33]. **Kimmirut**: *Malte s.n.* [120298] (CAN, GH), *s.n.* [118714] (CAN), *Oldenburg 105* (MIN, 2 ex), *Soper s.n.* (CAN), *Dutilly 1048* (QFA), *9085* (QFA) [KM-1], *Johansen 1116* (C) [KM-20]. **Pleasant Inlet**: *Saarela et al. 2724* (CAN, UBC) [PI-1].

#### Caryophyllaceae

##### *Arenaria* L.

***Arenariahumifusa* Wahlenb.**—Creeping sandwort | North American (N)–Amphi-Atlantic

Previously recorded in Kimmirut (Polunin 1940; Aiken et al. 2007). Newly recorded in the park and from Pleasant Inlet. Known from scattered sites across Baffin Island and elsewhere on southern Baffin Island, recorded from Dorset and Mallik islands, Iqaluit and Ogac Lake (Aiken et al. 2007; Saarela et al. 2020a).

**Katannilik Territorial Park**: *Saarela et al. 2477* (CAN) [EC-15]. **Kimmirut**: *Malte s.n.* [121009] (CAN) [KM-1]. **Pleasant Inlet**: *Saarela et al. 2725* (CAN) [PI-1].

***Arenarialongipedunculata* Hultén**—Long-stemmed sandwort | Amphi-Beringian–Cordilleran–North American (N)?

Newly recorded for the park, study area, Canadian Arctic Archipelago and Nunavut. Gillespie et al. (2015) provide details.

**Katannilik Territorial Park**: *Saarela et al. 2776* (CAN) [KM-19].

##### *Cerastium* L.

***Cerastiumalpinum* L.** (= C.alpinumsubsp.lanatum (Lam.) Ces.)—Alpine chickweed | Amphi-Atlantic (W)

The *Cerastiumalpinum* aggregate, including *C.arcticum*, is a taxonomically complicated polyploid group (Elven et al. 2011). Earlier treatments did not distinguish *C.alpinum* and *C.arcticum* as currently understood (Brysting and Elven 2000; Elven et al. 2011). *Cerastiumalpinum* s.str. has been recorded in Kimmirut and the park (Aiken et al. 2007). Widespread on Baffin Island and elsewhere on southern Baffin Island, recorded from Amadjuak Bay, Dorset and Mallik islands, Iqaluit, Lower Savage Islands, Ogac Lake and York Sound (*Wynne-Edwards 7318*, CAN 10018153) (Aiken et al. 2007; Saarela et al. 2020a).

**Katannilik Territorial Park**: *Soper s.n.* (CAN) [WR-1], *Saarela et al. 1962* (CAN, MICH, QFA, WLU) [MJ-11], *1989* (CAN, GH) [MJ-40], *2390* (ALA, ALTA, CAN, GH, MO, MT, NYBG, O, UBC, WIN) [LR-29], *2634* (CAN, US, UTC, UVIC, WTU) [TJ-3]. **Kimmirut**: *Malte s.n.* [126896] (CAN), *s.n.* [126858] (CAN, GH, TRT), *s.n.* [118747] (CAN), *Soper s.n.* (CAN), *Dutilly 1001* (GH) [KM-1].

***Cerastiumarcticum* Lange**—Arctic mouse-ear chickweed | North American (N)–Amphi-Atlantic–European (N)

Previously recorded in Kimmirut (Aiken et al. 2007). We did not collect the taxon in 2012. Widespread on Baffin Island and elsewhere on southern Baffin Island, recorded from Chorkbak Inlet, Dorset Island, Foxe Peninsula near Wildbird Islands, Iqaluit, Lower Savage Islands, Ogac Lake and Resolution Island (Aiken et al. 2007; Saarela et al. 2020a).

**Kimmirut**: *Malte s.n.* [126899] (CAN, GH) [KM-1].

##### *Cherleria* L.

***Cherleriabiflora* (L.) A.J.Moore & Dillenb.** (≡ *Minuartiabiflora* L.Schinz & Thell.; = *Arenariasajanensis* Willd. ex D.F.K.Schltdl.)—Mountain stitchwort | Circumpolar-alpine

Previously recorded in Kimmirut (Polunin 1940; Porsild 1957, 1964; Porsild and Cody 1980; Aiken et al. 2007). Newly recorded in the park. Widespread on Baffin Island and elsewhere on southern Baffin Island, recorded from Dorset and Mallik islands, Iqaluit, Lower Savage Islands, Newell Sound, Ogac Lake, Resolution Island and York Sound (*Wynne-Edwards 7308*, CAN 10046757) (Aiken et al. 2007; Saarela et al. 2020a).

**Katannilik Territorial Park**: *Saarela et al. 2213* (ALA, CAN, O) [GC-8], *2432* (CAN, GH) [EC-7]. **Kimmirut**: *Dutilly 1099* (CAN), *Polunin 2325* (CAN), *2309* (US), *2640* (GH) [KM-1].

##### *Honckenya* Ehrh.

**Honckenyapeploidessubsp.diffusa (Hornem.) Hultén** (≡ Arenariapeploidesvar.diffusa Hornem.)—Seabeach sandwort | Circumpolar

Newly recorded in the park, Pleasant Inlet and study area. The species grew on sandy seashores. Widespread on Baffin Island and elsewhere on southern Baffin Island, known from Brewster Point (*Potter 8212*, GH 01744527, MT00056276), Cormack Bay, Dorset and Mallik islands, Iqaluit, Jackman Sound (*Potter 8213*, GH-01744528) and Ogac Lake (Aiken et al. 2007; Saarela et al. 2020a).

**Katannilik Territorial Park**: *Saarela et al. 2581* (CAN, GH, QFA) [SF-17], *2611* (CAN) [TJ-6]. **Pleasant Inlet**: *Saarela et al. 2686* (CAN, MO, NFM, NYBG, US, UVIC) [PI-3], *2708* (ALA, CAN, MT, O, UBC, WIN) [PI-2].

##### *Sabulina* Rchb.

***Sabulinarossii* (R.Br. ex Richardson) Dillenb. & Kadereit** (≡ *Minuartiarossii* (R.Br. ex Richardson) Graebn.)—Ross’s stitchwort | Amphi-Beringian (E)–North American (N)–Amphi-Atlantic (W)

Newly recorded in the park and study area. Known from scattered collections on Baffin Island, most on the west side and elsewhere on southern Baffin Island, recorded from Dorset Island and Iqaluit (Aiken et al. 2007; Saarela et al. 2020a). We are unaware of confirmed vouchers from Iqaluit. A record Aiken et al. (2007) mapped from Lower Savage Islands (*Gillespie et al. 6726*, CAN 1004679) has been re-determined as *Cherleriabiflora*.

**Katannilik Territorial Park**: *Saarela et al. 2276* (CAN) [LR-25], *2485* (CAN) [EC-16], *2527* (CAN) [SF-26].

***Sabulinarubella* (Wahlenb.) Dillenb. & Kadereit** (≡ *Arenariarubella* (Wahlenb.) Sm., ≡ *Minuartiarubella* (Wahlenb.) Hiern)—Reddish stitchwort | Circumpolar-alpine

Previously recorded in Kimmirut (Polunin 1940; Porsild 1957, 1964; Porsild and Cody 1980; Aiken et al. 2007). Newly recorded in the park. Widespread on Baffin Island and elsewhere on southern Baffin Island, recorded from Dorset and Mallik islands, Iqaluit, Newell Sound (*McLaren 46*, CAN 10044202) and Ogac Lake (Aiken et al. 2007; Saarela et al. 2020a).

**Katannilik Territorial Park**: *Saarela et al. 1982* (CAN) [MJ-11], *2051* (CAN) [MJ-2], *2115* (ALTA, CAN, MO, MT, NYBG, UBC) [MJ-39], *2330* (ALA, CAN, GH, O, WIN) [LR-29], *2364* (CAN) [LR-8], *2513* (CAN) [SF-15], *2546* (CAN) [SF-12], *2555* (CAN) [SF-11], *2583* (CAN) [SF-20]. **Kimmirut**: *Malte s.n.* [126850] (CAN, GH), *s.n.* [126897] (CAN, GH), *s.n.* [121021] (CAN, GH, MT) [KM-1], *Archambault AA291* (CAN) [KM-3], *Saarela et al. 2729* (CAN) [KM-5].

***Sabulinastricta* (Sw.) Rchb.** (≡ *Minuartiastricta* (Sw.) Hiern; = *Arenariauliginosa* Schleich. ex Lam. & DC.)—Bog stitchwort | Circumpolar-alpine

Previously recorded in Kimmirut (Polunin 1940; Aiken et al. 2007). Newly recorded in the park. The species grew on a ridge near Livingstone River with *Cassiopetetragona*, *Dryasintegrifolia*, *Rhododendronlapponicum* and *Salixreticulata*. Widespread, but scattered on Baffin Island and elsewhere on southern Baffin Island, recorded from Beekman Peninsula, Dorset and Mallik islands, Iqaluit and Silliman’s Fossil Mount (Aiken et al. 2007; Saarela et al. 2020a).

**Katannilik Territorial Park**: *Saarela et al. 2267* (CAN) [LR-23]. **Kimmirut**: *Polunin 1166* (GH), *2342* (GH, CAN) [KM-1].

##### *Sagina* L.

**Saginanodosasubsp.borealis G.E.Crow** (= S.nodosaf.bulbillosa Polunin)—Northern knotted pearlwort | North American (N)–Amphi-Atlantic–European (N)–Asian (N)

Previously recorded in Kimmirut (Polunin 1940; Crow 1978; Porsild and Cody 1980; Aiken et al. 2007). Polunin’s collection is the only Baffin Island record. We did not encounter this taxon in 2012. This a shoreline plant that grows in rock crevices, wet gravel and sand and in moss tufts along rocky coasts (Crow 1978). Elsewhere in the Canadian Arctic Archipelago, known from Coats Island (Aiken et al. 2007). The specimen at CAN is the holotype of the name Saginanodosaf.bulbillosa Polunin.

**Kimmirut**: *Polunin 2312* (CAN, MO) [KM-1].

##### *Silene* L., nom. cons.

***Sileneacaulis* (L.) Jacq.**—Moss campion | Amphi-Beringian–North American–Amphi-Atlantic–European (N/C)–Asian (NW)

Previously recorded in Kimmirut (Porsild 1957, 1964; Porsild and Cody 1980; Aiken et al. 2007). Newly recorded in the park. Widespread on Baffin Island and elsewhere on southern Baffin Island, recorded from Bowdoin Harbour [Schooner Harbour] (*Robinson 11*, GH 01751207), Chorkbak Inlet, Dorset and Mallik islands, Foxe Peninsula near Wildbird Islands, Iqaluit, Lower Savage Islands, Perry Bay (*Jotcham s.n.*, ACAD- ECS004523) and Resolution Island (*Potter 8121*, GH 01751210) (Aiken et al. 2007; Saarela et al. 2020a).

**Katannilik Territorial Park**: *Saarela et al. 1987* (CAN, GH) [MJ-40], *2431* (ALA, CAN, O, WIN) [EC-7]. **Kimmirut**: *Malte s.n.* [126855] (CAN, GH), *s.n.* [126875] (CAN, GH), *s.n.* [126903] (CAN, GH), *s.n.* [118761] (CAN, MT), *Johansen 1119* (C) [KM-20].

***Sileneinvolucrata* (Cham. & Schltdl.) Bocquet** (= *Melandriumaffine* (J.Vahl ex Fr.) J.Vahl)—Arctic catchfly | Circumpolar-alpine

Previously recorded in Kimmirut (Porsild 1957, 1964; Porsild and Cody 1980; Aiken et al. 2007). Newly recorded in the park and Pleasant Inlet. Widespread on Baffin Island and elsewhere on southern Baffin Island, recorded from Amadjuak Lake, Brewster Point (*Wynne-Edwards 7366*, CAN 10045065), Dorset and Mallik islands, Foxe Peninsula, Ogac Lake and Ward Inlet (*Freeman s.n.*, US03631086) (Aiken et al. 2007; Saarela et al. 2020a). Researchers have recognized multiple subspecies, but taxonomic concepts differ amongst contemporary treatments (Morton 2005; Elven et al. 2011). Pending the resolution of this problem, we do not recognise infraspecific taxa.

**Katannilik Territorial Park**: *Saarela et al. 1981* (CAN) [MJ-11], *2037* (CAN, NYBG) [MJ-44], *2099* (CAN) [MJ-36], *2168* (ALTA, CAN, WIN) [CR-4], *2245* (CAN, MO, O, UBC, US) [WR-9]. **Kimmirut**: *Dutilly 1025* (CAN, 2 ex), *Malte s.n.* [126852] (CAN, GH), *s.n.* [118767] (CAN), *s.n.*/*464* [120305] (CAN, GH), *s.n.*/*642* [118768] (CAN, GH) [KM-1], *Oldenburg 91* (MIN), *Polunin 434* (GH) [KM-1]. **Pleasant Inlet**: *Saarela et al. 2682* (CAN) [PI-3].

**Sileneuralensissubsp.arctica (Fr.) Bocquet** (≡ Melandriumapetalumsubsp.arcticum (Fr.) Hultén)—Arctic nodding catchfly | Circumpolar

We follow the *Sileneuralensis* taxonomy that Elven et al. (2011) proposed, which differs from Morton (2005), who included the two subspecies recognized here plus a third one in a broadly circumscribed S.uralensissubsp.uralensis. Aiken et al. (2007) recognized all Canadian Arctic Archipelago plants as S.uralensissubsp.arctica, which they mapped in the study area.

**Kimmirut**: *Malte s.n.* [126884] (CAN), *s.n.* [118771] (CAN) [KM-1].

**Sileneuralensis(Rupr.)Bocquetsubsp.uralensis** (= *Melandriumapetalum* (L.) Fenzl)—Nodding catchfly | European (NE)–Asian (N)–Amphi-Beringian–North American (N)

Newly recorded in the park and study area. See taxonomic comments under the previous taxon.

**Katannilik Territorial Park**: *Saarela et al. 2266* (CAN) [LR-23], *2475* (CAN) [EC-15], *2484* (CAN, O) [EcC-16], *2582* (ALA, ALTA, CAN, GH, MT, WIN) [SF-20].

##### *Stellaria* L., nom. cons.

***Stellariahumifusa* Rottb.**—Salt-marsh starwort | Circumpolar–Amphi-Pacific

Previously recorded in Kimmirut (Polunin 1940; Porsild 1957, 1964; Porsild and Cody 1980; Aiken et al. 2007). Newly recorded in the park and from Pleasant Inlet. Widespread on Baffin Island and elsewhere on southern Baffin Island, known from Dorset and Mallik islands, Iqaluit and Ogac Lake (Aiken et al. 2007; Saarela et al. 2020a).

**Katannilik Territorial Park**: *Saarela et al. 2609* (ALA, CAN, GH, MT, O) [TJ-6]. **Kimmirut**: *Malte s.n.* [126851] (CAN, GH), *s.n.* [126892] (CAN, GH), *s.n.* [118786] (CAN) [KM-1], *Saarela et al. 2767* (CAN, US, WIN) [KM-16]. **Pleasant Inlet**: *Saarela et al. 2685* (CAN, UBC) [PI-3].

***Stellarialongipes* Goldie** (= *S.arenicola* Raup, = *S.crassipes* Hultén, = *S.edwardsii* R.Br., = *S.laeta* Richardson, = *S.monantha* Hultén, = *S.stricta* Richardson, = *S.subvestita* Greene)—Long-stalked starwort | Circumboreal-polar

Previously recorded in Kimmirut and the park (Polunin 1940; Porsild 1957, 1964; Porsild and Cody 1980; Aiken et al. 2007). Widespread on Baffin Island and elsewhere on southern Baffin Island, known from Dorset and Mallik islands, Foxe Peninsula near Wildbird Islands (*Manning 264*, CAN 10049954), Iqaluit, Nuwata (*Manning 220*, CAN 10049956), Perry Bay, Resolution Island (*Wynne*-*Edwards 7228*, CAN 10049380) and York Sound (*Wynne-Edwards 7307*, CAN 10049379) (Aiken et al. 2007; Saarela et al. 2020a).

**Katannilik Territorial Park**: *Soper s.n.* (CAN) [WR-1], *Saarela et al. 1971* (ALA, CAN, O, WIN) [MJ-8], *2073* (CAN) [MJ-43], *2206* (CAN, MICH, MO, NYBG, US) [GC-9], *2447* (ALTA, CAN, MT, UBC, UVIC) [EC-8]. **Kimmirut**: *Malte s.n.* [126892] (CAN, GH), *s.n.* [118788] (CAN, MT) [KM-1], *Johansen 1118* (C) [KM-20], *Saarela et al. 2790* (CAN, GH) [KM-19].

##### *Viscaria* Bernh., nom. cons.

***Viscariaalpina* (L.) G.Don** (≡ *Lychnisalpina* L.; = *Silenesuecica* (Lodd.) Greuter & Burdet) (Fig. 14G, H)—Alpine catchfly | Amphi-Atlantic–European (N/C)

Newly recorded in the park and study area. At a site 2 km south of Emergency Cabin 8, this species grew in a *Betula*-*Salix* thicket on a steep, east-facing slope with *Cassiopetetragona*, *Dryasintegrifolia*, *Salixreticulata* and *Vacciniumvitis-idaea*. Above the Willow River, it was locally common on a south-facing slope near a large *Salixplanifolia* stand in which the tallest shrubs reached 11 ft [3.4 m] with *Arctousalpina*, *Betulaglandulosa*, *Calamagrostiscanadensis*, *Empetrumnigrum*, *Festucabrachyphylla*, *Luzulaspicata* and *Stellarialongipes*. Elsewhere on Baffin Island, recorded from Newell Sound (*McLaren 38*, CAN 10046536) and Ogac Lake (Aiken et al. 2007). Not known elsewhere in the Canadian Arctic Archipelago.

**Katannilik Territorial Park**: *Saarela et al. 2487* (ALA, CAN, O) [EC-17], *2241* (ALTA, CAN, MO, MT, NYBG, US, WIN) [WR-7].

#### Montiaceae

##### *Montia* L.

**Figure 15.**
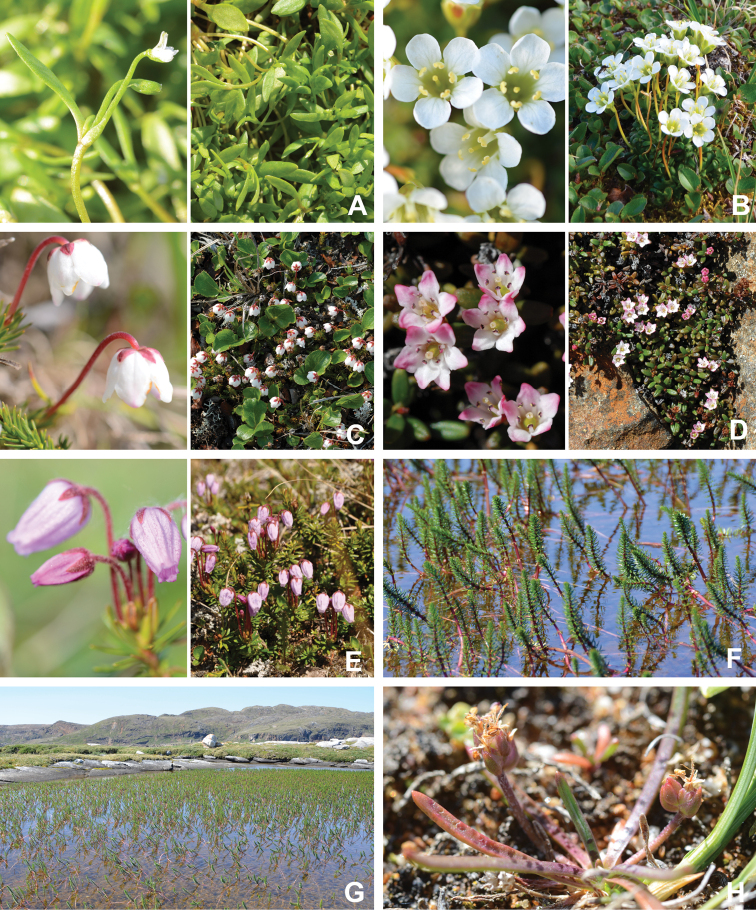
**A***Montiafontana* inflorescence (left) and habit (right), *Saarela et al. 2769***B***Diapensia* a inflorescences (left) and habit (right), *Saarela et al. 2161***C***Harrimanellahypnoides* inflorescences (left) and habit (right), *Saarela et al. 2417***D***Kalmiaprocumbens* inflorescences (left) and habit (right), *Saarela et al. 2562***E***Phyllodocecaerulea* inflorescences (left) and habit (right), *Saarela et al. 2146***F***Hippurisvulgaris* habit, *Saarela et al. 2604***G***Hippurisvulgaris* habitat, *Saarela et al. 2604*. **H***Plantagomaritima* habit, *Saarela et al. 2684*. Photos **A, B, C** left, **E** left, **F–H** by R.D. Bull, **C** right, **D** by L.J. Gillespie and **E** right by P.C. Sokoloff.

***Montiafontana* L.** (= *M.lamprosperma* Cham.) (Fig. 15A)—Water blinks | North American (NE)–Amphi-Atlantic–European & Amphi-Pacific/Beringian

Newly recorded in Kimmirut and the study area. This species grew below the Kimmirut garbage dump in a sewage-enriched, grassy delta above the high tide line with *Carexbicolor*, *Koenigiaislandica*, Potentillaanserinasubsp.groenlandica, Puccinelliaphryganodessubsp.neoarctica and P.tenellasubsp.langeana. Plants were abundant and large due to the nutrient-rich environment. Elsewhere on Baffin Island, recorded from Brewster Point (*Potter 8214*, MT00071440 det. J.M. Miller, GH 01860434, GH 01860404), Cormack Bay, Dorset Island, Great Plain of the Koukdjuak (*Boles et al. RB00-221*, CAN 10036137), Iqaluit and Newell Sound (*McLaren 57*, CAN 10036133) (Porsild and Cody 1980; Aiken et al. 2007; Saarela et al. 2020a). Porsild and Cody (1980) also mapped a record on the Cumberland Peninsula for which we are unaware of a voucher specimen. A site Aiken et al. (2007) mapped west of the study area, based on Porsild and Cody’s (1980) map, is an error.

**Kimmirut**: *Saarela et al. 2769* (ALA, ALTA, CAN, GH, MIN, MT, NYBG, O, QFA, UBC, US, UVIC, WIN, WLU) [KM-16].

#### Primulaceae

##### *Primula* L.

***Primulaegaliksensis* Wormskj.**—Greenland primrose | Amphi-Beringian–North American (N)–Amphi-Atlantic (W)

Our collections are the first records for Kimmirut, the study area, Baffin Island and the Canadian Arctic Archipelago. Gillespie et al. (2015) provide details.

**Kimmirut**: *Saarela et al. 2606* (CAN, US) [TJ-4], *2640* (CAN) [TJ-3].

#### Diapensiaceae

##### *Diapensia* L.

***Diapensialapponica* L.** (Fig. 15B)—Lapland diapensia | North American (NE)–Amphi-Atlantic–European (N)–Asian (NW)

Previously recorded in Kimmirut and the park (Polunin 1940; Porsild 1957, 1964; Porsild and Cody 1980; Aiken et al. 2007). Newly recorded from Pleasant Inlet. On Baffin Island, recorded at scattered sites north to the Isortoq River and Clyde Inlet and elsewhere on southern Baffin Island, recorded from Dorset Island, Iqaluit and sites along Hudson Strait west of the study area for which we are unaware of vouchers (Porsild and Cody 1980; Aiken et al. 2007; Saarela et al. 2020a). Elsewhere in the Canadian Arctic, recorded on Mansel and Southampton islands (Aiken et al. 2007).

**Katannilik Territorial Park**: *Soper s.n.* (CAN) [SF-1], *s.n.* (CAN) [WR-1], *Aiken & Iles 02-063* (CAN) [SF-2], *Saarela et al. 1974* (ALA, CAN, MT, O) [MJ-8], *1984* (CAN, UBC, US, WIN) [MJ-13], *2161* (ALTA, CAN, MO, WTU) [CR-2], *2423* (CAN, NFM, NYBG) [EC-7]. **Kimmirut**: *Malte s.n.* [119150] (CAN), *s.n.* [119132] (CAN), *s.n.* [121042] (CAN) [KM-1], *Johansen 1134* (C) [KM-20]. **Pleasant Inlet**: *Saarela et al. 2675* (CAN, WIN) [PI-3].

#### Ericaceae

##### *Andromeda* L.

***Andromedapolifolia* L.**—Bog rosemary | Circumboreal-polar

Newly recorded from the park, study area and Baffin Island. Gillespie et al. (2015) provide details.

**Katannilik Territorial Park**: *Saarela et al. 2186* (ALA, CAN, MO, MT, O, US, WIN) [GC-3].

##### *Arctous* (A.Gray) Nied.

***Arctousalpina* (L.) Nied.** (≡ *Arctostaphylosalpina* (L.) Spreng.)—Alpine bearberry | Circumpolar-alpine

Previously recorded in Kimmirut (Polunin 1940; Aiken et al. 2007). Newly recorded in the park. On Baffin Island, recorded at scattered sites north to Longstaff Bluff and Inugsuin Fiord and elsewhere on southern Baffin Island, recorded from Amadjuak Bay, Dorset and Mallik islands, Iqaluit, Ogac Lake, Pritzler Harbour, Resolution Island (*Potter 8153*, GH 01536684), Ukiurjak (formerly King Charles Cape) and York Sound (*Wynne-Edwards 73345*, CAN 10075753) (Aiken et al. 2007; Saarela et al. 2020a).

**Katannilik Territorial Park**: *Saarela et al. 1975* (ALA, CAN, MT, O, WIN) [MJ-8], *1995* (CAN) [MJ-41], *2418* (ALTA, CAN, MO, UBC, UVIC) [EC-7]. **Kimmirut**: *Malte s.n.* [119054] (US), *s.n.* [126894] (CAN, GH), *s.n.* [119051] (CAN), *s.n.* [119052] (CAN, GH), *s.n.* [119053] (CAN, GH), *Soper s.n.* (CAN), *Polunin 324* (CAN) [KM-1], *Johansen 1127* (C) [KM-20].

##### *Cassiope* D.Don

**Cassiopetetragona(L.)D.Donsubsp.tetragona**—Arctic heather | Circumpolar-alpine

Previously recorded in Kimmirut and the park (Polunin 1940; Porsild 1957, 1964; Porsild and Cody 1980; Aiken et al. 2007). Newly recorded from Pleasant Inlet. Widespread across Baffin Island and elsewhere on southern Baffin Island, recorded from Amadjuak Bay, Chorkbak Inlet, Dorset and Mallik islands, Iqaluit, Lower Savage Islands, Ogac Lake, Resolution Island (*Wynne-Edwards 7243*, CAN 10076133), Ukiurjak (formerly King Charles Cape) (*Baldwin 1871*, CAN 10074669) and York Sound (*Walker 827*, US 02992156) (Aiken et al. 2007; Saarela et al. 2020a).

**Katannilik Territorial Park**: *Soper s.n.* (CAN) [WR-1], *Saarela et al. 1940* (ALTA, CAN, MO, UBC, US, UVIC, WTU) [MJ-5]. **Kimmirut**: *Malte s.n.* [126866] (CAN, S), *Dutilly 1058*, *9131* (QFA), *1485* (MT) [KM-1], *Johansen 1132* (O) [KM-20]. **Pleasant Inlet**: *Saarela et al.* 2678 (ALA, CAN, MT, O, WIN) [PI-3].

##### *Empetrum* L.

***Empetrumnigrum* L.**—Crowberry | Circumboreal-polar

Previously recorded in Kimmirut and the park (Polunin 1940; Porsild and Cody 1980; Aiken et al. 2007). Newly recorded from Pleasant Inlet. Widespread across Baffin Island and elsewhere on southern Baffin Island, recorded from Amadjuak Bay, Dorset and Mallik islands, Iqaluit, Perry Bay and Resolution Island (*Potter 7517*, GH 01562721) (Aiken et al. 2007; Saarela et al. 2020a).

**Katannilik Territorial Park**: *Soper s.n.* (CAN, 2 ex) [WR-1], *Saarela et al. 1973* (CAN, NYBG, US) [MJ-8]. **Kimmirut**: *Malte s.n.* [119118] (CAN), *s.n.* [119120] (CAN), *s.n.* [119119] (CAN) [KM-1], *Johansen 1117* (C) [KM-20]. **Pleasant Inlet**: *Saarela et al. 2702* (ALA, ALTA, CAN, MT, O, WIN) [PI-2].

##### *Harrimanella* Coville

***Harrimanellahypnoides* (L.) Coville** (≡ *Cassiopehypnoides* (L.) D.Don) (Fig. 15C)—Moss heather | North American (NE)–Amphi-Atlantic–European (N)–Asian (NW)

Previously recorded in Kimmirut (Polunin 1940; Porsild 1957, 1964; Porsild and Cody 1980; Aiken et al. 2007). Newly recorded in the park and from Pleasant Inlet. On Baffin Island, recorded at scattered sites as far north as Ekalugad Fiord and elsewhere on southern Baffin Island, recorded from Dorset and Mallik islands, Iqaluit, Jackman Sound (*Potter 8151*, GH 01593315), Lower Savage Islands, Ogac Lake, Resolution Island (*Potter 8150*, GH 01593275) and Silliman’s Fossil Mount (Aiken et al. 2007; Saarela et al. 2020a). Elsewhere in the Canadian Arctic Archipelago, recorded on Mansel and Salisbury islands (Aiken et al. 2007).

**Katannilik Territorial Park**: *Saarela et al. 2033* (CAN, MO, UBC, UTC, UVIC, WTU) [MJ-14], *2159* (CAN, US) [CR-3], *2417* (CAN, O) [EC-7], *2566* (ALA, CAN, MT, WIN) [SF-5], **Kimmirut**: *Malte s.n.* [126886] (CAN, GH, UTC), *s.n.* [119039] (CAN), *s.n.*/*1196* [121038] (CAN, GH, QFA, S, US), *Polunin 1119* (GH) [KM-1], *Johansen 1133* (O) [KM-20]. **Pleasant Inlet**: *Saarela et al. 2677* (CAN) [PI-3].

##### *Kalmia* L.

***Kalmiaprocumbens* (L.) Gift, Kron & P.F.Stevens ex Galasso, Banfi & F.Conti.** (≡ *Loiseleuriaprocumbens* (L.) Desv.) (Fig. 15D)—Alpine azalea | Asian (NE)–Amphi-Beringian–North American (N)–Amphi-Atlantic–European (N)

Previously recorded in Kimmirut (Polunin 1940; Porsild 1957, 1964; Porsild and Cody 1980; Aiken et al. 2007). Newly recorded in the park and from Pleasant Inlet. Elsewhere on Baffin Island, recorded from Beekman Peninsula, Burwash Bay, Cormack Bay, Cornelius Grinnell Bay and Ogac Lake (Aiken et al. 2007). Not known elsewhere in the Canadian Arctic Archipelago.

**Katannilik Territorial Park**: *Saarela et al. 2562* (ALA, ALTA, CAN, MO, MT, O, WIN) [SF-6], *2612* (CAN, US, UVIC) [TJ-6]. **Kimmirut**: *Soper s.n.* (CAN, 2 ex), *Malte s.n.* [121041] (CAN), *Polunin 1130* (GH) [KM-1]. **Pleasant Inlet**: *Saarela et al. 2674* (CAN) [PI-3].

##### *Orthilia* Raf.

**Orthiliasecundasubsp.obtusata (Turcz.) Böcher** (≡ Pyrolasecundavar.obtusata Turcz.)—One-sided wintergreen | Asian (N/C)–Amphi-Beringian–North American

Newly recorded from the park, study area, Baffin Island and the eastern Canadian Arctic Archipelago. Gillespie et al. (2015) provide details.

**Katannilik Territorial Park**: *Saarela et al. 2489* (CAN) [EC-18].

##### *Phyllodoce* Salisb.

***Phyllodocecaerulea* (L.) Bab.** (Fig. 15E)—Purple mountain heather | North American (NE)–Amphi-Atlantic–European & Asian (C-NE)–Amphi-Beringian

Previously recorded in Kimmirut and the park (Porsild 1957, 1964; Porsild and Cody 1980; Aiken et al. 2007). Newly recorded from Pleasant Inlet. Elsewhere on Baffin Island, recorded from Amadjuak Bay, between Amadjuak Bay and Chorkbak Inlet, Beekman Peninsula, Cape Searle, Iqaluit, Jackman Sound (*Potter 8144*, MT00056278, *n.v.*), Ogac Lake, Penny Highlands, “Winton Bay Lake” (*Zimmerman 39b*, CAN 10074920) and York Sound (*Walker 826*, US-2311599; *Wynne-Edwards 7337*, CAN 10074912) (Aiken et al. 2007). Not otherwise known in the Canadian Arctic Archipelago.

**Katannilik Territorial Park**: *Soper s.n.* (CAN) [WR-1], *Saarela et al. 2034* (CAN, NYBG, US) [MJ-14], *2146* (CAN, UBC) [CR-7], *2563* (ALTA, CAN, MT) [SF-6]. **Kimmirut**: *Malte s.n.* [126856] (CAN), *s.n.* [119055] (CAN), *s.n.* [121014] (CAN), *Soper s.n.* (CAN), *Dutilly 1051*, *9133* (QFA), *Polunin 1119* (CAN) [KM-1], *Johansen 1130* (O) [KM-20]. **Pleasant Inlet**: *Saarela et al. 2676* (ALA, CAN, MO, O, WIN) [PI-3].

##### *Pyrola* L.

***Pyrolagrandiflora* Radius**—Large-flowered wintergreen | Circumpolar

Previously recorded in Kimmirut and the park (Polunin 1940; Porsild 1957, 1964; Porsild and Cody 1980; Aiken et al. 2007). Widespread across Baffin Island and elsewhere on southern Baffin Island, recorded from Amadjuak Bay, Dorset and Mallik islands, Griffen Bay (*Potter 8142*, MT00056280), Iqaluit, Lower Savage Islands, Resolution Island (*Dutilly 9281*, QFA0158242) and York Sound (*Wynne-Edwards 7311*, CAN 10074070) (Aiken et al. 2007; Saarela et al. 2020a).

**Katannilik Territorial Park**: *Soper s.n.* (CAN) [WR-1], *Saarela et al. 1921* (ALTA, CAN, MO, MT, WIN) [MJ-4], *2129* (CAN, O) [CR-12], *2357* (ALA, CAN) [LR-28]. **Kimmirut**: *Malte s.n.* [119036] (CAN), *s.n.* [119035] (CAN), *Soper s.n.* (CAN), *Dutilly 9110* (QFA) [KM-1], *Johansen 1126* (C) [KM-20].

##### *Rhododendron* L.

***Rhododendronlapponicum* (L.) Wahlenb.** (= R.lapponicumsubsp.alpinum (Glehn.) A.P.Khokhr.)—Lapland rosebay | Asian (NE)–Amphi-Beringian–North American (N)–Amphi-Atlantic (W)

Previously recorded in Kimmirut and the park (Polunin 1940; Porsild 1957, 1964; Porsild and Cody 1980; Aiken et al. 2007). Known from scattered sites across Baffin Island and elsewhere on southern Baffin Island, recorded from Amadjuak Bay, Brewster Point, Cormack Bay, Iqaluit and Mallik Island (Aiken et al. 2007; Saarela et al. 2020a).

**Katannilik Territorial Park**: *Soper s.n.* (CAN) [SF-1], *Soper s.n.* (CAN) [WR-1], *Saarela et al. 1958* (ALA, CAN, O) [MJ-3]. **Kimmirut**: *Malte s.n.* [120295] (CAN), *s.n.* [119061] (CAN), *Oldenburg 114* (MIN), *Polunin 471* (US), *Soper s.n.* (CAN), *Dutilly 9132* (QFA) [KM-1].

**Rhododendrontomentosumsubsp.decumbens (Aiton) Elven & D.F.Murray** (≡ *Ledumdecumbens* (Aiton) Lodd. ex Steud., ≡ L.palustrevar.decumbens Aiton, ≡ L.palustresubsp.decumbens (Aiton) Hultén; = *R.subarcticum* Harmaja, = R.tomentosumsubsp.subarcticum (Harmaja) G.D.Wallace)—Northern Labrador tea | Asian (N/C)–Amphi-Beringian–North American (N)

Previously recorded in Kimmirut and the park (Polunin 1940; Porsild 1957, 1964; Porsild and Cody 1980; Aiken et al. 2007). Widespread across Baffin Island and elsewhere on southern Baffin Island, recorded from Amadjuak Bay, Faris Island (*Aiken & McJanett 97-060*, CAN 10076574), Iqaluit, Perry Bay (*Jotcham s.n.*, ACAD-ECS004604), Winton Bay (*Zimmermann 33*, CAN 10076620) and York Sound (*Wynne-Edwards 7348*, CAN 10076607) (Aiken et al. 2007).

**Katannilik Territorial Park**: *Soper s.n.* (CAN) [WR-1], *Saarela et al. 1916* (ALA, CAN, MT, O, UBC, WIN) [MJ-4]. **Kimmirut**: *Malte s.n.* [119047] (CAN), *s.n.* [119043] (CAN, MT) [KM-1], *Johansen 1131* (O) [KM-20], *Archambault* AA266 (CAN) [KM-3].

##### *Vaccinium* L.

***Vacciniumuliginosum* L.** (= V.uliginosumsubsp.microphyllum (Lange) Tolm.)—Bilberry | Circumboreal-polar

Previously recorded in Kimmirut and the park (Polunin 1940; Porsild 1957, 1964; Porsild and Cody 1980; Aiken et al. 2007). Widespread across Baffin Island and elsewhere on southern Baffin Island, recorded from Brevoort Island (*Hurst 6*, CAN 10076628), Cape Haven, Cormack Bay (*Aiken 89-075*, CAN 10075169), Dorset and Mallik islands, Iqaluit and Lower Savage Islands (Aiken et al. 2007; Saarela et al. 2020a).

**Katannilik Territorial Park**: *Soper s.n.* (CAN) [WR-1], *Saarela et al. 1942* (ALA, CAN, O) [MJ-5], *2363* (CAN, MT, UBC, WIN) [LR-8]. **Kimmirut**: *Malte s.n.* [119062] (CAN) [KM-1], *Johansen 1129* (C) [KM-20].

**Vacciniumvitis-idaeasubsp.minus (Lodd., G.Lodd. & W.Lodd.) Hultén**—Mountain cranberry | Circumboreal-polar

Previously recorded in Kimmirut (Polunin 1940; Porsild 1957, 1964; Porsild and Cody 1980; Aiken et al. 2007). Newly recorded in the park. Widespread across Baffin Island and elsewhere on southern Baffin Island, recorded from Amadjuak Bay, Beekman Peninsula, Cormack Bay (*Aiken 89-069*, CAN 10076876), Dorset and Mallik islands, Iqaluit, Lower Savage Islands and Peter Force Island (*Wynne-Edwards 7483*, CAN 10076890) (Aiken et al. 2007; Saarela et al. 2020a).

**Katannilik Territorial Park**: *Saarela et al. 1923* (CAN, O) [MJ-4], *2425* (ALA, CAN, US, WIN) [EC-6], **Kimmirut**: *Malte s.n.* [120318] (CAN), *s.n.* [119066] (CAN, US) [KM-1], *Johansen 1128* (C) [KM-20].

**Boraginales**

#### Boraginaceae

##### *Mertensia* Roth

**Mertensiamaritimasubsp.tenella (Th.Fr.) Elven & Skarpaas**—Seaside bluebells | Amphi-Beringian–North American (N)–Amphi-Atlantic (W)

Previously recorded in Kimmirut (Porsild 1957, 1964; Porsild and Cody 1980; Aiken et al. 2007). Newly recorded from Pleasant Inlet. Known from scattered sites across Baffin Island and elsewhere on southern Baffin Island, recorded from Beekman Peninsula (*McLaren 9*, CAN 10077976), Brewster Point (*Walker 969*, US 02912568), Cormack Bay (*Aiken 89-035*, CAN 10077969), Dorset and Mallik islands, Foxe Peninsula near Wildbird Islands (*Manning 272*, CAN 10077959), Iqaluit and Ogac Lake (*Aiken & LeBlanc 04-087*, CAN 10077980) (Aiken et al. 2007; Saarela et al. 2020a).

**Kimmirut**: *Malte s.n.* [119117] (CAN) [KM-1]. **Pleasant Inlet**: *Saarela et al. 2687* (CAN, O) [PI-3], *2709* (ALA, ALTA, CAN, MT, UBC, US, UVIC, WIN) [PI-2].

**Lamiales**

#### Plantaginaceae

##### *Hippuris* L.

***Hippurislanceolata* Retz.**—Lance-leaved mare’s-tail | Circumpolar

Newly recorded in Kimmirut, the park, Pleasant Inlet and the study area. Aiken et al. (2007) mapped Malte’s Kimmirut collection as *H.vulgaris*; it has been redetermined as *H.lanceolata.* We follow the *Hippuris* taxonomy of Elven et al. (2011, 2012). Earlier range maps of these taxa in the Arctic are unreliable, given previous misunderstandings of species limits (Elven et al. 2011). Elsewhere on Baffin Island, recorded from Amadjuak Bay, Brewster Point, the head of Clyde Inlet, the head of Clearwater Fiord (formerly Kingua Fiord) at the head of Cumberland Sound, Iqaluit, Longstaff Bluff, Mallik Island and Nettilling Lake (Aiken et al. 2007; Saarela et al. 2020a).

**Katannilik Territorial Park**: *Saarela et al. 2189* (CAN) [GC-3]. **Kimmirut**: *Malte s.n.* [121005] (CAN, QFA, US) [KM-1]. **Pleasant Inlet**: *Saarela et al. 2717* (CAN, MT, UBC) [PI-1].

***Hippurisvulgaris* L.** (Fig. 15F, G)—Common mare’s-tail | Circumboreal

Newly recorded in Kimmirut, the park and the study area. The collection Aiken et al. (2007) mapped in Kimmirut as this species is *H.lanceolata*. Elsewhere on Baffin Island, recorded from the head of Clyde Inlet (*Wynne-Edwards 9082*, CAN 10073799) and Bylot Island (Aiken et al. 2007). See comments about *Hippuris* taxonomy above.

**Katannilik Territorial Park**: *Saarela et al. 2410* (ALTA, CAN, NYBG, US) [LC-3], *2444* (ALA, CAN, O, WIN) [EC-10], *2604* (CAN, O) [TJ-2]. **Kimmirut**: *Malte s.n.* (MT) [KM-1], *Johansen 1125* (C) [KM-20].

##### *Plantago* L.

***Plantagomaritima* L.** (= P.maritimasubsp.borealis (Lange) A.Blytt) (Fig. 15H)—Seaside plantain | Circumboreal-polar & South American (S)

Newly recorded from Pleasant Inlet and the study area. This species grew in a saline meadow along a small inlet below the high tide line with *Carexbicolor*, *C.subspathacea*, *C.ursina*, Puccinelliaphryganodessubsp.neoarctica, P.tenellasubsp.langeana and *Stellariahumifusa*. Elsewhere on Baffin Island, recorded from Brewster Point and Cormack Bay (Polunin 1939; Aiken et al. 2007) and not otherwise known from the Canadian Arctic Archipelago.

**Pleasant Inlet**: *Saarela et al. 2684* (ALA, CAN, O, WIN) [PI-3].

#### Lentibulariaceae

##### *Pinguicula* L.

***Pinguiculavulgaris* L.**—Common butterwort | Amphi-Pacific–North American–Amphi-Atlantic–European

Previously recorded in Kimmirut and the park (Polunin 1940; Porsild and Cody 1980; Aiken et al. 2007). Gillespie et al. (2015) reported our collections from the study area. Elsewhere on Baffin Island, known from Ogac Lake (Aiken et al. 2007) and elsewhere in the Canadian Arctic Archipelago, known from Victoria Island (Porsild and Cody 1980; Gillespie et al. 2015; Saarela et al. 2020b).

**Katannilik Territorial Park**: *Aiken & Iles 02-053* (CAN) [LS-1], *Saarela et al. 2264* (CAN) [LR-20], *2381* (CAN, MO) [LR-7], *2478* (ALA, CAN, WIN) [EC-14], *2531* (CAN) [SF-28], *2565* (CAN, O) [SF-5]. **Kimmirut**: *Polunin 2348* (CAN) [KM-1], *Saarela et al. 2787* (CAN) [KM-13].

##### *Utricularia* L.

***Utriculariaochroleuca* R.W.Hartm.**—Yellowish-white bladderwort | Circumboreal?

Newly recorded for the park, study area, Canadian Arctic Archipelago and Nunavut. Gillespie et al. (2015) provide details.

**Katannilik Territorial Park**: *Saarela et al.* 2464 (ALA, ALTA, CAN, MT, NYBG, O, UBC, US, WIN) [EC-1].

#### Orobanchaceae

##### *Bartsia* L.

**Figure 16.**
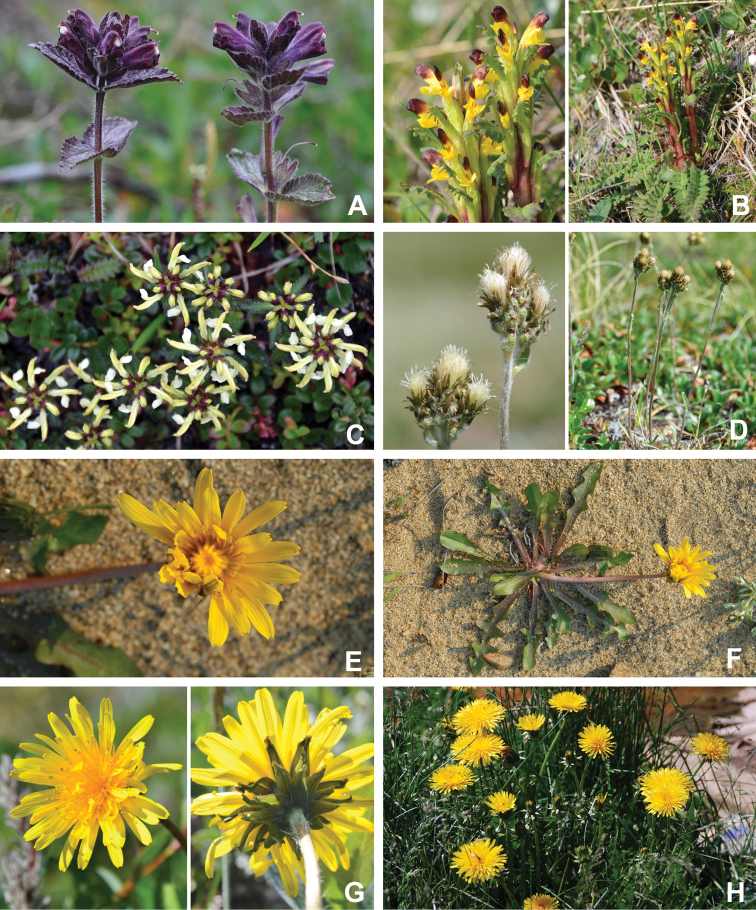
**A***Bartsiaalpina* inflorescences, *Saarela et al. 2258***B***Pedicularisflammea* inflorescences (left) and habit (right), *Saarela et al. 2422***C***Pedicularislapponica* inflorescences, 11 July 2012 **D**Antennariafriesianasubsp.friesiana inflorescences (left) and habit (right), *Saarela et al. 1920***E***Taraxacumholmenanium* inflorescence, *Saarela et al. 2420***F***Taraxacumholmenanium* habit, *Saarela et al. 2420***G***Taraxacumlapponicum* inflorescences, *Saarela et al. 2596***H***Taraxacumlapponicum* habit, *Saarela et al. 2756*. Photos **A, E, F** by L.J. Gillespie, **B–D, G** by R.D. Bull and **H** by J.M. Saarela.

***Bartsiaalpina* L.** (Fig. 16A)—Alpine bartsia | North American (NE)–Amphi-Atlantic–European (N/C)

Previously recorded in Kimmirut and the park (Polunin 1940; Porsild 1957, 1964; Aiken et al. 2007). Elsewhere on Baffin Island, recorded from Brewster Point (*Potter 8044*, GH 02033421), Jackman Sound (*Potter 8043*, GH 02033420), Ogac Lake and York Sound (Aiken et al. 2007).

**Katannilik Territorial Park**: *Aiken & Iles 02-054* (CAN) [LS-1], *Saarela et al. 2258* (CAN, MT, NFM, WTU) [LR-20], *2421* (CAN) [EC-7], *2483* (ALTA, CAN, GH, MIN, MO, UVIC) [EC-16], *2536* (ALA, CAN, O, QFA, WIN) [SF-10]. **Kimmirut**: *Malte s.n.* [119137] (CAN, MT), *s.n.*/*1166* [121008] (CAN, GH), *Soper s.n.* (CAN), *Polunin 416* (GH), *1154* (CAN), *1464* (US) [KM-1], *Saarela et al. 2667* (CAN, NYBG, UBC) [KM-9].

##### *Pedicularis* L.

***Pedicularisflammea* L.** (Fig. 16B)—Red-tipped lousewort | North American (N)–Amphi-Atlantic (W)

Previously recorded in Kimmirut and the park (Polunin 1940; Porsild 1957, [Bibr B94][Bibr B95]; Aiken et al. 2007). Widespread across Baffin Island as far north as Clyde Inlet and elsewhere on southern Baffin Island, recorded from between Amadjuak Bay and Chorkbak Inlet, Brewster Point (*Potter 8038*, GH 02080709), Dorset and Mallik islands, Foxe Peninsula near Wildbird Islands, near Griffen Bay (*Potter 8037*, GH 02080626), Iqaluit, Jackman Sound (*Potter 8036*, GH 0208710), Lower Savage Islands, Ogac Lake, Perry Bay and Resolution Island (Aiken et al. 2007; Saarela et al. 2020a).

**Katannilik Territorial Park**: *Soper s.n.* (CAN, 2 ex) [WR-1], *Aiken & Iles 02-048* (CAN) [CR-1], *Saarela et al. 1969* (CAN, MIN) [MJ-8], *2210* (ALA, CAN, O, WIN) [GC-8], *2323* (CAN) [LR-14], *2422* (CAN, QFA) [EC-7]. **Kimmirut**: *Malte s.n.*/*446* [120287] (CAN, GH, MT), *s.n.* [126882] (CAN, GH), *s.n.* [119159] (CAN), *s.n.* [119158] (CAN), *s.n.* [119157] (CAN), *s.n.* [119155] (CAN), *Oldenburg 109* (MIN), *Soper s.n.* (CAN), *Polunin 454* (GH), *410* (CAN), *Dutilly 1045a* (CAN), *9105* (QFA), *9107* (QFA) [KM-1], *Saarela et al. 2665* (CAN, MT) [KM-9], *2750* (ALTA, CAN) [KM-11].

***Pedicularishirsuta* L.**—Hairy lousewort | Circumpolar

Previously recorded in the park (Aiken et al. 2007). Widespread across Baffin Island and elsewhere on southern Baffin Island, recorded from between Amadjuak Bay and Chorkbak Inlet, Bowdoin Harbour [Schooner Harbour] (*Robinson 4a*, GH 02079070), Chorkbak Inlet, Dorset Island, Iqaluit, Jackman Sound (*Potter 8032*, GH 02079055), Ogac Lake (*Aiken & LeBlanc 04*-*226*, CAN 10080912), Silliman’s Fossil Mount and York Sound (*Walker 836*, CAN 10080930; *Wynne-Edwards 7263*, CAN 10080943) (Aiken et al. 2007; Saarela et al. 2020a).

**Katannilik Territorial Park**: *Soper s.n.* (CAN, 2 ex) [WR-1], *Saarela et al. 2057* (CAN) [MJ-16], *2067* (CAN) [MJ-43], *2075* (ALA, CAN, WIN) [MJ-34], *2317* (CAN, O, QFA) [LR-15].

***Pedicularislabradorica* Wirsing**—Labrador lousewort | Asian (N/C)–Amphi-Beringian–North American (N)

Previously recorded in Kimmirut and the park (Polunin 1940; Porsild and Cody 1980; Porsild 1957, 1964; Aiken et al. 2007). Elsewhere on Baffin Island, recorded from Cormack Bay and Iqaluit (head of Tarr Inlet) (Porsild and Cody 1980; Aiken et al. 2007). Porsild and Cody (1980) mapped an additional site in the Amadjuak Bay area for which we are unaware of a voucher.

**Katannilik Territorial Park**: *Soper s.n.* (CAN) [WR-1], *Aiken & Iles 02-043 b* (CAN) [MJ-1], *Saarela et al. 1941* (CAN, O), *1945* (ALA, CAN, QFA, WIN) [MJ-5]. **Kimmirut**: *Polunin 1182* (GH) [KM-1], *Johansen 1135* (C) [KM-20].

***Pedicularislanata* Willd. ex Cham. & Schltdl.**—Woolly lousewort | Amphi-Beringian–North American (N)

Previously recorded in Kimmirut and the park (Polunin 1940; Porsild 1957, 1964; Porsild and Cody 1980; Aiken et al. 2007). Widespread across Baffin Island and elsewhere on southern Baffin Island, recorded from Bowdoin Harbour [Schooner Harbour] (*Soper s.n.*, CAN 10080275), between Amadjuak Bay and Chorkbak Inlet (*Bell s.n.*, CAN 10081156), Amadjuak Lake, Brewster Point, Dorset and Mallik islands and Ukiurjak (formerly King Charles Cape) (Aiken et al. 2007; Saarela et al. 2020a).

**Katannilik Territorial Park**: *Soper s.n.* (CAN) [SF-1], *Saarela et al. 2265* (CAN, QFA, WIN) [LR-23], *2479* (ALTA, CAN) [EC-14], *2514* (CAN, MT) [SF-15]. **Kimmirut**: *Malte s.n.* [120286] (CAN), *s.n.* [126854] (CAN, GH), *Oldenburg 107* (MIN), *116* (MIN), *Soper s.n.* (CAN), *Dutilly 1045* (CAN) [KM-1], *Archambault AA254* (CAN) [KM-4], *Saarela et al. 2792* (ALA, CAN, O) [KM-12].

***Pedicularislapponica* L.** (Fig. 16C)—Lapland lousewort | Circumpolar-alpine

Previously recorded in Kimmirut and the park (Polunin 1940; Porsild 1957, 1964; Porsild and Cody 1980; Aiken et al. 2007). Aiken et al. (2007) mapped Soper’s inland collection, but not the multiple Kimmirut records. Elsewhere on Baffin Island, recorded from Amadjuak Bay, between Amadjuak Bay and Chorkbak Inlet, Burwash Bay, Cormack Bay, Cumberland Gulf, near Griffin Bay (*Potter 8031*, GH 02079627), Ogac Lake (*Aiken & LeBlanc s.n.*, CAN 10080546) and Ward Inlet (Polunin 1940; Aiken et al. 2007).

**Katannilik Territorial Park**: *Soper s.n.* (CAN) [WR-1], *Aiken & Iles 02-021* (CAN) [MJ-1], *Saarela et al. 1946* (ALA, CAN, O) [MJ-5], *2564* (CAN, MT, QFA) [SF-5]. **Kimmirut**: *Malte s.n.* [126883] (CAN, GH), *s.n.* [119179] (CAN), *s.n.* [119177] (CAN, GH), *s.n.* [119176] (CAN, GH), *s.n.* [119178] (CAN, GH), *Oldenburg 77* (MIN), *Soper s.n.* (CAN), *Polunin 430*, *1160*, *1243* (GH), *Dutilly 9104* (QFA) [KM-1], *Saarela et al. 2780* (CAN) [KM-19].

**Asterales**

#### Campanulaceae

##### *Campanula* L.

***Campanularotundifolia* L.**—Harebell | Circumboreal-polar

Newly recorded in the park and the study area. Aiken et al. (2007) mapped a record in the Kimmirut area, based on Porsild and Cody’s (1980) map; however, this was an error, as there is no such record in Porsild and Cody (1980). This species grew along the edge of a large, hummocky sedge meadow with *Anthoxanthummonticola*, *Carexarctogena*, *C.bigelowii*, *Poaarctica* and *Stellarialongipes* and on the lower slopes of ridges at the edge of a low willow thicket with *Anthoxanthummonticola*, *Salixuva-ursi*, *Saxifragatricuspidata* and *Vacciniumuliginosum*. Elsewhere on Baffin Island, known from scattered sites on the Hall and Cumberland Peninsulas, Beekman Peninsula, Brewster Point, Newell Sound and Ogac Lake (Aiken et al. 2007). Not known elsewhere in the Canadian Arctic Archipelago. Elven et al. (2011) summarized the considerable variation within the species. Lammers (2007) treated Canadian plants called *C.rotundifolia* as *C.gieseckeana* Vest ex Schult.

**Katannilik Territorial Park**: *Saarela et al. 2401* (CAN, WIN) [LC-3], *2448* (ALA, CAN, O) [EC-8].

##### *Melanocalyx* Morin

***Melanocalyxuniflora* (L.) Morin** (≡ *Campanulauniflora* L.)—Arctic bellflower | Amphi-Beringian–North American (N)–Amphi-Atlantic

Previously recorded in Kimmirut (Polunin 1940; Porsild 1957, 1964; Porsild and Cody 1980; Aiken et al. 2007). Newly recorded in the park. Widespread on Baffin Island and elsewhere on southern Baffin Island, recorded from Amadjuak Bay, Bowdoin Harbour [Schooner Harbour] (*Soper s.n.*, CAN 10082068), Dorset and Mallik islands, Iqaluit, Pritzler Harbour and Resolution Island (Aiken et al. 2007; Saarela et al. 2020a). Taxonomy follows Morin (2020).

**Katannilik Territorial Park**: *Saarela et al. 1922* (ALA, CAN, O, WIN) [MJ-4], *2400* (ALTA, CAN, MO, MT, UBC, US) [LC-3]. **Kimmirut**: *Malte s.n.* [120291] (CAN), *Soper s.n.* (CAN), *Polunin 1253* (CAN), *Dutilly 1050* (O [as *1050a*], QFA) [KM-1].

#### Asteraceae

##### *Antennaria* Gaertn.

**Antennariaalpinasubsp.canescens (Lange) Chmiel.** (≡ *A.canescens* (Lange) Malte)—Alpine pussytoes | Amphi-Beringian (E)?–North American–Amphi-Atlantic (W)

Previously recorded in Kimmirut (Polunin 1940; Aiken et al. 2007). Newly recorded in the park. Elsewhere on Baffin Island, recorded from Beekman Peninsula, Iqaluit, Newell Sound (*McLaren 61*, CAN 10082580, *McLaren 62*, CAN 10082585), Ogac Lake and a few sites further north (Aiken et al. 2007).

**Katannilik Territorial Park**: *Saarela et al. 2081* (ALA, CAN, UBC) [MJ-33], *2167* (CAN, WIN) [CR-4], *2307* (CAN) [LR-5], *2524* (CAN) [SF-24], *2552* (CAN, MT, O) [SF-11]. **Kimmirut**: *Malte s.n.* [119192] (CAN), *Polunin 1258* (CAN) [KM-1], *Saarela et al. 2733* (CAN, MT) [KM-7], *2777* (CAN, QFA) [KM-19].

**Antennariafriesiana(Trautv.)E.Ekmansubsp.friesiana** (= *A.ekmaniana* A.E.Porsild) (Fig. 16D)—Fries’ pussy-toes | Asian (NE)–Amphi-Beringian–North American (N)

Previously recorded in the park (Polunin 1940; Aiken et al. 2007). Not known from Kimmirut. Widespread across Baffin Island and elsewhere on southern Baffin Island, recorded from Amadjuak Bay (*Bell s.n.*, CAN 10083285), Dorset Island, Iqaluit, Newell Sound (*McLaren 63*, CAN 10082491) and Ogac Lake (Aiken et al. 2007; Saarela et al. 2020a).

**Katannilik Territorial Park**: *Aiken & Iles 02-044* (CAN) [MJ-1], *Saarela et al. 1920* (CAN, GH, MIN) [MJ-4], *2039* (CAN) [MJ-23].

**Antennariamonocephalasubsp.angustata (Greene) Hultén** (≡ *A.angustat*a Greene)—Pygmy pussy-toes | Amphi-Beringian–North American (N)

Previously recorded in Kimmirut and the park (Polunin 1940; Aiken et al. 2007). Recorded on Baffin Island north to the Clyde River area and elsewhere on southern Baffin Island, recorded from Dorset Island, Iqaluit, Newell Sound, Ogac Lake, Resolution Island, Silliman’s Fossil Mount and Ukiurjak (formerly King Charles Cape) (Aiken et al. 2007; Saarela et al. 2020a).

**Katannilik Territorial Park**: *Soper s.n.* (CAN) [WR-1], *Aiken & Iles 02-049* (CAN) [LR-2], *02-057* (CAN) [LS-1], *Saarela et al. 2166* (ALTA, CAN, MO, MT, UVIC, WTU) [CR-4], *2306* (CAN) [LR-5], *2424* (CAN, MT, NYBG, UTC) [EC-7], *2449* (CAN, MT, US) [EC-8], *2554* (CAN, MT) [SF-11]. **Kimmirut**: *Malte s.n.* [119193] (CAN), *s.n.* [119191] (CAN), *s.n.* [119190] (CAN), *Polunin 2308* (CAN), *Dutilly 991* (CAN) [KM-1], *Saarela et al. 2668* (CAN) [KM-9], *2779* (CAN) [KM-19].

##### *Arnica* L.

**ArnicaangustifoliaVahlsubsp.angustifolia**—Alpine arnica | North American (N)–Amphi-Atlantic (W)

Previously recorded in Kimmirut and the park (Polunin 1940; Aiken et al. 2007). Known from scattered sites across Baffin Island and elsewhere on southern Baffin Island, recorded from Amadjuak Bay, Beekman Peninsula, Brewster Point (*Wynne-Edwards 7404*, CAN 10082858; *Wynne-Edwards 7364*, CAN 10082859), Iqaluit, Ogac Lake and Silliman’s Fossil Mount (Aiken et al. 2007). Porsild and Cody (1980) mapped sites on Foxe Peninsula for which we are unaware of vouchers.

**Katannilik Territorial Park**: *Soper s.n.* (CAN, 2 ex) [WR-1], *Saarela et al. 1956* (CAN, MO, NFM, NYBG, QFA, UBC, US) [MJ-10], *2139* (ALTA, CAN, WIN) [CR-8]. **Kimmirut**: *Dutilly 1006* (CAN), *1007* (QFA), *Malte s.n.* [119195] (CAN) [KM-1], *Saarela et al. 2784* (ALA, CAN, MT, O) [KM-19].

##### *Artemisia* L.

**ArtemisiaborealisPallassubsp.borealis**—Boreal wormwood | European (NE)–Asian (N/C)–Amphi-Beringian–Cordilleran–North American (N)

Previously recorded in Kimmirut and the park (Polunin 1940; Aiken et al. 2007). Elsewhere on Baffin Island, recorded from Beekman Peninsula, Brewster Point (*Wynne-Edwards 7403*, CAN 10083894), Iqaluit, Ogac Lake (*Aiken & LeBlanc 04-211*, CAN 10083901) and York Sound (*Wynne-Edwards 7266*, CAN 10083890) (Aiken et al. 2007).

**Katannilik Territorial Park**: *Aiken & Iles 02-043* (CAN), *02-043a* (CAN), *02-045* (CAN) [MJ-1], *Saarela et al. 1966* (CAN, GH, MIN, NFM, QFA, WTU) [MJ-11], *2391* (CAN, MT, O) [LR-29], *2392* (ALA, ALTA, CAN, MT, O, UBC, US, WIN) [LR-29]. **Kimmirut**: *Malte s.n.* [119196] (CAN, QFA), *Polunin 1239* (US), *1250* (CAN) [KM-1], *Saarela et al. 2747* (CAN, MO, NYBG) [KM-11].

##### *Erigeron* L.

***Erigeroneriocephalus* J.Vahl** (≡ E.uniflorussubsp.eriocephalus (J.Vahl) Cronquist)—Woolly-headed fleabane | Circumpolar

Newly recorded in the park and study area. Widespread on Baffin Island and elsewhere on southern Baffin Island, recorded from Beekman Peninsula, Dorset and Mallik islands, Foxe Peninsula near Wildbird Islands, Iqaluit, Ogac Lake and York Sound (Aiken et al. 2007; Saarela et al. 2020a).

**Katannilik Territorial Park**: *Saarela et al.* 2231 (ALA, CAN, O, WIN) [WR-4], *2578* (CAN, MT) [SF-3].

***Erigeronhumilis* Graham**—Low fleabane | Amphi-Beringian–North American (N)–Amphi-Atlantic (W)

Previously recorded in Kimmirut and the park (Polunin 1940; Aiken et al. 2007). Newly recorded from Pleasant Inlet. Known from scattered sites across Baffin Island and elsewhere on southern Baffin Island, recorded from Dorset and Mallik islands, Foxe Peninsula near Wildbird Islands, Iqaluit, Ogac Lake and York Sound (Aiken et al. 2007; Saarela et al. 2020a).

**Katannilik Territorial Park**: *Aiken & Iles 02-042 a* (CAN) [MJ-1], *Saarela et al. 2215* (CAN) [GC-8], *2229* (CAN, UBC, WIN) [WR-4], *2296* (CAN) [LR-27], *2430* (CAN, US) [EC-7], *2512* (ALTA, CAN) [SF-15], *2523* (CAN) [SF-24], *2620* (ALA, CAN, O) [TJ-1]. **Kimmirut**: *Malte s.n.* [119200], *s.n.* [119199] (CAN, MT, QFA), *Polunin 1251* (US), *Dutilly 1041* (QFA), *Soper s.n.* (CAN) [KM-1], *Archambault AA261* (CAN) [KM-4]. **Pleasant Inlet**: *Saarela et al. 2723* (CAN, MT) [PI-1].

##### *Hulteniella* Tzvelev

***Hulteniellaintegrifolia* (Richardson) Tzvelev** (≡ *Chrysanthemumintegrifolium* Richardson)—Small arctic daisy | Amphi-Beringian–North American (N)

Previously recorded in Kimmirut and the park (Polunin 1940; Aiken et al. 2007). Widespread across Baffin Island and elsewhere on southern Baffin Island, recorded from Amadjuak Bay, Bowdoin Harbour [Schooner Harbour] (*Soper s.n.*, CAN 10084624), Dorset and Mallik islands, Foxe Peninsula near Wildbird Islands, Iqaluit and Silliman’s Fossil Mount (Aiken et al. 2007; Saarela et al. 2020a).

**Katannilik Territorial Park**: *Aiken & Iles 02-055* (CAN) [SF-2], *Saarela et al. 2482* (CAN, UBC, WIN) [EC-16], *2507* (CAN, O) [SF-15], *2548* (ALA, CAN, MT) [SF-13]. **Kimmirut**: *Dutilly 998* (CAN, QFA), *9112* (CAN), *Polunin 407* (CAN) [KM-1].

##### *Taraxacum* F.H.Wigg., nom. cons.

*Taraxacum* taxonomy follows Brouillet (2006).

***Taraxacumceratophorum* (Ledeb.) DC.** (= *T.lacerum* Greene, = *T.malteanum* Dahlstedt)—Horned dandelion | Circumboreal-polar

Previously recorded in Kimmirut (Polunin 1940; Aiken et al. 2007). Newly recorded in the park. Known from scattered sites across Baffin Island and elsewhere on southern Baffin Island, recorded from Amadjuak Bay (*Soper s.n.*, CAN 10089548), Brewster Point, Dorset and Mallik islands, Iqaluit, Lower Savage Islands (*Gillespie et al. 6742*, CAN 10085083) and Ogac Lake (Aiken et al. 2007; Saarela et al. 2020a).

**Katannilik Territorial Park**: *Saarela et al. 1953* (CAN) [MJ-10], *2063* (CAN, MT) [MJ-30], *2089* (CAN) [MJ-38]. **Kimmirut**: *Malte s.n.* [119207] (S), *s.n.* [120293] (S), *s.n.* (CAN, 2 ex [KM-1].

***Taraxacumholmenianum* Sahlin** (Fig. 16E, F)—Holmen’s dandelion | North American (N)

Newly recorded in the park and study area. The species grew at the base of a steep sandy riverbank near the mouth of a small creek. Elsewhere on Baffin Island, recorded from the head of Clyde Inlet, Inugsuin Fiord, Isortoq River and Resolution Island (Aiken et al. 2007).

**Katannilik Territorial Park**: *Saarela et al. 2420* (CAN) [EC-7].

***Taraxacumlapponicum* Kihlman ex Hand.-Mazz.** (Fig. 16G, H)—Lapland dandelion

Previously recorded in Kimmirut (Polunin 1940; Porsild 1957, 1964; Porsild and Cody 1980). Newly recorded in the park. Elsewhere on Baffin Island, recorded from Beekman Peninsula (*McLaren 70*, CAN 10085394; *McLaren 122*, CAN 10085392), Iqaluit (*Wynne-Edwards 9313*, CAN 10085391), Newell Sound (*McLaren 37*, CAN 10085398), Ogac Lake (*Gillespie et al. 6751*, CAN 10001164, *6754*, CAN 10001163) and York Sound (*Wynne-Edwards 7335*, CAN 10085393).

**Katannilik Territorial Park**: *Saarela et al. 2117* (CAN, MO) [MJ-39], *2205* (CAN, WIN) [GC-9], *2348* (ALA, CAN, MT, O) [LR-35], *2367* (CAN, MO, MT, UTC, WTU) [LR-9], *2408* (CAN) [LC-3], *2433* (ALTA, CAN, UBC) [EC-9], *2502* (CAN, GH, MIN, QFA) [LS-2], *2537* (CAN, NFM) [SF-10], *2577* (CAN, US) [SF-18], *2596* (CAN) [TJ-1]. **Kimmirut**: *Malte s.n.* [119208] (CAN), *Polunin 1727* (US), *2300* (CAN) [KM-1], *Saarela et al. 2664* (ALA, CAN, MT, O) [KM-18], *2756* (ALA, ALTA, CAN, MICH, MO, MT, NYBG, O, UBC, US, WIN, WLU) [KM-15], *2789* (CAN, MIN, QFA) [KM-19].

### ﻿Excluded taxa

***Cerastiumbeeringianum*** Cham. & Schltdl.—Aiken et al. (2007) mapped a Soper collection (CAN-52230) from Koukdjuak River [Soper River]. We were unable to locate this voucher. The species is otherwise known from scattered sites across southern Baffin Island, including Upper Savage Islands (*Bell 34354*, CAN 10046312) (Aiken et al. 2007).

***Eriophorumrusseolum*** Fr. ex Hartm.—Polunin (1940) reported Eriophorumchamissonisf.albidum (F.Nyl.) Fernald from inland of Lake Harbour. Porsild and Cody (1980) mapped E.russeolumvar.albidum Nyl. in the Kimmirut area. Aiken et al. (2007) mapped E.russeolumsubsp.leiocarpum M.S.Novos. from the study area vicinity area, based on Porsild and Cody’s (1980) map. We have not seen vouchers that support these records.

***Euphrasiawettsteinii*** G.L.Gusarova—Polunin (1940) reported *Euphrasiaarctica* Lange ex Rostr. in Lake Harbour, based on his collection no. 2347 taken in 1936, and some later treatments mapped the record (Porsild 1957; Porsild and Cody 1980). Aiken et al. (2007) did not map the record from the study area. We have not seen a voucher. The name *E.arctica* has been misapplied in the Canadian Arctic. Plants on Baffin Island are now ascribed to *E.wettsteinii*.

***Puccinelliaangustata*** (R.Br.) E.L.Rand & Redfield—Polunin (1940) reported this taxon in Lake Harbour, based on his 1934 and 1936 observations. In 1953, Sorensen redetermined a 1936 collection (*Polunin 1163*, CAN), which Polunin had determined as P.vaginatavar.paradoxa T.J.Sørensen (= *P.vaginata*) as this species. J.M. Saarela confirmed the identification as *P.vaginata* in 2012. A 1934 collection (*Polunin 701*, F-892992, MICH 1422985) identified as *P.angustata* requires physical examination to confirm its identity. If confirmed, this would be the southernmost record of the taxon on Baffin Island, based on the map in Aiken et al. (2007).

**Tephroserispalustrissubsp.congesta** (R.Br.) Holub—Porsild and Cody (1980) mapped this species in the study area. Aiken et al. (2007) mapped it (erroneously) just north of the study area, based on the map in Porsild and Cody (1980). We have not seen a supporting voucher.

## Supplementary materials

Supplementary material 1 Dataset of herbarium specimens documenting the vascular plant flora of Kattanilik Territorial Park and Kimmirut and vicinity, Baffin Island, Nunavut, Canada Data type occurrences https://binary.pensoft.net/file/790806This dataset is made available under the Open Database License (http://opendatacommons.org/licenses/odbl/1.0/). The Open Database License (ODbL) is a license agreement intended to allow users to freely share, modify, and use this Dataset while maintaining this same freedom for others, provided that the original source and author(s) are credited.Jeffery M. Saarela, Paul C. Sokoloff, Lynn J. Gillespie, Roger D. Bull
